# Copper-catalyzed direct C–H arylselenation of 4-nitro-pyrazoles and other heterocycles with selenium powder and aryl iodides. Access to unsymmetrical heteroaryl selenides†

**DOI:** 10.1039/c9ra05004c

**Published:** 2019-08-13

**Authors:** Michał Jakubczyk, Satenik Mkrtchyan, Izabela D. Madura, Paulina H. Marek, Viktor O. Iaroshenko

**Affiliations:** Laboratory of Homogeneous Catalysis and Molecular Design at the Centre of Molecular and Macromolecular Studies, Polish Academy of Sciences Sienkiewicza 112 PL-90-363 Łodź Poland iva108@gmail.com viktori@cbmm.lodz.pl; Department of Inorganic Chemistry, Faculty of Chemistry, Warsaw University of Technology Noakowskiego 3 00-664 Warsaw Poland; Faculty of Chemistry, University of Warsaw Pasteura 1 02-093 Warsaw Poland

## Abstract

A one-pot, Cu-catalyzed direct C–H arylselenation protocol using elemental Se and aryl iodides was developed for nitro-substituted, *N*-alkylated pyrazoles, imidazoles and other heterocycles including 4*H*-chromen-4-one. This general and concise method allows one to obtain a large number of unsymmetrical heteroaryl selenides bearing a variety of substituents. The presence of the nitro group was confirmed to be essential for the C–H activation and can also be used for further functionalisation and manipulation. Several examples of heteroannulated benzoselenazines were also synthesized using the developed synthetic protocol.

## Abstract 2

In this work, we elaborated a general and straightforward method which permits the rapid assembly of unsymmetrical heteroaryl-aryl selenides containing 4-nitropyrazole, 4-nitroimidazole and a few other heterocyclic scaffolds.

## Graphical abstract

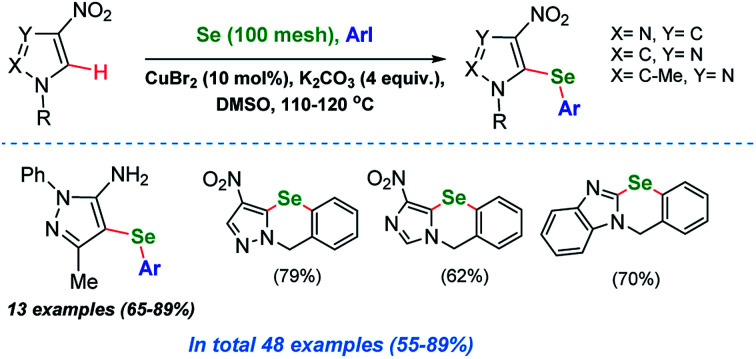

## Introduction

Organoselenium compounds have been known for many years to have biological activity that can be useful in medicine.^1^ Their therapeutic properties include antitumor,^2^ antioxidant,^3,4^ antiinflammatory, antiviral, antimicrobial and neuroprotective action.^5^ Among these, the antioxidant effect seems to be the most important mode of action recognized for organoselenium therapeutics as the oxidative stress is a symptom and often the cause of many diseases. Mammalian organisms are equipped with glutathione peroxidases (GPx)^6,7^ – a family of antioxidant enzymes that naturally contain selenium in their active site in the form of a selenocysteine^8^ (Sec) residue. One of the most thoroughly studied synthetic drugs that demonstrates the so called GPx-like activity^9^ – ebselen, is often considered a comparative standard when investigating other organoselenium drug candidates.^10–12^

Diorganyl selenides receive much attention, both due to their biological activity^13–17^ and their value in synthetic chemistry.^18–24^ Non-cyclic diorganyl selenides can be grouped, according to the structure of the organyl substituents, as symmetrical and unsymmetrical compounds. This nominal division, present in the literature comes from the varying difficulty and complexity of the synthetic methodology. In a historical context, sodium or potassium selenides (Na_2_Se, K_2_Se) or polyselenides (Na_2_Se_*x*_ or K_2_Se_*x*_) can be reacted with bromides to yield symmetrical dialkyl selenides^25–27^ or with arenediazonium salts to prepare symmetrical diaryl selenides.^28–34^ In a more modern context, TM-catalyzed cross-coupling reactions are one of the most common methods for the preparation of aryl chalcogenides.^35^ Particularly, copper-catalyzed methods utilizing: elemental selenium with aryl halides,^36–38^ potassium selenocyanates with aryl halides,^39,40^ triarylbismuthanes with elemental selenium^41^ or boronic acids with diaryl diselenides,^42^ elemental selenium^43^ or selenourea^44^ – can be applied. Some of these methods yield also diselenides as by- or main-products, depending on the reaction conditions. Unsymmetrical diaryl selenides can be obtained by similar methods from elemental selenium,^36,38^ diselenides^45–48^ and selenols^49,50^ as a selenium source. Additionally, two other methods have been developed. Nucleophilic substitution of bromine in PhSeBr by mild nucleophiles (arylboronic acids, arylsiloxanes, and arylstannanes) catalyzed by alumina-supported copper catalyst^51^ and a transition metal-free, base promoted reaction of arylhydrazines with diaryl diselenides.^52^

Unsymmetrical aryl-Se-heteroaryl compounds represent yet another advanced challenge as synthetic targets. Structures bearing pyridine and thiophen moieties at selenium are available by previously mentioned methods from diselenides.^53–55^ Special attention should be paid to those reagents in the context of unsymmetrical heteroaryl selenides' synthesis. In a recent review, Arsenyan summarizes the progress in this matter, listing all the possible synthetic pathways to obtain aryl-Se-heteroaryl compounds (bearing a variety of pharmacophores) from diaryl diselenides.^56^ These transformations include nucleophilic and electrophilic reactions (synthons RSe^+^ and RSe^−^), reactions involving radicals (RSe˙), copper catalysis, single electron transfer (SET) reactions (photochemical reactions) and direct heteroaryl selenation of activated C(sp^2^)–H bond.

The C–H bond is the most widespread structural fragment in organic chemistry, and its functionalization has been the subject of intensive studies.^67–71^ In recent years, TM-catalyzed C–H activation reactions emerged as one of the most important methodologies in modern organic chemistry. This applies also to selenation. The TM-catalyzed direct arylselenation of C–H bonds is one of the most efficient methods for the synthesis of unsymmetrical diaryl selenides. The to-date developed protocols involve the use of diselenides (palladium,^63^ rhodium,^72^ ruthenium^64,73^ iron oxide,^74^ silver,^75,76^ and copper^65,66,77^ catalyzed) or ArSeCl (ruthenium^78^ catalyzed) as selenium source. There are also a few very recent examples of copper-catalyzed one-pot three-component procedures involving Se powder, which can be considered an obvious step in methodology development, since diselenides are easily obtained from aryl iodides and selenium powder in similar catalytic conditions.^37^

Wu *et al.* obtained a library of (phenylseleno)-1*H*-indoles selecting CuO as the best catalyst.^61^ Their protocol is also suitable for the formation of an intramolecular C–Se–C bond. The authors pointed out that the free NH group of the indol was critical as the *N*-substituted starting materials did not undergo arylselenation in selected conditions. However, Guo *et al.* showed that also *N*-substituted indoles can undergo C–H arylselenation in specific conditions.^59^ In the same paper, the authors revisit the selenation of imidazo[1,2-*a*]pyridines, previously submitted to reaction with 4-coumarinyl triflates.^60^ 2-(2-Bromophenyl)imidazo[1,2-*a*]pyridines were also explored as starting materials in an intramolecular variant of the reaction by Wang *et al.*^58^ In another paper, Wu *et al.* proposed conditions for C–H arylselenation of 2-phenyl- and 2-aryl-1,3,4-oxadiazoles,^62^ a scaffold found in many drugs.^79^ Slightly different conditions for the same starting materials were also selected by Braga *et al.*^57^

Since we have our long-standing interest in TM-catalyzed C–H activation, we were curious if a general, practical and concise approach could be realized for the arylselenation of other heterocycles – widening the scope of the starting materials. The regioselectivity of C–H activation reactions of more complex functionalized substrates containing two or more reactive C–H bonds is an important topic of current research.^67–71,80–90^ The regioselectivity of such reactions is controlled by the presence of functional groups in the substrate. This includes directing substituents (carbonyl, cyano, *etc.*),^80–83,85,88–90^ halogen substituents (mostly fluorine or chlorine),^80–83,85,88–93^ and ring heteroatoms (namely – sulfur, nitrogen, and oxygen). The use of the nitro group as a regio-directing substituent in C–H activations has been investigated previously^94,95^ also by our group in the context of arylation of nitro-pyrazoles,^96^ nitro-imidazoles^97^ and other nitro-heteroarenes.^98^ Herein we describe a one-pot method to obtain unsymmetrical diorganyl selenides from C–H activated heterocycles (including nitro-substituted), aryl iodides and elemental selenium powder.

## Results and discussion

**Scheme 1:**
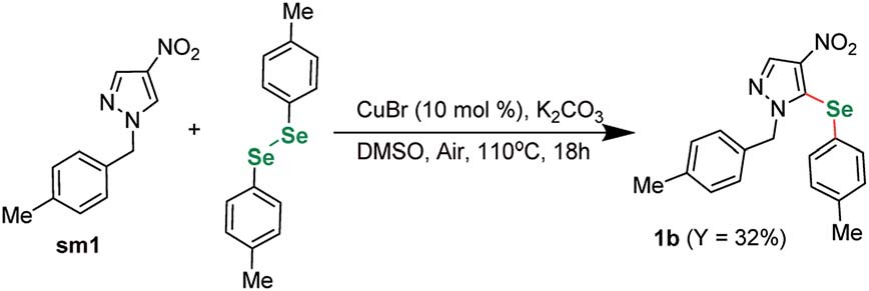
Test reaction for 4-nitro-1*H*-pyrasole derivative.

In order to confirm the validity of the literature protocols we started our investigations by performing a test reaction between a *N*-substituted nitro-heterocycle and diphenyl diselenide (Scheme 1). Basing on our previous research on TM-catalyzed C–H functionalization of nitro-substituted (hetero)arenes we selected pyrazole sm1 as a model compound for this experiment. The conditions were selected following the literature on reactions involving diaryl diselenides and our experience. The reaction between sm1 (1 equiv.) and 1,2-di-*p*-tolyldiselane (1 equiv.) catalyzed by CuBr (0, 1 equiv.) under basic conditions (3 equiv. K_2_CO_3_) in DMSO under air gave the expected product in 32% yield after 18 h in 110 °C. The selenation occurred exclusively at the C(5) position of the pyrazole ring.

With this encouraging result in hand we moved to a three-component reaction. The above conditions applied to the same starting material sm1 in reaction with 4-iodotoluene and selenium powder gave the expected product in 38% yield (Table 1, entry 1). With this result we began the optimization of the conditions.

**Table 1:** Optimization of the reaction conditions^a^

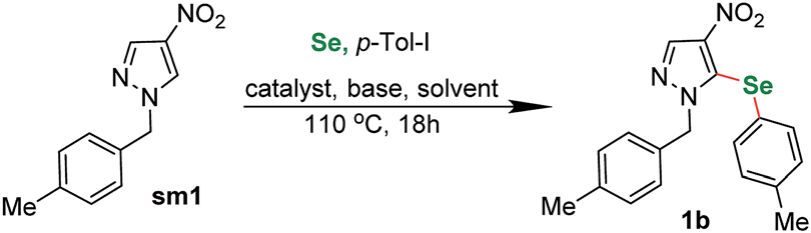					
Entry	Catalyst	Base	Solvent	Yield%	Ref.
1	CuBr	K_2_CO_3_	DMSO	38	
2	CuI	KHCO_3_	DMSO	23	57
3	CuI	None	DMF	NR	58
4	Cu(OAc)_2_	KOH	DMF	30^b^	59
5	CuO	None	DMF	NR	60
6	CuO	Na_3_PO_4_·12H_2_O	DMSO	46^c^	61
7	CuCl_2_	Na_2_CO_3_	DMF	39^c^	62
8	CuI	K_2_CO_3_	DMSO	14^c^	37
9	Pd(OAc)_2_	K_2_CO_3_	DMSO	51^c^	63
10	PdCl_2_(PPh_3_)_2_	K_2_CO_3_	DMSO	55^c^	
11	[Ru(*p*-cymene)Cl_2_]_2_	K_2_CO_3_	DCE	27	64
12	CuI	None	DMSO	NR	65
13	CuO	K_2_CO_3_	DMF	43^c^	66
14	CuBr_2_	K_2_CO_3_	DMF	45^c^	66
15	CuBr_2_	K_2_CO_3_	DMA	40^c^	
16	CuBr_2_	K_2_CO_3_	Toluene	9^c^	
17	CuBr_2_	K_2_CO_3_	DMSO	74^c^	
**18**	**CuBr**_**2**_	**K**_**2**_ **CO**_**3**_	**DMSO**	**83**	
19	CuCl_2_	K_2_CO_3_	DMSO	63	
20	CuBr_2_	KOH	DMSO	70	
21	NiCl_2_(PPh_3_)_2_	K_2_CO_3_	DMSO	Trace^c^	
22	ReOCl_3_(PPh_3_)_2_	K_2_CO_3_	DMSO	15^c^	
23	Co(OAc)_2_	K_2_CO_3_	DMSO	10^c^	
24	FeCl_2_	K_2_CO_3_	DMSO	9^c^	
25	AgOAc	K_2_CO_3_	DMSO	12^c^	
26	PtCl_2_(bpy)	K_2_CO_3_	DMSO	29^c^	

a Reaction conditions unless specified otherwise: 1 equiv. sm1, 2 equiv. aryl iodide, 3 equiv. selenium powder (100 mesh), 4 equiv. base, catalyst (10 mol%), 1 mL dry solvent. Performed in a Teflon screw cap-sealed pressure tube.

b Ligand added – Phen (10 mol%).

c Reaction loaded in glovebox under argon.

At start we applied the conditions matching those known in the literature to work for selenation of heteroaryls with elemental selenium. Braga *et al.*^57^ tested many variants involving CuI and a handful of other copper salts for the selenation of 1,3,4-oxadiazoles, for most of which he obtained quite satisfactory results. In our case however, the combination of CuI and KHCO_3_ gave only 23% yield (Table 1, entry 2). Following the procedure optimized by Wang *et al.*^58^ we skipped the base and switched to DMF, this combination also was not optimal (Table 1, entry 3). This is not however surprising, since the authors of the mentioned work assumed a radical pathway in their reaction involving a derivative of imidazo[1,2-*a*]pyridine.

The same substrates were investigated by Guo *et al.* twice, in combination with aryl iodides and coumarinyl triflates. Application of similar conditions did not raise the yield significantly (Table 1, entries 4 and 5). Another set of conditions, following the work of Wu and Wu *et al.*^61^ pointed at CuO in the combination with strong base in DMSO under argon (46% yield, Table 1, entry 6). This prompted us to apply Cu(ii) salt again (Table 1, entry 7). A few other combinations were tried (entries 8, 12, 13, 19) with moderate success, but only after switching to CuBr_2_ the yields raised to higher levels (entries 14–18 and 20). Finally, the optimal conditions were found (entry 18). Beside the copper salt, the choice of the base seems to be less important than the choice of the solvent and aerobic conditions. This indicates that an oxidant might be consumed during the course of the reaction, since both – DMSO and O_2_ in air can act as oxidizing agents. A handful of other conditions were tested, including catalysts based on more expensive transition metals, known to work in C–H activation protocols. Among those, only the use of Pd(ii) salts yielded more than 50% of the target product.

Having acquired the optimal conditions for our starting material, we moved on to the scope assessment. 4-Nitropyrazole sm1 was submitted to reaction with a number of substituted iodobenzene derivatives (Table 2, entries 1–9). The results were mostly satisfactory, although in some cases the duration and temperature of the reaction had to be slightly extended. In order to test the influence of the alkyl substituent at N(1) position we submitted derivatives with phenethyl-(sm2), phenylpropyl-(sm3) and butyl-(sm4) substituents to the reaction. It is of note, for the 4-nitropyrozoles in question the reactions with *o*-fluoroiodobenzene all gave similar results (yield = 71–77%, Table 2, entries 6, 11, 16, 20). In the case of *m*-fluoroiodobenzene the yields are slightly lower and range from 67 to 76% (Table 2, entries 7, 12, 17, 21), what can be attributed to a different inductive EWG effect.

**Table 2:** Scope of the pyrazole substrates *vs.* aryl iodides^a^

Entry	Structure	R=	Number	Time/h	Temp./°C	Yield/%
1	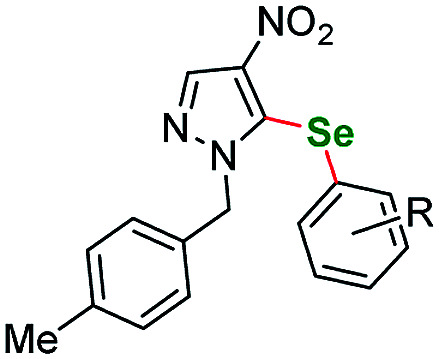	*m*-Me	1a	24	115	77
2		*p*-Me	1b	25	115	83
3		*p*-Et	1c	24	115	85
4		*m*-CF_3_	1d	26	115	80
5		*p*-CF_3_	1e	25	115	70
6		*o*-F	1f	25	115	77
7		*m*-F	1g	25	115	79
8		*p*-F	1h	35	115	81
9		*p*-Br	1i	25	115	64
10	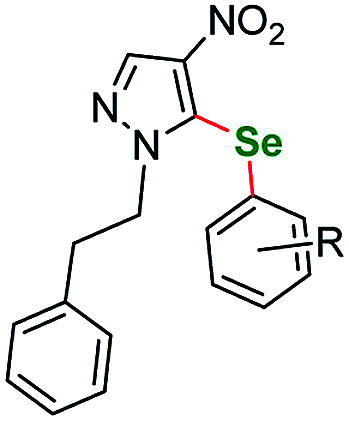	*m*-CF_3_	2a	30	115	83
11		*o*-F	2b	30	115	71
12		*m*-F	2c	25	115	82
13		*p*-Cl	2d	25	115	79
14		*p*-MeO	2e	25	115	72
15		2-(3-Br-py)	2f	25	115	56
16	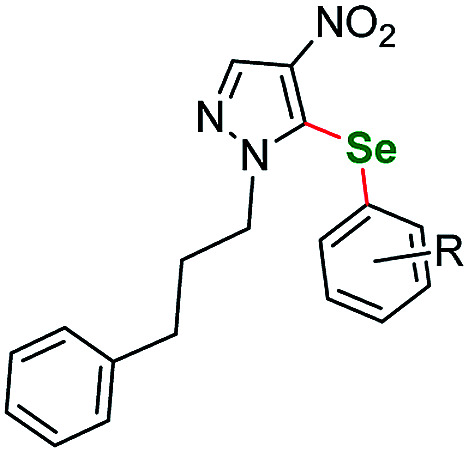	*o*-F	3a	26	115	75
17		*m*-F	3b	26	115	80
18		*p*-F	3c	26	115	86
19	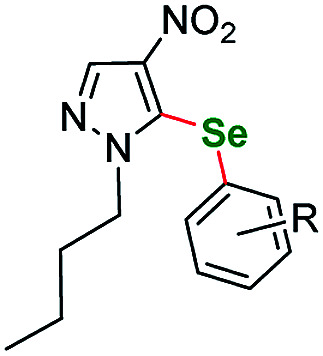	*o*-Me	4a	24	115	69
20		*o*-F	4b	24	115	74
21		*m*-F	4c	24	115	81
22		*p*-F	4d	30	115	79
23	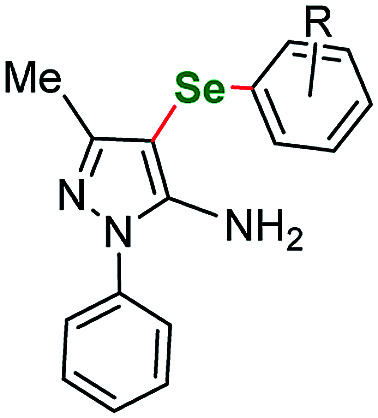	*m*-Me	5a	24	110	79
24		*p*-Me	5b	24	110	89
25		*p*-Et	5c	24	110	83
26		*m*-CF_3_	5d	24	110	76
27		*p*-CF_3_	5e	17	110	85
28		*o*-F	5f	18	110	80
29		*m*-F	5g	19	110	79
30		*p*-F	5h	17	110	74
31		*o*-Br	5i	22	110	66
32		*p*-Cl	5j	24	110	71
33		*m*-NO_2_	5k	24	110	80
34		*p*-MeO	5l	24	110	75
35		*o*-CH_3_OOC	5m	20	110	65

a Reaction conditions unless specified otherwise: 1 equiv. sm1–sm5, 2 equiv. aryl iodide, 3 equiv. selenium powder (100 mesh), 4 equiv. K_2_CO_3_, CuBr_2_ (10 mol%), 1 mL dry DMSO. Performed in a Teflon screw cap-sealed pressure tube, loaded in air.

It is worth to mention that the removal or exchange of the pyrazole C(4)-nitro substituent makes the starting materials unreactive in our protocol. The unsubstituted 1-phenethyl-1*H*-pyrazole as well as the ethyl 1-phenethyl-1*H*-pyrazole-4-carboxylate derivatives gave only faint traces of the products or no reaction at all when submitted to the reaction with a variety of phenyl iodides. This result shows the impact of the directing effect of the nitro group. The same situation is true for 4-nitro *N*-phenyl pyrazoles. Derivatives with *p*-tolyl- and 4-fluorophenyl-substituents both gave negative results what indicates that electron-withdrawing substituents at the N(1) position can also render the starting material unreactive at the C(5) position. Therefore, as a next step we submitted an electron rich 3-methyl-1-phenyl-1*H*-pyrazole-5-amine (sm5) that has only the C(4)–H bond available for transformation. As expected, this starting material underwent the C–H activation under the optimized conditions much easier. This allowed for a broader scope of aryl iodides to be tested, however all the attempts gave very satisfactory results. Lower yields were recorded only for *o*-Br and *o*-CH_3_OOC substituted aryl iodides, what could be caused by the volume of those substituents (Table 2, entries 31 and 35 respectively).

Following our previous work, we were eager to test also 4-nitro imidazole derivatives within our protocol. The first attempts however were disappointing in terms of the yield. We conducted a short optimization again (Table 3), for starting material sm7. The prolongation of the reaction course and raising the temperature had only limited influence on the reaction yield. CuBr_2_ remained the best catalyst. Finally, switching to Cs_2_CO_3_ as base and DMA as solvent raised the yield to about 68% (Table 3, entry 8). Similarly as for pyrazoles, the selenation occurred exclusively at the C(5) position of the imidazole ring. We also conducted several experiments regarding *N*-substitution of 4-nitroimidazoles. Similarly as for pyrazoles, starting material with *p*-tolyl- substituent at the N(1) position gave only trace product. We also submitted to the protocol two *N*-methyl derivatives. 1-Methyl-4-nitro-1*H*-imidazole did not react at all, whereas 1,2-dimethyl-4-nitro-1*H*-imidazole gave only trace product.

**Table 3:** Optimization of reaction conditions for imidazole derivatives^b^

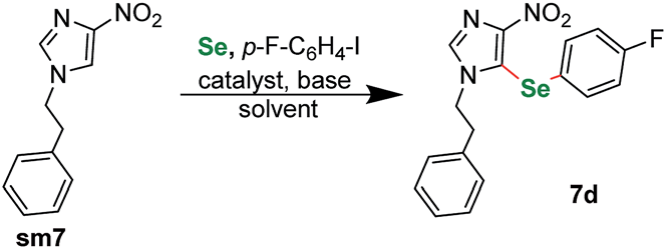						
Entry	Catalyst	Base	Solvent	Temp./°C	Time/h	Yield/%
1	CuBr_2_	K_2_CO_3_	DMSO	110	18	10
2	CuBr_2_	K_2_CO_3_	DMSO	125	96	18
3	CuBr_2_	K_2_CO_3_	DMSO	130	40	14
4	CuBr_2_	K_2_CO_3_	Toluene	130	40	NR
5	CuBr_2_	K_2_CO_3_	DMF	130	40	22
6	CuBr_2_	K_2_CO_3_	DMA	130	40	34
7	CuBr_2_	Cs_2_CO_3_	DMSO	115	24	49
**8**	**CuBr**_**2**_	**Cs**_**2**_ **CO**_**3**_	**DMA**	**115**	**24**	**68**
9	CuBr_2_	Li_2_CO_3_	DMSO	115	40	NR
10	CuI	K_2_CO_3_	DMSO	115	40	NR
11	Cu(OAc)_2_	K_2_CO_3_	DMSO	115	40	Trace
12	NiCl_2_·6H_2_O	K_2_CO_3_	DMSO	115	40	NR
13	AgOAc	K_2_CO_3_	DMSO	115	40	Trace
14	PdCl_2_(PPh_3_)_2_	K_2_CO_3_	DMSO	115	40	Trace^a^

a Reaction conditions unless specified otherwise: 1 equiv. sm7, 2 equiv. aryl iodide, 3 equiv. selenium powder (100 mesh), 4 equiv. base, catalyst (10 mol%), 1 mL dry solvent. Performed in a Teflon screw cap-sealed pressure tube.

b Loaded in glovebox under argon.

To the best of our knowledge there are only a few reports of compounds structurally similar to ours that contain the pyrazole or imidazole structural moiety, all of them were obtained from diselenides as selenium source. Perin and Schumacher *et al.* developed an oxidant promoted mild protocol using potassium peroxymonosulfate (Oxone) to conduct direct selenation of *N*-unsubstituted pyrazoles.^99^ Yan *et al.* successfully selenated a series of *N*-phenyl substituted pyrasoles *via* I_2_ mediated protocol.^100^ Zhang and Zhong *et al.* used a FeBr_3_/I_2_ complex to introduce phenyl- and benzyl-selanyl groups into a series of *N*-phenyl aminopyrazoles.^101^ Schiesser *et al.* performed a lithium/selenium exchange on a N-SEM protected (2-(trimethylsilyl)ethoxymethyl-) imidazole ring as a step in the synthesis of selenofonsartan analogues.^102^ We tested also benzimidazole derivative sm10 as an example of fused imidazole derivative. The outcome was quite satisfying (Table 4, entry 9–72% yield, entry 10–77% yield). A triazole derivative sm11 was also tested as an example of this family of five-membered heterocycles.

**Table 4:** Scope of the imidazoles and other heteroaryl substrates *vs.* aryl iodides^b^

Entry	Structure	R=	Number	Time/h	Temp./°C	Yield/%
1	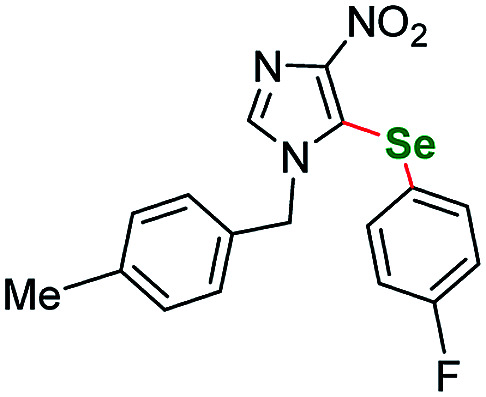		6	24	115	65^a^
2	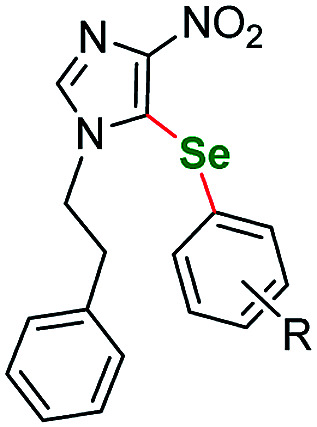	*p*-Me	7a	24	115	86^a^
3		*o*-F	7b	30	115	55^a^
4		*m*-F	7c	24	120	73^a^
5		*p*-F	7d	30	115	68^a^
6	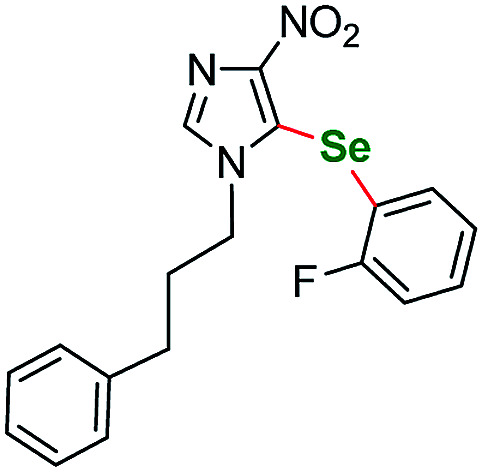		8	24	120	60^a^
7	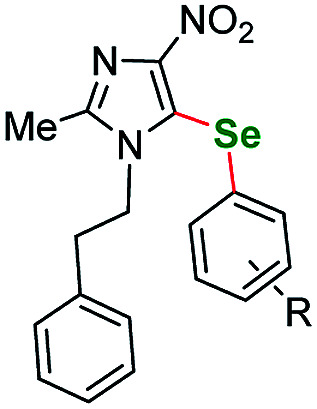	*o*-F	9a	25	120	57^a^
8		*p*-F	9b	30	120	73^a^
9	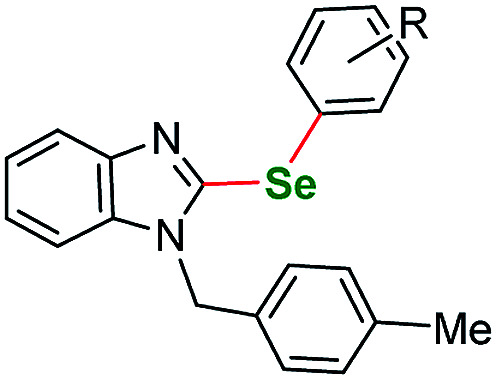	3,5-diCF_3_	10a	24	115	72
10		*m*-OCF_3_	10b	24	115	77
11	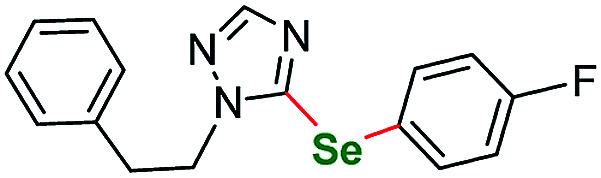		11	24	115	67
12	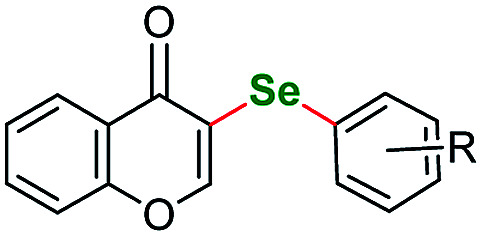	*p*-Et	12a	30	120	46
13		*p*-F	12b	30	120	48

a Reaction conditions unless specified otherwise: 1 equiv. sm6–sm12, 2 equiv. aryl iodide, 3 equiv. selenium powder (100 mesh), 4 equiv. K_2_CO_3_, CuBr_2_ (10 mol%), 1 mL dry DMSO. Performed in a Teflon screw cap-sealed Pressure Tube, loaded in air.

b Base – Cs_2_CO_3_ and solvent – DMA.

Chromones and their derivatives are common motifs abundant in biologically active compounds, numerous natural products, and pharmaceuticals.^103–105^ Following our interest in those scaffolds^106,107^ we submitted 4*H*-chromen-4-one (sm12) to aryloselenation to our optimized reaction conditions. The two attempts we undertook gave satisfactory results (Table 4, entries 12 and 13). To the best of our knowledge there are only a few literature reports of selenated chromones (either by direct C–H activation^108,109^ or *via de novo* cyclization^110,111^) none of them obtained with the use of elemental selenium. With respect to the latter methodology we also submitted an enaminone to our protocol however, the reaction of (*E*)-3-(dimethylamino)-1-(2-hydroxyphenyl)prop-2-en-1-one was unsuccessful. A few other heteroaryl starting materials were tested within the current protocol giving negative results. Among them were: 3-methyl-5-nitro-1-phenyl-1*H*-pyrazolo[3,4-*b*]pyridine, 1,3-dimethyl-6-aminouracil, 3,4,5-trimethoxyaniline and 3-nitropyridine.

Following other seminal works in the field we were curious to see if our protocol can be used to perform an intramolecular cyclization introducing selenium. We procured a series of heterocycles bearing 2-iodobenzyl- substituent at the N(1) ring atom: 1-(2-iodobenzyl)-1*H*-pyrazole, 1-(2-iodobenzyl)-4-nitro-1*H*-pyrazole, ethyl 1-(2-iodobenzyl)-1*H*-pyrazole-4-carboxylate, 1-(2-iodobenzyl)-1*H*-imidazole, 1-(2-iodobenzyl)-4-nitro-1*H*-imidazole, ethyl 1-(2-iodobenzyl)-1*H*-imidazole-4-carboxylate and 1-(2-iodobenzyl)-1*H*-benzo[*d*]imidazole. Similarly, to the parent starting materials tested in the intermolecular attempts, only the nitro-derivatives and benzimidazole successfully reacted giving benzoselenazines 13a, 13b and 13c in 79%, 62% and 70% yield respectively (Fig. 2).

**Fig. 1:**
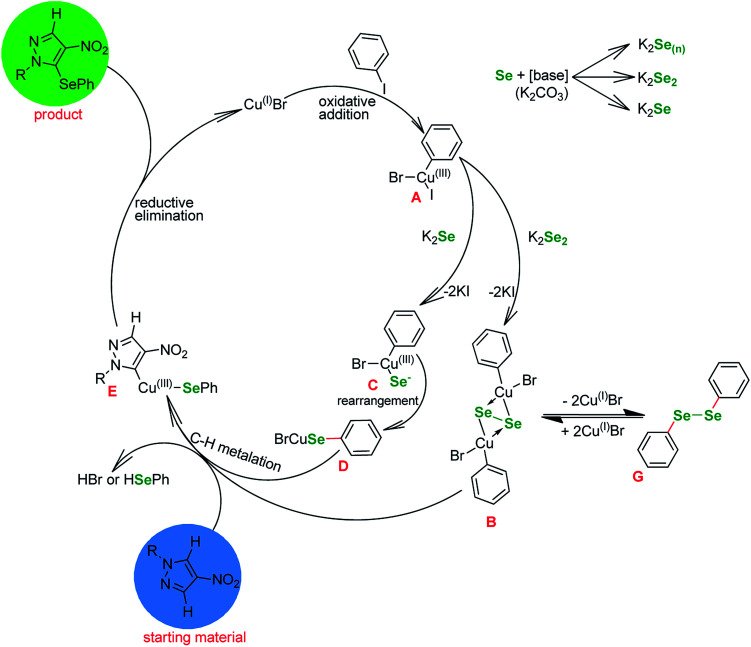
Proposed mechanism of the reaction.

Basing on the literature reports, our observations and the outcome of the optimizations (Tables 1 and 3) we propose the following mechanism (Fig. 1). Since the reaction is not proceeding without base (Table 1, entries 3, 5 and 12) it is safe to assume that the selenium enters the catalytic cycle in a form of a selenide anion (Se^2−^)^61,62^ or diselenide anion (Se_2_^2−^).^57^ The reduction of elemental selenium with base is a well known process. The first step in the catalytic cycle involving copper is oxidative addition of Cu^(I)^ halide into the I–C_Ar_ bond of the phenyl iodide, forming Cu^(III)^ intermediate A.

**Fig. 2:**
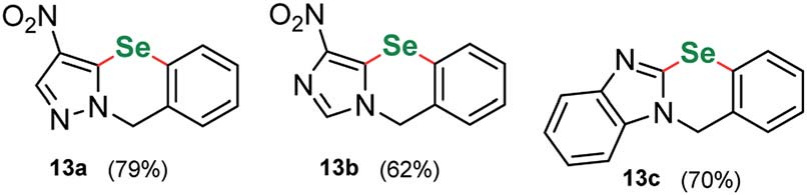
Cyclic benzoselenazine derivatives.

These species react with the reduced selenium to produce a copper–selenium complex. At this point it is unpractical to distinguish between mono, di and polyselenide anions and whether the Se–Se bond is retained or not. Many authors formally agree on the existence of a square-planar species B, which is a common point with similar to our copper-catalyzed selenations utilizing diselenides, since it can undergo a reversible transformation to diphenyl diselenide G.^59^ However, at this point it is necessary to depict the transfer of the Se atom (C to D) and the formation of Se–C bond. Next, the selenium–copper complex enters reaction with the heterocyclic starting material in a metalation step, with the formal extraction of HBr or HSePh species. The intermediate E undergoes reductive elimination to give the desired product and regenerate the catalyst.

**Fig. 3:**
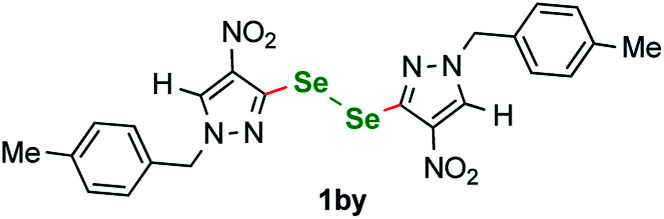
Byproduct 1by: (1,2-bis(1-(4-methylbenzyl)-4-nitro-1*H*-pyrazole-5-yl)diselane).

**Fig. 4:**
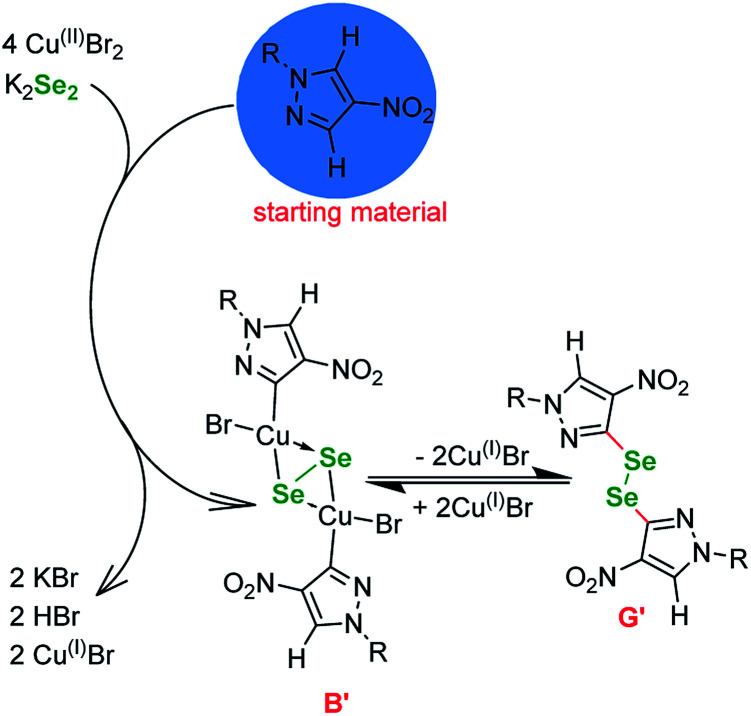
Mechanistic explanation for the presence of byproduct 1by.

We have also detected an unusual byproduct in the crude mixtures from reactions 1a–1i. Diselenide 1by (Fig. 3) was isolated during the chromatographic purification of products 1e and 1g from the crude mixtures in 10% and 8% yield. The presence of this diselenide can be explained as follows (Fig. 4). The starting material is oxidized by Cu^(II)^ species forming a copper complex that reacts with K_2_Se_2_ to give an analogous to B square-planar species B′, that can also undergo a reversible transformation yielding byproduct G′.

**Fig. 5:**
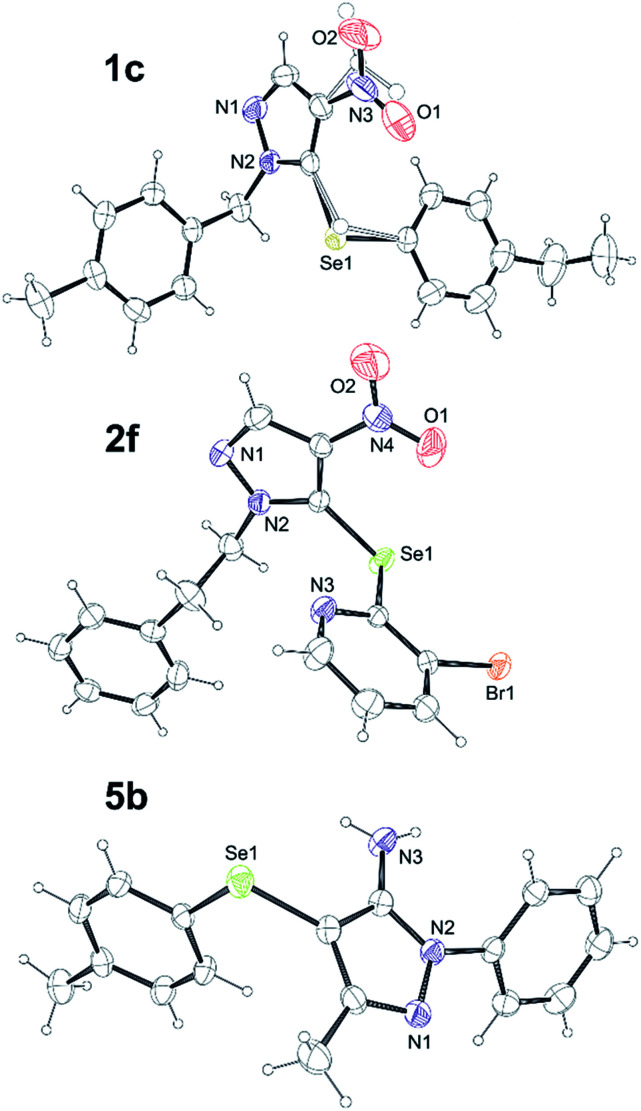
Ortep drawings of molecules 1c, 2f and 5b found in crystals. The thermal ellipsoids are drawn with 30% probability. Only the labels for atoms heavier than carbon atom are given. In 1c minor components of disordered groups are depicted with spheres of arbitrary radius.

Finally, compounds 1c, 2f, 5b, 5d, 5f, 5i, 5k and 5m were obtained as single crystals suitable for X-ray diffraction analysis. X-ray crystallographic analyses of the title structures helped us to corroborate the structural constitution of the samples and determined the position of Se–Ar substituent (See Fig. 1 in ESI†). The representative structures of compounds 1c, 2f and 5b are depicted in the Fig. 5.

## Conclusions

We have proposed a general method to obtain unsymmetrical heteroaryl-aryl selenides containing 4-nitropyrazole, 4-nitroimidazole and a few other scaffolds by copper-catalyzed direct C–H selenation of *N*-substituted heteroaryls with iodobenzenes and elemental selenium. The scope and limitations of our methodology has been assessed in respect to both organic starting materials. A plausible mechanism has been proposed in relation to the previous research. The established reaction conditions work very well for 4-nitropyrasole derivatives and acceptably good for 4-nitroimidazoles. A total of 48 compounds have been prepared, majority of them bearing easily transformable nitro group and a few of them substituted with a similarly useful groups in the benzene ring. The protocol is also useful for closing a benzoselenazine ring from appropriate starting materials.

## Conflicts of interest

Authors declare no conflict of interest.

## Supplementary Material

RA-009-C9RA05004C-s001

RA-009-C9RA05004C-s002
