# Supplementary material for: Copper-catalyzed direct C–H arylselenation of 4-nitro-pyrazoles and other heterocycles with selenium powder and aryl iodides. Access to unsymmetrical heteroaryl selenides
Source: RSC Adv. 2019 Aug 13;9(44):25368–76. doi: 10.1039/c9ra05004c (PMC9070035; doi:10.1039/c9ra05004c)
Supplement: RA-009-C9RA05004C-s001 [file RA-009-C9RA05004C-s001.pdf]

Electronic Supplementary Material (ESI) for RSC Advances.
This journal is © The Royal Society of Chemistry 2019

Copper-catalyzed direct C-H arylselenation of 4-nitro-pyrazoles and other heterocycles with selenium powder and aryl iodides. Access to unsymmetrical heteroaryl selenides.

Michał Jakubczyk,[a] Satenik Mkrtchyan,*[a] Izabela D. Madura,[b] Paulina H. Marek,[b,c] and Viktor O. Iaroshenko.*[a]

[a] *Laboratory of Homogeneous Catalysis and Molecular Design at the Centre of Molecular and Macromolecular Studies, Polish Academy of Sciences, Sienkiewicza 112, PL-90-363 Łodź, Poland. E-mail: iva108@gmail.com , viktori@cbmm.lodz.pl*

[b] *Department of Inorganic Chemistry, Faculty of Chemistry, Warsaw University of Technology. Noakowskiego 3, 00-664, Warsaw, Poland.*

[c] *University of Warsaw, Faculty of Chemistry, Pasteura 1, 02-093 Warsaw, Poland.*

Table of Contents

(A) Experimental section -- 3
(B) Characterization of products -- 5
(C) Copies ^{1}H and ^{13}C NMR spectra -- 30
(D) X-ray single crystal analysis of compounds **1c, 2f, 5b, 5d, 5f, 5i, 5k and 5m** -- 145

(A) Experimental section.

Known starting materials: 1-[(4-methylphenyl)methyl]-4-nitro-1*H*-pyrazole (**sm1**), 4-nitro-1-phenethyl-1*H*-pyrazole (**sm2**), 4-nitro-1-butyl-1*H*-pyrazole (**sm4**), 3-methyl-1-phenyl-1*H*-pyrazol-5-amine (**sm5**), 4-nitro-1-phenethyl-1*H*-imidazole (**sm7**), 2-methyl-4-nitro-1-phenethyl-1*H*-imidazole (**sm9**), 1-[(4-methylphenyl)methyl]-1*H*-benzimidazole (**sm10**), 1-phenethyl-1H-1,2,4-triazole (**sm11**), 4*H*-chromen-4-one (**sm12**), 1-(2-iodobenzyl)-1*H*-benzo[*d*]imidazole (**sm15**) - were prepared according to literature procedures.[1–7] Triazole **sm9** was obtained as a 1 : 8.5 molar ratio mixture of 4*H*- and 1*H*- 1,2,4-triazole *N*-alkylation products, respectively. However, only the 1*H*-isomer underwent the selenylation giving the expected C(5)-substituted product after chromatographic separation from impurities and both starting materials (**Table 4**, Entry 46). Starting materials **sm3**, **sm6**, **sm8**, **sm13** and **sm14** (**Scheme 1**) were prepared according to the general procedure described below. Commercially available reagents, catalysts and anhydrous and degassed solvents were used without further purification. Flash column chromatography was performed with Merck silica gel 60 (230-400 mesh). The solvents for column chromatography were distilled before use. Thin layer chromatography was carried out using Merck TLC Silica gel 60 F254 and visualized by short-wavelength ultraviolet light or by treatment with iodine or potassium permanganate ($KMnO_4$) stain. Melting points are uncorrected. ^{1}H, ^{13}C and ^{19}F NMR spectra were recorded on a Bruker 200 and 500 MHz at 20ºC. All ^{1}H NMR spectra are reported in parts per million (ppm) downfield of TMS and were measured relative to the signals for $CHCl_3$ (7.26 ppm) and DMSO (2.50 ppm). All ^{13}C NMR spectra were reported in ppm relative to residual $CHCl_3$ (77.00 ppm) or DMSO (39.70 ppm) and were obtained with ^{1}H decoupling. Coupling constants, *J*, are reported in Hertz (Hz). Gas chromatographic analyses was performed on Gas Chromatograph Mass Spectrometer GCMS-QP2010 Ultra instrument. Isolation of obtained compounds was achieved by column chromatography on Silica gel.

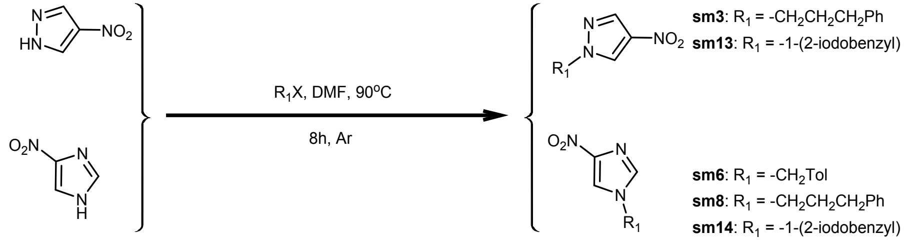

Scheme 1: Preparation of previously unknown starting materials.

General procedure for the *N*-alkylation of pyrazol and imidazole derivatives (starting materials **sm3**, **sm6**, **sm8**, **sm13**, **sm14**): Corresponding 4-nitro-1*H*-pyrazole (1eq.) and K_2CO_3 (2.3eq.) successively were weighed to air and placed in a Schlenk flask, equipped with a magnetic stir bar, which then was capped with a rubber septum. The reaction

vessel was evacuated and backfilled with argon. The DMF (8mL for 1g of 4-nitro-1*H*-pyrazole) and corresponding alkyl bromide (1.3eq.) were added via a syringe, and the reaction was heated to 90 °C for 8 h. Upon completion, the reaction was cooled to room temperature and concentrated under vacuum. The crude mass was washed with water, which was extracted with chloroform afterward. Finally, the organic phase was dried (Na_2SO_4), filtered, and evaporated to dryness, or (if necessary) the residue was purified by column chromatography typically using hexane/ethyl acetate mixtures to provide the desired alkylated product.

General procedure for the synthesis of diorganyl selenides (compounds 1a-1i, **2a-2f**, **3a-3c**, **4a-4d**, **5a-5m**, **6**, **7a-7d**, **8**, **9a**, **9b**, **10a**, **10b**, **11**, **12a**, **12b**): An Ace Glass Pressure Tube was equipped with a suitable stirring pellet and loaded with 250mg (1.15mmol, 1eq.) of 1-[(4-methylphenyl)methyl]-4-nitro-1*H*-pyrazole, 502mg (2.30mmol, 2eq.) of 3-iodotoluene, 273mg (3.45mmol, 3.5eq.) of selenium (100 mesh), 26mg (0.11mmol, 0.1eq.) of $CuBr_2$ and 636mg (4.60mmol, 4eq.) of K_2CO_3. 1mL of dry DMSO was added and the tube was carefully closed with an original Ace Glass Tube (Teflon) plug. The vessel was placed in an aluminium heating-block on a hotplate. The reaction was kept in 115°C for 24h. After cooling down - the pressure tube was carefully opened under venting hood and the mixture was washed to a flask with MeOH. The volatiles were removed using a rotary evaporator and DMSO was removed by heating to about 60 °C under vacuum (oil vacuum pump). The solid crude mixture was purified by column chromatography typically using hexane/ethyl acetate gradient to provide the desired selenide.

General procedure for the synthesis of benzoselenazines (compounds 13a, 13b and 13c): An Ace Glass Pressure Tube was equipped with a suitable stirring pellet and loaded with 360mg (1.07mmol, 1eq.) of 1-(2-iodobenzyl)-1*H*-benzo[*d*]imidazole (**sm15**), 196mg (2.47mmol, 2.3eq.) of selenium (100 mesh), 24mg (0.10mmol, 0.1eq.) of $CuBr_2$ and 596mg (4.30mmol, 4eq.) of K_2CO_3. 1mL of dry DMSO was added and the tube was carefully closed with an original Ace Glass Tube (Teflon) plug. The vessel was placed in an aluminium heating-block on a hotplate. The reaction was kept in 115 °C for 24h. After cooling down - the pressure tube was carefully opened under venting hood and the mixture was washed to a flask with MeOH. The volatiles were removed using a rotary evaporator and DMSO was removed by heating to about 60 °C under vacuum (oil vacuum pump). The solid crude mixture was purified by column chromatography typically using hexane/ethyl acetate gradient to provide the desired benzoselenazine.

(B) Characterization of products.

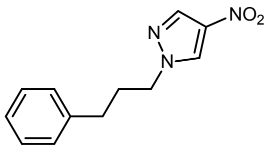

4-nitro-1-(3-phenylpropyl)-1H-pyrazole (sm3): white powder, mp. 55-56 °C, ^{1}H NMR (500 MHz, $CDCl_3$): δ (ppm): 8.08 (s, 2H, C(3) and C(5)-pyrazole), 7.31 (t, 2H, ^{3}J = 7.4 Hz, CH_{Ar}), 7.22 (t, 1H, ^{3}J = 7.4 Hz, CH_{Ar}), 7.17 (d, 2H, ^{3}J = 7.0 Hz, CH_{Ar}), 4.14 (t, 2H, ^{3}J = 7.1 Hz, CH_2), 2.65 (t, 2H, ^{3}J = 7.5 Hz, CH_2), 2.33 - 2.18 (m, 2H, CH_2), ^{13}C NMR (126 MHz, $CDCl_3$): δ (ppm): 139.98, 135.94, 128.80, 128.56, 128.47, 126.58, 52.72, 32.47, 31.08, MS (GC, 70 eV): *m/z* = 231 (M^+, 19%), 126 (29), 118 (100), 91 (31), 65 (12), HRMS (TOF MS ES+): calcd for $C_{12}H_{14}N_3O_2$ $(M + H)^+$ 232.1086, found 232.1088.

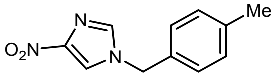

1-[(4-methylphenyl)methyl]-4-nitro-1H-imidazole (sm6): white powder, 103-104 °C, ^{1}H NMR (500 MHz, $CDCl_3$): δ (ppm): 7.70 (s, 1H, C(2)-imidazole), 7.47 (s, 1H, C(5)-imidazole), 7.22 (d, 2H, ^{3}J = 7.8 Hz, CH_{Ar}), 7.13 (d, 2H, ^{3}J = 7.9 Hz, CH_{Ar}), 5.12 (s, 2H, CH_2), 2.37 (s, 3H, CH_3), ^{13}C NMR (126 MHz, $CDCl_3$): δ (ppm): 139.55, 136.03, 130.65, 130.29, 128.12, 119.38, 52.17, 21.31, MS (GC, 70 eV): *m/z* = 217 (M^+, 11%), 105 (100), 77 (13), HRMS (TOF MS ES+): calcd for $C_{11}H_{11}N_3O_2Na$ $(M + Na)^+$ 240.0749, found 240.0754.

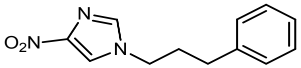

4-nitro-1-(3-phenylpropyl)-1H-imidazole (sm8): white powder, mp. 79-80 °C, ^{1}H NMR (500 MHz, $CDCl_3$): δ (ppm): 7.75 (s, 1H, C(2)-imidazole), 7.40 (s, 1H, C(5)-imidazole), 7.32 (t, 2H, ^{3}J = 7.5 Hz, CH_{Ar}), 7.24 (t, 1H, ^{3}J = 7.4 Hz, CH_{Ar}), 7.15 (d, 2H, ^{3}J = 7.2 Hz, CH_{Ar}), 4.01 (t, 2H, ^{3}J = 7.2 Hz, CH_2), 2.67 (t, 2H, ^{3}J = 7.4 Hz, CH_2), 2.21 (quint, 2H, ^{3}J = 7.3 Hz, CH_2), ^{13}C NMR (126 MHz, $CDCl_3$): δ (ppm): 139.36, 136.08, 128.96, 128.40, 126.84, 119.13, 47.57, 32.35, 31.88, MS (GC, 70 eV): *m/z* = 231 (M^+, 29%), 127 (100), 118 (57), 91 (82), 77 (13), 65 (24), HRMS (TOF MS ES+): calcd for $C_{12}H_{13}N_3O_2Na$ $(M + Na)^+$ 254.0905, found 254.0906.

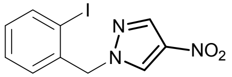

1-(2-iodobenzyl)-4-nitro-1H-pyrazole (sm13): light yellow crystals, mp. 100 °C, ^{1}H NMR (500 MHz, $CDCl_3$): δ (ppm): 8.12 (s, 1H, C(3)-pyrazole), 8.11 (s, 1H, C(5)-pyrazole), 7.91 (dd, 1H, ^{3}J = 7.9 Hz, ^{4}J = 1.0 Hz, CH_{Ar}), 7.39 (td, 1H, ^{3}J = 7.6 Hz, ^{4}J = 1.1 Hz, CH_{Ar}), 7.23 (dd, 1H, ^{3}J = 7.7 Hz, ^{4}J = 1.4 Hz, CH_{Ar}), 7.10 (td, 1H, ^{3}J =7.8 Hz, ^{4}J =1.6 Hz, CH_{Ar}), 5.40 (s, 2H, CH_2), ^{13}C NMR (126 MHz, $CDCl_3$): δ (ppm): 140.29, 136.58, 136.23, 136.14, 130.98, 130.49, 129.26, 129.04, 99.40, 61.55, MS (GC, 70 eV): *m/z* = 217 (2-iodobenzene-CH_2^+, 14%), minus iodide 202 (100), 156 (35), 129 (10), 90 (43), HRMS (TOF MS ES+): calcd for $C_{10}H_9IN_3O_2$ (M + H)$^+$ 329.9739, found 329.9742.

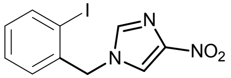

1-(2-iodobenzyl)-4-nitro-1H-imidazole (sm14): white powder, mp. 76-77 °C, ^{1}H NMR (500 MHz, $CDCl_3$): δ (ppm): 7.84 - 7.80 (m, 1H, CH_{Ar}), 7.73 (s, 1H, C(2)-imidazole), 7.51 (s, 1H, C(5)-imidazole), 7.36 - 7.30 (m, 1H, CH_{Ar}), 7.19 (d, 1H, ^{3}J = 6.5 Hz, CH_{Ar}), 7.06 - 7.01 (m, 1H, CH_{Ar}), 5.23 (s, 2H, CH_2), ^{13}C NMR (126 MHz, $CDCl_3$): δ (ppm): 147.87, 140.22, 136.53, 136.21, 130.94, 130.02, 129.26, 119.69, 99.16, 56.34, MS (GC, 70 eV): *m/z* = 329 (M$^+$, 22%), 217 (100), 202 (28), 156 (84), 90 (65), HRMS (TOF MS ES+): calcd for $C_{10}H_8IN_3O_2$ (M + H)$^+$ 329.9739, found 329.9744.

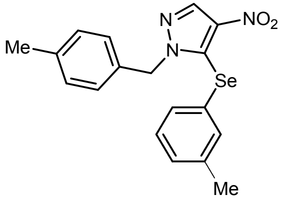

1-[(4-methylphenyl)methyl]-5-[(3-methylphenyl)selanyl]-4-nitro-1H-pyrazole (1a): light yellow viscous oil, ^{1}H NMR (500 MHz, $CDCl_3$): δ (ppm): 8.25 (s, 1H, C(3)-pyrazole), 7.19 - 7.03 (m, 8H, CH_{Ar}), 5.41 (s, 2H, CH_2), 2.33 (s, 3H, CH_3), 2.24 (s, 3H, CH_3), ^{13}C NMR (126 MHz, $CDCl_3$): δ (ppm): 139.88, 138.21, 137.88, 137.47, 133.11, 132.15, 129.63, 129.61, 129.54, 129.52, 128.30, 127.80, 127.76, 55.40, 21.29, 21.20, MS (GC, 70 eV): *m/z* = 387 (M$^+$, 5%), 280 (6), 222 (12), 195 (4), 105 (100), 91 (6), 77 (11), HRMS (TOF MS ES+): calcd for $C_{18}H_{18}N_3O_2Se$ (M + H)$^+$ 388.0564, found 388.0566.

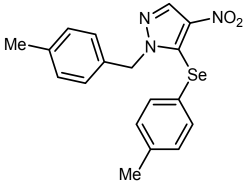

1-[(4-methylphenyl)methyl]-5-[(4-methylphenyl)selanyl]-4-nitro-1H-pyrazole (1b): white powder, mp. 55-56 °C, ^{1}H NMR (500 MHz, $CDCl_3$): δ (ppm): 8.23 (s, 1H, C(3)-pyrazole), 7.26 (d, 2H, ^3J = 8.1 Hz, CH_{Ar}), 7.11 (d, 2H, ^3J = 8.0 Hz, CH_{Ar}), 7.04 (dd, 4H, ^3J = 8.1 Hz, ^4J = 4.7 Hz, CH_{Ar}), 5.39 (s, 2H, CH_2), 2.33 (s, 3H, CH_3), 2.31 (s, 3H, CH_3), ^{13}C NMR (126 MHz, $CDCl_3$): δ (ppm): 139.12, 138.21, 137.73, 137.45, 133.08, 132.17, 130.65, 129.52, 128.78, 127.72, 124.25, 55.33, 21.21, MS (GC, 70 eV): *m/z* = 387 (M^+, 5%), 280 (7), 195 (3), 119 (2), 105 (100), 91 (6), 77 (12), HRMS (TOF MS ES+): calcd for $C_{18}H_{18}N_3O_2Se$ $(M + H)^+$ 388.0564, found 388.0562.

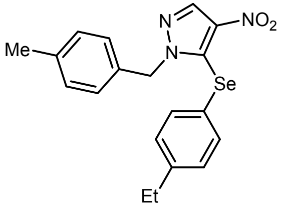

1-[(4-methylphenyl)methyl]-5-[(4-ethylphenyl)selanyl]-4-nitro-1H-pyrazole (1c): yellow powder, mp. 58 °C, ^{1}H NMR (500 MHz, $CDCl_3$): δ (ppm): 8.24 (s, 1H, C(3)-pyrazole), 7.29 (d, 2H, ^3J = 8.2 Hz, CH_{Ar}), 7.10 (d, 2H, ^3J = 8.0 Hz, CH_{Ar}), 7.07 (d, 2H, ^3J = 8.1 Hz, CH_{Ar}), 7.04 (d, 2H, ^3J = 8.0 Hz, CH_{Ar}), 5.41 (s, 2H, CH_2), 2.61 (q, 2H, ^3J = 7.6 Hz, CH_2), 2.33 (s, 3H, CH_3), 1.21 (t, 3H, ^3J = 7.6 Hz, CH_3), ^{13}C NMR (126 MHz, $CDCl_3$): δ (ppm): 145.36, 138.18, 137.75, 137.45, 133.15, 132.16, 129.51, 129.47, 128.73, 127.72, 124.47, 55.30, 28.54, 21.21, 15.39, MS (GC, 70 eV): *m/z* = 401 (M^+, 5%), 280 (8), 209 (3), 105 (100), 77 (11), HRMS (TOF MS ES+): calcd for $C_{19}H_{20}N_3O_2Se$ $(M + H)^+$ 402.0721, found 402.0721.

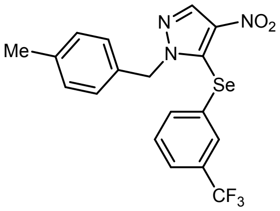

1-[(4-methylphenyl)methyl]-5-[(3-(trifluoromethyl)phenyl)selanyl]-4-nitro-1H-pyrazole (1d): light yellow powder, mp. 68-70 °C, ^{1}H NMR (500 MHz, $CDCl_3$): δ (ppm): 8.28 (s, 1H, C(3)-pyrazole), 7.50 (d, 1H, ^{3}J = 7.8 Hz, CH_{Ar}), 7.45 - 7.40 (m, 2H, CH_{Ar}), 7.31 (t, 1H, ^{3}J = 8.0 Hz, CH_{Ar}), 7.07 (q, 4H, ^{3}J = 8.3 Hz, CH_{Ar}), 5.51 (s, 2H, CH_2), 2.30 (s, 3H, CH_3), ^{13}C NMR (126 MHz, $CDCl_3$): δ (ppm): 138.56, 138.28, 137.63, 135.44, 131.99 (q, $^{2}J_{CF}$ = 32.7 Hz, C_{ipso}-CF_3), 131.85, 130.11, 129.68, 129.35, 128.64 (dd, J_{CF} = 7.5 Hz, J_{CF} = 3.7 Hz), 127.63, 126.66, 125.32 (dd, J_{CF} = 6.9 Hz, J_{CF} = 3.3 Hz), 123.34 (q, $^{1}J_{CF}$ = 273.0 Hz, CF_3), 55.87, 21.16, ^{19}F{^{1}H} NMR (188.34 MHz, $CDCl_3$): δ (ppm): -62.15 (CF_3), MS (GC, 70 eV): *m/z* = 441 (M^+, 5%), 280 (3), 105 (100), 77 (10), HRMS (TOF MS ES+): calcd for $C_{18}H_{15}F_3N_3O_2Se$ $(M + H)^+$ 442.0282, found 442.0277.

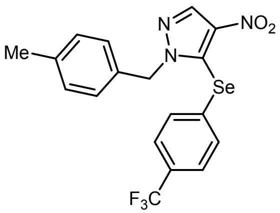

1-[(4-methylphenyl)methyl]-5-[(4-(trifluoromethyl)phenyl)selanyl]-4-nitro-1H-pyrazole (1e): white powder, mp. 75-76 °C, ^{1}H NMR (500 MHz, $CDCl_3$): δ (ppm): 8.30 (s, 1H, C(3)-pyrazole), 7.40 (d, 2H, ^{3}J = 8.2 Hz, CH_{Ar}), 7.27 - 7.24 (m, 2H, CH_{Ar}), 7.03 (q, 4H, ^{3}J = 8.2 Hz, CH_{Ar}), 5.49 (s, 2H, CH_2), 2.29 (s, 3H, CH_3), ^{13}C NMR (126 MHz, $CDCl_3$): δ (ppm): δ 138.59, 138.53, 137.69, 133.4 (d, $^{2}J_{CF}$ = 11.8 Hz, C_{ipso}-CF_3), 131.97, 131.82, 131.34, 129.66, 127.73, 126.52 (dd, J_{CF} = 7.1 Hz, J_{CF} = 3.3 Hz), 126.23, 125.08 (q, $^{1}J_{CF}$ = 288.3 Hz, CF_3), 55.89, 21.19, ^{19}F{^{1}H} NMR (188.34 MHz, $CDCl_3$): δ (ppm): -62.20 (CF_3), MS (GC, 70 eV): *m/z* = 441 (M^+, 7%), 280 (6), 105 (100), 77 (13), HRMS (TOF MS ES+): calcd for $C_{18}H_{14}F_3N_3O_2SeNa$ $(M + Na)^+$ 464.0101, found 464.0104.

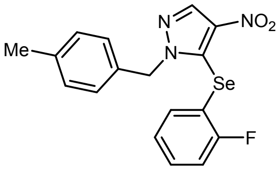

1-[(4-methylphenyl)methyl]-5-[(2-fluorophenyl)selanyl]-4-nitro-1H-pyrazole (1f): light yellow powder, mp. 63-65 °C, ^{1}H NMR (500 MHz, $CDCl_3$): δ (ppm): 8.25 (s, 1H, C(3)-pyrazole), 7.33 - 7.23 (m, 2H, CH_{Ar}), 7.13 - 6.99 (m, 6H, CH_{Ar}), 5.51 (s, 2H, CH_2), 2.32 (s, 3H, CH_3), ^{13}C NMR (126 MHz, $CDCl_3$): δ (ppm): 161.10 (d, $^{1}J_{CF}$ = 245.3 Hz, C_{ipso}-F), 138.34, 138.25, 137.52, 134.11 (d, J = 1.6 Hz), 132.06, 130.91 (d, J_{CF} = 7.8 Hz), 129.59, 127.82, 126.33 (d, J = 1.7 Hz), 125.43 (d, J_{CF} = 3.3 Hz), 116.12 (d, $^{2}J_{CF}$ = 22.6 Hz), 115.07 (d, $^{2}J_{CF}$ =

21.4 Hz), 55.57, 21.20, $^{19}F\{^1H\}$ NMR (188.34 MHz, $CDCl_3$): δ (ppm): -103.00 (CF), MS (GC, 70 eV): *m/z* = 391 (M^+, 5%), 280 (5), 105 (100), 79 (12), 77 (11), HRMS (TOF MS ES+): calcd for $C_{17}H_{15}FN_3O_2Se$ $(M + H)^+$ 392.0314, found 392.0317.

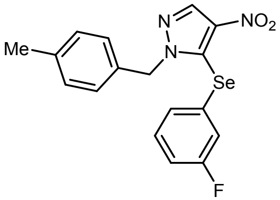

1-[(4-methylphenyl)methyl]-5-[(3-fluorophenyl)selanyl]-4-nitro-1H-pyrazole (1g): yellow oil, ^1H NMR (500 MHz, $CDCl_3$): δ (ppm): 8.27 (s, 1H, C(3)-pyrazole), 7.23 - 7.16 (m, 1H, CH_{Ar}), 7.12 - 7.05 (m, 5H, CH_{Ar}), 6.96 (td, 1H, ^3J = 8.4 Hz, ^4J = 2.0 Hz, CH_{Ar}), 6.88 (dd, 1H, ^3J = 8.4 Hz, ^5J = 1.3 Hz, CH_{Ar}), 5.47 (s, 2H, CH_2), 2.32 (s, 3H, CH_3), ^{13}C NMR (126 MHz, $CDCl_3$): δ (ppm): 162.79 (d, $^1J_{CF}$ = 251.4 Hz, C_{ipso}-F), 138.47, 138.19, 137.56, 131.96, 131.02 (d, J_{CF} = 8.1 Hz), 129.74 (d, J_{CF} = 7.1 Hz), 129.61, 127.74, 127.55 (d, J_{CF} = 3.1 Hz), 126.91, 118.93 (d, $^2J_{CF}$ = 23.2 Hz), 115.61 (d, $^2J_{CF}$ = 21.1 Hz), 55.70, 21.18, $^{19}F\{^1H\}$ NMR (188.34 MHz, $CDCl_3$): δ (ppm): -109.57 (CF), MS (GC, 70 eV): *m/z* = 391 (M^+, 6%), 280 (6), 105 (100), 79 (12), 77 (11), HRMS (TOF MS ES+): calcd for $C_{17}H_{15}FN_3O_2Se$ $(M + H)^+$ 392.0314, found 392.0316.

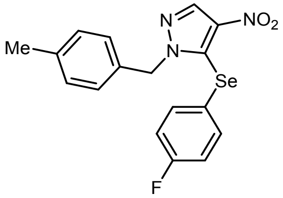

1-[(4-methylphenyl)methyl]-5-[(4-fluorophenyl)selanyl]-4-nitro-1H-pyrazole (1h): light yellow oil, ^1H NMR (500 MHz, $CDCl_3$): δ (ppm): 8.24 (s, 1H, C(3)-pyrazole), 7.35 - 7.29 (m, 2H, CH_{Ar}), 7.1 (d, 2H, ^3J = 8.0 Hz, CH_{Ar}), 7.0 (d, 2H, ^3J = 8.0 Hz, CH_{Ar}), 6.95 - 6.88 (m, 2H, CH_{Ar}), 5.46 (s, 2H, CH_2), 2.33 (s, 3H, CH_3), ^{13}C NMR (126 MHz, $CDCl_3$): δ (ppm): 163.21 (d, $^1J_{CF}$ = 250.0 Hz, C_{ipso}-F), 138.45, 137.86, 137.61, 135.41 (d, J_{CF} = 8.2 Hz), 132.15, 129.67, 128.26, 127.65, 122,48 (d, J_{CF} = 3.3 Hz), 117.10 (d, $^2J_{CF}$ = 22.0 Hz,), 55.58, 21.26, $^{19}F\{^1H\}$ NMR (188.34 MHz, $CDCl_3$): δ (ppm): -110.86 (CF), MS (GC, 70 eV): *m/z* = 391 (M^+, 5%), 280 (4), 105 (100), 77 (12), HRMS (TOF MS ES+): calcd for $C_{17}H_{15}FN_3O_2Se$ $(M + H)^+$ 392.0314, found 392.0314.

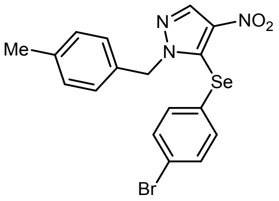

1-[(4-methylphenyl)methyl]-5-[(4-bromophenyl)selanyl]-4-nitro-1H-pyrazole (1i): white powder, mp. 100-101 °C, ^{1}H NMR (500 MHz, $CDCl_3$): δ (ppm): 8.32 (s, 1H, C(3)-pyrazole), 7.52 (d, 1H, ^{3}J = 7.8 Hz, CH_{Ar}), 7.13 - 7.00 (m, 6H, CH_{Ar}), 6.65 (d, 1H, ^{3}J = 7.8 Hz, CH_{Ar}), 5.45 (s, 2H, CH_2), 2.28 (s, 3H, CH_3), ^{13}C NMR (126 MHz, $CDCl_3$): δ (ppm): 138.90, 138.47, 137.70, 133.31, 132.22, 131.78, 130.39, 129.59, 128.85, 128.57, 128.12, 126.79, 123.28, 55.85, 21.24, MS (GC, 70 eV): *m/z* = 451 (M^+, 5%), 280 (8), 105 (100), 79 (12), 77 (12), HRMS (TOF MS ES+): calcd for $C_{17}H_{15}BrN_3O_2Se$ $(M + H)^+$ 451.9513, found 451.9510.

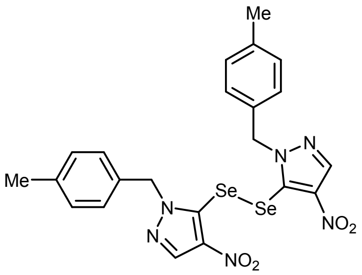

Identification of byproduct 1,2-bis(1-(4-methylbenzyl)-4-nitro-1H-pyrazol-5-yl)diselane (1by): colorless crystals, mp. 59-60 °C, ^{1}H NMR (500 MHz, $CDCl_3$): δ (ppm): 8.22 (s, 2H, C(3)-pyrazole), 7.19 (d, 4H, ^{3}J = 7.9 Hz, CH_{Ar}), 7.10 (d, 4H, ^{3}J = 8.0 Hz, CH_{Ar}), 5.30 (s, 4H, CH_2), 2.33 (s, 6H, CH_3), ^{13}C NMR (126 MHz, $CDCl_3$): δ (ppm): 138.76, 137.47, 131.82, 129.91, 127.75, 125.96, 56.01, 21.33, MS (GC, 70 eV): *m/z* = 592 (M^+, 3%), 296 (37), 279 (11), 176 (7), 121 (26), 105 (100), 79 (17), 77 (17), HRMS (TOF MS ES+): calcd for $C_{22}H_{21}N_6O_4Se_2$ $(M + H)^+$ 592.9955, found 592.9967.

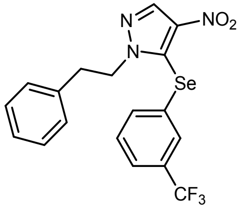

5-(3-(trifluoromethyl)phenylselanyl)-4-nitro-1-phenethyl-1H-pyrazole (2a): light yellow oil, ^{1}H NMR (500 MHz, $CDCl_3$): δ (ppm): 8.31 (s, 1H, C(3)-pyrazole), 7.60 (s, 1H, CH_{Ar}), 7.53 (d, 1H, ^{3}J = 7.6 Hz, CH_{Ar}), 7.38 - 7.28 (m, 2H, CH_{Ar}), 7.27 - 7.20 (m, 3H, CH_{Ar}), 6.99 (dd, 2H, ^{3}J = 7.4 Hz, ^{4}J = 1.9 Hz, CH_{Ar}), 4.58 (t, 2H, ^{3}J = 7.1 Hz, CH_2), 3.13 (t, 2H, ^{3}J = 7.1 Hz, CH_2), ^{13}C NMR (126 MHz, $CDCl_3$): δ (ppm): 137.88, 137.54, 136.70, 135.01, 132.13 (q, $^{2}J_{CF}$ = 33.0 Hz, C_{ipso}-CF_3), 130.32, 129.77, 128.98, 128.88, 128.34 (dd, J_{CF} = 7.5 Hz, J_{CF} = 3.9 Hz), 127.51, 127.39, 125.33 (q, J_{CF} = 3.6 Hz), 123.41 (q, $^{1}J_{CF}$ = 272.9 Hz, CF_3), 53.11, 36.37, $^{19}F\{^{1}H\}$ NMR (188.34 MHz, $CDCl_3$): δ (ppm): -62.14 (CF_3), MS (GC, 70 eV): *m/z* = 441 (M^+, 7%), 337 (76), 230 (24), 176 (28), 104 (94), 91 (100), 77 (38), 65 (21), HRMS (TOF MS ES+): calcd for $C_{18}H_{15}F_3N_3O_2Se$ $(M + H)^+$ 442.0282, found 442.0285.

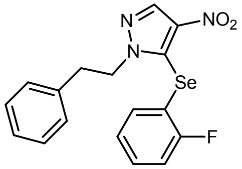

5-(2-fluorophenylselanyl)-4-nitro-1-phenethyl-1H-pyrazole (2b): light yellow oil, ^{1}H NMR (500 MHz, $CDCl_3$): δ (ppm): 8.26 (s, 1H, C(3)-pyrazole), 7.35 - 7.20 (m, 5H, CH_{Ar}), 7.10 - 6.99 (m, 4H, CH_{Ar}), 4.61 (t, 2H, ^{3}J = 7.2 Hz, CH_2), 3.12 (t, 2H, ^{3}J = 7.2 Hz, CH_2), ^{13}C NMR (126 MHz, $CDCl_3$): δ (ppm): 161.23 (d, $^{1}J_{CF}$ = 245.3 Hz, C_{ipso}-F), 137.67, 137.60, 136.87, 134.41, 131.04 (d, J_{CF} = 7.8 Hz), 128.92, 127.29, 127.04, 125.47 (d, J_{CF} = 3.3 Hz), 116.13 (d, $^{2}J_{CF}$ = 22.8 Hz), 115.14 (d, $^{2}J_{CF}$ = 21.4 Hz), 53.04, 36.39, $^{19}F\{^{1}H\}$ NMR (188.34 MHz, $CDCl_3$): δ (ppm): -103.01 (CF), MS (GC, 70 eV): *m/z* = 391 (M^+, 10%), 287 (88), 176 (39), 109 (44), 104 (55), 91 (100), 77 (45), 65 (23), HRMS (TOF MS ES+): calcd for $C_{17}H_{15}FN_3O_2Se$ $(M + H)^+$ 392.0314, found 392.0320.

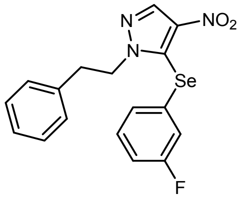

5-(3-fluorophenylselanyl)-4-nitro-1-phenethyl-1H-pyrazole (2c): light yellow oil, ^{1}H NMR (500 MHz, $CDCl_3$): δ (ppm): 8.30 (s, 1H, C(3)-pyrazole), 7.35 - 7.16 (m, 4H, CH_{Ar}), 7.08 - 6.90 (m, 5H, CH_{Ar}), 4.56 (t, 2H, ^{3}J = 7.2 Hz, CH_2), 3.13 (t, 2H, ^{3}J = 7.2 Hz, CH_2), ^{13}C NMR (126 MHz, $CDCl_3$): δ (ppm): 162.85 (d, $^{1}J_{CF}$ = 251.5 Hz, C_{ipso}-F), 137.70, 137.49, 136.76, 131.13 (d, J_{CF} = 8.0 Hz), 130.07 (d, J_{CF} = 7.0 Hz), 128.89, 128.83, 127.63, 127.29, 127.26 (d, J_{CF} = 3.4 Hz), 118.65 (d, $^{2}J_{CF}$ = 23.1 Hz), 115.60 (d, $^{2}J_{CF}$ = 21.0 Hz), 53.00, 36.24, $^{19}F\{^{1}H\}$ NMR (188.34 MHz, $CDCl_3$): δ (ppm): -109.39 (CF), MS (GC, 70 eV): *m/z* = 391 (M^+, 12%), 287 (80), 176 (36), 109 (36), 104 (60), 91 (100), 77 (41), 65 (21), HRMS (TOF MS ES+): calcd for $C_{17}H_{15}FN_3O_2Se$ $(M + H)^+$ 392.0314, found 392.0319.

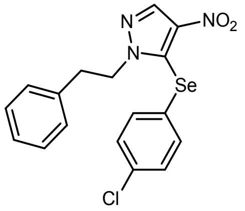

5-(4-chlorophenylselanyl)-4-nitro-1-phenethyl-1H-pyrazole (2d): yellow crystals, mp. 95 °C, ^{1}H NMR (500 MHz, $CDCl_3$): δ (ppm): 8.27 (s, 1H, C(3)-pyrazole), 7.32 - 7.22 (m, 3H, CH_{Ar}), 7.22 - 7.12 (m, 4H, CH_{Ar}), 7.01 (dd, 2H, ^{3}J = 7.3 Hz, ^{4}J = 2.0 Hz, CH_{Ar}), 4.55 (t, 2H, ^{3}J = 7.1 Hz, CH_2), 3.13 (t, 2H, ^{3}J = 7.1 Hz, CH_2), ^{13}C NMR (126 MHz, $CDCl_3$): δ (ppm): 137.73, 137.32, 136.85, 135.03, 133.67, 130.06, 128.95, 128.90, 128.30, 127.30, 126.50, 52.97, 36.23, MS (GC, 70 eV): *m/z* = 407 (M^+, 12%), 303 (60), 280 (15), 176 (40), 125 (23), 104 (48), 91 (100), 77 (51), 65 (23), HRMS (TOF MS ES+): calcd for $C_{17}H_{15}ClN_3O_2Se$ $(M + H)^+$ 408.0018, found 408.0017.

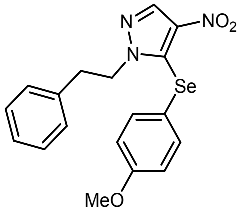

5-(4-methoxyphenylselanyl)-4-nitro-1-phenethyl-1H-pyrazole (2e): yellow viscous oil, ^{1}H NMR (500 MHz, $CDCl_3$): δ (ppm): 8.22 (s, 1H, C(3)-pyrazole), 7.35 (d, 2H, ^{3}J = 8.9 Hz, CH_{Ar}), 7.31 - 7.21 (m, 3H, CH_{Ar}), 7.04 (d, 2H, ^{3}J = 8.0 Hz, CH_{Ar}), 6.79 (d, 2H, ^{3}J = 8.9 Hz, CH_{Ar}), 4.50 - 4.45 (m, 2H, CH_2), 3.77 (s, 3H, OCH_3), 3.11 - 3.03 (m, 2H, CH_2), ^{13}C NMR (126 MHz, $CDCl_3$): δ (ppm): 160.48, 137.40, 137.01, 136.97, 135.58, 129.76, 128.85, 128.84, 127.14, 117.73, 115.55, 55.43, 52.79, 36.11, MS (GC, 70 eV): *m/z* = 403 (M^+, 29%), 299 (17), 280 (22), 123 (100), 105 (25), 91 (37), 77 (24), HRMS (TOF MS ES+): calcd for $C_{18}H_{18}N_3O_3Se$ $(M + H)^+$ 404.0513, found 404.0521.

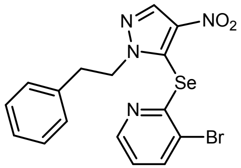

2-(4-nitro-1-phenethyl-1H-pyrazol-5-ylselanyl)-3-bromopyridine (2f): white powder, 120-121 °C, ^{1}H NMR (500 MHz, $CDCl_3$): δ (ppm): 8.34 (s, 1H, C(3)-pyrazole), 8.13 (dd, 1H, ^{3}J = 4.7 Hz, ^{4}J = 1.5 Hz, CH_{Ar}), 7.71 (dd, 1H, ^{3}J = 7.9 Hz, ^{4}J = 1.5 Hz, CH_{Ar}), 7.29 - 7.18 (m, 3H, CH_{Ar}), 7.08 - 7.03 (m, 2H, CH_{Ar}), 6.97 (dd, 1H, ^{3}J = 7.9, ^{3}J = 4.7 Hz, CH_{Ar}), 4.54 (t, 2H, ^{3}J = 7.3 Hz, CH_2), 3.17 (t, 2H, ^{3}J = 7.3 Hz, CH_2), ^{13}C NMR (126 MHz, $CDCl_3$): δ (ppm): 154.85, 148.86, 139.71, 138.14, 137.60, 137.06, 128.93, 128.83, 127.18, 122.73, 120.59, 53.29, 36.52, MS (GC, 70 eV): *m/z* = 406 (minus nitro, M^+, 100%), 355 (13), 302 (42), 287 (7), 236 (8), 195 (13), 156 (23), 104 (74), 91 (79), 77 (84), 65 (35), HRMS (TOF MS ES+): calcd for $C_{16}H_{14}BrN_4O_2Se$ $(M + H)^+$ 452.9465, found 452.9467.

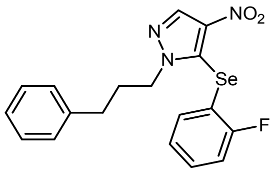

5-(2-fluorophenylselanyl)-4-nitro-1-(3-phenylpropyl)-1H-pyrazole (3a): yellow viscous oil, ^{1}H NMR (500 MHz, $CDCl_3$): δ (ppm): 8.23 (s, 1H, C(3)-pyrazole), 7.35 - 7.27 (m, 4H, CH_{Ar}), 7.21 (t, 1H, ^{3}J = 7.4 Hz, CH_{Ar}), 7.14 - 7.06 (m, 4H, CH_{Ar}), 4.31 (t, 2H, ^{3}J = 7.3 Hz, CH_2), 2.60 (t, 2H, ^{3}J = 7.7 Hz, CH_2), 2.14 (quint, 2H, ^{3}J = 7.6 Hz, CH_2), ^{13}C NMR (126 MHz, $CDCl_3$): δ (ppm): 161.19 (d, $^{1}J_{CF}$ = 245.6 Hz, C_{ipso}-F), 140.28, 137.85, 137.25, 134.33, 131.12 (d, J_{CF} = 7.7 Hz), 128.61, 128.35, 126.34, 126.31 (d, J_{CF} = 8.0 Hz), 125.54 (d, J_{CF} = 3.4 Hz), 116.22 (d, $^{2}J_{CF}$ = 22.7 Hz), 115.02 (d, $^{2}J_{CF}$ = 21.5 Hz), 51.48, 32.61, 31.15, $^{19}F\{^{1}H\}$ NMR (188.34 MHz, $CDCl_3$): δ (ppm): -102.72 (CF), MS (GC, 70 eV): *m/z* = 405 (M^+, 1%), 287 (5), 230 (100), 202 (12), 175 (9), 117 (48), 109 (35), 91 (76), 65 (17), HRMS (TOF MS ES+): calcd for $C_{18}H_{17}FN_3O_2Se$ $(M + H)^+$ 406.0470, found 406.0474.

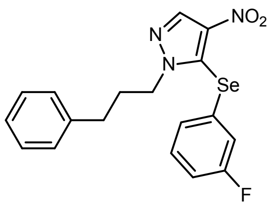

5-(3-fluorophenylselanyl)-4-nitro-1-(3-phenylpropyl)-1H-pyrazole (3b): yellow viscous oil, ^{1}H NMR (500 MHz, $CDCl_3$): δ (ppm): 8.25 (s, 1H, C(3)-pyrazole), 7.36 - 7.26 (m, 4H, CH_{Ar}), 7.19 – 7.14 (m, 3H, CH_{Ar}), 7.11 – 7.04 (m, 2H, CH_{Ar}), 4.23 (t, 2H, ^{3}J = 7.4 Hz, CH_2), 2.58 (t, 2H, ^{3}J = 7.5 Hz, CH_2), 2.11 (quint, 2H, ^{3}J = 7.5 Hz, CH_2), ^{13}C NMR (126 MHz, $CDCl_3$): δ (ppm): 162.94 (d, $^{1}J_{CF}$ = 251.8 Hz, C_{ipso}-F), 140.19, 137.87, 137.37, 131.31 (d, J_{CF} = 8.0 Hz), 129.89 (d, J_{CF} = 7.0 Hz), 128.70, 128.41, 127.60 (d, J_{CF} = 3.2 Hz), 126.82, 126.46, 119.01 (d, $^{2}J_{CF}$ = 23.1 Hz), 115.85 (d, $^{2}J_{CF}$ = 21.1 Hz), 51.52, 32.64, 31.22, ^{19}F{^{1}H} NMR (188.34 MHz, $CDCl_3$): δ (ppm): -109.20 (CF), MS (GC, 70 eV): *m/z* = 405 (M^+, 1%), 287 (4), 230 (100), 202 (10), 175 (7), 117 (45), 109 (21), 91 (61), 65 (14), HRMS (TOF MS ES+): calcd for $C_{18}H_{17}FN_3O_2Se$ $(M + H)^+$ 406.0470, found 406.0472.

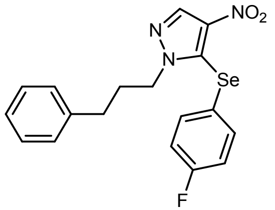

5-(4-fluorophenylselanyl)-4-nitro-1-(3-phenylpropyl)-1H-pyrazole (3c): yellow viscous oil, ^{1}H NMR (500 MHz, $CDCl_3$): δ (ppm): 8.21 (s, 1H, C(3)-pyrazole), 7.41 - 7.38 (m, 2H, CH_{Ar}), 7.30 (t, 2H, ^{3}J = 7.5 Hz, CH_{Ar}), 7.22 (t, 1H, ^{3}J = 7.4 Hz, CH_{Ar}), 7.13 (d, 2H, ^{3}J = 7.2 Hz, CH_{Ar}), 6.97 (t, 2H, ^{3}J = 8.6 Hz, CH_{Ar}), 4.22 (t, 2H, ^{3}J = 7.5 Hz, CH_2), 2.59 (t, 2H, ^{3}J = 7.5 Hz, CH_2), 2.10 (quint, 2H, ^{3}J = 7.5 Hz, CH_2), ^{13}C NMR (126 MHz, $CDCl_3$): δ (ppm): 163.02 (d, $^{1}J_{CF}$ = 250.0 Hz, C_{ipso}-F), 140.18, 137.33, 137.08, 135.24 (d, J_{CF} = 8.2 Hz), 128.54, 128.31, 127.83, 126.29, 122.51 (d, J_{CF} = 3.3 Hz), 117.14 (d, $^{2}J_{CF}$ = 22.0 Hz), 51.27, 32.50, 31.04, ^{19}F{^{1}H} NMR (188.34 MHz, $CDCl_3$): δ (ppm): -110.50 (CF), MS (GC, 70 eV): *m/z* = 405 (M^+, 1%), 287 (4), 230 (100), 202 (10), 175 (6), 117 (42), 109 (42), 91 (57), 65 (13), HRMS (TOF MS ES+): calcd for $C_{18}H_{17}FN_3O_2Se$ $(M + H)^+$ 406.0470, found 406.0473.

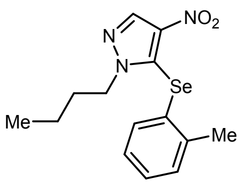

5-(2-methylphenylselanyl)-1-butyl-4-nitro-1H-pyrazole (4a): yellow viscous oil, ^{1}H NMR (500 MHz, $CDCl_3$): δ (ppm): 8.23 (s, 1H, C(3)-pyrazole), 7.24 - 7.20 (m, 2H, CH_{Ar}), 7.13 (d, 1H, ^{3}J = 7.5 Hz, CH_{Ar}), 7.09 - 7.04 (m, 1H, CH_{Ar}), 4.09 (t, 2H, ^{3}J = 7.5 Hz, CH_2), 2.40 (s, 3H, C_{Ar}-CH_3), 1.64 (quint, 2H, ^{3}J = 7.5 Hz, CH_2), 1.19 (sext, 2H, ^{3}J = 7.5 Hz, CH_2), 0.84 (t, 3H, ^{3}J = 7.3 Hz, CH_3), ^{13}C NMR (126 MHz, $CDCl_3$): δ (ppm): 139.34, 137.63, 137.19, 132.34, 131.05, 129.34, 128.87, 127.83, 127.45, 51.86, 31.75, 22.18, 19.72, 13.60, MS (GC, 70 eV): *m/z* = 339 (M^+, 30%), 322 (9), 232 (100), 186 (15), 176 (25), 168 (58), 128 (10), 115 (17), 105 (63), 91 (96), 77 (18), 65 (31), 55 (26), 41 (45), HRMS (TOF MS ES+): calcd for $C_{14}H_{18}N_3O_2Se$ $(M + H)^+$ 340.0564, found 340.0568.

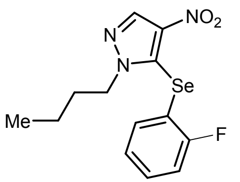

5-(2-fluorophenylselanyl)-1-butyl-4-nitro-1H-pyrazole (4b): yellow viscous oil, ^{1}H NMR (500 MHz, $CDCl_3$): δ (ppm): 8.24 (s,1H, C(3)-pyrazole), 7.30 - 7.23 (m, 1H, CH_{Ar}), 7.15 (d, 1H, ^{3}J = 7.8 Hz, CH_{Ar}), 7.11 - 7.05 (m, 1H, CH_{Ar}), 7.01 (td, 1H, ^{3}J = 8.4 Hz, ^{4}J = 2.4 Hz, CH_{Ar}), 4.24 (t, 2H, ^{3}J = 7.4 Hz, CH_2), 1.73 (quint, 2H, ^{3}J = 7.5 Hz, CH_2), 1.26 (sext, 2H, ^{3}J = 7.5 Hz, CH_2), 0.89 (t, 3H, ^{3}J = 7.3 Hz, CH_3), ^{13}C NMR (126 MHz, $CDCl_3$): δ (ppm): 162.99 (d, $^{1}J_{CF}$ = 251.7 Hz, C_{ipso}-F), 137.76, 137.33, 131.29 (d, J_{CF} = 8.1 Hz), 130.03 (d, J_{CF} = 7.1 Hz), 127.48 (d, J_{CF} = 3.2 Hz), 126.66, 118.88 (d, $^{2}J_{CF}$ = 23.1 Hz), 115.78 (d, $^{2}J_{CF}$ = 21.1 Hz), 51.97, 32.04, 19.74, 13.64, ^{19}F{^{1}H} NMR (188.34 MHz, $CDCl_3$): δ (ppm): -109.41 (CF), MS (GC, 70 eV): *m/z* = 343 (M^+, 10%), 339 (21), 322 (5), 232 (97), 186 (11), 176 (29), 168 (100), 115 (10), 105 (39), 91 (44), 77 (9), 65 (17), 55 (27), 41 (38), HRMS (TOF MS ES+): calcd for $C_{13}H_{15}FN_3O_2Se$ $(M + H)^+$ 344.0314, found 344.0326.

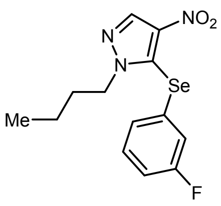

5-(3-fluorophenylselanyl)-1-butyl-4-nitro-1H-pyrazole (4c): yellow viscous oil, ^{1}H NMR (500 MHz, $CDCl_3$): δ (ppm): 8.21 (s,1H, C(3)-pyrazole), 7.39 - 7.28 (m, 2H, CH_{Ar}), 7.08 (q, 2H, ^{3}J = 8.2 Hz, CH_{Ar}), 4.28 (t, 2H, ^{3}J = 7.4 Hz, CH_2), 1.74 (quint, 2H, ^{3}J = 7.5 Hz, CH_2), 1.27 (sext, 2H, ^{3}J = 7.5 Hz, CH_2), 0.89 (t, 3H, ^{3}J = 7.4 Hz, CH_3), ^{13}C NMR (126 MHz, $CDCl_3$): δ (ppm): 161.21 (d, $^{1}J_{CF}$ = 245.5 Hz, C_{ipso}-F), 137.81, 137.23, 134.29, 131.09 (d, J_{CF} = 7.7 Hz), 126.11, 125.55 (d, J_{CF} = 3.4 Hz), 116.22 (d, $^{2}J_{CF}$ = 22.7 Hz), 115.12 (d, $^{2}J_{CF}$ = 21.4 Hz), 51.92, 32.03, 19.73, 13.63, ^{19}F{^{1}H} NMR (188.34 MHz, $CDCl_3$): δ (ppm): -102.91 (CF), MS (GC, 70 eV): *m/z* = 343 (M^+, 11%), 232 (42), 168 (100), 109 (53), 91 (12), 55 (17), 41 (25), HRMS (TOF MS ES+): calcd for $C_{13}H_{15}FN_3O_2Se$ $(M + H)^+$ 344.0314, found 344.0316.

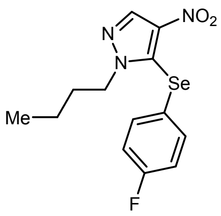

5-(4-fluorophenylselanyl)-1-butyl-4-nitro-1H-pyrazole (4d): yellow oil, ^{1}H NMR (500 MHz, $CDCl_3$): δ (ppm): 8.16 (s, 1H, C(3)-pyrazole), 7.46 (d, 1H, ^{3}J = 8.4 Hz, CH_{Ar}), 7.45 (d, 1H, ^{3}J = 8.4 Hz, CH_{Ar}), 6.98 (t, 2H, ^{3}J = 8.6 Hz, CH_{Ar}), 4.20 (t, 2H, ^{3}J = 7.4 Hz, CH_2), 1.69 (quint, 2H, ^{3}J = 7.5 Hz, CH_2), 1.23 (sext, 2H, ^{3}J = 7.5 Hz, CH_2), 0.87 (t, 3H, ^{3}J = 7.3 Hz, CH_3), ^{13}C NMR (126 MHz, $CDCl_3$): δ (ppm): 163.08 (d, $^{1}J_{CF}$ = 250.0 Hz, C_{ipso}-F), 137.28, 137.09, 135.15 (d, J_{CF} = 8.2 Hz), 127.72, 122.67 (d, J_{CF} = 3.4 Hz), 117.15 (d, $^{2}J_{CF}$ = 22.0 Hz), 51.75, 31.89, 19.65, 13.55, ^{19}F{^{1}H} NMR (188.34 MHz, $CDCl_3$): δ (ppm): -110.75 (CF), MS (GC, 70 eV): *m/z* = 343 (M^+, 23%), 232 (76), 190 (17), 175 (43), 168 (99), 109 (100), 95 (24), 83 (25), 55 (39), 41 (53), HRMS (TOF MS ES+): calcd for $C_{13}H_{15}FN_3O_2Se$ $(M + H)^+$ 344.0314, found 344.0317.

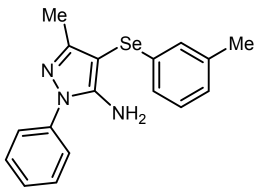

4-(m-tolylselanyl)-3-methyl-1-phenyl-1H-pyrazol-5-amine (5a): dark brown viscous oil, ^{1}H NMR (500 MHz, $CDCl_3$): δ (ppm): 7.61 (dd, 2H, ^{3}J = 8.5 Hz, ^{4}J = 1.1 Hz, CH_{Ar}), 7.49 (t, 2H, ^{3}J = 7.9 Hz, CH_{Ar}), 7.35 (t, 1H, ^{3}J = 7.4 Hz, CH_{Ar}), 7.11 (t, 1H, ^{3}J = 7.6 Hz, CH_{Ar}), 7.06 (br.s, 1H, CH_{Ar}), 7.01 (d, 1H, ^{3}J = 7.8 Hz, CH_{Ar}), 6.97 (d, 1H, ^{3}J = 7.4 Hz, CH_{Ar}), 4.23 (br. s, 2H, NH_2), 2.293 (s, 3H, CH_3), 2.290 (s, 3H, CH_3), ^{13}C NMR (126 MHz, $CDCl_3$): δ (ppm): 153.31, 148.69, 139.21, 138.75, 132.74, 129.74, 129.19, 128.64, 127.56, 126.95, 125.10, 123.54, 84.76, 21.54, 13.25, MS (GC, 70 eV): *m/z* = 343 (M^+, 26%), 263 (100), 222 (13), 131 (15), 119 (24), 91 (15), 77 (45), 66 (12), HRMS (TOF MS ES+): calcd for $C_{17}H_{18}N_3Se$ $(M + H)^+$ 344.0666, found 344.0676.

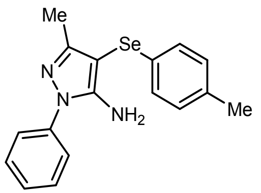

4-(p-tolylselanyl)-3-methyl-1-phenyl-1H-pyrazol-5-amine (5b): pale yellow crystals, mp. 98-99 °C, ^{1}H NMR (500 MHz, $CDCl_3$): δ (ppm): 7.58 (dd, 2H, ^{3}J = 8.5 Hz, ^{4}J = 1.0 Hz, CH_{Ar}), 7.47 (t, 2H, ^{3}J = 7.9 Hz, CH_{Ar}), 7.33 (t, 1H, ^{3}J = 7.4 Hz, CH_{Ar}), 7.14 (d, 2H, ^{3}J = 8.2 Hz, CH_{Ar}), 7.04 (d, 2H, ^{3}J = 8.00 Hz, CH_{Ar}), 4.23 (br. s, 2H, NH_2), 2.287 (s, 3H, CH_3), 2.282 (s, 3H, CH_3), ^{13}C NMR (126 MHz, $CDCl_3$): δ (ppm): 153.10, 148.51, 138.81, 135.75, 130.06, 129.58, 129.06, 128.26, 127.32, 123.36, 84.94, 20.99, 13.19, MS (GC, 70 eV): *m/z* = 343 (M^+, 27%), 263 (100), 222 (12), 131 (11), 119 (17), 91 (14), 77 (35), HRMS (TOF MS ES+): calcd for $C_{17}H_{18}N_3Se$ $(M + H)^+$ 344.0666, found 344.0674.

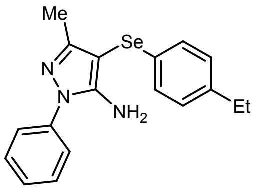

4-(4-ethylphenylselanyl)-3-methyl-1-phenyl-1H-pyrazol-5-amine (5c): dark brown viscous oil, ^{1}H NMR (500 MHz, $CDCl_3$): δ (ppm): 7.58 (dd, 2H, ^{3}J = 8.5 Hz, ^{4}J = 1.0 Hz, CH_{Ar}), 7.47 (t, 2H, ^{3}J = 7.9 Hz, CH_{Ar}), 7.33 (t, 1H, ^{3}J = 7.4 Hz, CH_{Ar}), 7.16 (d, 2H, ^{3}J = 8.2 Hz, CH_{Ar}), 7.06 (d, 2H, ^{3}J = 8.2 Hz, CH_{Ar}), 4.23 (br. s, 2H, NH_2), 2.58 (q, 2H, ^{3}J = 7.6 Hz, CH_2), 2.28 (s, 3H, CH_3), 1.20 (t, 3H, ^{3}J = 7.6 Hz, CH_3), ^{13}C NMR (126 MHz, $CDCl_3$): δ (ppm): 153.19, 148.58, 142.21, 138.79, 129.61, 129.36, 128.92, 128.27, 127.38, 123.43, 84.91, 28.41, 15.65, 13.19, MS (GC, 70 eV): *m/z* = 357 (M^+, 25%), 277 (100), 262 (32), 131 (9), 119 (16), 77 (34), HRMS (TOF MS ES+): calcd for $C_{18}H_{20}N_3Se$ $(M + H)^+$ 358.0822, found 358.0831.

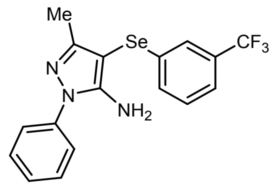

4-(3-(trifluoromethyl)phenylselanyl)-3-methyl-1-phenyl-1H-pyrazol-5-amine (5d): dark green powder, mp. 104-105 °C, ^{1}H NMR (500 MHz, $CDCl_3$): δ (ppm): 7.58 (dd, 2H, ^{3}J = 8.5 Hz, ^{4}J = 1.0 Hz, CH_{Ar}), 7.60 - 7.47 (m, 3H, CH_{Ar}), 7.40 (d, 1H, ^{3}J = 7.3 Hz, CH_{Ar}), 7.37 - 7.30 (m, 3H, CH_{Ar}), 4.25 (br. s, 2H, NH_2), 2.26 (s, 3H, CH_3), ^{13}C NMR (126 MHz, $CDCl_3$): δ (ppm): 153.12, 148.78, 138.65, 134.59, 131.54 (q, $^{2}J_{CF}$ = 32.2 Hz, C_{ipso}-CF_3), 123.88 (q, $^{1}J_{CF}$ = 272.8 Hz, CF_3), 131.02, 129.74, 129.59, 127.68, 124.52 (dd, J_{CF} = 7.6 Hz, J_{CF} = 3.7 Hz), 123.61, 122.75 (dd, J_{CF} = 7.1 Hz, J_{CF} = 3.4 Hz), 83.50, 13.14, ^{19}F{^{1}H} NMR (188.34 MHz, $CDCl_3$): δ (ppm): -62.09 (CF_3), MS (GC, 70 eV): *m/z* = 397 (M^+, 40%), 317 (100), 276 (13), 131 (19), 119 (36), 77 (53), HRMS (TOF MS ES+): calcd for $C_{17}H_{15}F_3N_3Se$ $(M + H)^+$ 398.0383, found 398.0392.

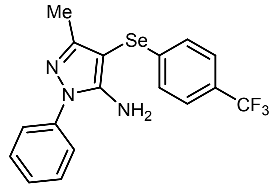

4-(4-(trifluoromethyl)phenylselanyl)-3-methyl-1-phenyl-1H-pyrazol-5-amine (5e): dark brown viscous oil, ^{1}H NMR (500 MHz, $CDCl_3$): δ (ppm): 7.58 (dd, 2H, ^{3}J = 8.5 Hz, ^{4}J = 1.1 Hz, CH_{Ar}), 7.51 - 7.42 (m, 4H, CH_{Ar}), 7.35 (t, 1H, ^{3}J = 7.4 Hz, CH_{Ar}), 7.30 (m, 2H, CH_{Ar}), 4.25 (br. s, 2H, NH_2), 2.25 (s, 3H, CH_3), ^{13}C NMR (126 MHz, $CDCl_3$): δ (ppm): 153.13, 148.78, 138.69, 138.62, 129.70, 128.07 (q, $^{2}J_{CF}$ = 32.5 Hz, C_{ipso}-CF_3), 127.62, 125.94 (dd, J_{CF} = 7.1 Hz, J_{CF} = 3.4 Hz), 124.28 (q, $^{1}J_{CF}$ = 271.9 Hz, CF_3), 123.49, 83.23, 77.16, 13.10, ^{19}F{^{1}H} NMR (188.34 MHz, $CDCl_3$): δ (ppm): -61.77 (CF_3), MS (GC, 70 eV): *m/z* = 397 (M^+, 43%), 317 (100), 276 (13), 131 (16), 119 (33), 77 (48), HRMS (TOF MS ES+): calcd for $C_{17}H_{15}F_3N_3Se$ $(M + H)^+$ 398.0383, found 398.0393.

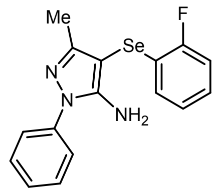

4-(2-fluorophenylselanyl)-3-methyl-1-phenyl-1H-pyrazol-5-amine (5f): light brown powder, mp. 97-99 °C, ^{1}H NMR (500 MHz, $CDCl_3$): δ (ppm): 7.55 (dd, 2H, ^{3}J = 8.5 Hz, ^{4}J = 1.0 Hz, CH_{Ar}), 7.44 (t, 2H, ^{3}J = 7.9 Hz, CH_{Ar}), 7.31 (t, 1H, ^{3}J = 7.4 Hz, CH_{Ar}), 7.16 - 7.09 (m, 1H, CH_{Ar}), 7.02 - 6.94 (m, 3H, CH_{Ar}), 4.28 (br. s, 2H, NH_2), 2.24 (s, 3H, CH_3), ^{13}C NMR (126 MHz, $CDCl_3$): δ (ppm): 160.15 (d, $^{1}J_{CF}$ = 241.1 Hz, C_{ipso}-F), 153.30, 148.93, 138.57, 129.52, 129.49 (d, J_{CF} = 7.3 Hz), 127.59 (d, J_{CF} = 7.3 Hz), 127.36, 124.88 (d, J_{CF} = 3.0 Hz), 123.30, 119.52 (d, $^{2}J_{CF}$ = 22.2 Hz), 115.20 (d, $^{2}J_{CF}$ = 22.1 Hz), 81.63, 12.99, ^{19}F{^{1}H} NMR (188.34 MHz, $CDCl_3$): δ (ppm): -107.38 (CF), MS (GC, 70 eV): *m/z* = 347 (M^+, 39%), 267 (100), 250 (9), 226 (10), 131 (16), 119 (30), 77 (51), HRMS (TOF MS ES+): calcd for $C_{16}H_{15}FN_3Se$ $(M + H)^+$ 348.0415, found 348.0424.

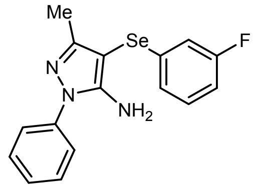

4-(3-fluorophenylselanyl)-3-methyl-1-phenyl-1H-pyrazol-5-amine (5g): black viscous oil, ^{1}H NMR (500 MHz, $CDCl_3$): δ (ppm): 7.59 (d, 2H, ^{3}J = 7.6 Hz, CH_{Ar}), 7.48 (t, 2H, ^{3}J = 7.9 Hz, CH_{Ar}), 7.35 (t, 1H, ^{3}J = 7.4 Hz, CH_{Ar}), 7.17 (td, 1H, ^{3}J = 8.0 Hz, ^{3}J = 5.9 Hz, CH_{Ar}), 7.01 (d, 1H, ^{3}J = 7.8 Hz, CH_{Ar}), 6.92 - 6.87 (m, 1H, CH_{Ar}), 6.84 (td, 1H, ^{3}J = 8.4 Hz, ^{3}J = 2.4 Hz), 4.23 (br. s, 2H, NH_2), 2.26 (s, 3H, CH_3), ^{13}C NMR (126 MHz, $CDCl_3$): δ (ppm): 163.33 (d, $^{1}J_{CF}$ = 249.2 Hz, C_{ipso}-F), 153.11, 148.69, 138.65, 135.37 (d, J_{CF} = 6.2 Hz), 130.42 (d, J_{CF} = 8.0 Hz), 129.66, 127.55, 123.51, 123.35 (d, J_{CF} = 2.8 Hz), 114.68 (d, $^{2}J_{CF}$ = 23.3 Hz), 112.84 (d, $^{2}J_{CF}$ = 21.4 Hz), 83.80, 13.11, ^{19}F{^{1}H} NMR (188.34 MHz, $CDCl_3$): δ (ppm): -111.27 (CF), MS (GC, 70 eV): *m/z* = 347 (M^+, 35%), 267 (100), 226 (13), 131 (15), 119 (30), 77 (49), HRMS (TOF MS ES+): calcd for $C_{16}H_{15}FN_3Se$ $(M + H)^+$ 348.0415, found 348.0422.

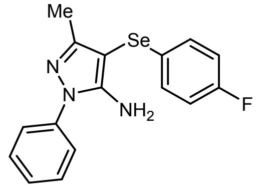

4-(4-fluorophenylselanyl)-3-methyl-1-phenyl-1H-pyrazol-5-amine (5h): light brown viscous oil, ^{1}H NMR (500 MHz, $CDCl_3$): δ (ppm): 7.58 (dd, 2H, ^{3}J = 8.5 Hz, ^{4}J = 1.2 Hz, CH_{Ar}), 7.50 - 7.45 (m, 2H, CH_{Ar}), 7.34 (t, 1H, ^{3}J = 7.4 Hz, CH_{Ar}), 7.22 - 7.17 (m, 2H, CH_{Ar}), 6.93 (t, 2H, ^{3}J = 8.8 Hz, CH_{Ar}), 4.24 (br. s, 2H, NH_2), 2.27 (s, 3H, CH_3), ^{13}C NMR (126 MHz, $CDCl_3$): δ (ppm): 161.76 (d, $^{1}J_{CF}$ = 244.8 Hz, C_{ipso}-F), 153.04, 148.57, 138.73, 129.97 (d, J_{CF} = 7.6 Hz), 129.70, 127.55, 127.32 (d, J_{CF} = 2.9 Hz), 123.49, 116.42 (d, $^{2}J_{CF}$ = 21.7 Hz), 84.99, 13.20, ^{19}F{^{1}H} NMR (188.34 MHz, $CDCl_3$): δ (ppm): -116.26 (CF), MS (GC, 70 eV): *m/z* = 347 (M^+, 31%), 267 (100), 226 (14), 131 (12), 119 (25), 77 (45), 66 (14), HRMS (TOF MS ES+): calcd for $C_{16}H_{15}FN_3Se$ $(M + H)^+$ 348.0415, found 348.0426.

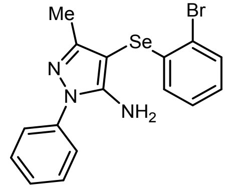

4-(2-bromophenylselanyl)-3-methyl-1-phenyl-1H-pyrazol-5-amine (5i): green crystals, mp. 109-110 °C, ^{1}H NMR (500 MHz, $CDCl_3$): δ (ppm): 7.60 (dd, 2H, ^{3}J = 8.5 Hz, ^{4}J = 1.1 Hz, CH_{Ar}), 7.53 - 7.44 (m, 3H, CH_{Ar}), 7.36 (t, 1H, ^{3}J = 7.4 Hz, CH_{Ar}), 7.13 (td, 1H, ^{3}J = 7.8 Hz, ^{4}J = 1.3 Hz, CH_{Ar}), 7.03 (td, 1H, ^{3}J = 7.6 Hz, ^{4}J = 1.6 Hz, CH_{Ar}), 6.85 (dd, 1H, ^{3}J = 7.9 Hz, ^{4}J = 1.6 Hz, CH_{Ar}), 4.25 (br. s, 2H, NH_2), 2.26 (s, 3H, CH_3), ^{13}C NMR (126 MHz, $CDCl_3$): δ (ppm): 153.55, 149.01, 138.61, 135.48, 132.90, 129.77, 128.03, 127.95, 127.70, 127.08, 123.59, 122.16, 84.35, 13.14, MS (GC, 70 eV): *m/z* = 407 (M^+, 40), 329 (46), 327 (46), 247 (30), 233 (11), 156 (12), 131 (35), 119 (56), 104 (14), 77 (100), 66 (28), 51 (24), 39 (17), HRMS (TOF MS ES+): calcd for $C_{16}H_{15}BrN_3Se$ $(M + H)^+$ 407.9615, found 407.9617.

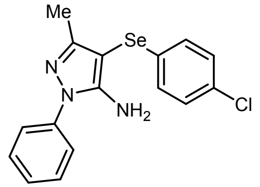

4-(4-chlorophenylselanyl)-3-methyl-1-phenyl-1H-pyrazol-5-amine (5j): dark brown viscous oil, ^{1}H NMR (500 MHz, $CDCl_3$): δ (ppm): 7.59 (dd, 2H, ^3J = 8.5 Hz, ^4J = 1.1 Hz, CH_{Ar}), 7.50 (t, 2H, ^3J = 7.9 Hz, CH_{Ar}), 7.37 (t, 1H, ^3J = 7.4 Hz, CH_{Ar}), 7.21 - 7.13 (m, 4H, CH_{Ar}), 4.24 (br. s, 2H, NH_2), 2.27 (s, 3H, CH_3), ^{13}C NMR (126 MHz, $CDCl_3$): δ (ppm): 153.04, 148.63, 138.64, 131.91, 131.30, 129.67, 129.33, 129.27, 127.54, 123.45, 84.22, 13.14, MS (GC, 70 eV): *m/z* = 363 (M^+, 29%), 283 (100), 247 (12), 131 (14), 119 (32), 77 (61), 66 (18), 51 (15), 39 (11), HRMS (TOF MS ES+): calcd for $C_{16}H_{15}ClN_3Se$ $(M + H)^+$ 364.0120, found 364.0122.

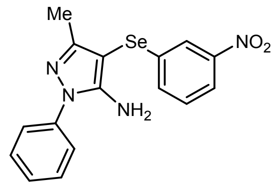

4-(3-nitrophenylselanyl)-3-methyl-1-phenyl-1H-pyrazol-5-amine (5k): green powder, mp. 150-151 °C, ^{1}H NMR (500 MHz, $CDCl_3$): δ (ppm): 8.08 (t, 1H, ^4J = 1.9 Hz, CH_{Ar}), 8.00 (ddd, 1H, ^3J = 8.0 Hz, ^4J = 2.2 Hz, ^4J = 0.9 Hz, CH_{Ar}), 7.60 (dd, 2H, ^3J = 8.5 Hz, ^4J = 1.1 Hz, CH_{Ar}), 7.54 - 7.47 (m, 3H, CH_{Ar}), 7.38 (t, 2H, ^3J = 8.0 Hz, CH_{Ar}), 4.28 (br. s, 2H, NH_2), 2.27 (s, 3H, CH_3), ^{13}C NMR (126 MHz, $CDCl_3$): δ (ppm): 153.05, 148.94, 148.89, 138.46, 135.88, 133.64, 129.93, 129.86, 127.95, 123.79, 122.68, 120.99, 83.21, 13.17, MS (GC, 70 eV): *m/z* = 374 (M^+, 40%), 294 (100), 247 (17), 131 (30), 119 (52), 77 (70), 66 (16), 51 (16), 39 (11), HRMS (TOF MS ES+): calcd for $C_{16}H_{15}N_4O_2Se$ $(M + H)^+$ 375.0360, found 375.0365.

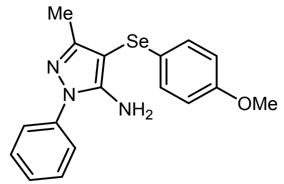

4-(4-methoxyphenylselanyl)-3-methyl-1-phenyl-1H-pyrazol-5-amine (5l): dark yellow viscous oil, ^{1}H NMR (500 MHz, $CDCl_3$): δ (ppm): 7.58 (dd, 2H, ^3J = 8.5 Hz, ^4J = 1.1 Hz, CH_{Ar}), 7.48 (t, 2H, ^3J = 7.9 Hz, CH_{Ar}), 7.34 (t, 1H, ^3J = 7.4 Hz, CH_{Ar}), 7.21 (d, 2H, ^3J = 8.9 Hz, CH_{Ar}), 6.79 (d, 2H, ^3J = 8.8 Hz, CH_{Ar}), 4.23 (br. s, 2H, NH_2), 3.76 (s, 3H, OCH_3), 2.29 (s, 3H, CH_3), ^{13}C NMR (126 MHz, $CDCl_3$): δ (ppm): 158.63, 153.03, 148.45, 138.77, 130.45, 129.72, 127.52, 123.53, 122.79, 115.14, 85.95, 55.46, 13.26, MS (GC, 70 eV): *m/z* = 359 (M^+, 19%), 279 (100), 264 (35), 119 (20), 77 (42), 66 (10), HRMS (TOF MS ES+): calcd for $C_{17}H_{18}N_3OSe$ $(M + H)^+$ 360.0615, found 360.0631.

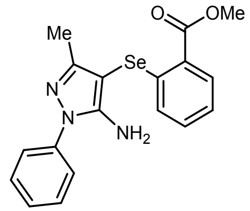

methyl 2-[(5-amino-3-methyl-1-phenyl-1H-pyrazol-4-yl)selanyl]benzoate (5m): dark green crystals, mp. 160-163 °C, ^{1}H NMR (500 MHz, $CDCl_3$): δ (ppm): 8.07 (dd, 1H, ^{3}J = 7.8 Hz, ^{4}J = 1.4 Hz, CH_{Ar}), 7.61 (dd, 2H, ^{3}J = 8.4 Hz, ^{4}J = 1.0 Hz, CH_{Ar}), 7.48 (t, 2H, ^{3}J = 7.9 Hz, CH_{Ar}), 7.36 - 7.28 (m, 2H, CH_{Ar}), 7.22 - 7.17 (m, 1H, CH_{Ar}), 7.13 (dd, 1H, ^{3}J = 8.9 Hz, ^{4}J = 0.8 Hz, CH_{Ar}), 4.21 (br. s, 2H, NH_2), 3.97 (s, 3H, $COOCH_3$), 2.22 (s, 3H, CH_3), ^{13}C NMR (126 MHz, $CDCl_3$): δ (ppm): 167.64, 153.82, 149.09, 139.31, 138.82, 133.00, 131.82, 129.66, 127.70, 127.42, 125.09, 123.46, 85.23, 52.43, 13.19, MS (GC, 70 eV): *m/z* = 387 (M^+, 100%), 307 (64), 275 (75), 252 (22), 215 (20), 184 (14), 172 (43), 157 (12), 131 (44), 119 (61), 104 (17), 91 (16), 77 (95), 66 (21), 51 (17), 39 (11), HRMS (TOF MS ES+): calcd for $C_{18}H_{18}N_3O_2Se$ $(M + H)^+$ 388.0564, found 388.0578.

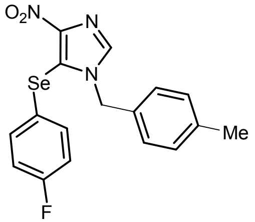

5-(4-fluorophenylselanyl)-1-[(4-methylphenyl)methyl]-4-nitro-1H-imidazole (6): light yellow crystals, mp. 114-115 °C, ^{1}H NMR (500 MHz, $CDCl_3$): δ (ppm): 7.55 (s, 1H, C(2)-imidazole), 7.36 - 7.28 (m, 2H, CH_{Ar}), 7.12 (d, 2H, ^{3}J = 7.9 Hz, CH_{Ar}), 6.95 - 6.88 (m, 4H, CH_{Ar}), 5.17 (s, 2H, CH_2), 2.34 (s, 3H, CH_3), ^{13}C NMR (126 MHz, $CDCl_3$): δ (ppm): 163.06 (d, $^{1}J_{CF}$ = 249.6 Hz, C_{ipso}-F), 150.69, 138.99, 137.88, 134.90 (d, J_{CF} = 8.1 Hz), 131.05, 130.02, 127.55, 123.03 (d, J_{CF} = 3.5 Hz), 117.80, 117.07 (d, $^{2}J_{CF}$ = 22.0 Hz), 51.06, 21.23, ^{19}F{^{1}H} NMR (188.34 MHz, $CDCl_3$): δ (ppm): -111.27 (CF), MS (GC, 70 eV): *m/z* = 391 (M^+, 8%), 280 (3), 105 (100), 79 (14), HRMS (TOF MS ES+): calcd for $C_{17}H_{15}FN_3O_2Se$ $(M + H)^+$ 392.0314, found 392.0313.

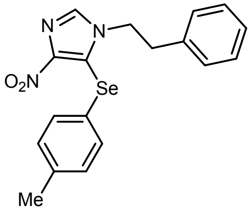

5-(p-tolylselanyl)-4-nitro-1-phenethyl-1H-imidazole (7a): yellow powder, mp. 94-95 °C, ^{1}H NMR (500 MHz, $CDCl_3$): δ (ppm): 7.32 (d, 2H, ^3J = 8.1 Hz, CH_{Ar}), 7.30 - 7.22 (m, 4H, CH_{Ar}), 7.09 (d, 2H, ^3J = 8.0 Hz CH_{Ar}), 6.95 (d, 2H, ^3J = 6.4 Hz, CH_{Ar}), 4.22 (t, 2H, ^3J = 7.1 Hz, CH_2), 2.90 (t, 2H, ^3J = 7.1 Hz, CH_2), 2.31 (s, 3H, CH_3), ^{13}C NMR (126 MHz, $CDCl_3$): δ (ppm): 150.46, 139.01, 137.73, 136.32, 132.39, 130.85, 129.16, 128.74, 127.55, 125.02, 48.99, 36.86, 21.27, MS (GC, 70 eV): *m/z* = 387 (M^+, 8%), 280 (27), 105 (100), 91 (33), 79 (23), HRMS (TOF MS ES+): calcd for $C_{18}H_{18}N_3O_2Se$ $(M + H)^+$ 388.0564, found 388.0566.

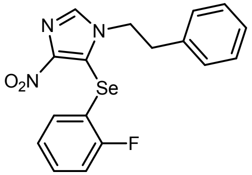

5-(2-fluorophenylselanyl)-4-nitro-1-phenethyl-1H-imidazole (7b): yellow powder, mp. 100-101 °C, ^{1}H NMR (500 MHz, $CDCl_3$): δ (ppm): 7.33 (s, 1H, C(2)-imidazole), 7.33 - 7.22 (m, 5H, CH_{Ar}), 7.10 - 7.03 (m, 2H, CH_{Ar}), 7.02 - 6.97 (m, 2H, CH_{Ar}), 4.37 (t, 2H, ^3J = 7.0 Hz, CH_2), 2.97 (t, 2H, ^3J = 7.0 Hz, CH_2), ^{13}C NMR (126 MHz, $CDCl_3$): δ (ppm): 161.05 (d, $^1J_{CF}$ = 244.6 Hz, C_{ipso}-F), 150.96, 137.79, 136.21, 133.89, 130.84 (d, J_{CF} = 7.7 Hz), 129.18, 128.77, 127.60, 125.56 (d, J_{CF} = 3.3 Hz), 116.17 (d, $^2J_{CF}$ = 22.7 Hz), 115.96, 115.64 (d, $^2J_{CF}$ = 21.5 Hz), 48.98, 37.28, ^{19}F{^{1}H} NMR (188.34 MHz, $CDCl_3$): δ (ppm): -103.60 (CF), MS (GC, 70 eV): *m/z* = 391 (M^+, 11%), 280 (18), 105 (100), 91 (48), 79 (29), HRMS (TOF MS ES+): calcd for $C_{17}H_{15}FN_3O_2Se$ $(M + H)^+$ 392.0314, found 392.0316.

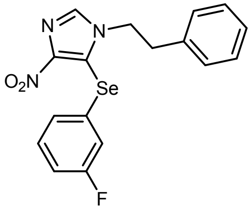

5-(3-fluorophenylselanyl)-4-nitro-1-phenethyl-1H-imidazole (7c): yellow powder, mp. 71-72 °C, ^{1}H NMR (500 MHz, $CDCl_3$): δ (ppm): 7.35 (s, 1H, C(2)-imidazole), 7.30 - 7.21 (m, 4H, CH_{Ar}), 7.10 (d, 1H, ^{3}J = 7.8 Hz, CH_{Ar}), 7.02 (d, 1H, ^{3}J = 8.3 Hz, CH_{Ar}), 6.99 - 6.94 (m, 3H, CH_{Ar}), 4.29 (t, 2H, ^{3}J = 7.0 Hz, CH_2), 2.94 (t, 2H, ^{3}J = 7.0 Hz, CH_2), ^{13}C NMR (126 MHz, $CDCl_3$): δ (ppm): 163.00 (d, $^{1}J_{CF}$ = 251.6 Hz, C_{ipso}-F), 150.96, 138.04, 136.05, 131.26 (d, J_{CF} = 8.1 Hz), 130.69 (d, J_{CF} = 6.9 Hz), 129.15, 128.67, 127.58, 126.66 (d, J_{CF} = 3.1 Hz), 118.06 (d, $^{2}J_{CF}$ = 23.1 Hz), 116.30, 115.46 (d, $^{2}J_{CF}$ = 21.1 Hz), 48.99, 37.02, ^{19}F{^{1}H} NMR (188.34 MHz, $CDCl_3$): δ (ppm): -109.35 (CF), MS (GC, 70 eV): *m/z* = 391 (M^+, 12%), 280 (14), 105 (100), 91 (49), 79 (31), HRMS (TOF MS ES+): calcd for $C_{17}H_{15}FN_3O_2Se$ $(M + H)^+$ 392.0314, found 392.0321.

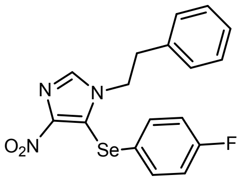

5-(4-fluorophenylselanyl)-4-nitro-1-phenethyl-1H-imidazole (7d): yellow powder, mp. 105-106 °C, ^{1}H NMR (500 MHz, $CDCl_3$): δ (ppm): 7.45 - 7.37 (m, 2H, CH_{Ar}), 7.29 (s, 1H, C(2)-imidazole), 7.28 - 7.22 (m, 3H, CH_{Ar}), 7.01 - 6.93 (m, 4H, CH_{Ar}), 4.29 (t, 2H, ^{3}J = 7.0 Hz, CH_2), 2.94 (t, 2H, ^{3}J = 7.0 Hz, CH_2), ^{13}C NMR (126 MHz, $CDCl_3$): δ (ppm): 163.01 (d, $^{1}J_{CF}$ = 249.7 Hz, C_{ipso}-F), 150.50, 137.75, 136.12, 134.45 (d, J_{CF} = 8.1 Hz), 129.17, 128.68, 127.59, 123.31 (d, J_{CF} = 3.3 Hz), 117.63, 117.25 (d, $^{2}J_{CF}$ = 22.0 Hz), 48.90, 36.99, ^{19}F{^{1}H} NMR (188.34 MHz, $CDCl_3$): δ (ppm): -111.13 (CF), MS (GC, 70 eV): *m/z* = 391 (M^+, 7%), 280 (13), 105 (100), 91 (42), 79 (25), HRMS (TOF MS ES+): calcd for $C_{17}H_{15}FN_3O_2Se$ $(M + H)^+$ 392.0314, found 392.0318.

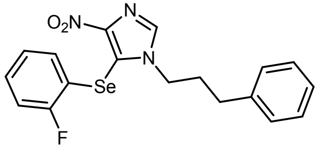

5-(2-fluorophenylselanyl)-4-nitro-1-phenylpropyl-1H-imidazole (8): yellow viscous oil, ^{1}H NMR (500 MHz, $CDCl_3$): δ (ppm): 7.78 (br. s. 1H, C(2)-imidazole), 7.30 - 7.19 (m, 5H, CH_{Ar}), 7.10 - 7.02 (m, 4H, CH_{Ar}), 4.08 (t, 2H, ^{3}J = 7.4 Hz, CH_2), 2.60 (t, 2H, ^{3}J = 7.4 Hz, CH_2), 2.06 (quint, 2H, ^{3}J = 7.4 Hz, CH_2), ^{13}C NMR (126 MHz, $CDCl_3$): δ (ppm): 160.94 (d, $^{1}J_{CF}$ = 244.9 Hz, C_{ipso}-F), 139.54, 133.66, 130.74 (d, J_{CF} = 7.7 Hz), 128.74, 128.24, 126.58, 125.50 (d, J_{CF} = 3.2 Hz), 116.11 (d, $^{2}J_{CF}$ = 22.5 Hz), 115.47 (d, $^{2}J_{CF}$ = 21.4 Hz), 47.36, 32.50, 31.87, ^{19}F{^{1}H} NMR (188.34 MHz, $CDCl_3$): δ (ppm): -103.55 (CF), MS (GC, 70 eV): *m/z* = 405 (M^+, 17%), 294 (13), 230 (26), 184 (38), 117 (68), 91 (100), HRMS (TOF MS ES+): calcd for $C_{18}H_{17}FN_3O_2Se$ $(M + H)^+$ 406.0470, found 406.0469.

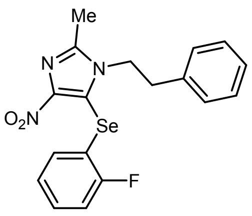

5-(2-fluorophenylselanyl)-2-methyl-4-nitro-1-phenethyl-1H-imidazole (9a): dark yellow powder, mp. 85-86 °C, ^{1}H NMR (500 MHz, $CDCl_3$): δ (ppm): 7.33 - 7.23 (m, 5H, CH_{Ar}), 7.05 (q, 2H, ^{3}J = 7.8 Hz, CH_{Ar}), 7.01 - 6.98 (m, 2H, CH_{Ar}), 4.31 (t, 2H, ^{3}J = 7.1 Hz, CH_2), 2.93 (t, 2H, ^{3}J = 7.1 Hz, CH_2), 2.21 (s, 3H, CH_3), ^{13}C NMR (126 MHz, $CDCl_3$): δ (ppm): 160.92 (d, $^{1}J_{CF}$ = 244.1 Hz, C_{ipso}-F), 150.07, 147.01, 136.40, 133.55, 133.54 (d, J = 1.9 Hz), 130.57 (d, J_{CF} = 7.7 Hz), 129.19, 128.94, 127.62, 125.52 (d, J_{CF} = 3.3 Hz), 116.10 (d, $^{2}J_{CF}$ = 22.3 Hz), 115.31, 47.96, 36.93, 13.96, $^{19}F\{^{1}H\}$ NMR (188.34 MHz, $CDCl_3$): δ (ppm): -103.97 (CF), MS (GC, 70 eV): *m/z* = 405 (M^+, 14%), 294 (12), 230 (12), 213 (12), 184 (8), 105 (100), 91 (43), 79 (30), HRMS (TOF MS ES+): calcd for $C_{18}H_{17}FN_3O_2Se$ $(M + H)^+$ 406.0470, found 406.0475.

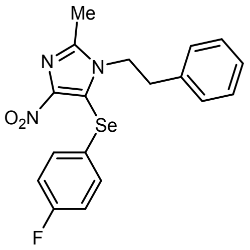

5-(4-fluorophenylselanyl)-2-methyl-4-nitro-1-phenethyl-1H-imidazole (9b): yellow powder, mp. 108-109 °C, ^{1}H NMR (500 MHz, $CDCl_3$): δ (ppm): 7.43 (dd, 2H, ^{3}J = 8.7 Hz, ^{3}J = 5.2 Hz, CH_{Ar}), 7.32 - 7.26 (m, 3H, CH_{Ar}), 6.98 (m, 4H, CH_{Ar}), 4.24 (t, 2H, ^{3}J = 7.1 Hz, CH_2), 2.90 (t, 2H, ^{3}J = 7.1 Hz, CH_2), 2.16 (s, 3H, CH_3), ^{13}C NMR (126 MHz, $CDCl_3$): δ (ppm): 162.98 (d, $^{1}J_{CF}$ = 249.1 Hz, C_{ipso}-F), 149.67, 146.94, 136.36, 134.11 (d, J_{CF} = 8.1 Hz), 129.23, 128.85, 127.66, 123.86 (d, J_{CF} = 2.8 Hz), 117.24 (d, $^{2}J_{CF}$ = 22.0 Hz), 116.96, 47.87, 36.74, 13.93, $^{19}F\{^{1}H\}$ NMR (188.34 MHz, $CDCl_3$): δ (ppm): -111.54 (CF), MS (GC, 70 eV): *m/z* = 405 (M^+, 11%), 294 (18), 230 (10), 213 (10), 105 (100), 91 (36), 79 (26), HRMS (TOF MS ES+): calcd for $C_{18}H_{17}FN_3O_2Se$ $(M + H)^+$ 406.0470, found 406.0466.

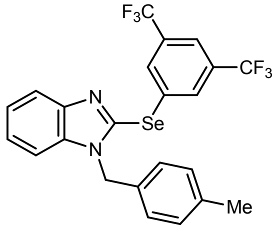

2-(3,5-bis(trifluoromethyl)phenylselanyl)-1-[(4-methylphenyl)methyl]-1H-benzo[d]imidazole (10a): yellow powder, mp. 94-95 °C, ^{1}H NMR (500 MHz, $CDCl_3$): δ (ppm): 7.86 - 7.83 (m, 3H, CH_{Ar}), 7.67 (s, 1H, CH_{Ar}), 7.36 - 7.29 (m, 3H, CH_{Ar}), 6.96 (d, H, ^3J = 7.9 Hz, CH_{Ar}), 6.83 (d, H, ^3J = 7.9 Hz, CH_{Ar}), 5.48 (s, 2H, CH_2), 2.24 (s, 3H, CH_3), ^{13}C NMR (126 MHz, $CDCl_3$): δ (ppm): 141.91, 137.82, 136.11, 132.41, 132.25 – 132.13 (m, C_{ipso}-CF_3), 131.00, 129.53, 126.26, 124.16, 122.98, 122.64 (q, $^1J_{CF}$ = 272.9 Hz, CF_3), 121.72 (m), 120.18, 110.27, 48.90, 20.92. ^{19}F{^{1}H} NMR (188.34 MHz, $CDCl_3$): δ (ppm): -62.37 (CF_3), MS (GC, 70 eV): *m/z* = 514 (M^+, 8%), 433 (3), 317 (11), 105 (100), 79 (12), HRMS (TOF MS ES+): calcd for $C_{23}H_{17}F_6N_2Se$ $(M + H)^+$ 515.0461, found 515.0462.

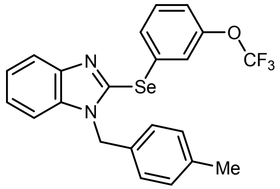

2-(3-(trifluoromethoxy)phenylselanyl)-1-[(4-methylphenyl)methyl]-1H-benzo[d]imidazole (10b): yellow viscous oil, ^{1}H NMR (500 MHz, $CDCl_3$): δ (ppm): 7.85 - 7.83 (m, 1H, CH_{Ar}), 7.40 (d, 1H, ^3J = 7.9 Hz, CH_{Ar}), 7.34 (s, 1H, CH_{Ar}), 7.30 - 7.25 (m, 3H, CH_{Ar}), 7.22 (t, 1H, ^3J = 8.0 Hz, CH_{Ar}), 7.08 (d, 1H, ^3J = 8.3 Hz, CH_{Ar}), 7.02 (d, 2H, ^3J = 7.9 Hz, CH_{Ar}), 6.89 (d, 2H, ^3J = 8.0 Hz, CH_{Ar}), 5.44 (s, 2H, CH_2), 2.28 (s, 3H, CH_3), ^{13}C NMR (126 MHz, $CDCl_3$): δ (ppm): 149.53, 144.01, 143.15, 137.82, 136.04, 132.62, 130.67, 130.64, 130.11, 129.58, 126.58, 124.71, 123.86, 122.81, 120.40 (q, J_{CF} = 257.9 Hz, OCF_3), 120.35, 120.14, 110.48, 49.08, 21.12, ^{19}F{^{1}H} NMR (188.34 MHz, $CDCl_3$): δ (ppm): -57.15 (CF_3), MS (GC, 70 eV): *m/z* = 462 (M^+, 9%), 382 (8), 265 (13), 105 (100), 79 (13), HRMS (TOF MS ES+): calcd for $C_{22}H_{18}F_3N_2OSe$ $(M + H)^+$ 463.0536, found 463.0557.

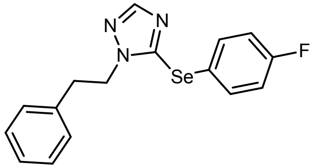

5-(4-fluorophenylselanyl)-1-phenethyl-1H-1,2,4-triazole (11): yellow powder, mp. 87-88 °C, ^{1}H NMR (500 MHz, $CDCl_3$): δ (ppm): 7.97 (s, 1H, C(3)-triazole), 7.45 - 7.41 (m, 2H, CH_{Ar}), 7.28 - 7.22 (m, 3H, CH_{Ar}), 7.04 - 7.02 (m, 2H, CH_{Ar}), 7.00 - 6.95 (m, 2H, CH_{Ar}), 4.44 (t, 2H, ^{3}J = 7.2 Hz, CH_2), 3.10 (t, 2H, ^{3}J = 7.2 Hz, CH_2), ^{13}C NMR (126 MHz, $CDCl_3$): δ (ppm): 163.16 (d, $^{1}J_{CF}$ = 249.4 Hz, C_{ipso}-F), 152.78, 137.16, 135.80 (d, J_{CF} = 8.2 Hz), 128.91, 128.89, 127.14, 121.67 (d, J_{CF} = 3.3 Hz), 117.02 (d, $^{2}J_{CF}$ = 21.9 Hz), 51.24, 36.32, ^{19}F{^{1}H} NMR (188.34 MHz, $CDCl_3$): δ (ppm): -111.33 (CF), MS (GC, 70 eV): *m/z* = 347 (M^+, 37%), 243 (100), 216 (14), 175 (23), 163 (26), 136 (19), 122 (18), 104 (70), 91 (82), 77 (32), 65 (30), 55 (54), HRMS (TOF MS ES+): calcd for $C_{16}H_{15}FN_3Se$ $(M + H)^+$ 348.0415, found 348.0420.

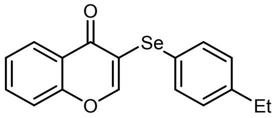

3-(4-ethylphenylselanyl)-4H-chromen-4-one (12a): yellow viscous oil, ^{1}H NMR (500 MHz, $CDCl_3$): δ (ppm): 8.24 (dd, 1H, ^{3}J = 8.3 Hz, ^{4}J = 1.6 Hz, CH_{Ar}), 7.79 (s, 1H, CH_{Ar}), 7.70 - 7.62 (m, 1H, CH_{Ar}), 7.55 (d, 2H, ^{3}J = 8.1 Hz, CH_{Ar}), 7.44 - 7.38 (m, 2H, CH_{Ar}), 7.15 (d, 2H, ^{3}J = 8.1 Hz, CH_{Ar}), 2.63 (q, 2H, ^{3}J = 7.6 Hz, CH_2), 1.22 (t, 3H, ^{3}J = 7.6 Hz, CH_3), ^{13}C NMR (126 MHz, $CDCl_3$): δ (ppm): 175.43, 156.47, 155.03, 144.92, 134.78, 133.88, 129.39, 126.45, 125.60, 124.25, 123.17, 118.68, 118.16, 28.68, 15.55, MS (GC, 70 eV): *m/z* = 330 (M^+, 46%), 279 (15), 249 (48), 235 (38), 210 (23), 195 (17), 167 (45), 149 (100), 115 (48), 105 (16), 89 (16), 71 (25), 57 (33), 43 (23), HRMS (TOF MS ES+): calcd for $C_{17}H_{15}O_2Se$ $(M + H)^+$ 331.0237, found 331.0245.

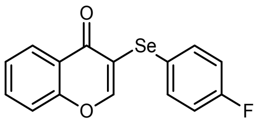

3-(4-fluorophenylselanyl)-4H-chromen-4-one (12b): brown powder, mp. 84-85 °C, ^{1}H NMR (500 MHz, $CDCl_3$): δ (ppm): 8.23 (dd, 1H, ^{3}J = 8.1 Hz, ^{4}J = 1.4 Hz, CH_{Ar}), 7.89 (s, 1H, CH_{Ar}), 7.67 (ddd, 1H, ^{3}J = 8.7 Hz, ^{3}J = 7.2 Hz, ^{4}J = 1.6 Hz, CH_{Ar}), 7.62 (dd, 2H, ^{3}J = 8.8 Hz, ^{3}J = 5.4 Hz, CH_{Ar}), 7.47 - 7.37 (m, 2H, CH_{Ar}), 7.00 (t, 2H, ^{3}J = 8.7 Hz, CH_{Ar}), ^{13}C NMR (126 MHz, $CDCl_3$): δ (ppm): 175.31, 163.09 (d, $^{1}J_{CF}$ = 248.5 Hz, C_{ipso}-F), 156.47, 155.68, 136.55 (d, J_{CF} = 8.1 Hz), 134.04, 126.46, 125.76, 123.26, 122.64 (d, J_{CF} = 3.4 Hz), 118.21, 118.17,

116.90 (d, $^{2}J_{CF}$ = 21.7 Hz), $^{19}F\{^{1}H\}$ NMR (188.34 MHz, $CDCl_3$): δ (ppm): -112.24 (CF), MS (GC, 70 eV): *m/z* = 320 (M^+, 79%), 239 (56), 200 (51), 183 (16), 120 (100), 92 (47), 75 (16), 63 (25), HRMS (TOF MS ES+): calcd for $C_{15}H_{10}FO_2Se$ $(M + H)^+$ 320.9830, found 320.9837.

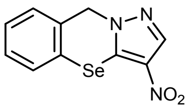

3-nitro-9H-pyrazolo[5,1-b][1,3]benzoselenazine (13a): green powder, mp. 195-196 °C, ^{1}H NMR (500 MHz, $CDCl_3$): 8.18 (s, 1H, C(2)), 7.55 - 7.53 (m, 1H, CH_{Ar}), 7.37 - 7.34 (m, 3H, CH_{Ar}), 5.27 (s, 2H, CH_2), ^{13}C NMR (126 MHz, $CDCl_3$): δ (ppm): 136.55, 131.06, 130.33, 129.43, 129.05, 128.94, 128.37, 125.09, 53.97, MS (GC, 70 eV): *m/z* = 281 (M^+, 100%), 235 (10), 206 (10), 169 (22), 155 (44), 126 (20), 115 (33), 105 (17), 89 (32), 77 (14), 63 (17), HRMS (TOF MS ES+): calcd for $C_{10}H_8N_3O_2Se$ $(M + H)^+$ 281.9782, found 281.9785.

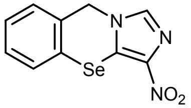

3-nitro-9H-imidazo[5,1-b][1,3]benzoselenazine (13b): yellow powder, mp. 228-229 °C, ^{1}H NMR (500 MHz, DMSO-d_6): 8.16 (s, 1H, C(2)), 7.78 - 7.75 (m, 1H, CH_{Ar}), 7.52 - 7.50 (m, 1H, CH_{Ar}), 7.42 - 7.37 (m, 2H, CH_{Ar}), 5.31 (s, 2H, CH_2), ^{13}C NMR (126 MHz, DMSO-d_6): δ (ppm): 144.87, 138.03, 131.33, 129.55, 129.13, 129.01, 128.06, 125.78, 123.28, 48.85, MS (GC, 70 eV): *m/z* = 281 (M^+, 100%), 235 (20), 208 (58), 196 (10), 181 (23), 169 (54), 155 (17), 128 (18), 116 (17), 89 (50), 77 (11), 63 (21), HRMS (TOF MS ES+): calcd for $C_{10}H_8N_3O_2Se$ $(M + H)^+$ 281.9782, found 281.9785.

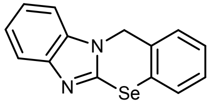

12H-benzimidazo[2,1-b][1,3]benzoselenazine (13c): colorless crystals, mp. 174-175 °C, ^{1}H NMR (500 MHz, $CDCl_3$): 7.70 (s, 1H, CH_{Ar}), 7.46 - 7.21 (m, 7H, CH_{Ar}), 5.09 (s, 2H, CH_2), ^{13}C NMR (126 MHz, $CDCl_3$): δ (ppm): 144.45, 143.24, 135.03, 131.96, 129.81, 128.82, 128.42, 127.47, 126.94, 122.32, 122.10, 118.81, 108.48, 47.92, MS (GC, 70 eV): *m/z* = 286 (M^+, 41%), 206 (100), 103 (10), 77 (12), HRMS (TOF MS ES+): calcd for $C_{14}H_{11}N_2Se$ $(M + H)^+$ 287.0087, found 287.0092.

References

1 V. O. Iaroshenko, A. Gevorgyan, O. Davydova, A. Villinger and P. Langer, *J. Org. Chem.*, 2014, **79**, 2906–2915.
2 Y. P. Ustimenko, A. M. Agafontsev, V. Y. Komarov and A. V. Tkachev, *Mendeleev Commun.*, 2018, **28**, 584–586.
3 V. O. Iaroshenko, A. Gevorgyan, S. Mkrtchyan, K. Arakelyan, T. Grigoryan, J. Yedoyan, A. Villinger and P. Langer, *J. Org. Chem.*, 2015, **80**, 2103–2119.
4 E. Apohan, U. Yilmaz, O. Yilmaz, A. Serindag, H. Küçükbay, O. Yesilada and Y. Baran, *J. Organomet. Chem.*, 2017, **828**, 52–58.
5 C. Ding, Y. Yu, Q. Yu, Z. Xie, Y. Zhou, J. Zhou, G. Liang and Z. Song, *ChemCatChem*, 2018, **10**, 5397–5401.
6 C. P. Owen, I. Shahid, M. S. Olusanjo, C. H. Patel, S. Dhanani and S. Ahmed, *J. Steroid Biochem. Mol. Biol.*, 2008, **111**, 117–127.
7 A. M. Linsenmeier, C. M. Williams and S. Bräse, *J. Org. Chem.*, 2011, **76**, 9127–9132.

(C) Copies ^{1}H and ^{13}C NMR spectra.

Compound **sm3**

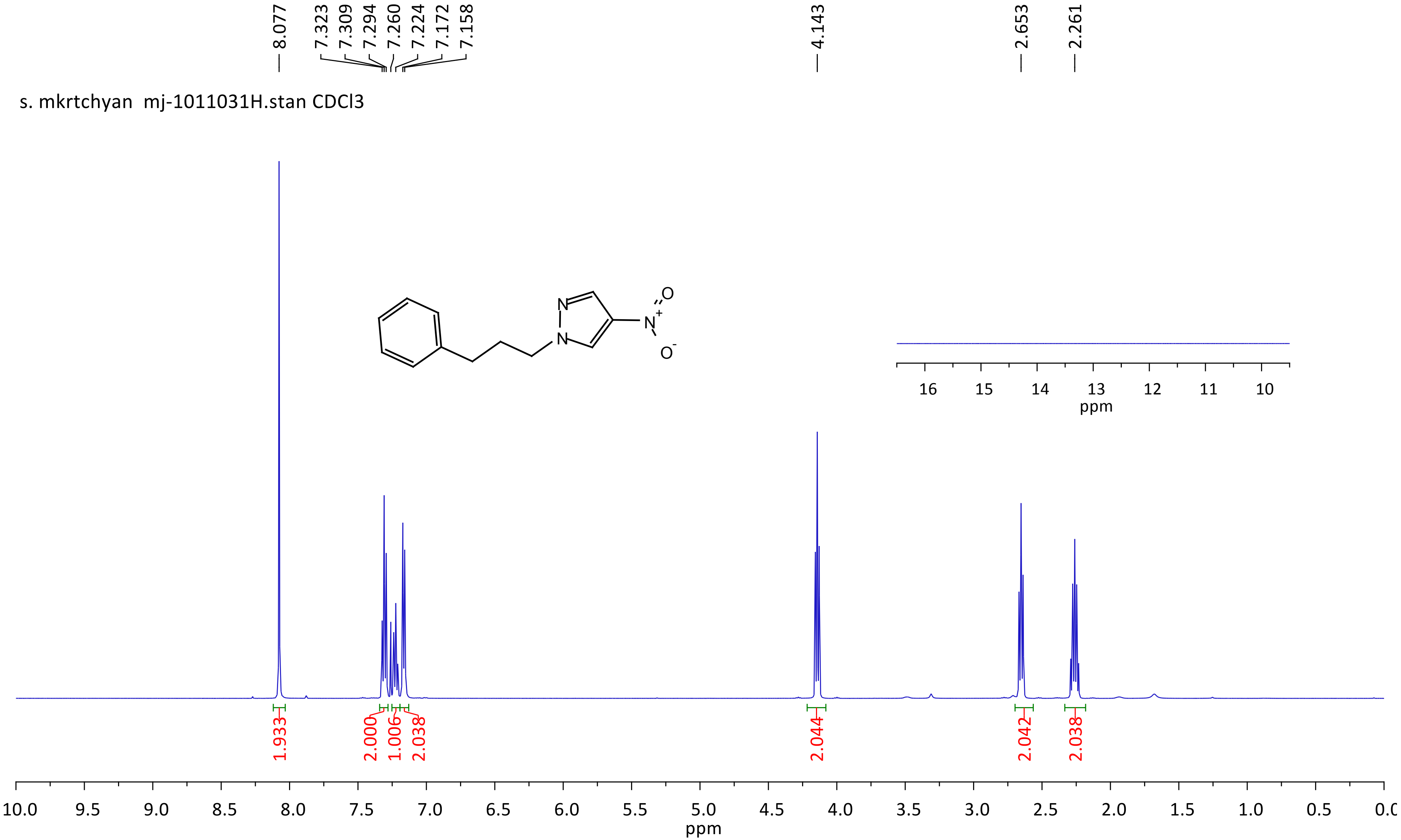

Comment	s. mkrtchyan mj-1011031H.stan CDCl3
Number of Scans	24
Spectrometer Frequency	500.13
Spectral Width	12335.5
Spectral Size	65536

Compound **sm3**

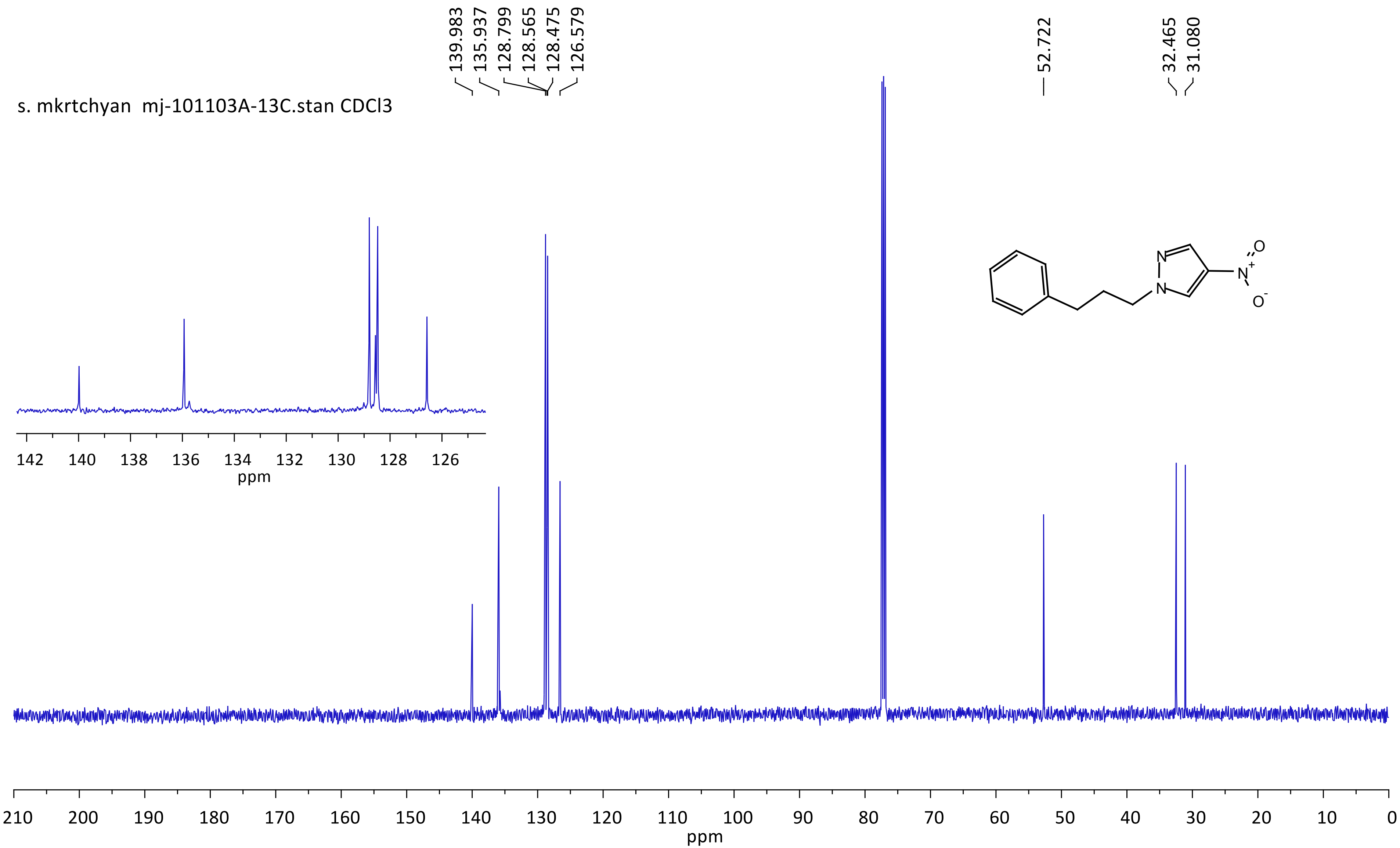

Comment	s. mkrtchyan mj-101103A-13C.stan CDCl3
Number of Scans	512
Spectrometer Frequency	125.76
Spectral Width	36057.7
Spectral Size	65536

Compound **sm6**

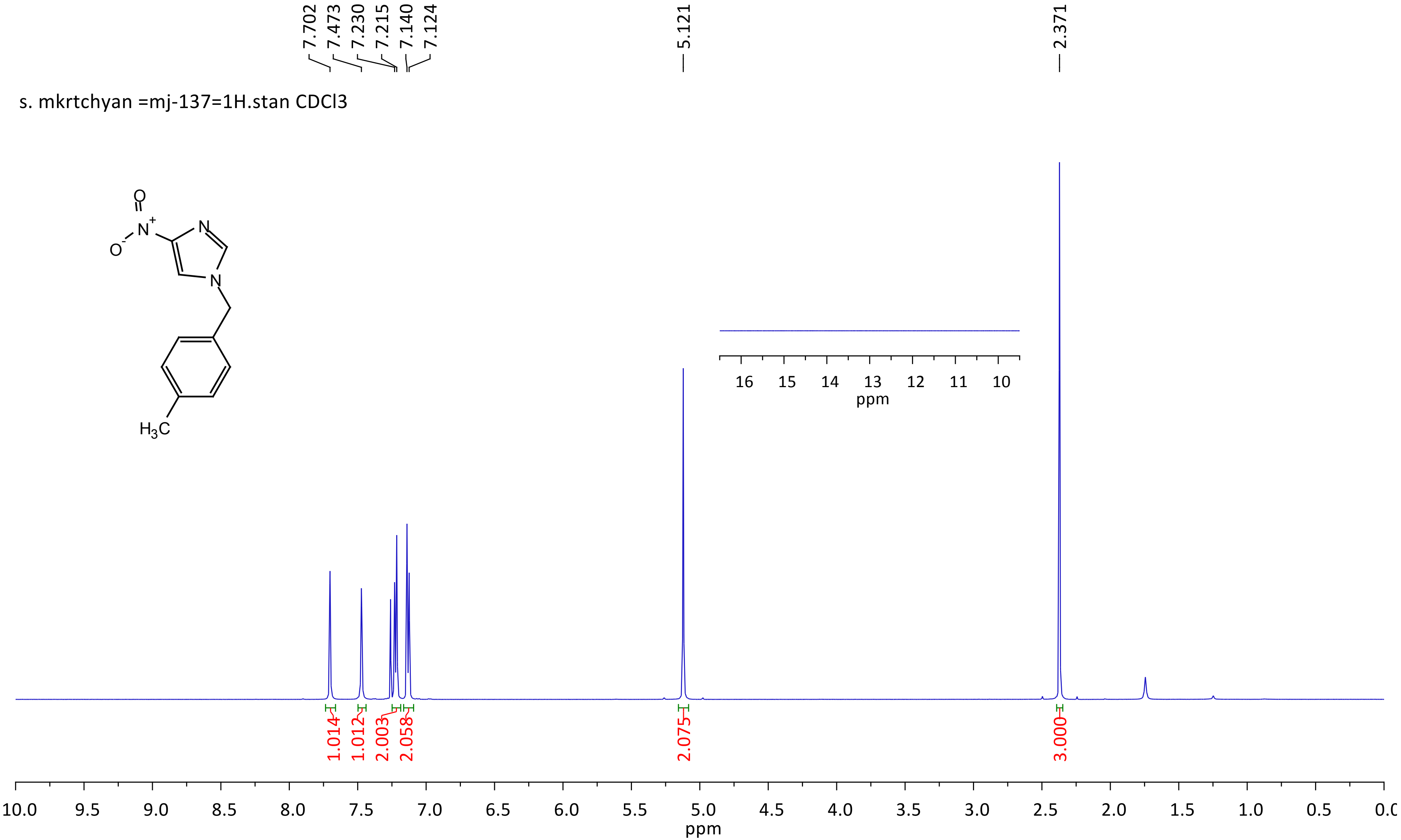

Comment	s. mkrtchyan =mj-137=1H.stan CDCl3
Number of Scans	24
Spectrometer Frequency	500.13
Spectral Width	12335.5
Spectral Size	65536

Compound **sm6**

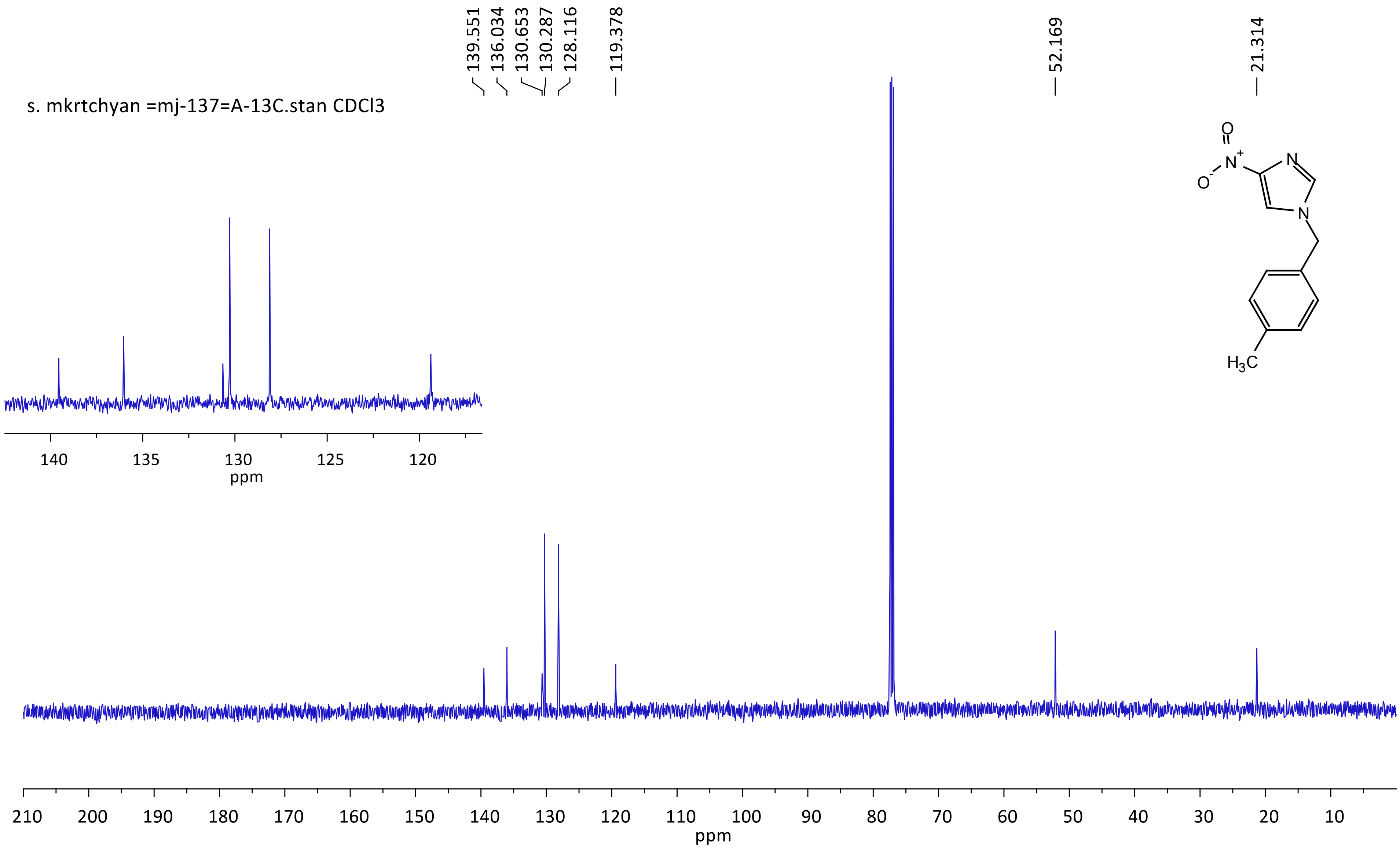

Comment	s. mkrtchyan =mj-137=A-13C.stan CDCl3
Number of Scans	256
Spectrometer Frequency	125.76
Spectral Width	36057.7
Spectral Size	65536

Compound **sm8**

s. mkrtchyan =mj-136-516=1H.stan CDCl3

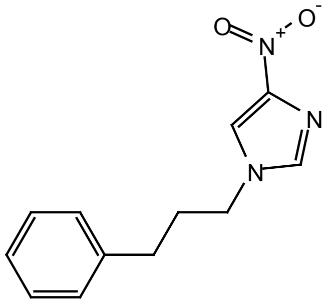

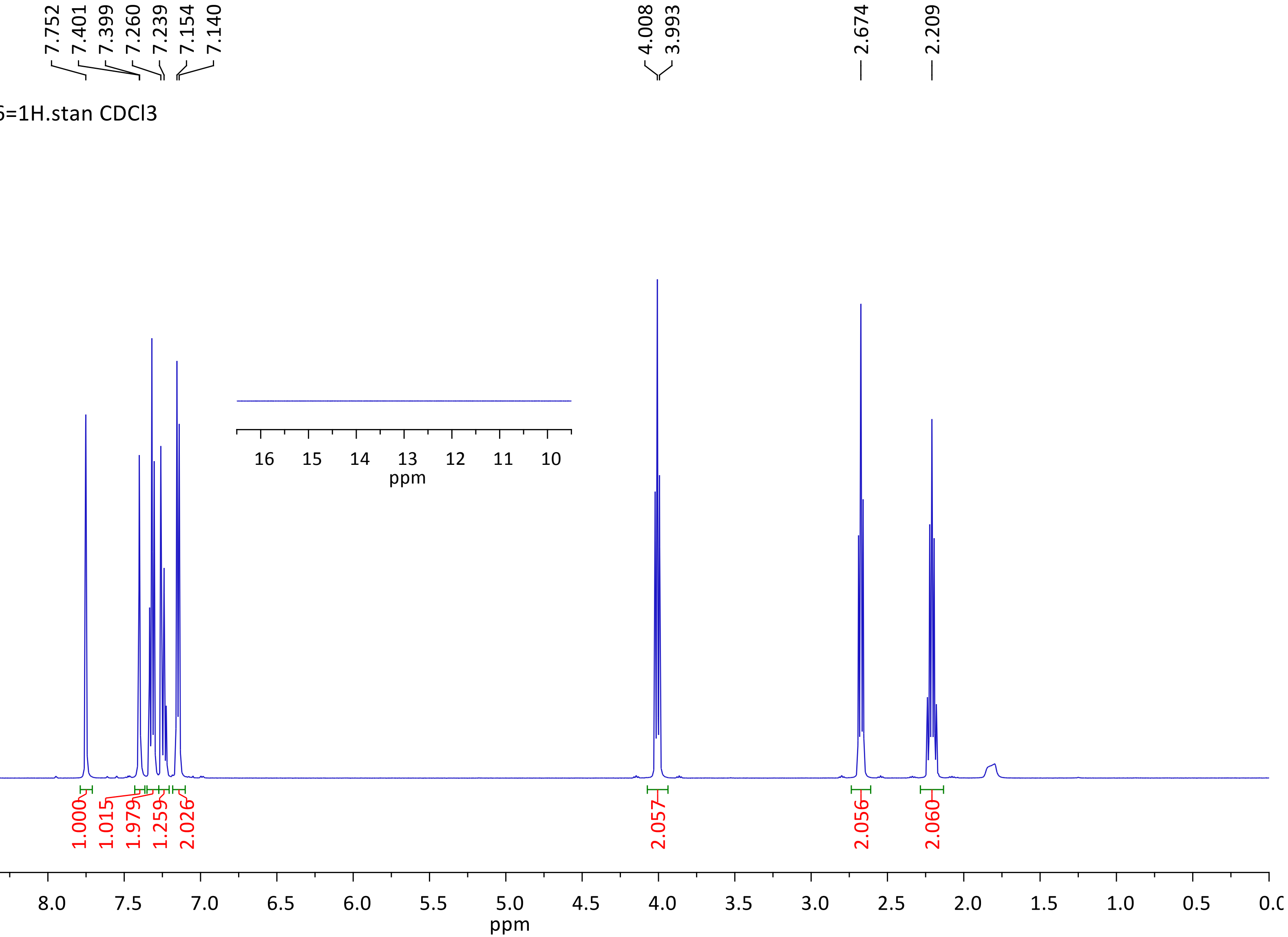

Comment	s. mkrtchyan =mj-136-516=1H.stan CDCl3
Number of Scans	24
Spectrometer Frequency	500.13
Spectral Width	12335.5
Spectral Size	65536

Compound **sm8**

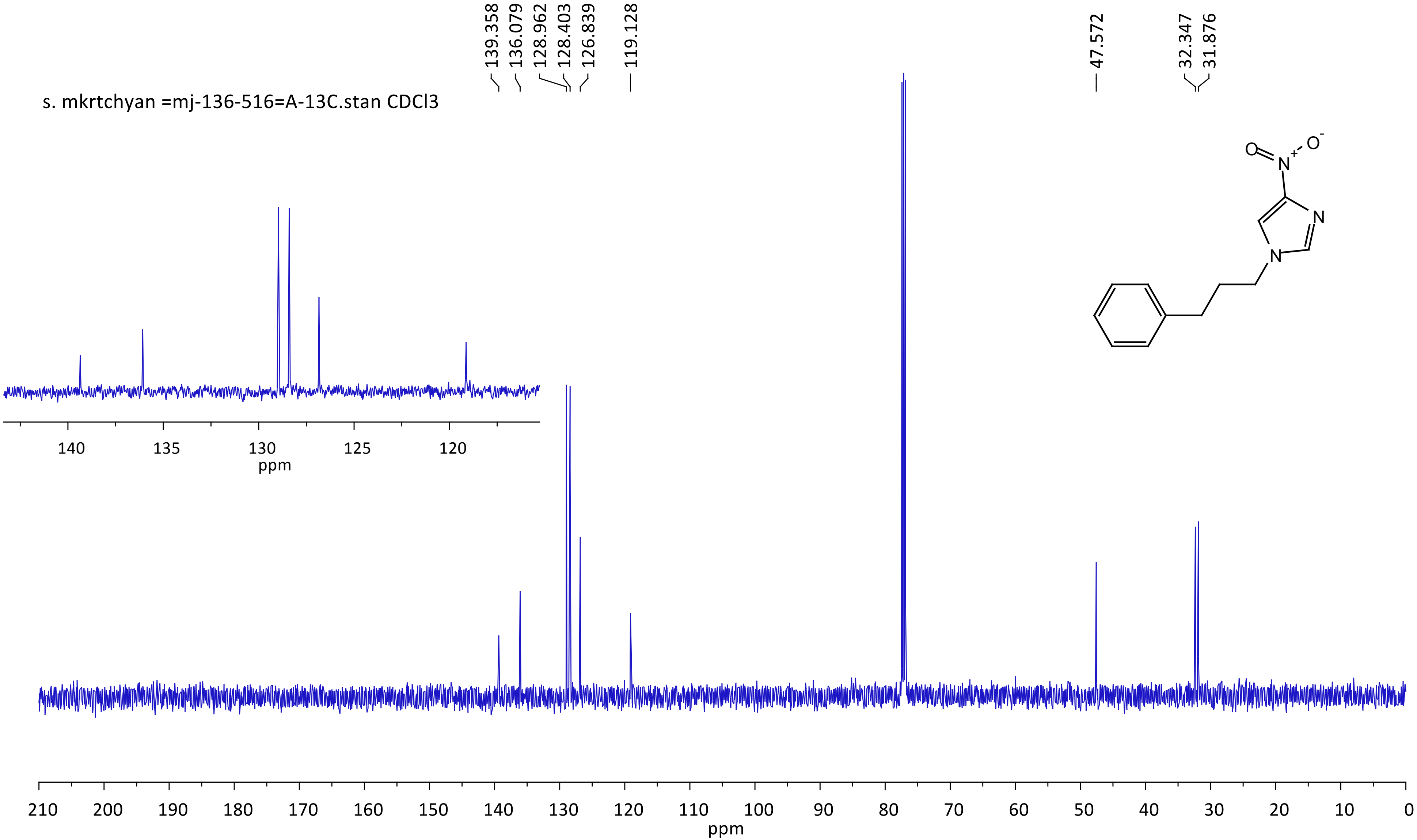

Comment	s. mkrtchyan =mj-136-516=A-13C.stan CDCl3
Number of Scans	128
Spectrometer Frequency	125.76
Spectral Width	36057.7
Spectral Size	65536

Compound **sm13**

s. mkrtychyan =mj-123-crs=1H.stan CDCl3

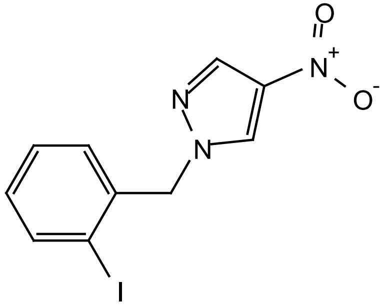

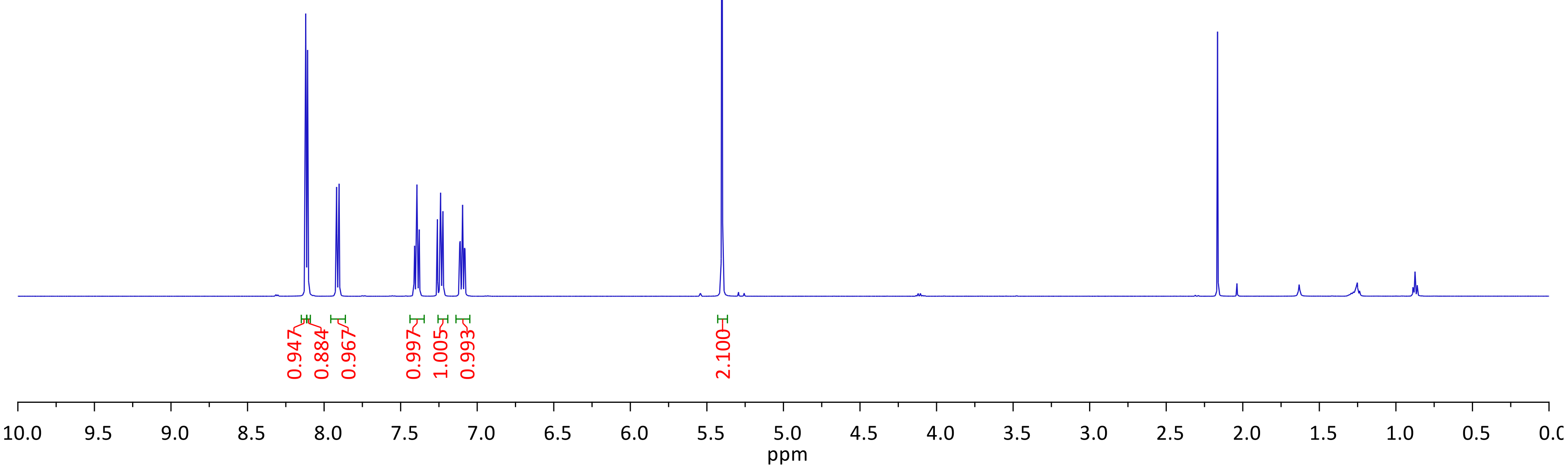

Comment	s. mkrtychyan =mj-123-crs=1H.stan CDCl3
Number of Scans	24
Spectrometer Frequency	500.13
Spectral Width	12335.5
Spectral Size	65536

Compound **sm13**

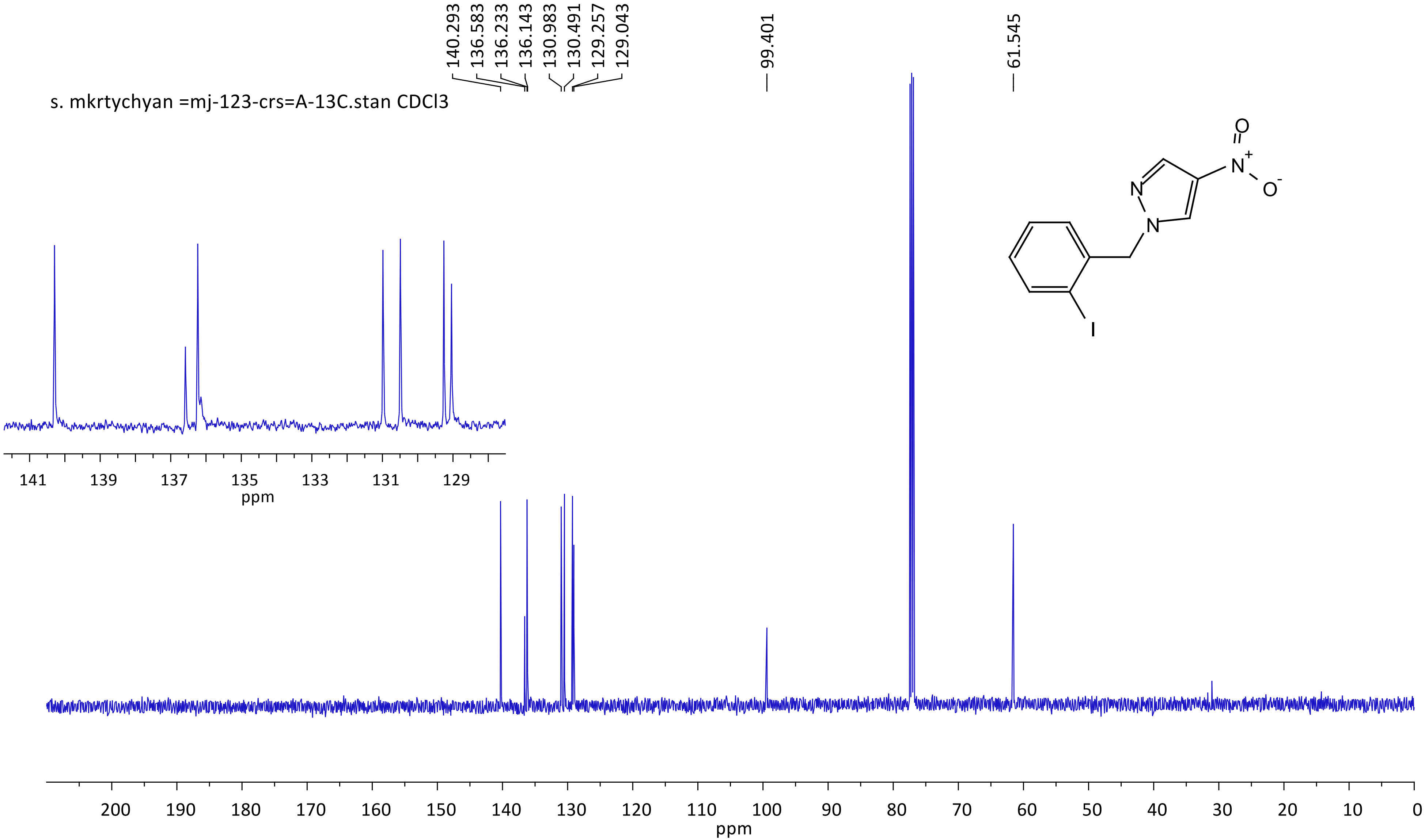

Comment	s. mkrtychyan =mj-123-crs=A-13C.stan CDCl3
Number of Scans	224
Spectrometer Frequency	125.76
Spectral Width	36057.7
Spectral Size	65536

Compound **sm14**

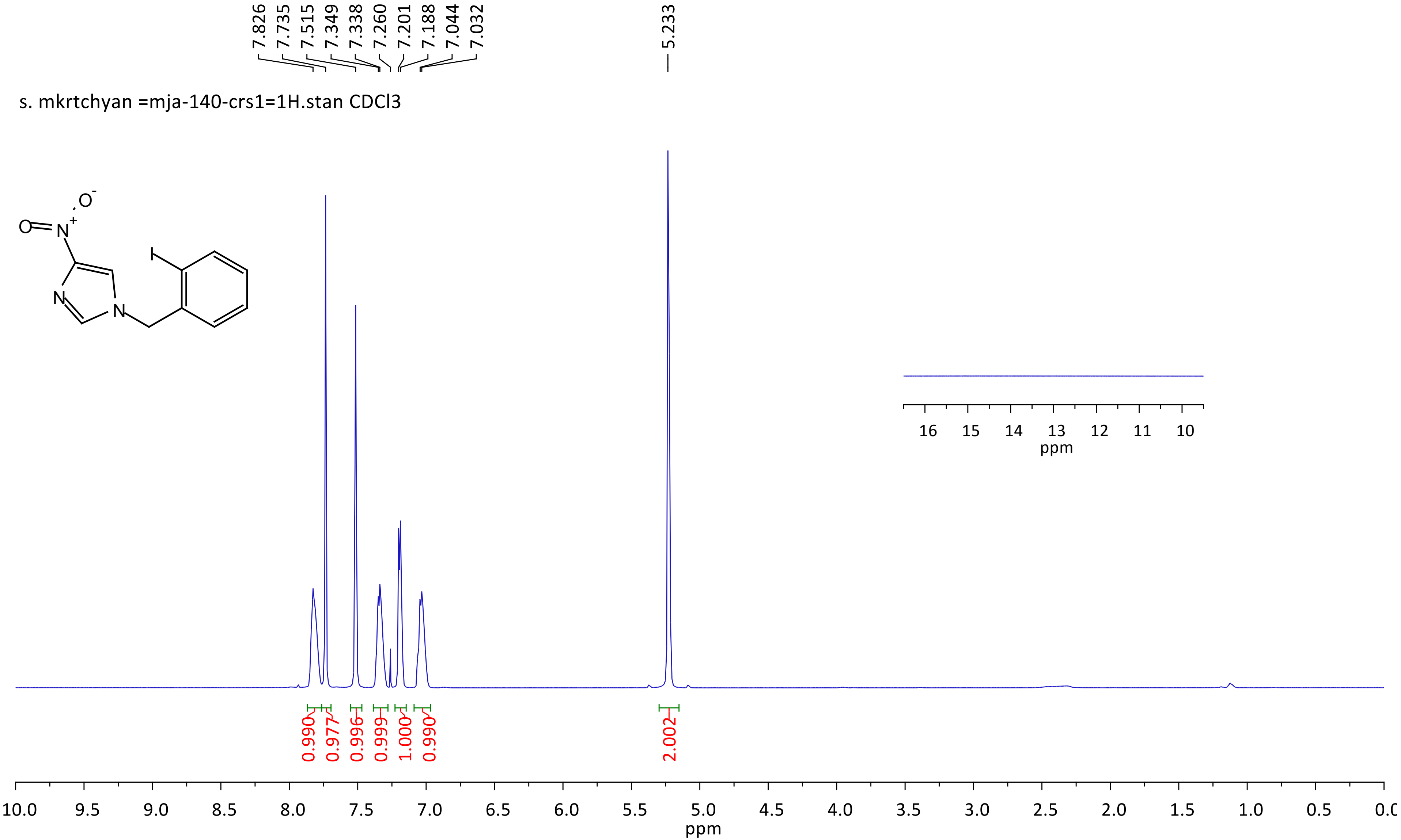

Comment	s. mkrtchyan =mja-140-crs1=1H.stan CDCl3
Number of Scans	24
Spectrometer Frequency	500.13
Spectral Width	12335.5
Spectral Size	65536

Compound **sm14**

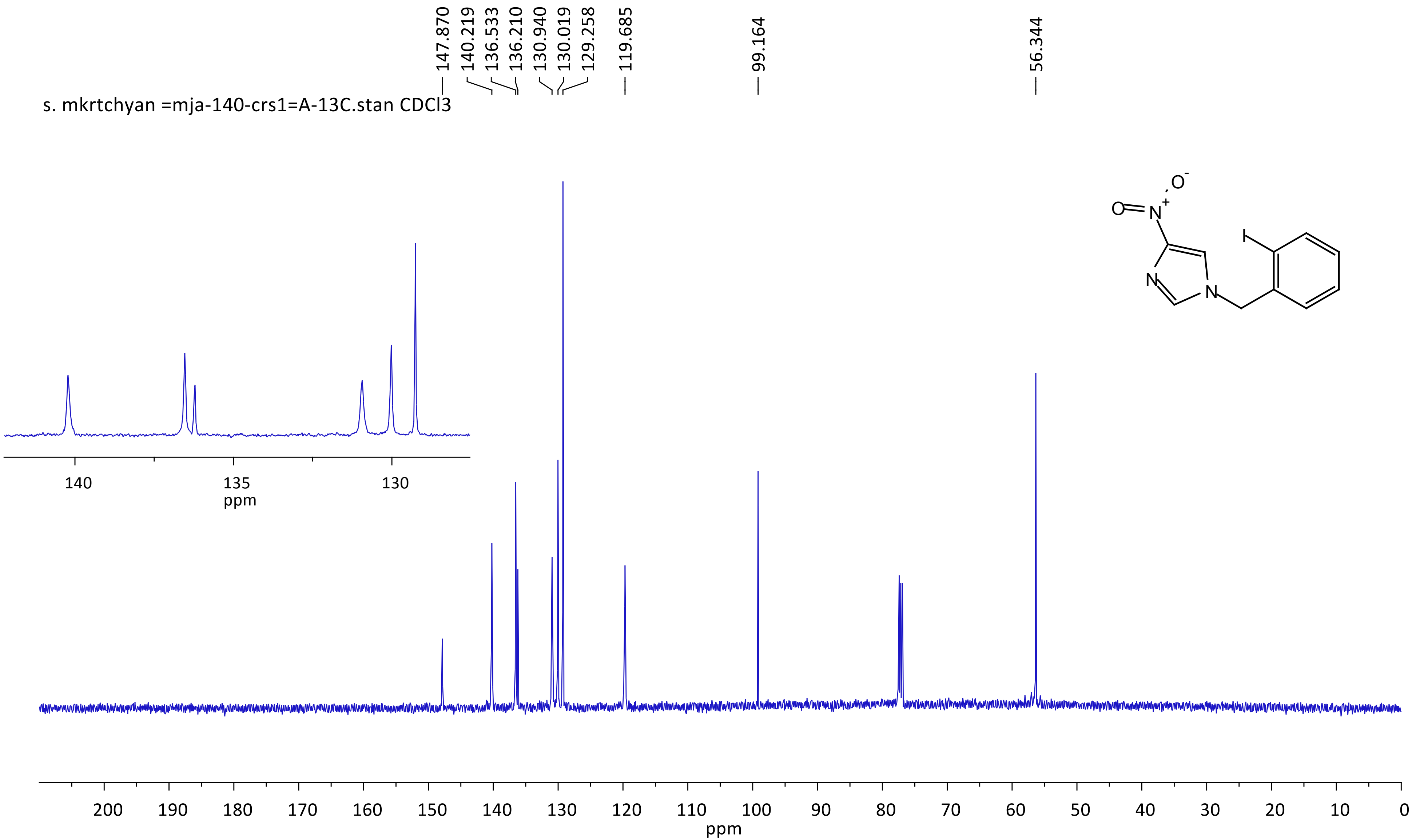

Comment	s. mkrtchyan =mja-140-crs1=A-13C.stan CDCl3
Number of Scans	512
Spectrometer Frequency	125.76
Spectral Width	36057.7
Spectral Size	65536

Compound **1a**

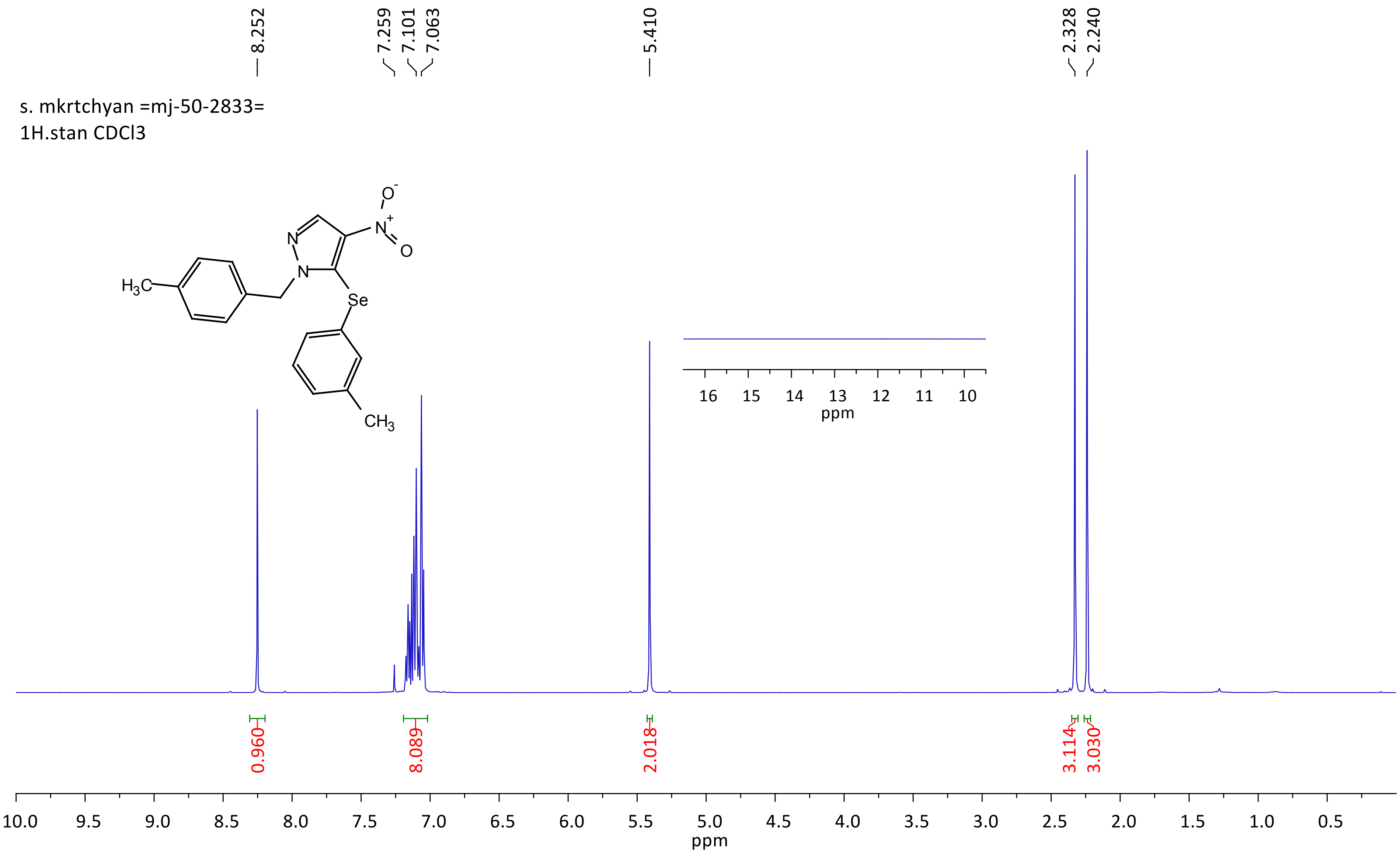

Comment	s. mkrtchyan =mj-50-2833=1H.stan CDCl3
Number of Scans	24
Spectrometer Frequency	500.13
Spectral Width	12335.5
Spectral Size	65536

Compound **1a**

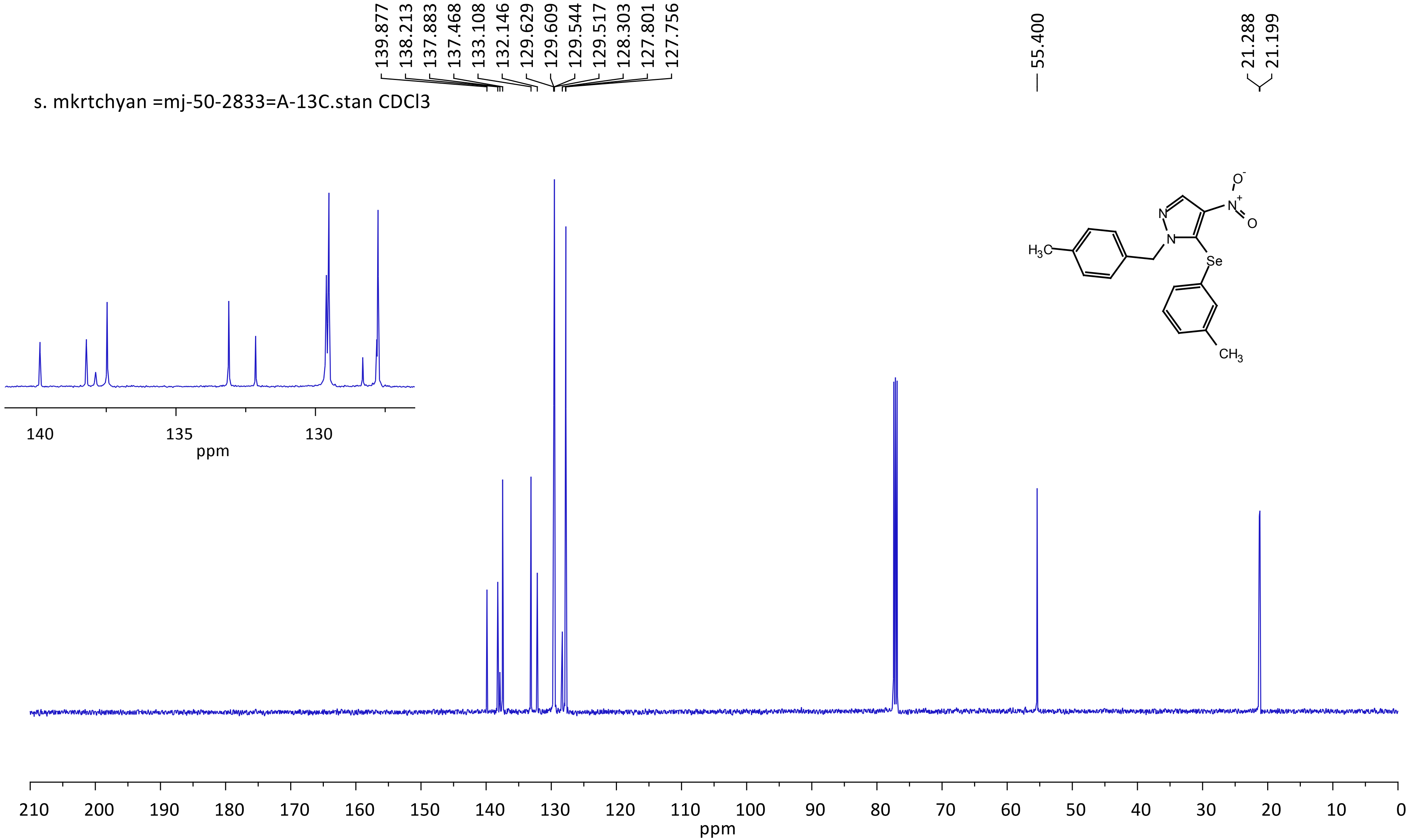

Comment	s. mkrtchyan =mj-50-2833=A-13C.stan CDCl3
Number of Scans	512
Spectrometer Frequency	125.76
Spectral Width	36057.7
Spectral Size	65536

Compound **1b**

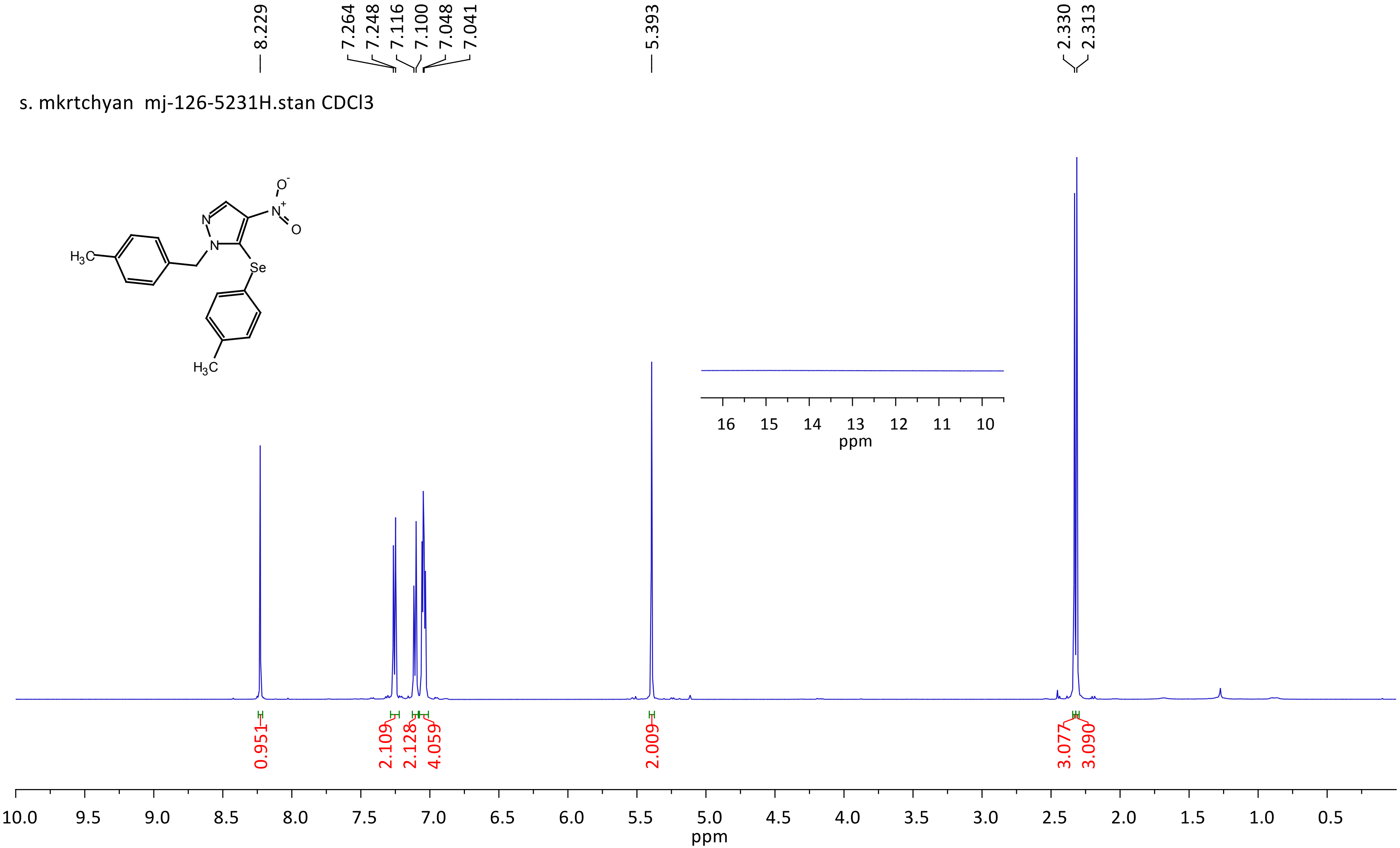

Comment	s. mkrtchyan mj-126-5231H.stan CDCl3
Number of Scans	24
Spectrometer Frequency	500.13
Spectral Width	12335.5
Spectral Size	65536

Compound **1b**

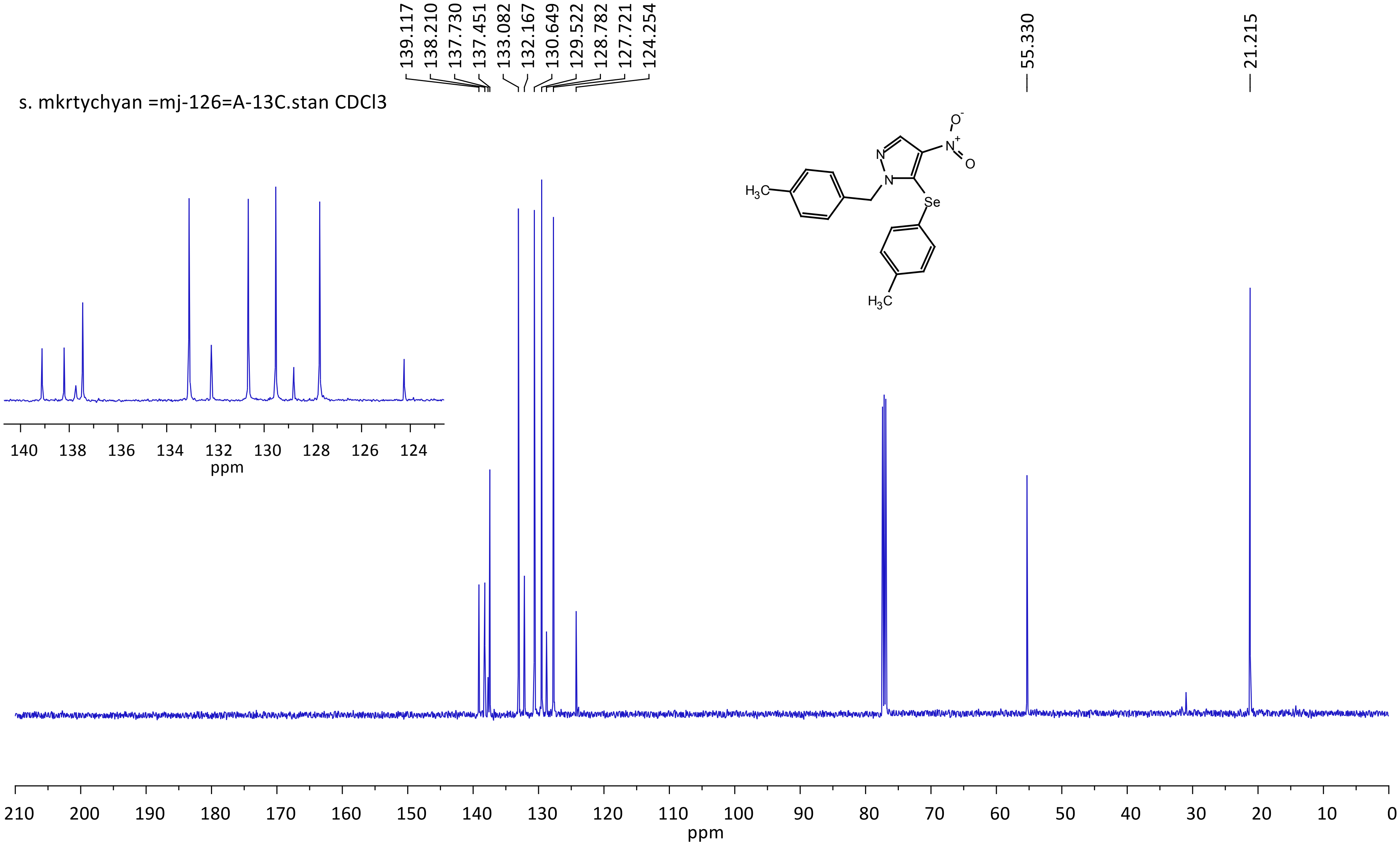

Comment	s. mkrtychyan =mj-126=A-13C.stan CDCl3
Number of Scans	512
Spectrometer Frequency	125.76
Spectral Width	36057.7
Spectral Size	65536

Compound **1c**

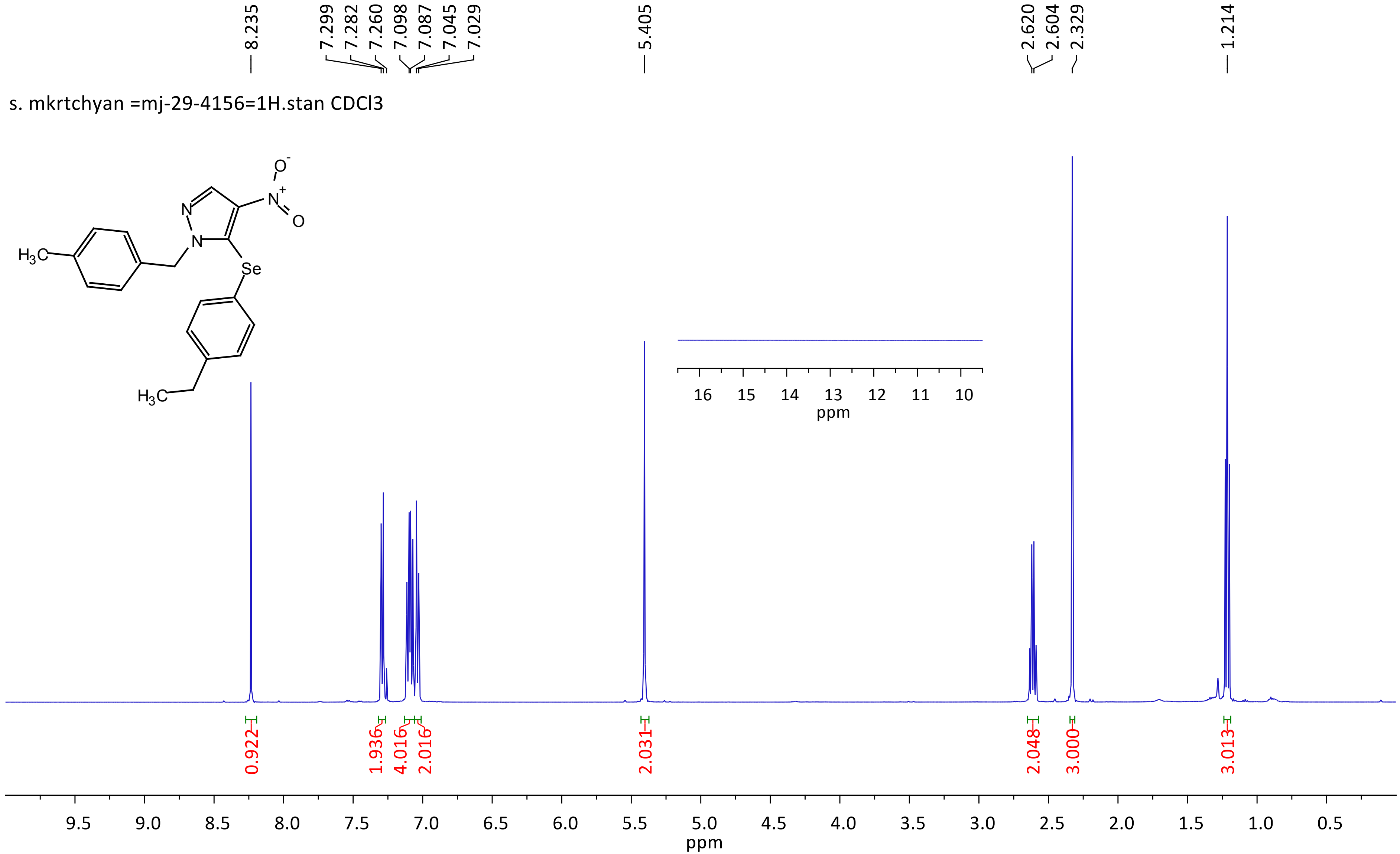

Comment	S.mkrtchyan =MJ-29-KI-F4156=1H.stan CDCl3
Number of Scans	16
Spectrometer Frequency	500.13
Spectral Width	12335.5
Spectral Size	65536

Compound **1c**

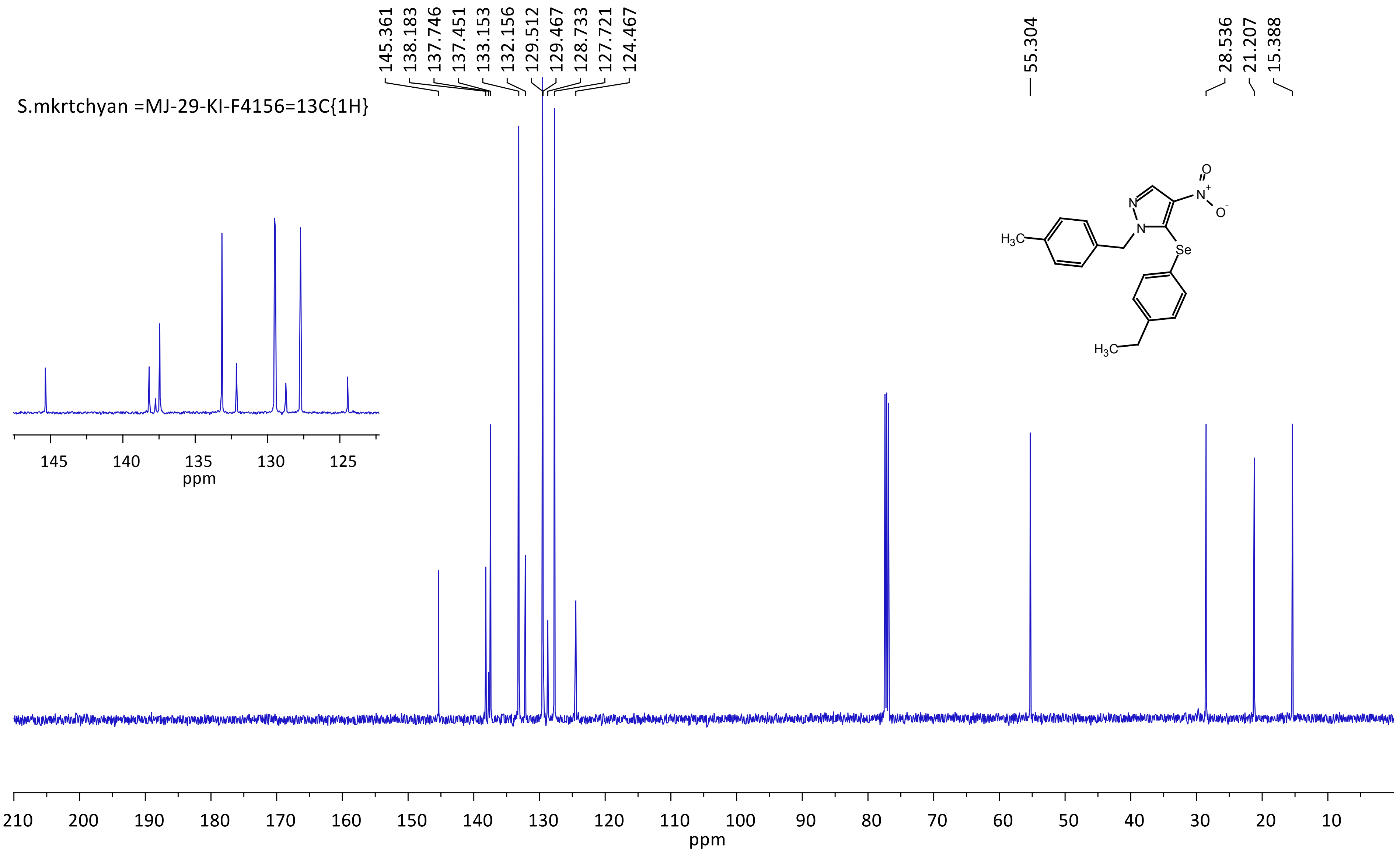

Comment	S.mkrtchyan =MJ-29-KI-F4156=13C{1H}
Number of Scans	256
Spectrometer Frequency	125.76
Spectral Width	36057.7
Spectral Size	65536

Compound **1d**

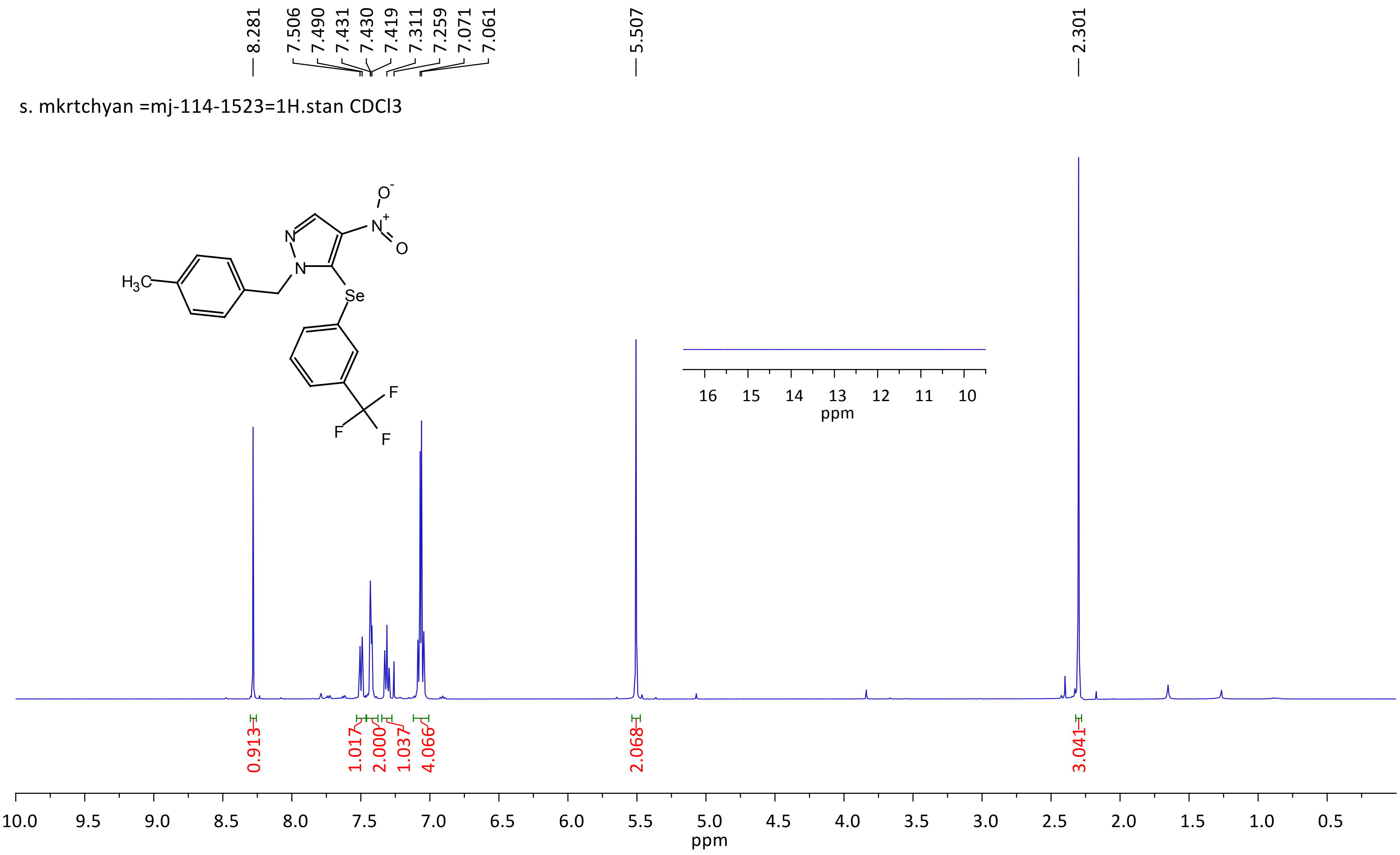

Comment	s. mkrtchyan =mj-114-1523=1H.stan CDCl3
Number of Scans	32
Spectrometer Frequency	500.13
Spectral Width	12335.5
Spectral Size	65536

Compound **1d**

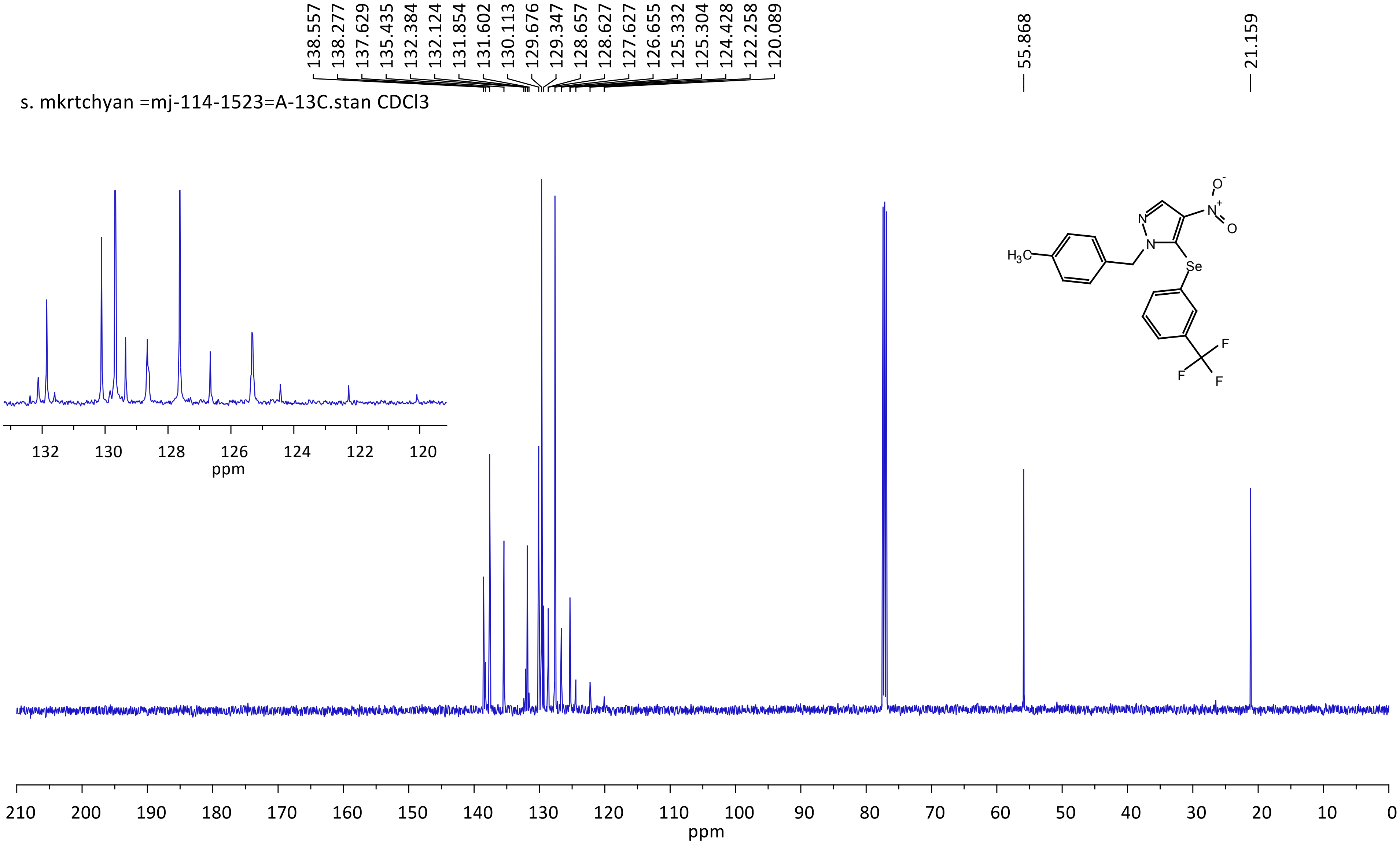

Comment	s. mkrtchyan =mj-114-1523=A-13C.stan CDCl3
Number of Scans	336
Spectrometer Frequency	125.76
Spectral Width	36057.7
Spectral Size	65536

Compound **1e**

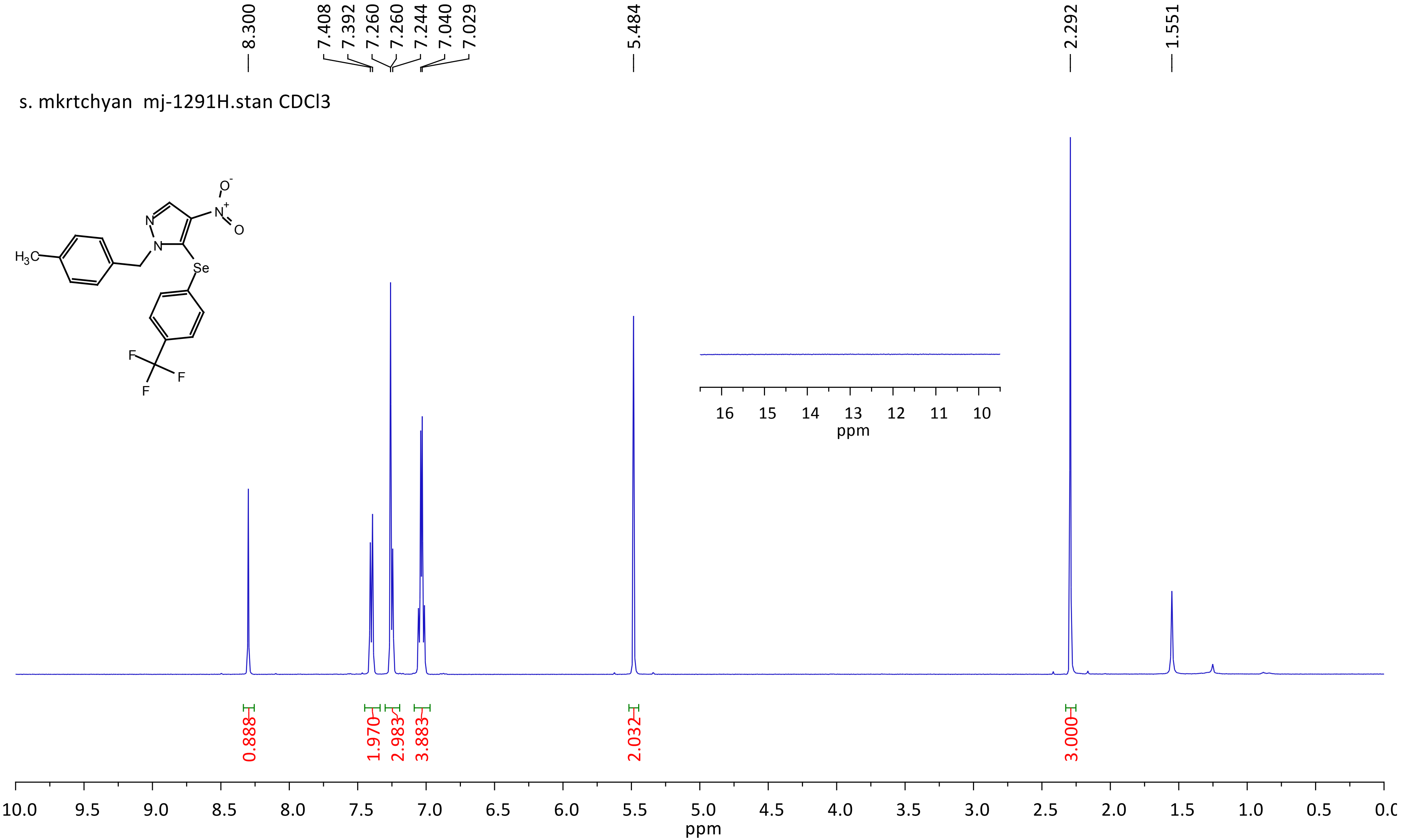

Comment	s. mkrtchyan mj-1291H.stan CDCl3
Number of Scans	24
Spectrometer Frequency	500.13
Spectral Width	12335.5
Spectral Size	65536

Compound **1e**

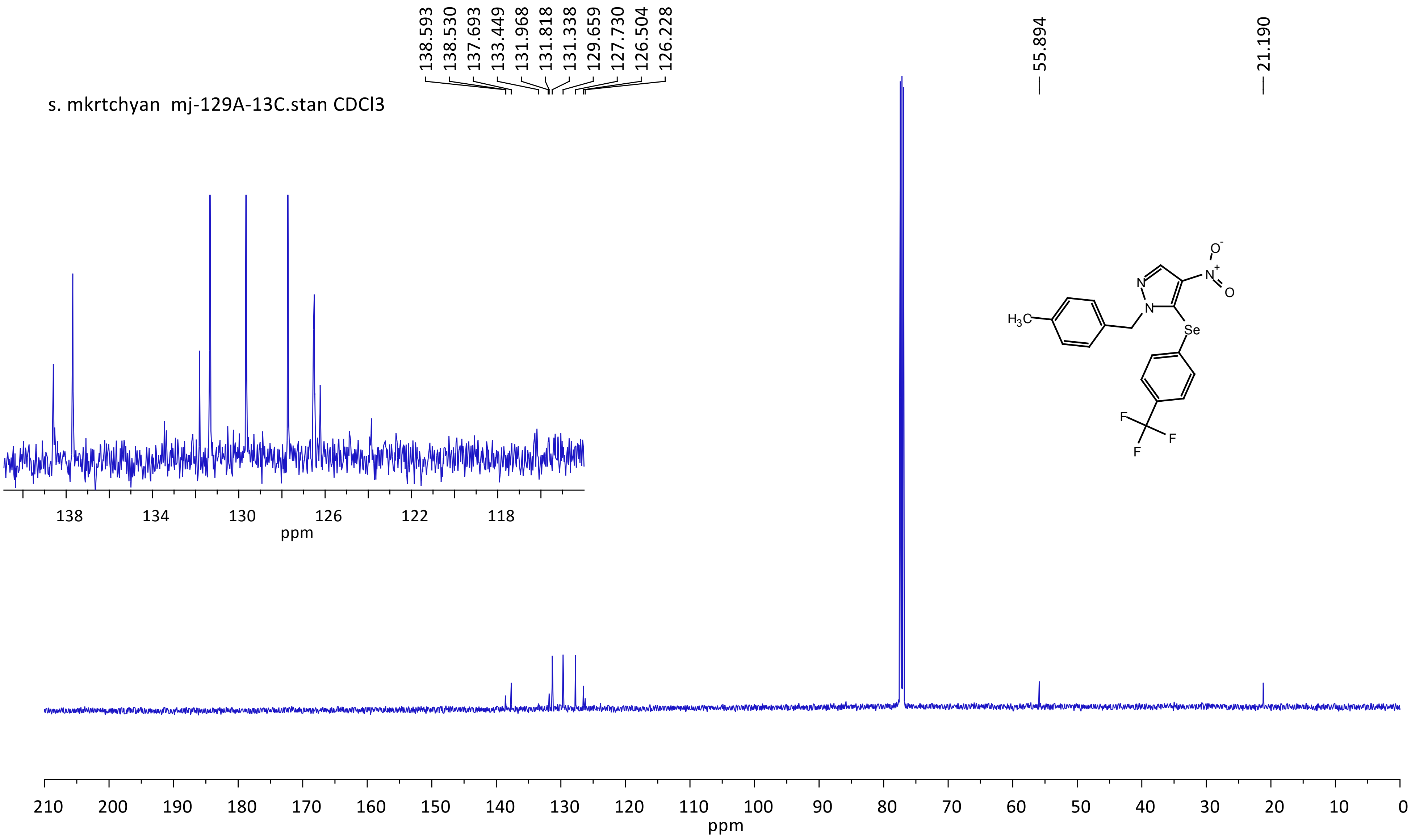

Comment	s. mkrtchyan mj-129A-13C.stan CDCl3
Number of Scans	2048
Spectrometer Frequency	125.76
Spectral Width	36057.7
Spectral Size	65536

Compound **1f**

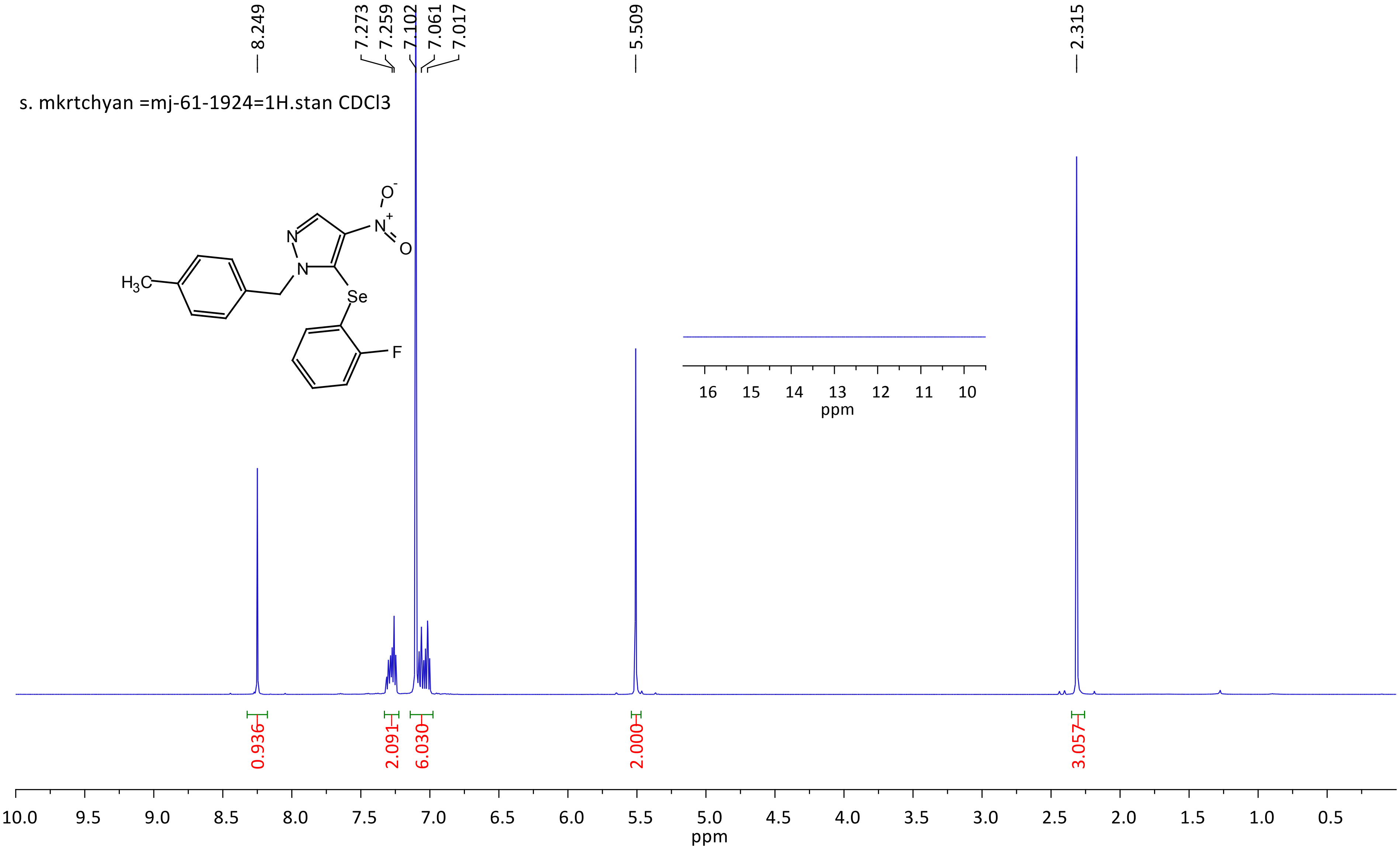

Comment	s. mkrtchyan =mj-61-1924=1H.stan CDCl3
Number of Scans	24
Spectrometer Frequency	500.13
Spectral Width	12335.5
Spectral Size	65536

Compound **1f**

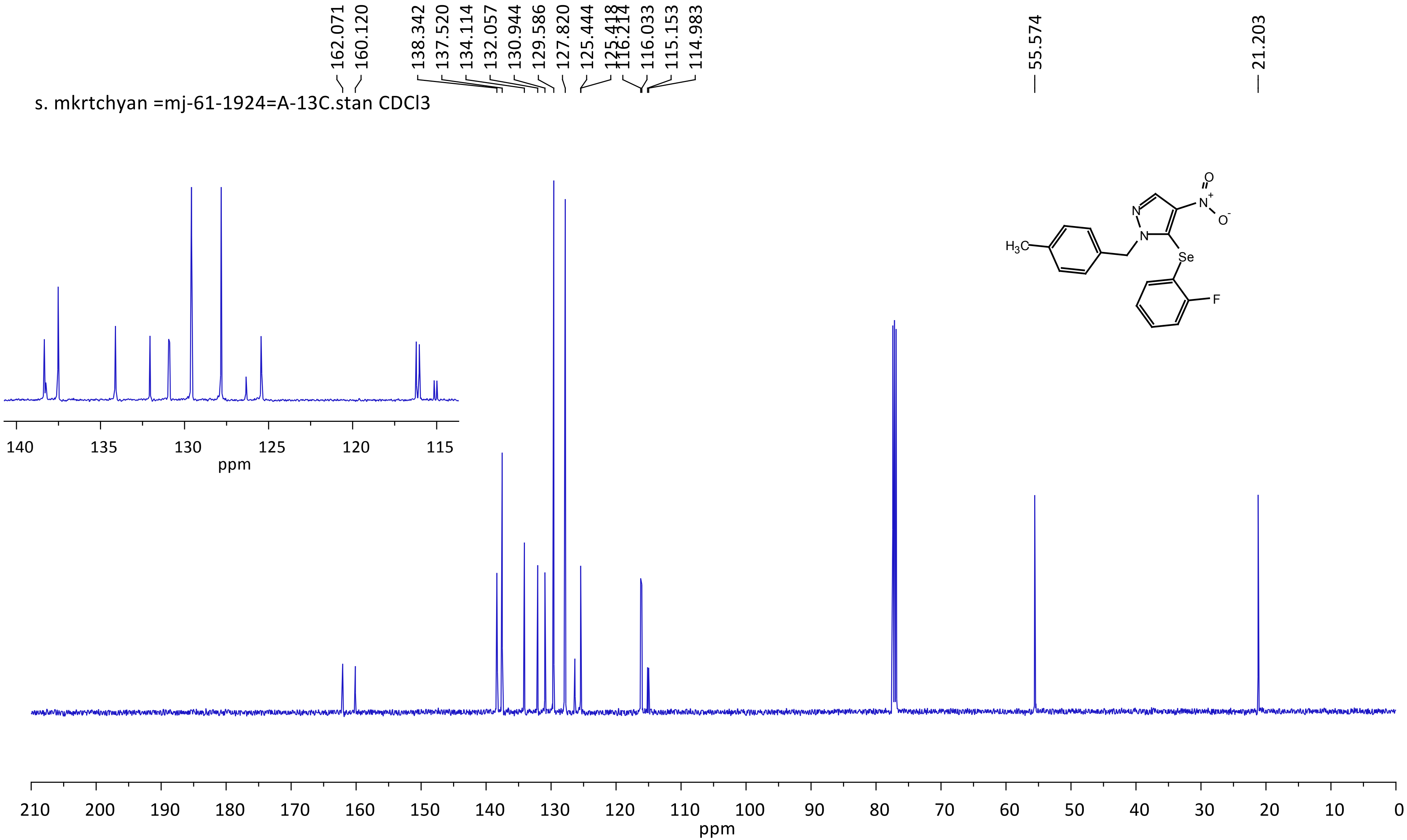

Comment	s. mkrtchyan =mj-61-1924=A-13C.stan CDCl3
Number of Scans	512
Spectrometer Frequency	125.76
Spectral Width	36057.7
Spectral Size	65536

Compound **1g**

s. mkrtchyan =mj-125-1116=1H.stan CDCl3

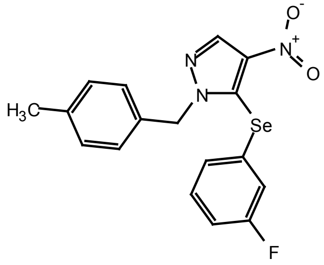

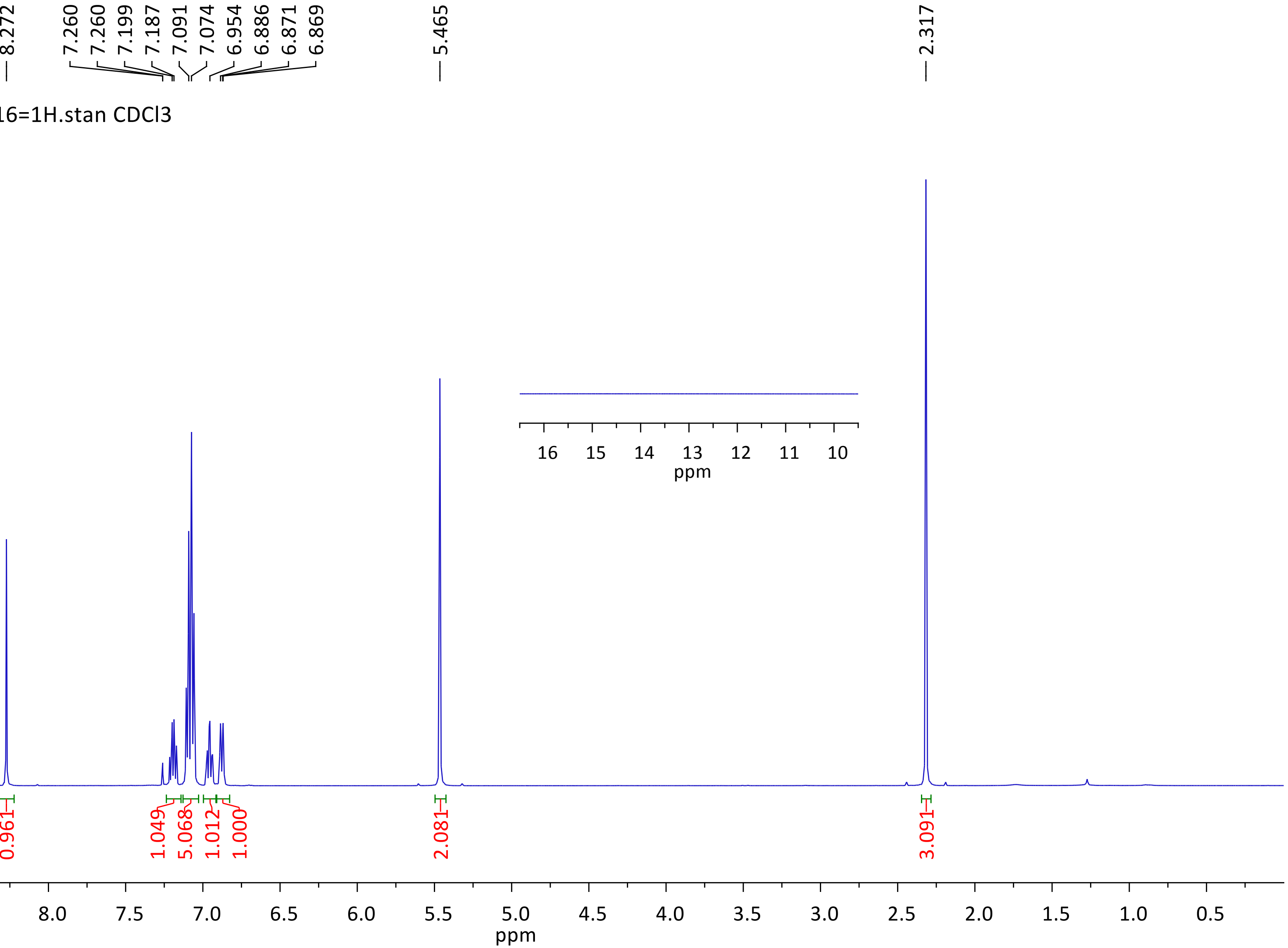

Comment	s. mkrtchyan =mj-125-1116=1H.stan CDCl3
Number of Scans	24
Spectrometer Frequency	500.13
Spectral Width	12335.5
Spectral Size	65536

Compound **1g**

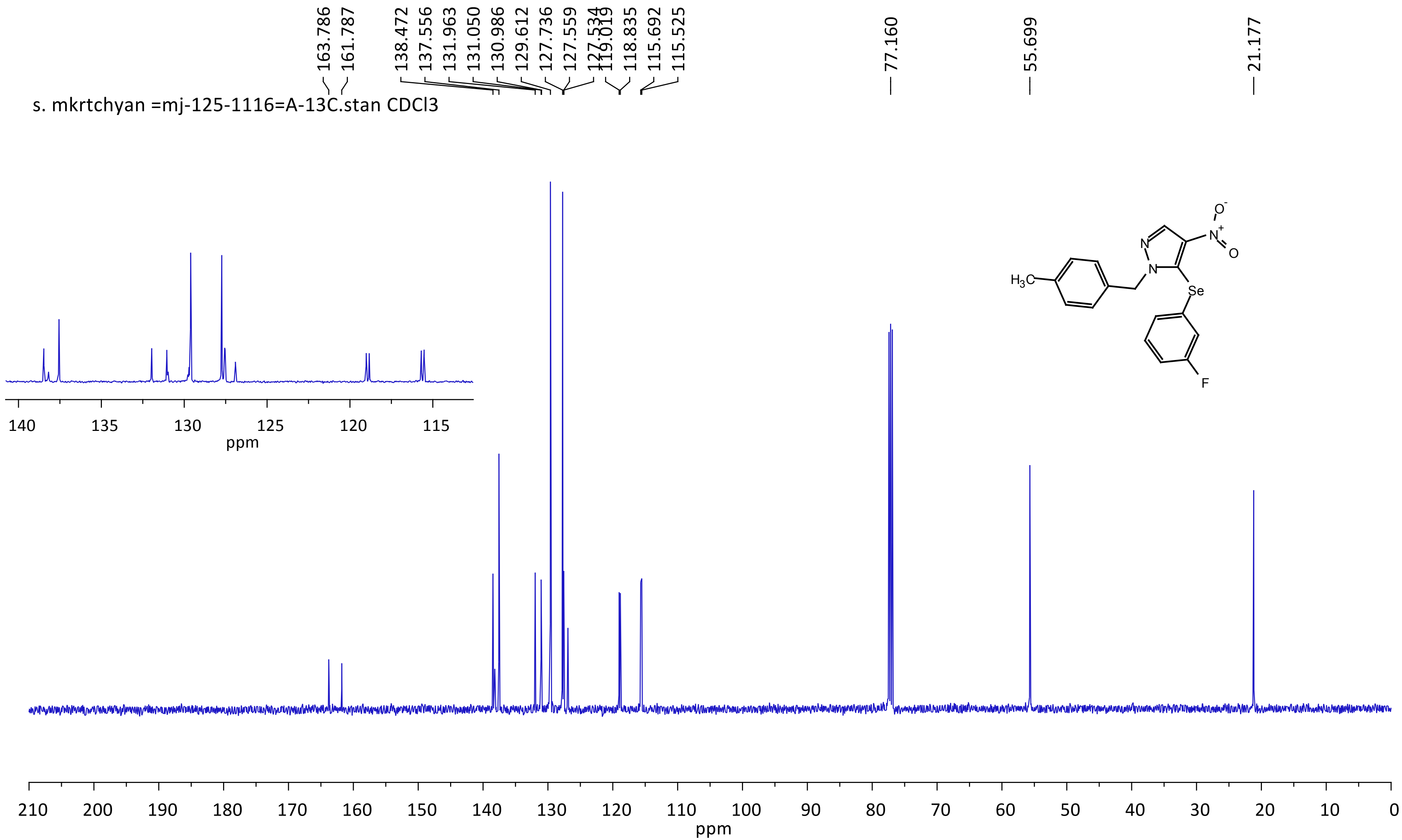

Comment	s. mkrtchyan =mj-125-1116=A-13C.stan CDCl3
Number of Scans	256
Spectrometer Frequency	125.76
Spectral Width	36057.7
Spectral Size	65536

Compound **1h**

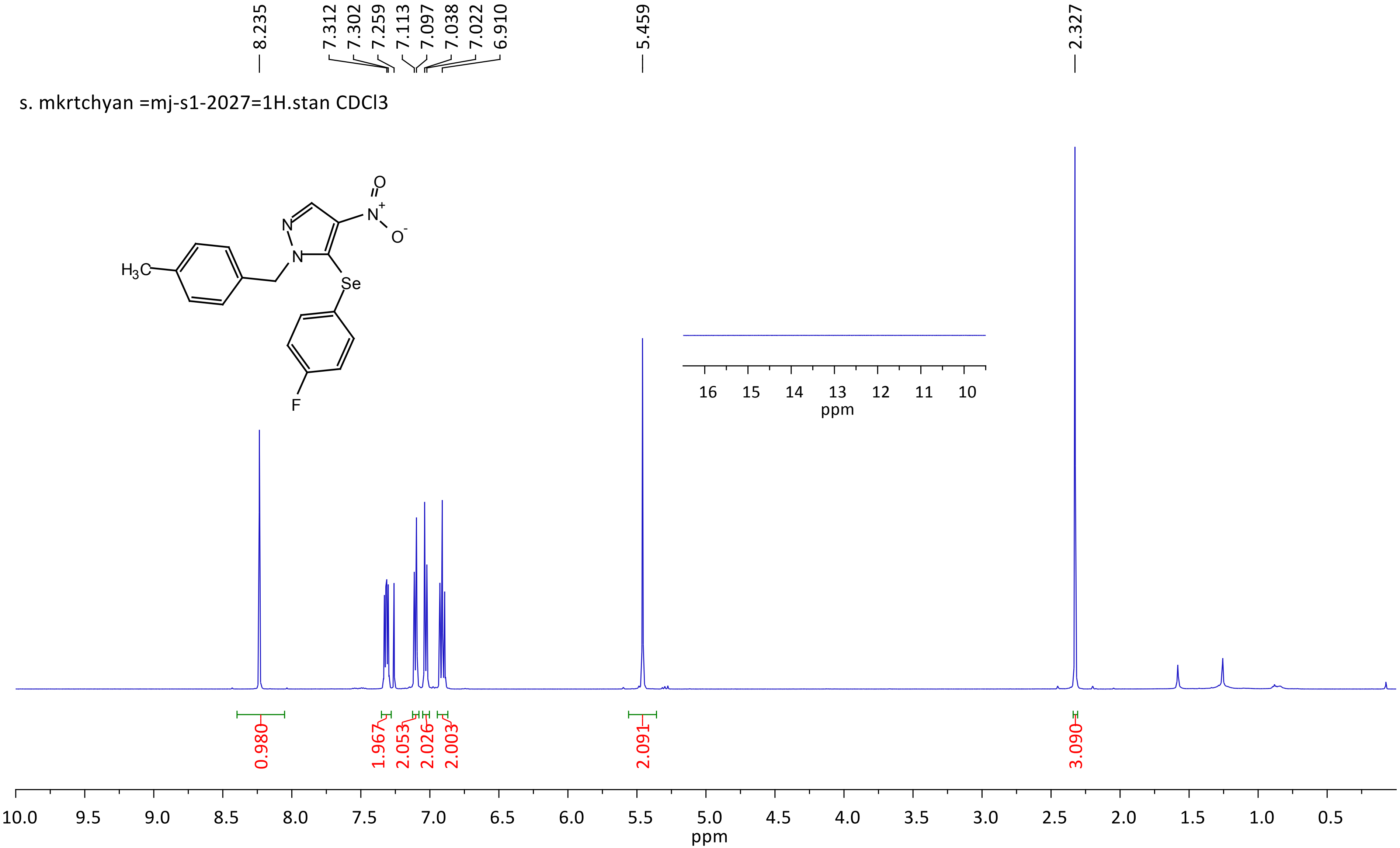

Comment	s. mkrtchyan =mj-s1-2027=1H.stan CDCl3
Number of Scans	24
Spectrometer Frequency	500.13
Spectral Width	12335.5
Spectral Size	65536

Compound **1h**

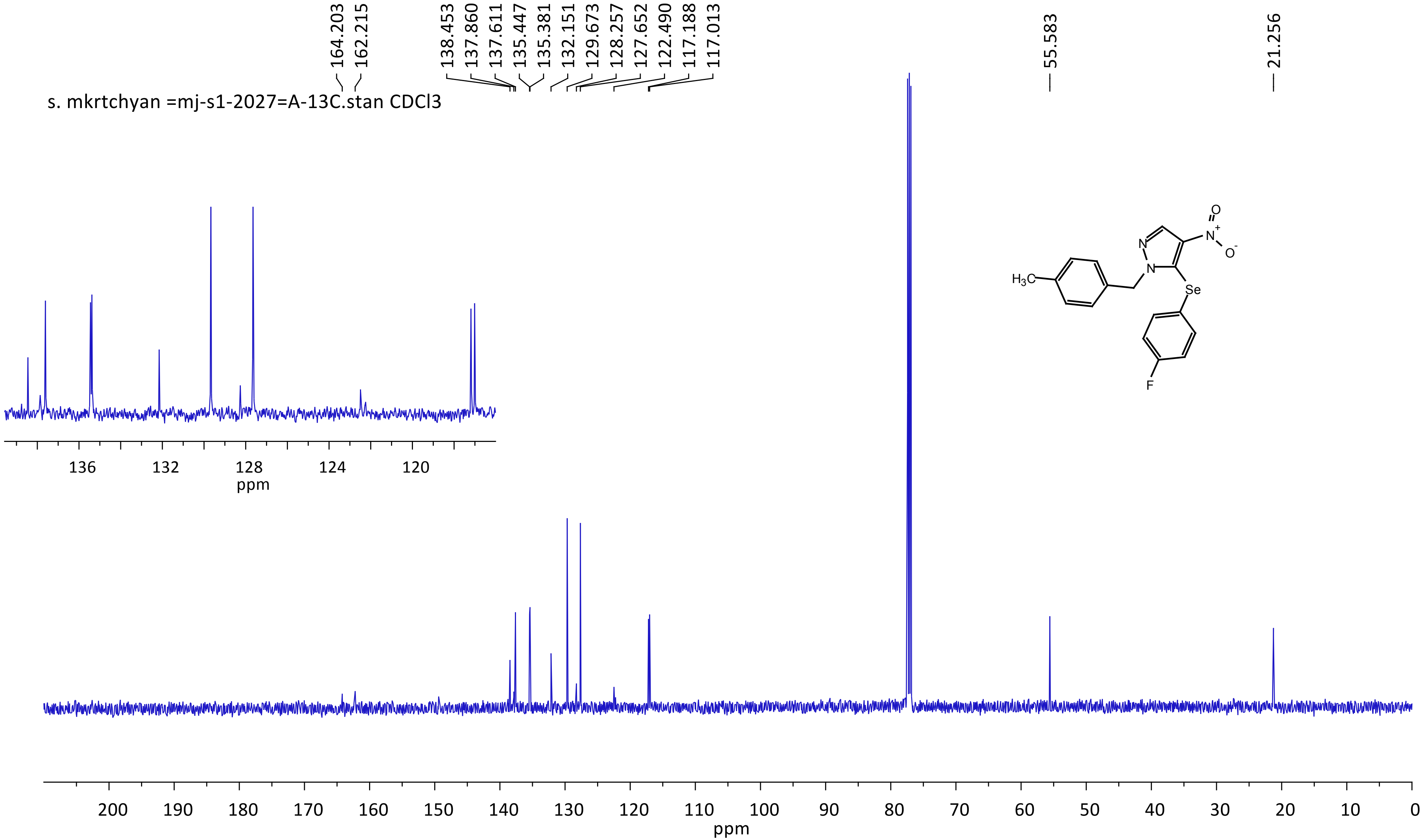

Comment	s. mkrtchyan =mj-s1-2027=A-13C.stan CDCl3
Number of Scans	256
Spectrometer Frequency	125.76
Spectral Width	36057.7
Spectral Size	65536

Compound **1i**

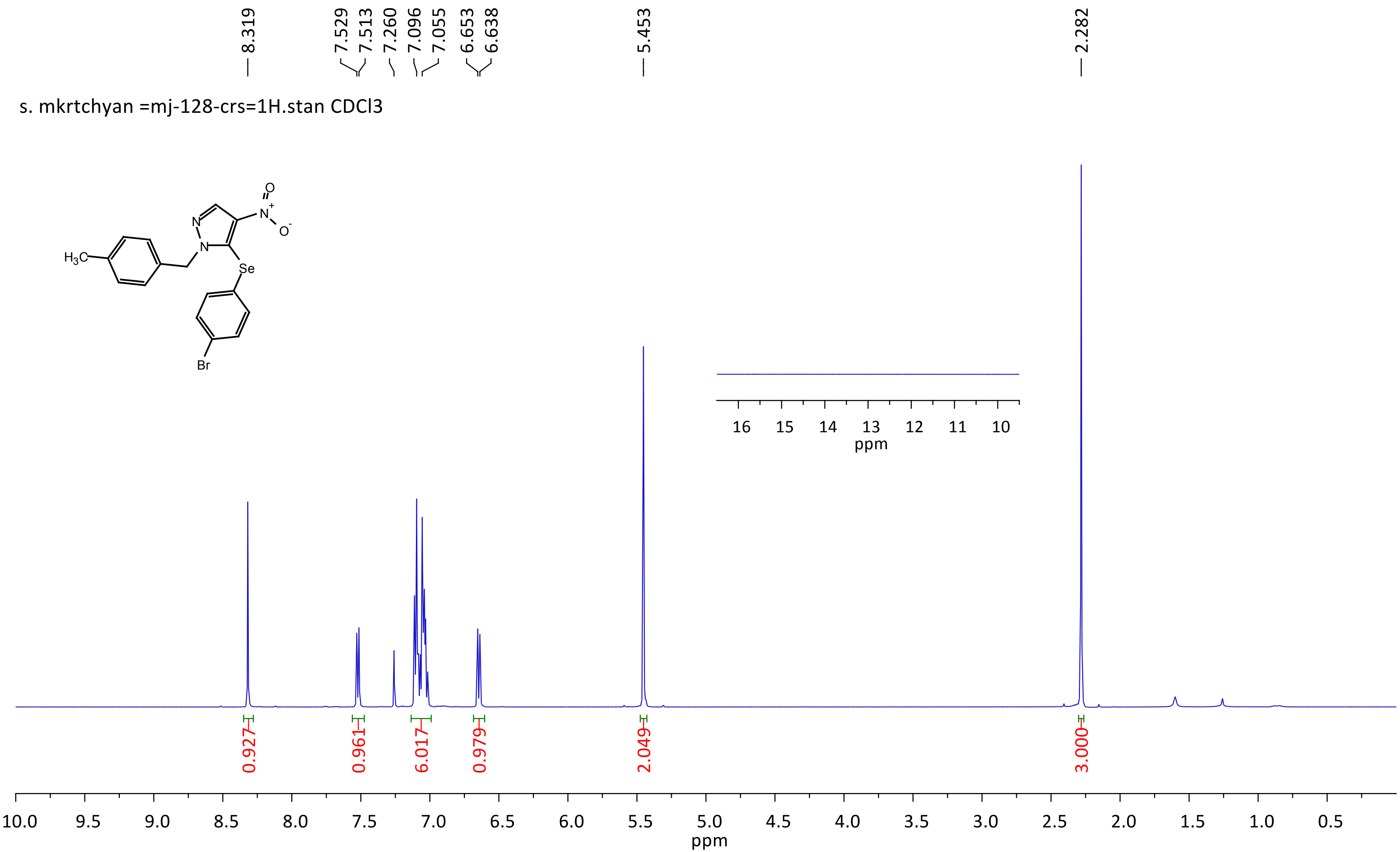

Comment	s. mkrtchyan =mj-128-crs=1H.stan CDCl3
Number of Scans	24
Spectrometer Frequency	500.13
Spectral Width	12335.5
Spectral Size	65536

Compound **1i**

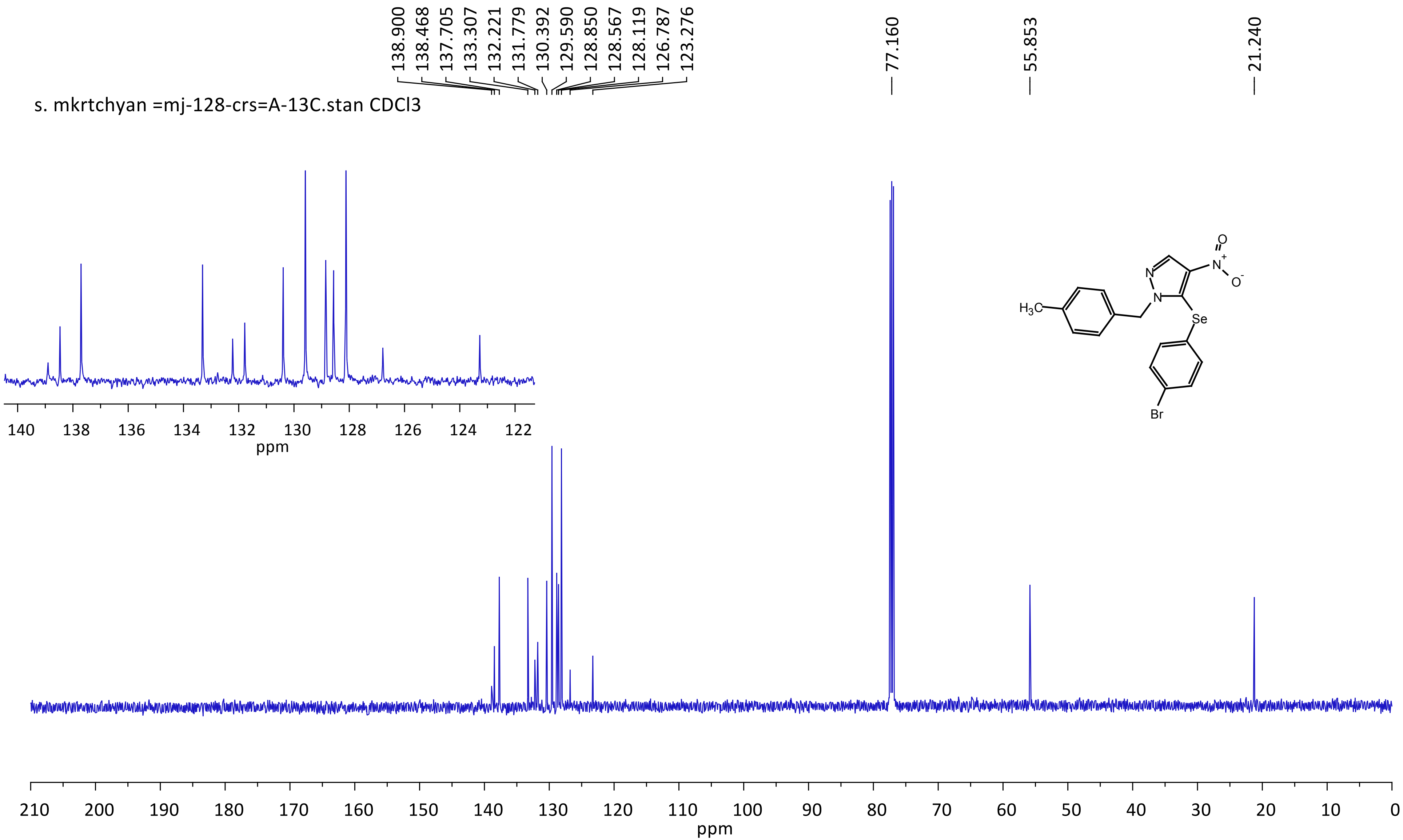

Comment	s. mkrtchyan =mj-128-crs=A-13C.stan CDCl3
Number of Scans	256
Spectrometer Frequency	125.76
Spectral Width	36057.7
Spectral Size	65536

Compound **2a**

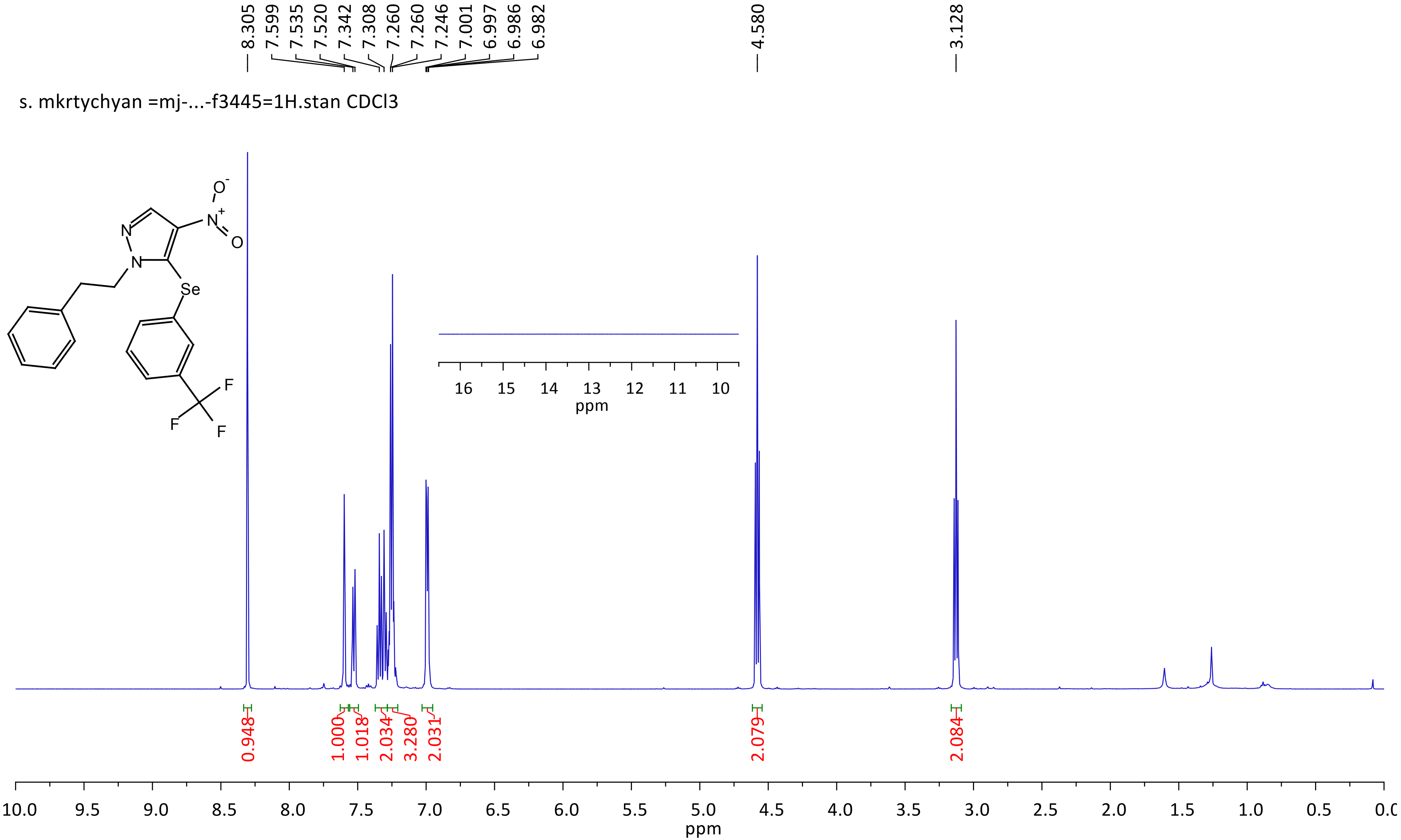

Comment	s. mkrtychyan =mj-...-f3445=1H.stan CDCl3
Number of Scans	24
Spectrometer Frequency	500.13
Spectral Width	12335.5
Spectral Size	65536

Compound **2a**

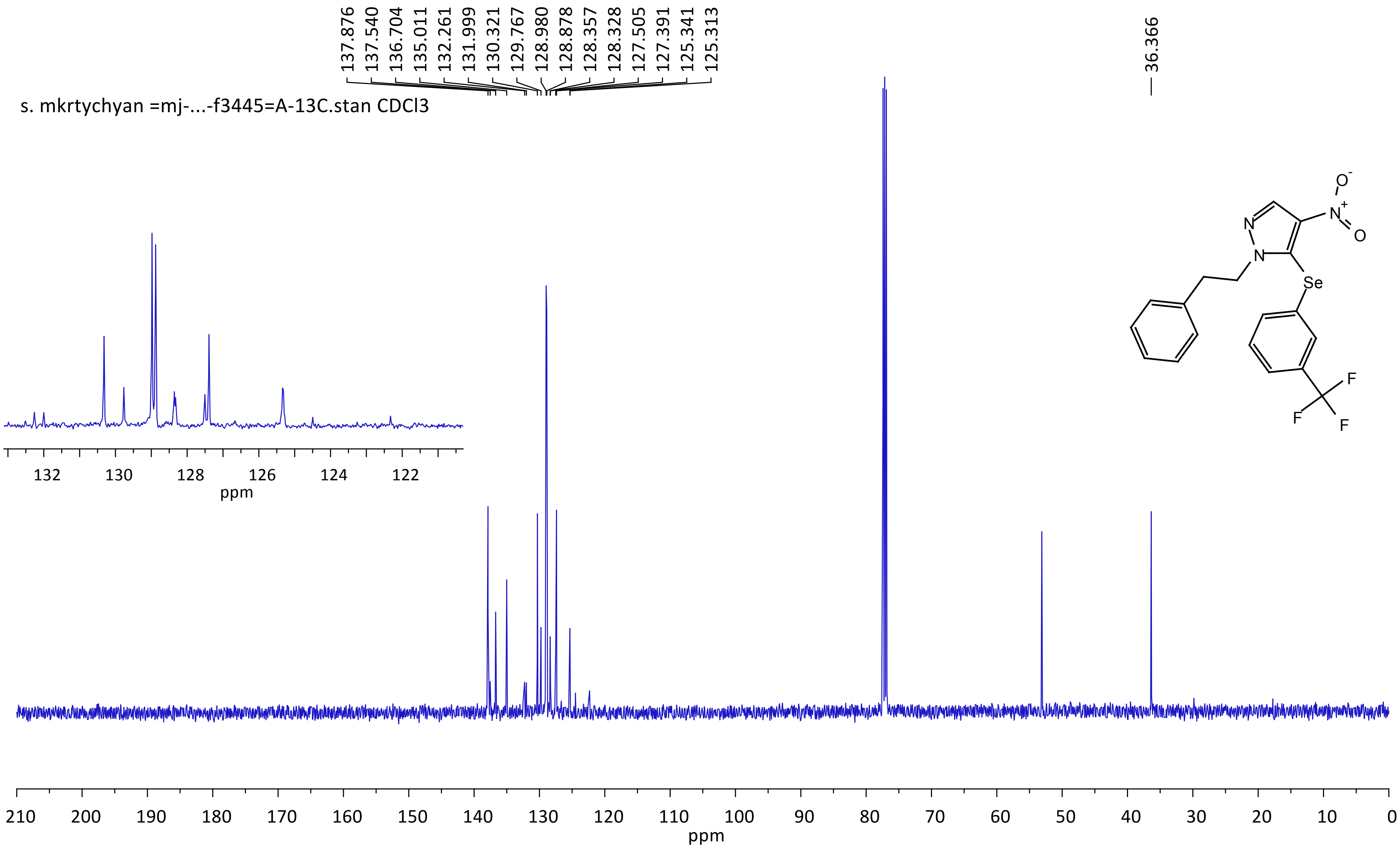

Comment	s. mkrtychyan =mj-...-f3445=A-13C.stan CDCl3
Number of Scans	256
Spectrometer Frequency	125.76
Spectral Width	36057.7
Spectral Size	65536

Compound **2b**

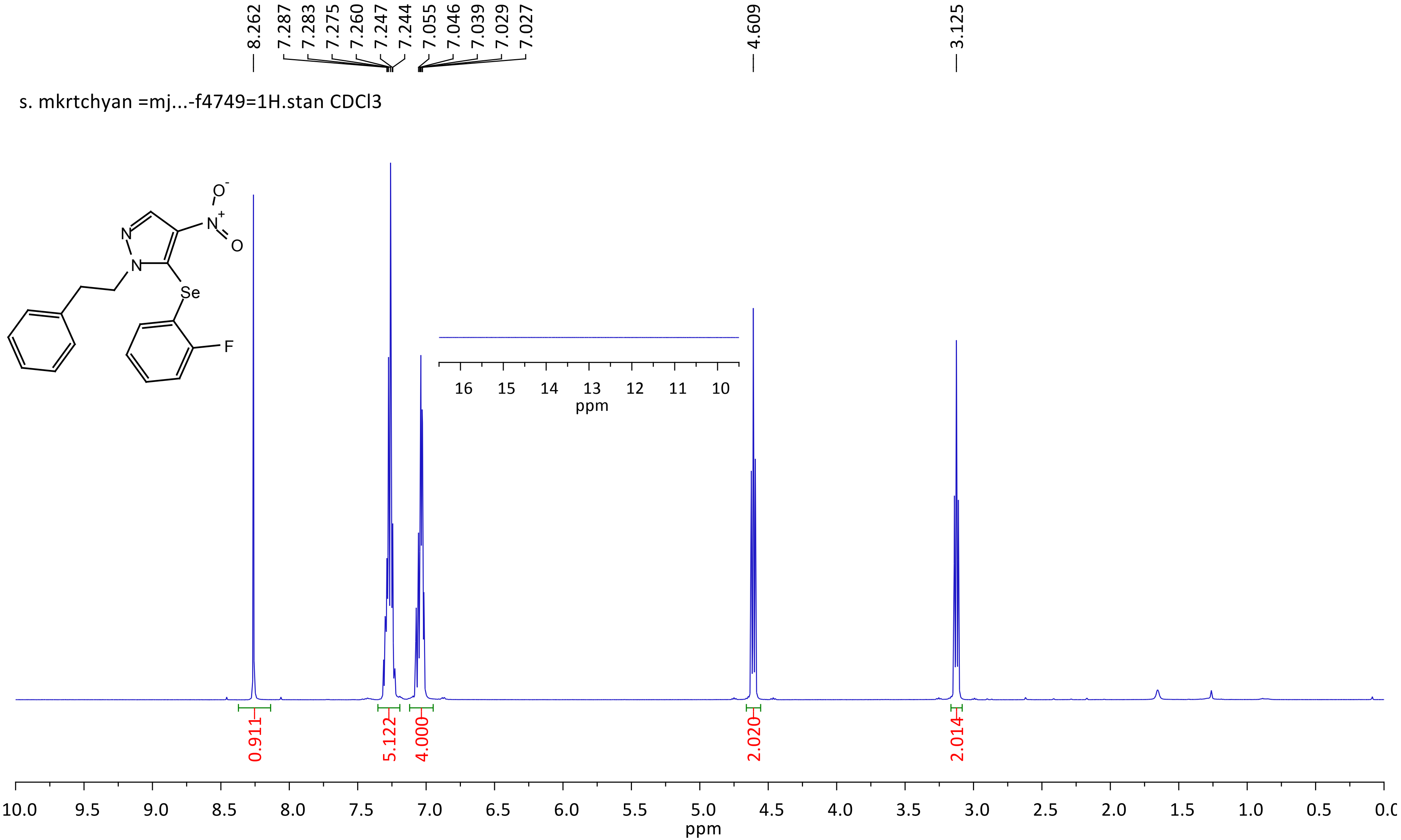

Comment	s. mkrtchyan =mj...-f4749=1H.stan CDCl3
Number of Scans	24
Spectrometer Frequency	500.13
Spectral Width	12335.5
Spectral Size	65536

Compound **2b**

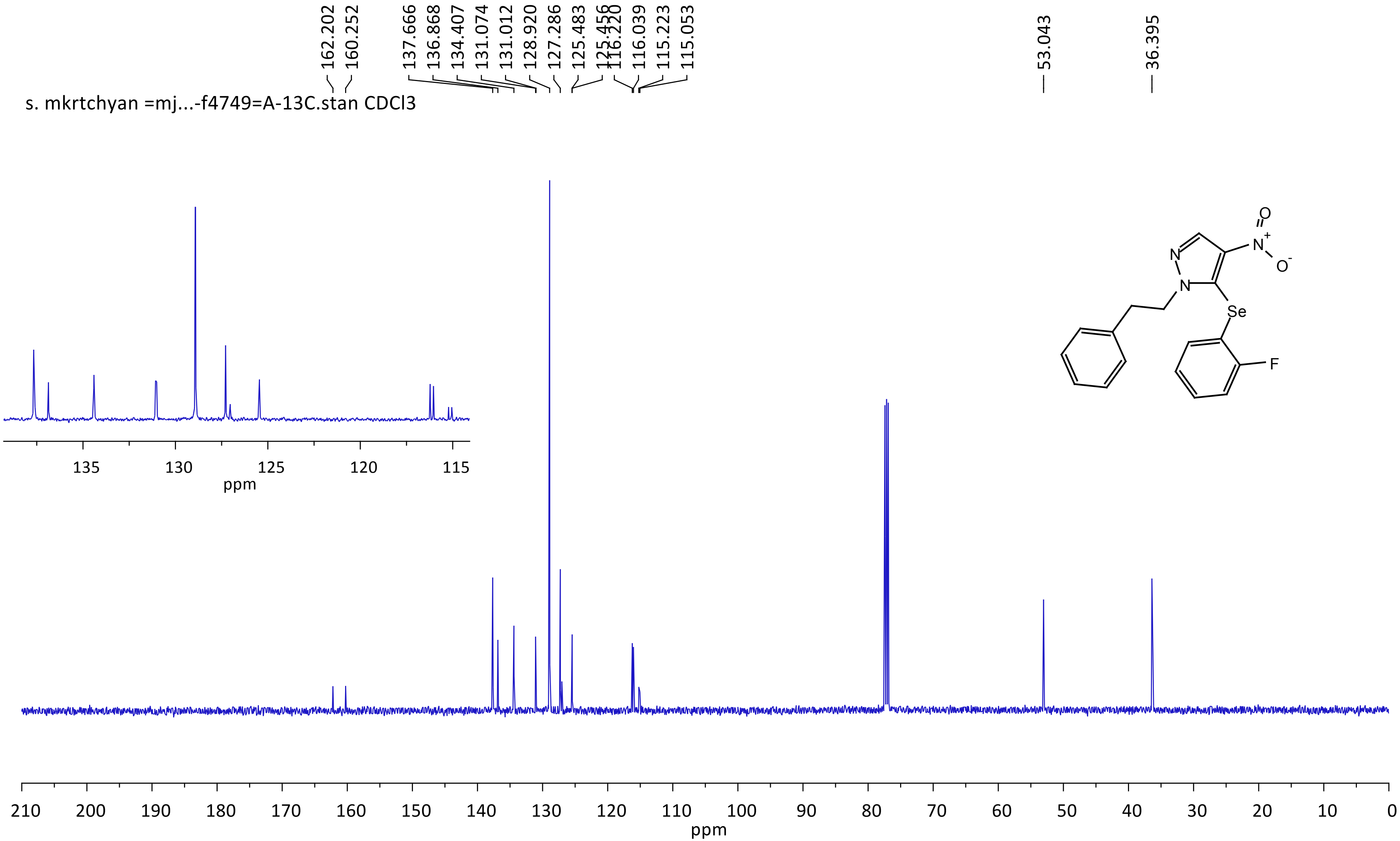

Comment	s. mkrtchyan =mj...-f4749=A-13C.stan CDCl3
Number of Scans	256
Spectrometer Frequency	125.76
Spectral Width	36057.7
Spectral Size	65536

Compound **2c**

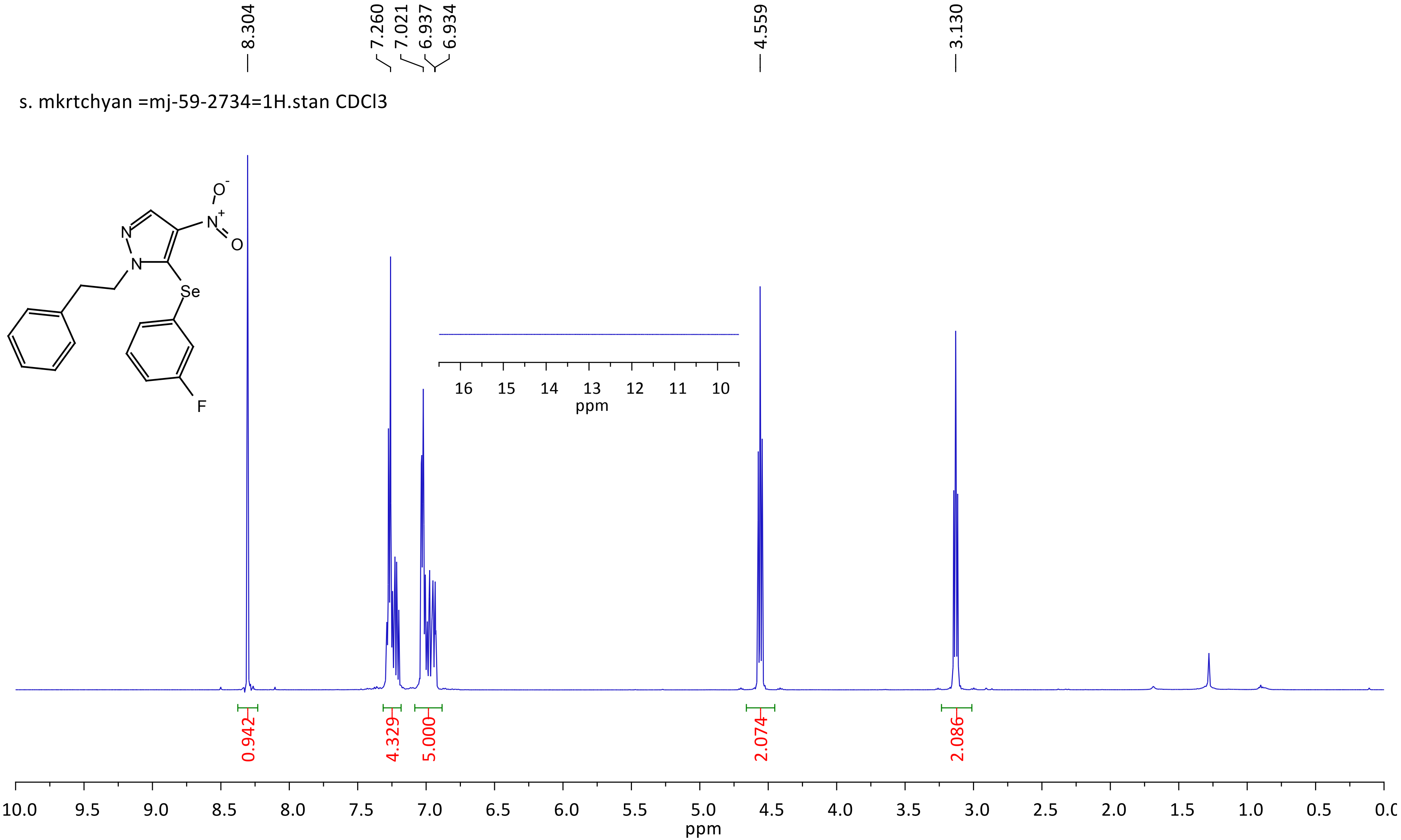

Comment	s. mkrtchyan =mj-59-2734=1H.stan CDCl3
Number of Scans	24
Spectrometer Frequency	500.13
Spectral Width	12335.5
Spectral Size	65536

Compound **2c**

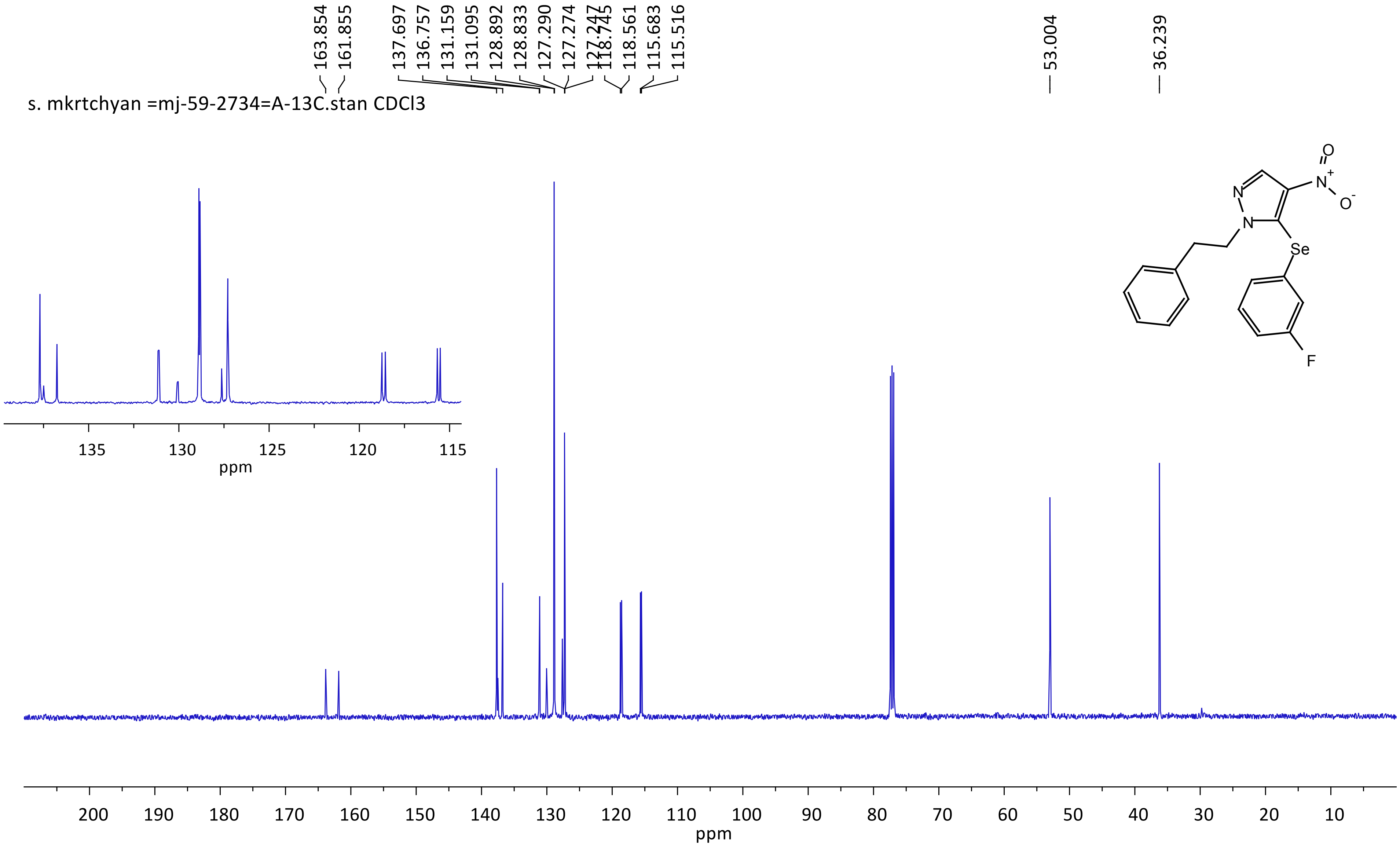

Comment	s. mkrtchyan =mj-59-2734=A-13C.stan CDCl3
Number of Scans	512
Spectrometer Frequency	125.76
Spectral Width	36057.7
Spectral Size	65536

Compound **2d**

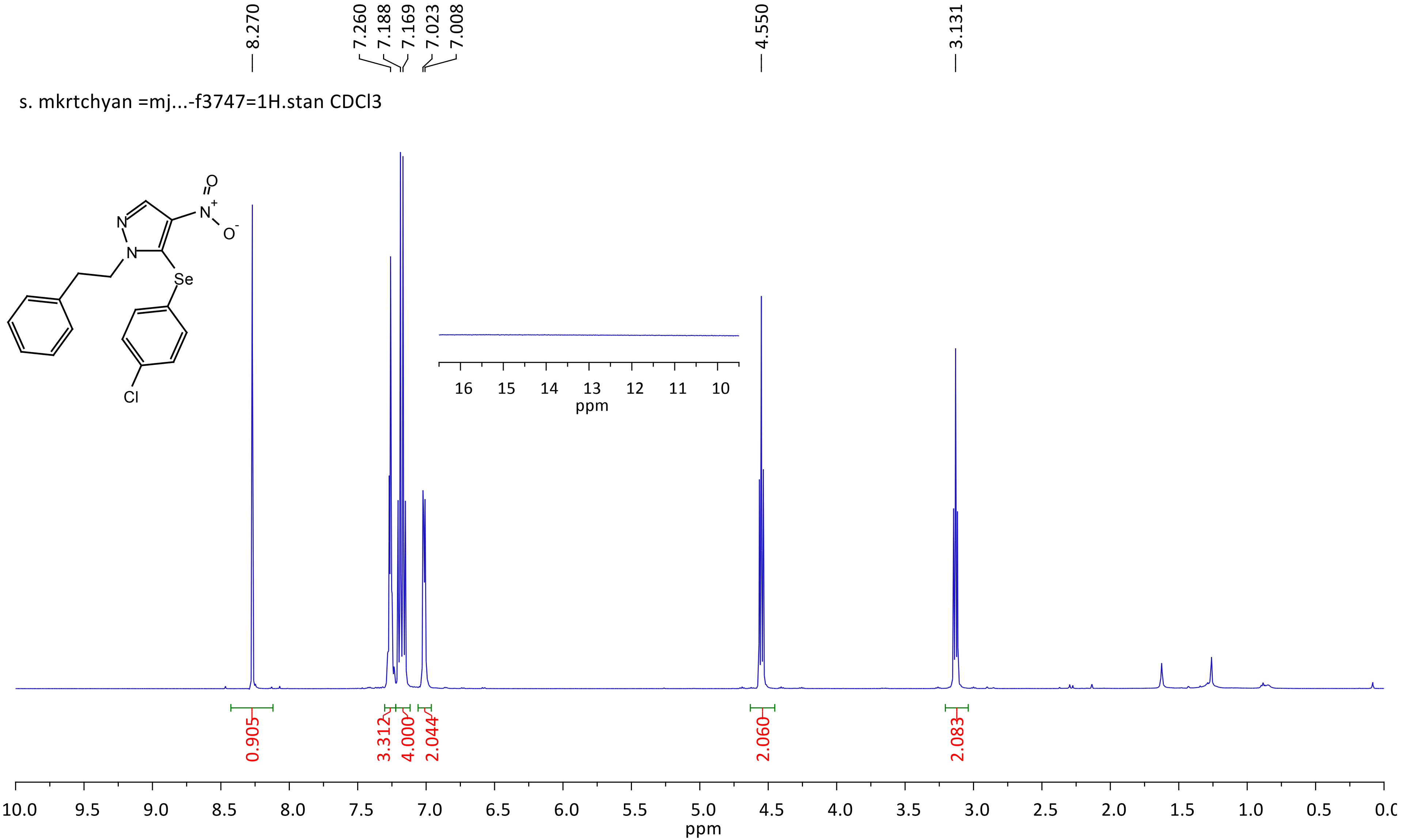

Comment	s. mkrtchyan =mj...-f3747=1H.stan CDCl3
Number of Scans	24
Spectrometer Frequency	500.13
Spectral Width	12335.5
Spectral Size	65536

Compound **2d**

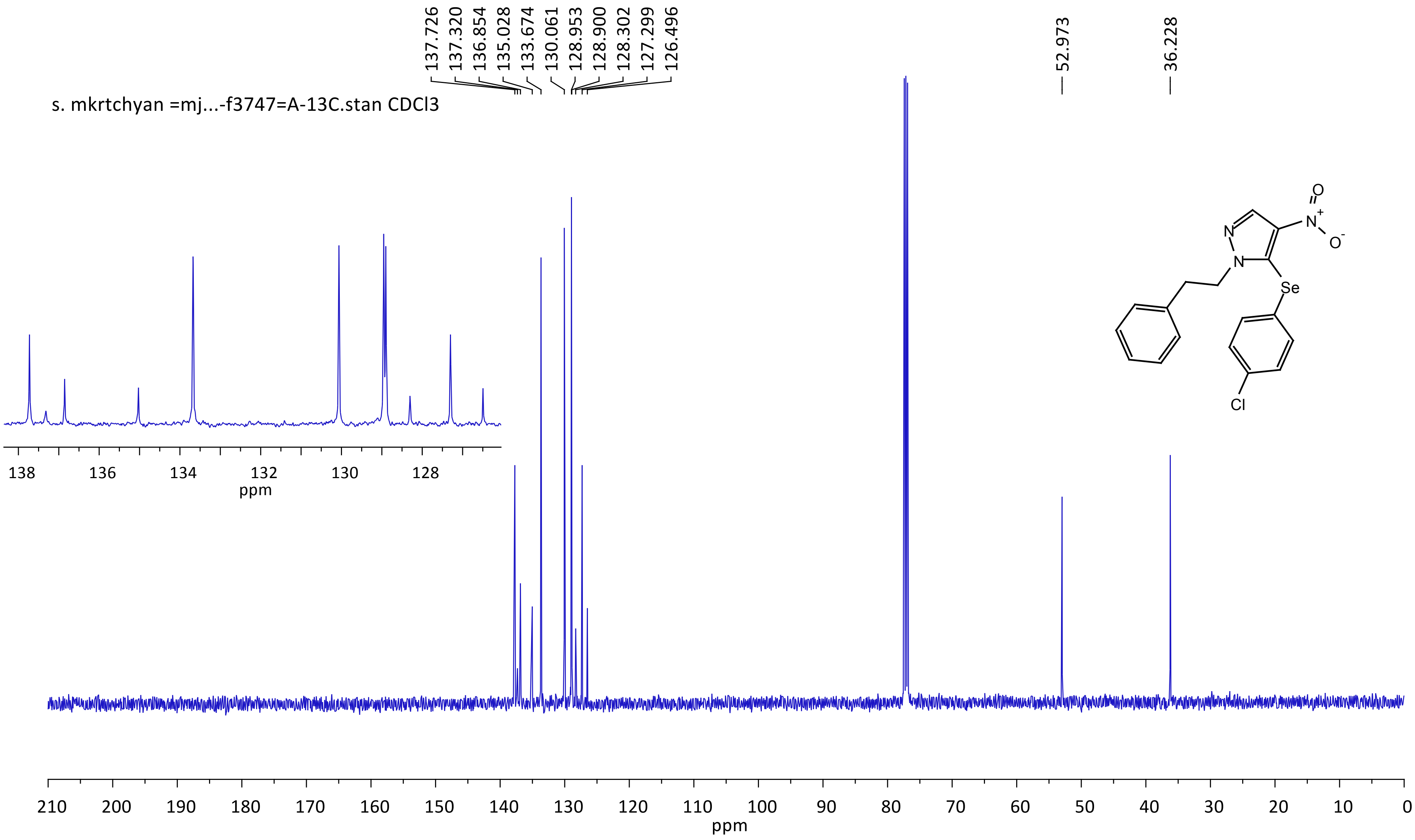

Comment	s. mkrtchyan =mj...-f3747=A-13C.stan CDCl3
Number of Scans	256
Spectrometer Frequency	125.76
Spectral Width	36057.7
Spectral Size	65536

Compound **2e**

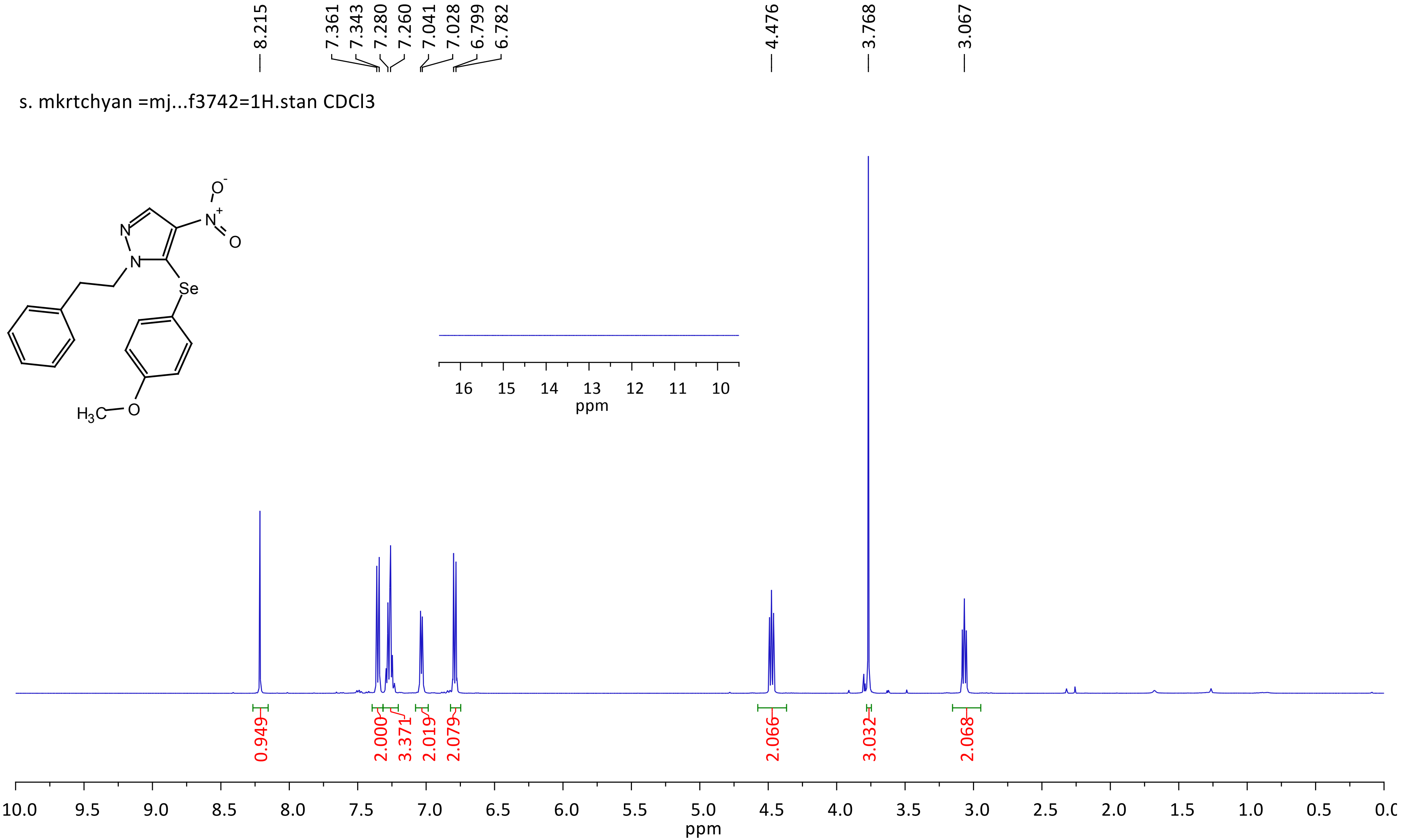

Comment	s. mkrtchyan =mj...f3742=1H.stan CDCl3
Number of Scans	24
Spectrometer Frequency	500.13
Spectral Width	12335.5
Spectral Size	65536

Compound **2e**

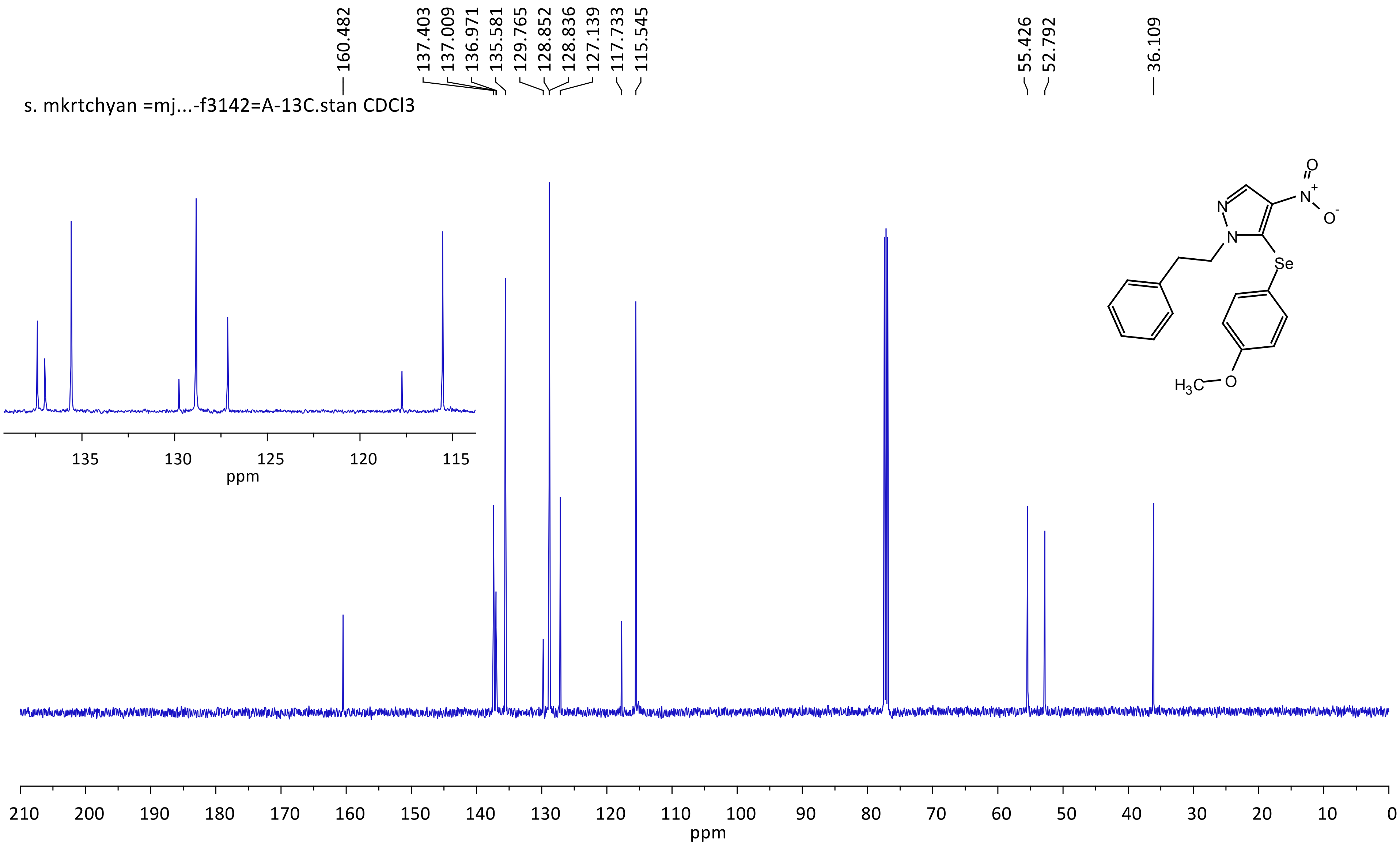

Comment	s. mkrtchyan =mj...-f3142=<br>A-13C.stan CDCl3 {C:\ NMR_Data\ Service\ CBMM} nmrsu 9
Number of Scans	256
Spectrometer Frequency	125.76
Spectral Width	36057.7
[illegible]	[illegible]

Compound **2f**

s. mkrtchyan =mj60=1H.stan CDCl3

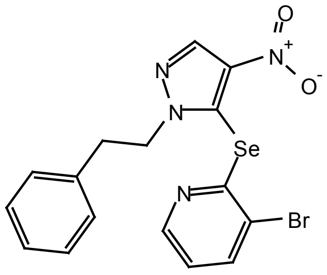

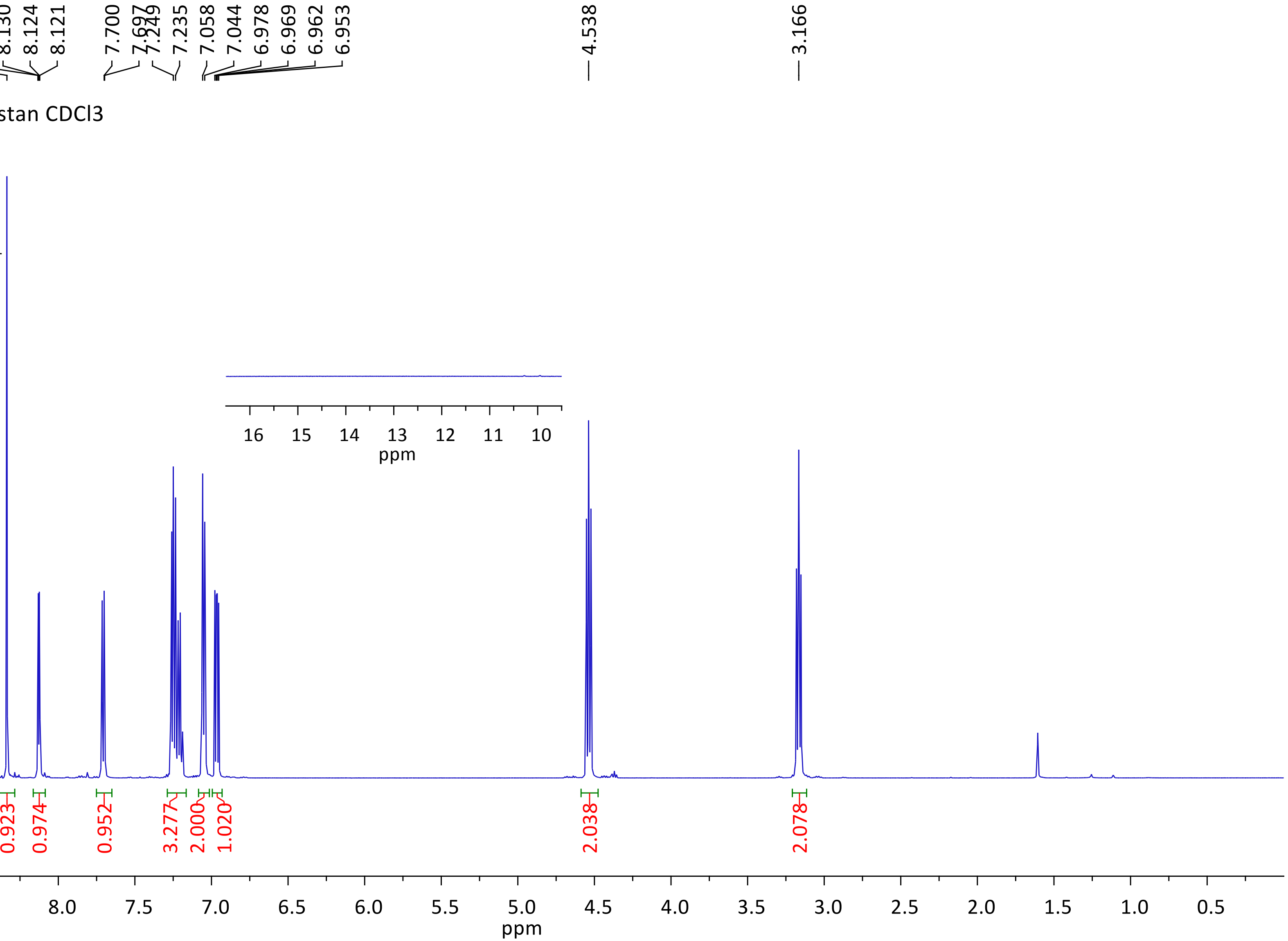

Comment	s. mkrtchyan =mj60=1H.stan CDCl3
Number of Scans	24
Spectrometer Frequency	500.13
Spectral Width	12335.5
Spectral Size	65536

Compound **2f**

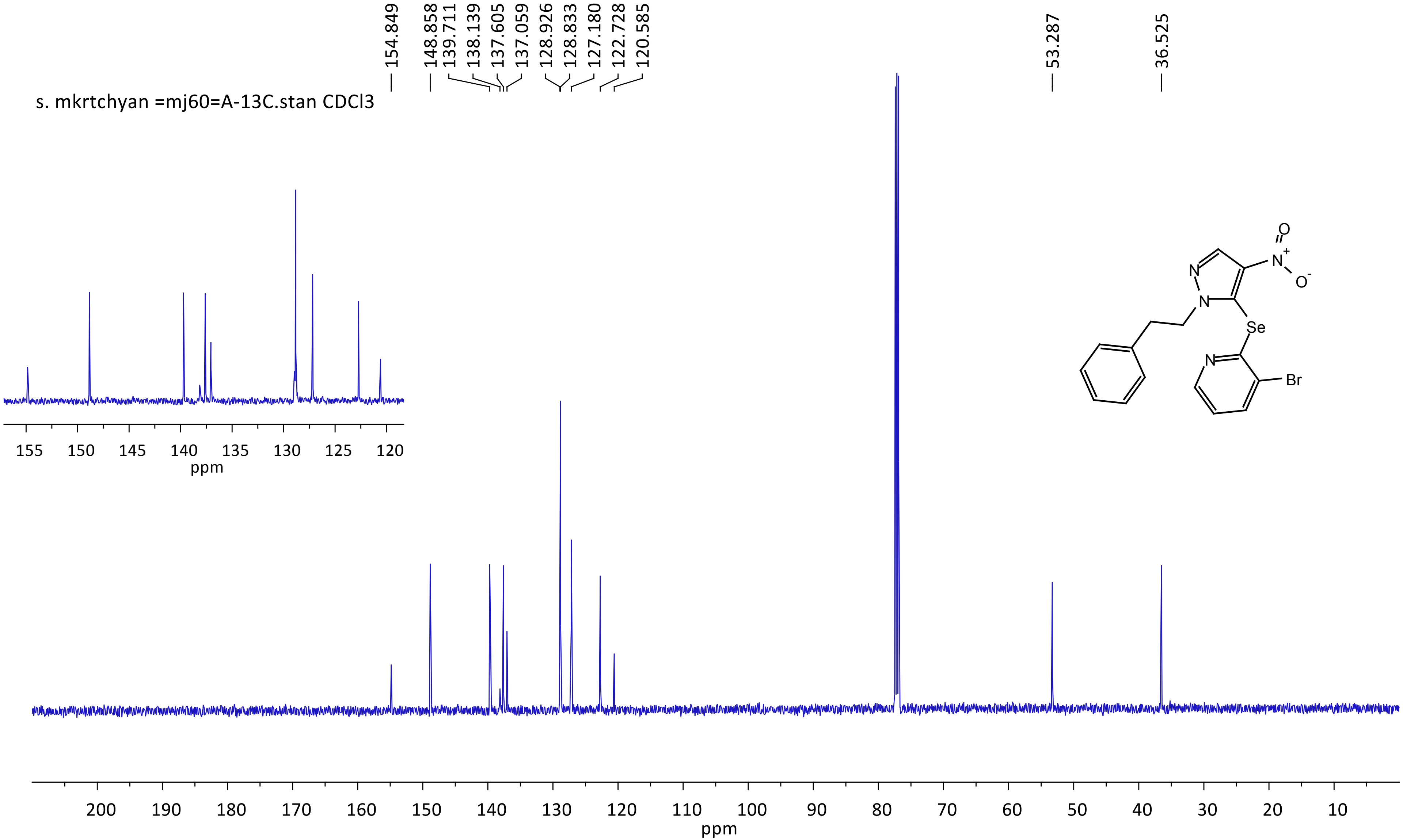

Comment	s. mkrtchyan =mj60=<br>A-13C.stan CDCl3 {C:\ NMR_Data\ Service\ CBMM} nmrsu 6
Number of Scans	512
Spectrometer Frequency	125.76
Spectral Width	36057.7
[illegible]	[illegible]

Compound **3a**

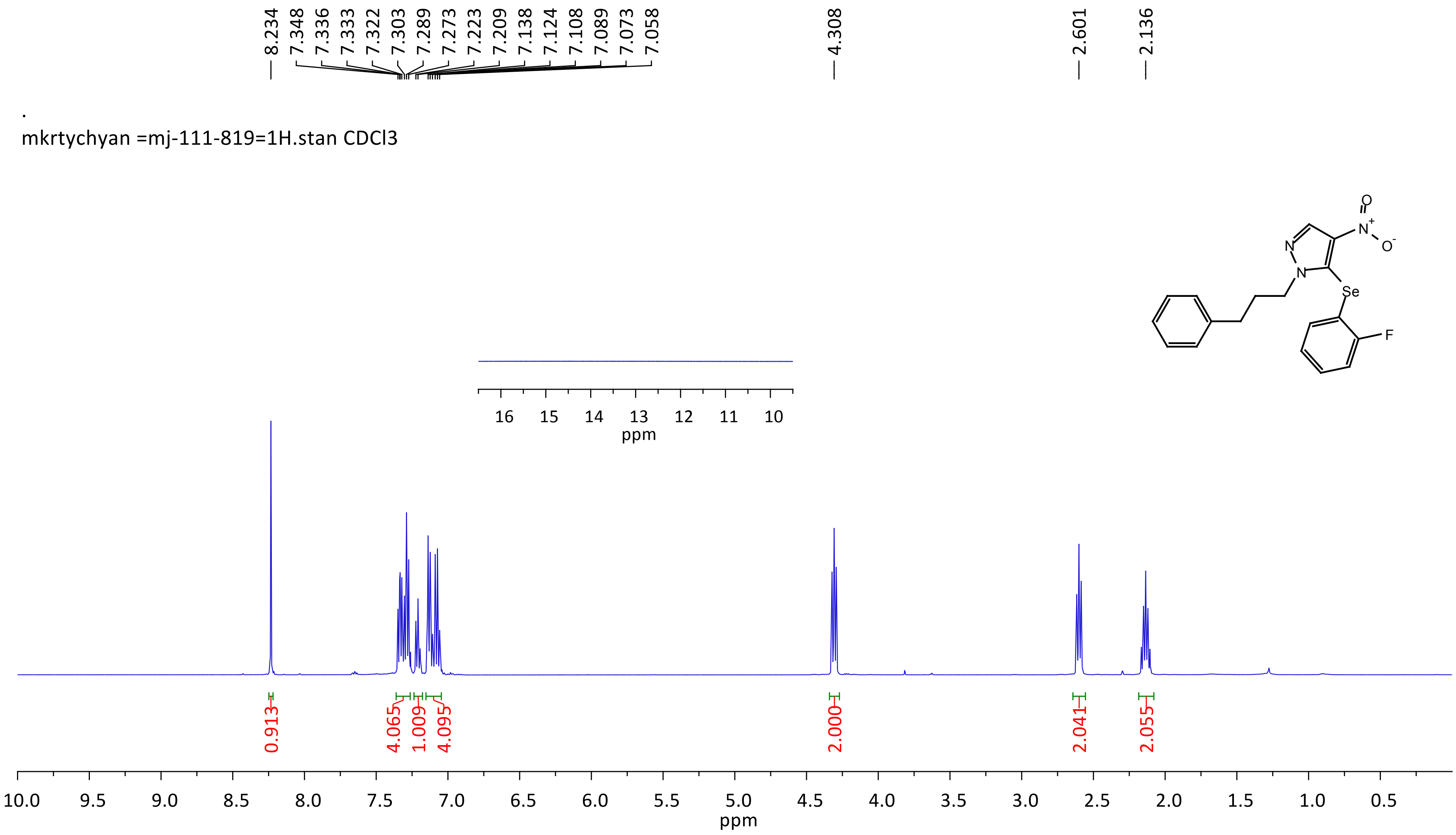

Comment	s. mkrtychyan =mj-111-819=1H.stan CDCl3
Number of Scans	24
Spectrometer Frequency	500.13
Spectral Width	12335.5
Spectral Size	65536

Compound **3a**

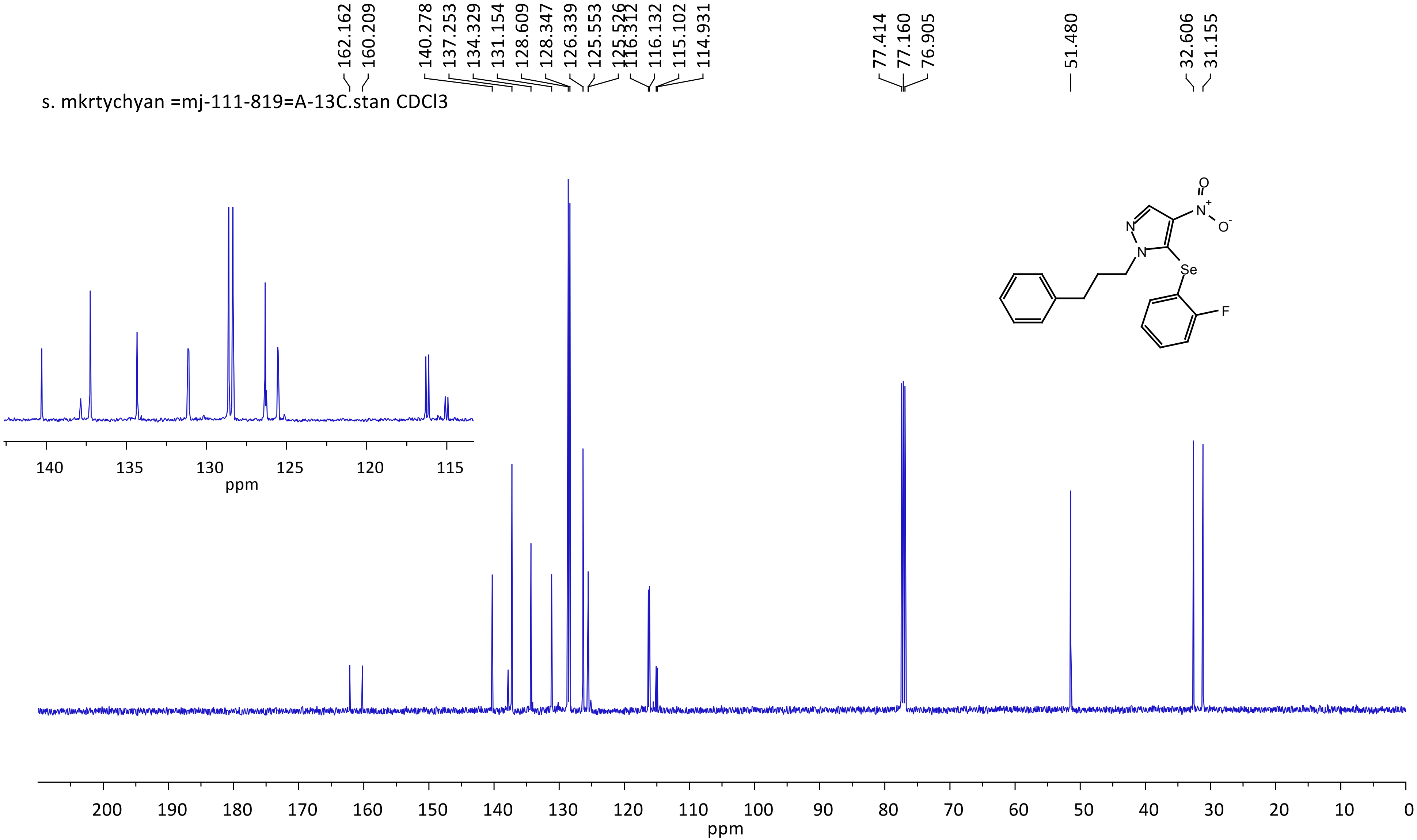

Comment	s. mkrtychyan =mj-111-819=A-13C.stan CDCl3
Number of Scans	512
Spectrometer Frequency	125.76
Spectral Width	36057.7
Spectral Size	65536

Compound **3b**

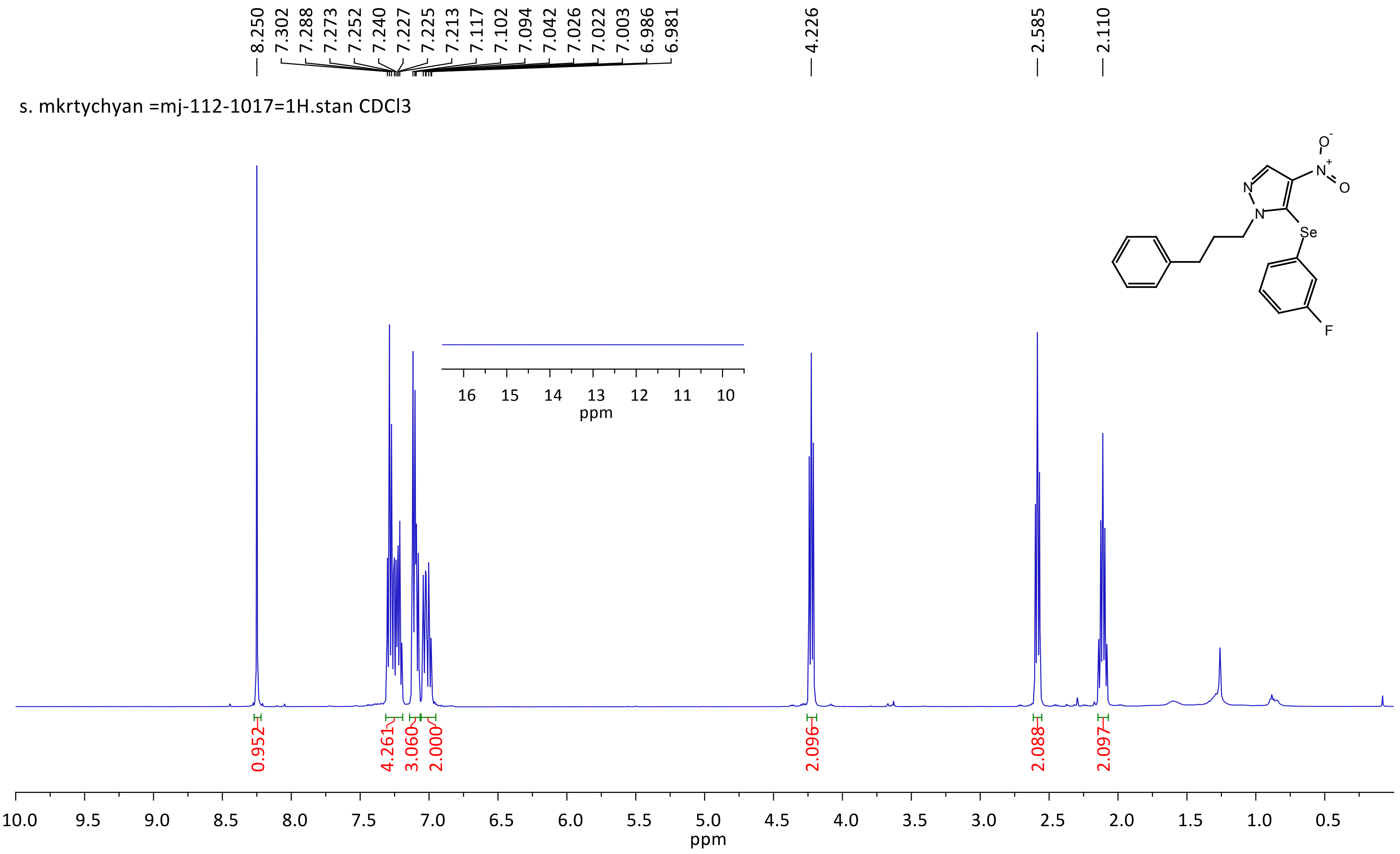

Comment	s. mkrtychyan =mj-112-1017=1H.stan CDCl3
Number of Scans	24
Spectrometer Frequency	500.13
Spectral Width	12335.5
Spectral Size	65536

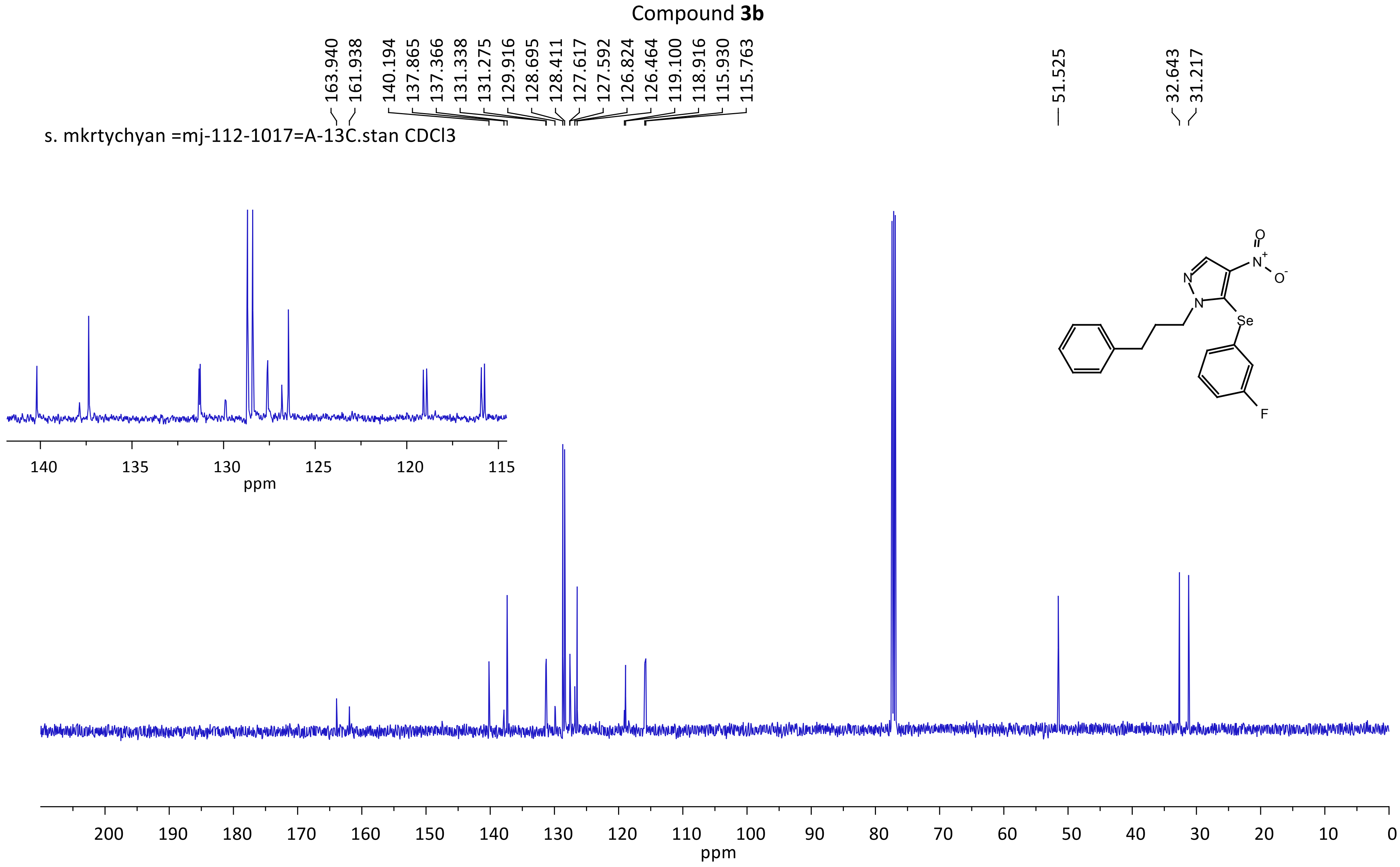

Comment	s. mkrtychyan =mj-112-1017=A-13C.stan CDCl3
Number of Scans	256
Spectrometer Frequency	125.76
Spectral Width	36057.7
Spectral Size	65536

Compound **3c**

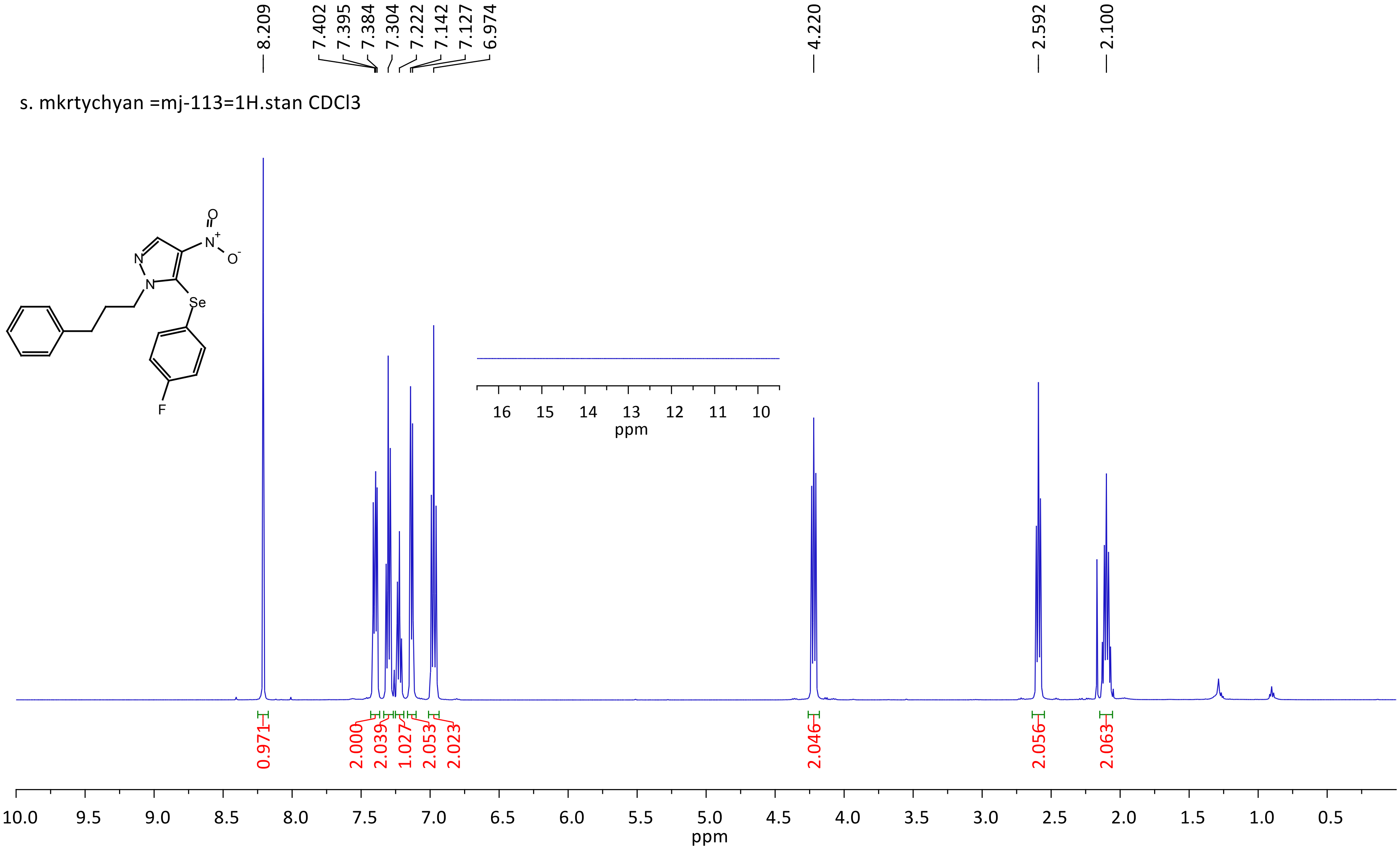

Comment	s. mkrtychyan =mj-113=1H.stan CDCl3
Number of Scans	24
Spectrometer Frequency	500.13
Spectral Width	12335.5
Spectral Size	65536

Compound **3c**

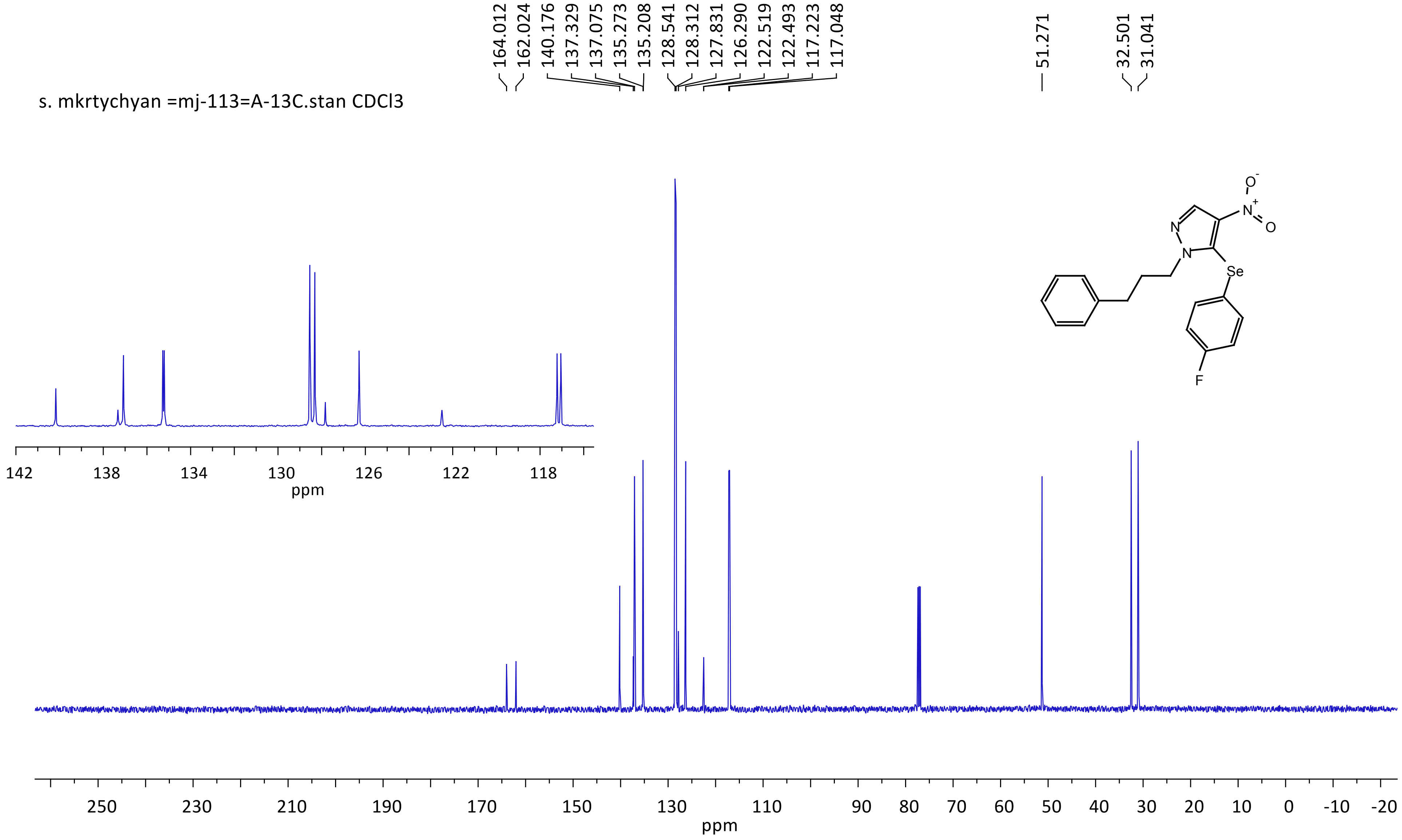

Comment	s. mkrtychyan =mj-113=A-13C.stan CDCl3
Number of Scans	128
Spectrometer Frequency	125.76
Spectral Width	36057.7
Spectral Size	65536

Compound **4a**

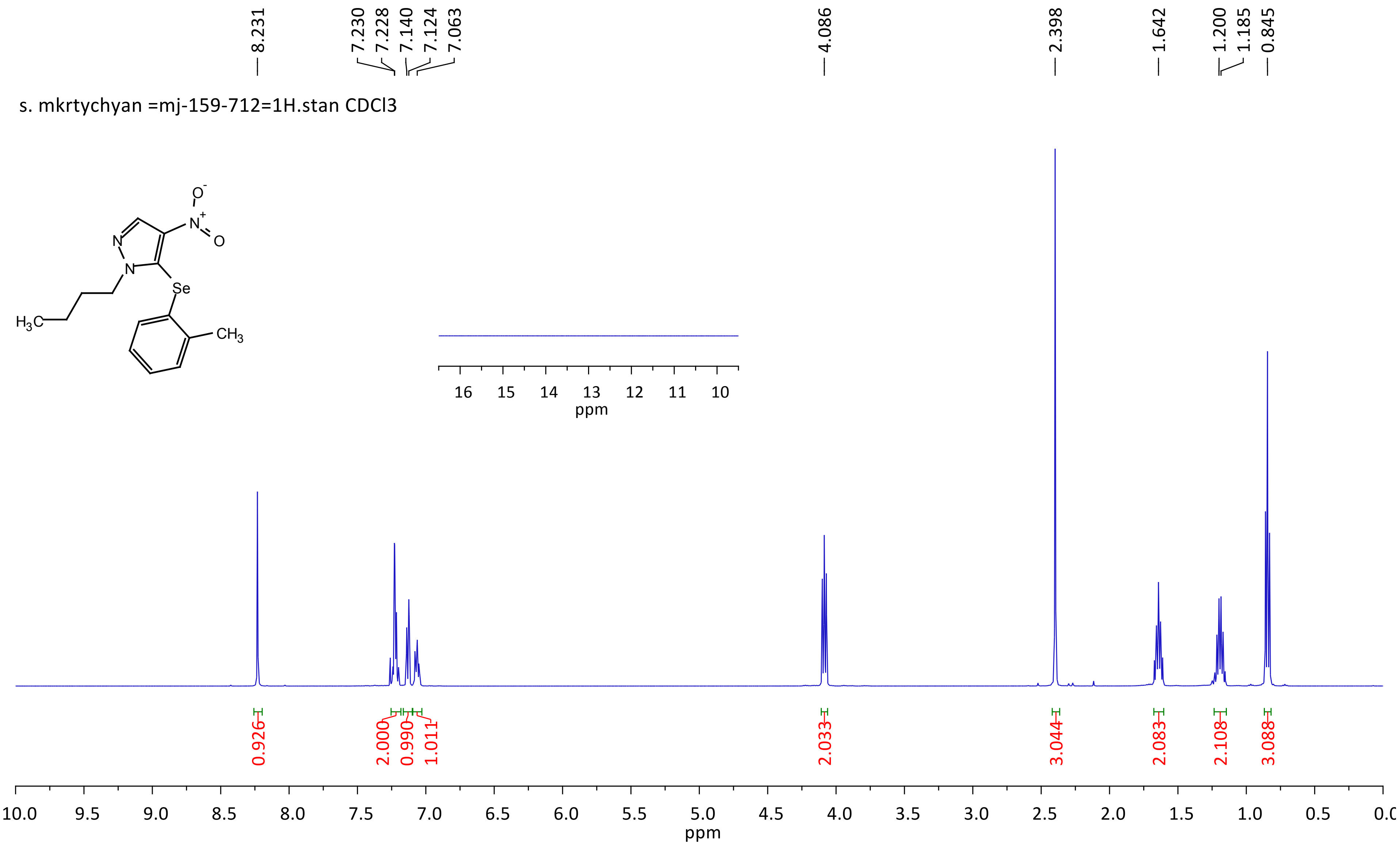

Comment	s. mkrtychyan =mj-159-712=1H.stan CDCl3
Number of Scans	24
Spectrometer Frequency	500.13
Spectral Width	12335.5
Spectral Size	65536

Compound **4a**

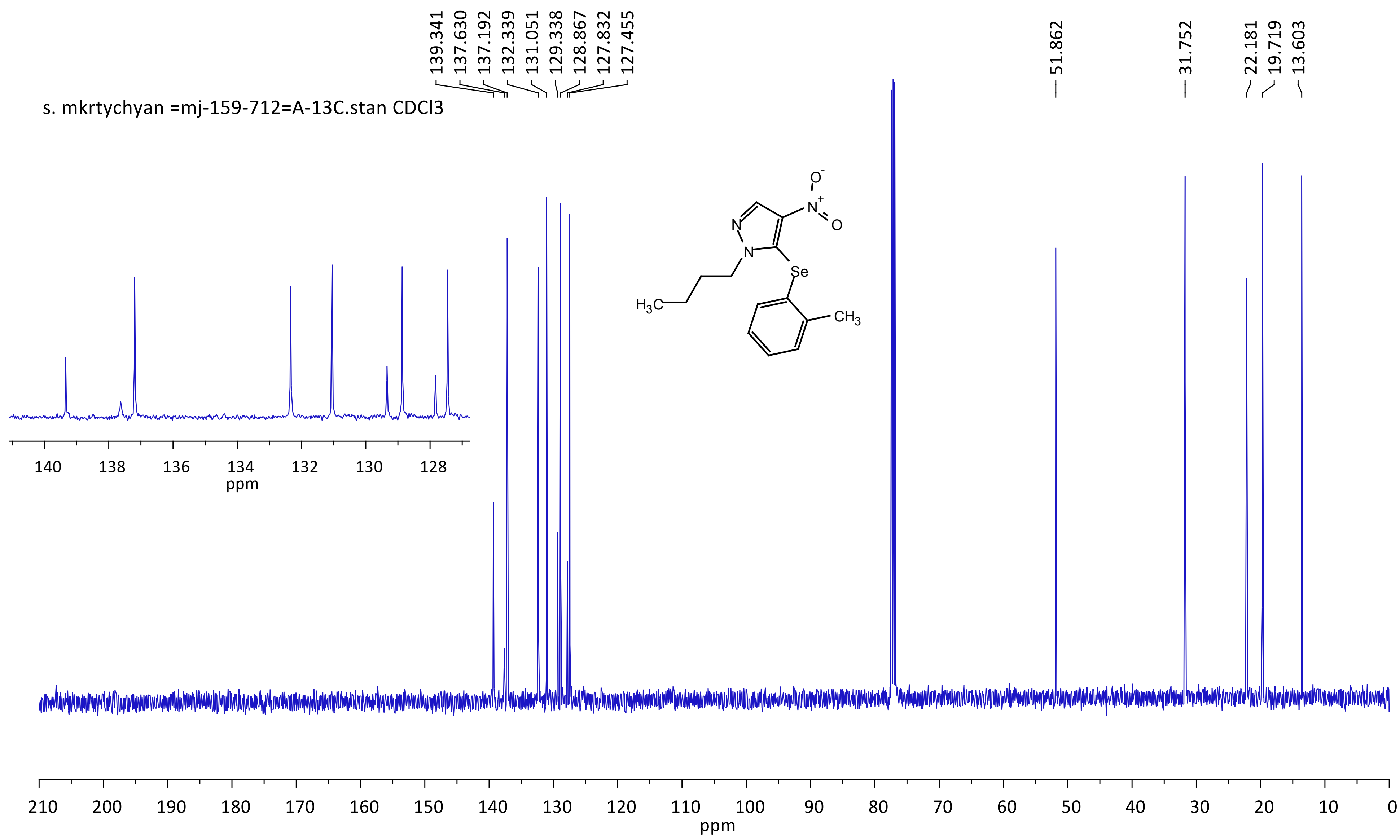

Comment	s. mkrtychyan =mj-159-712=A-13C.stan CDCl3
Number of Scans	296
Spectrometer Frequency	125.76
Spectral Width	36057.7
Spectral Size	65536

Compound **4b**

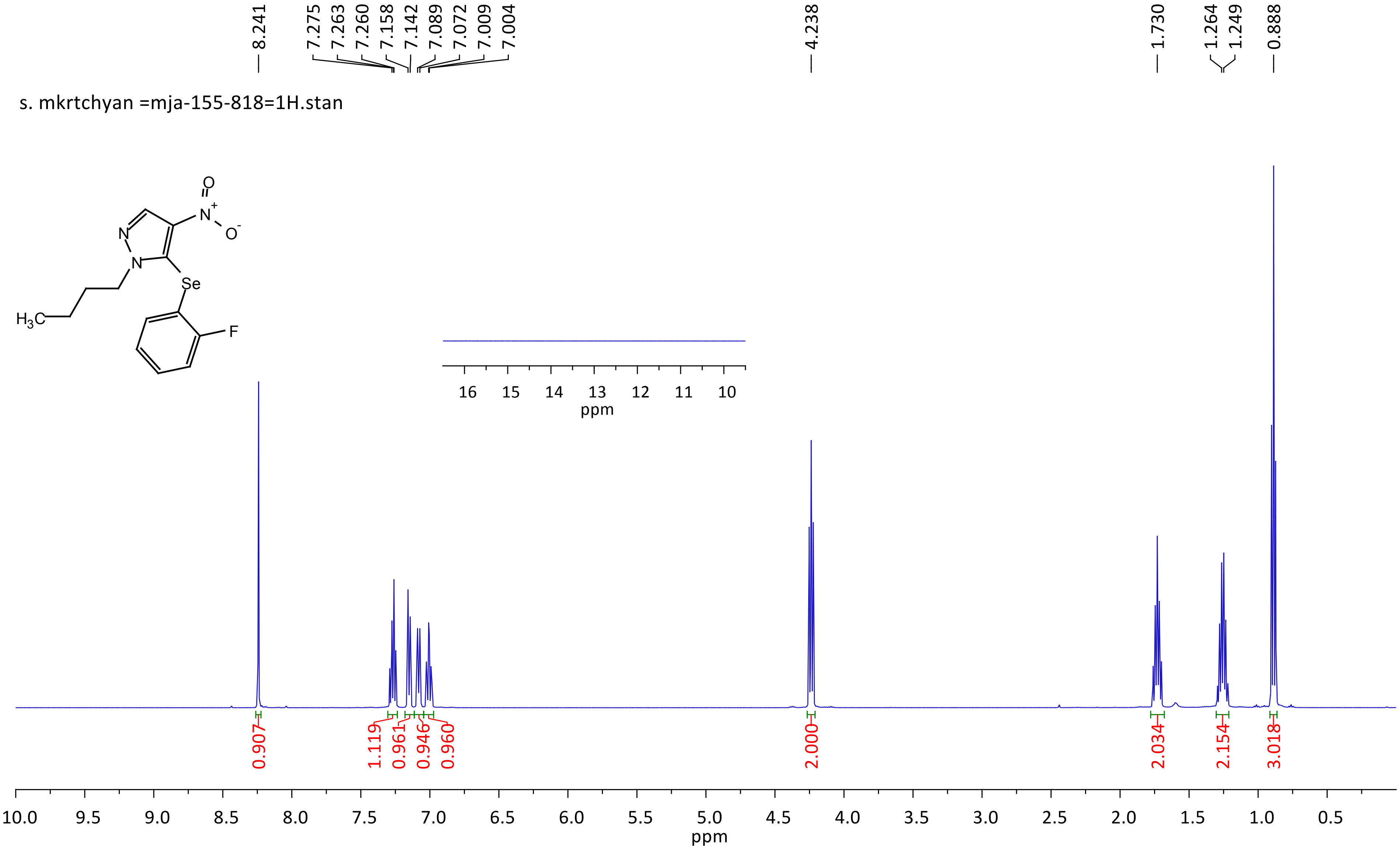

Comment	s. mkrtchyan =mja-155-818=1H.stan
Number of Scans	24
Spectrometer Frequency	500.13
Spectral Width	12335.5
Spectral Size	65536

Compound **4b**

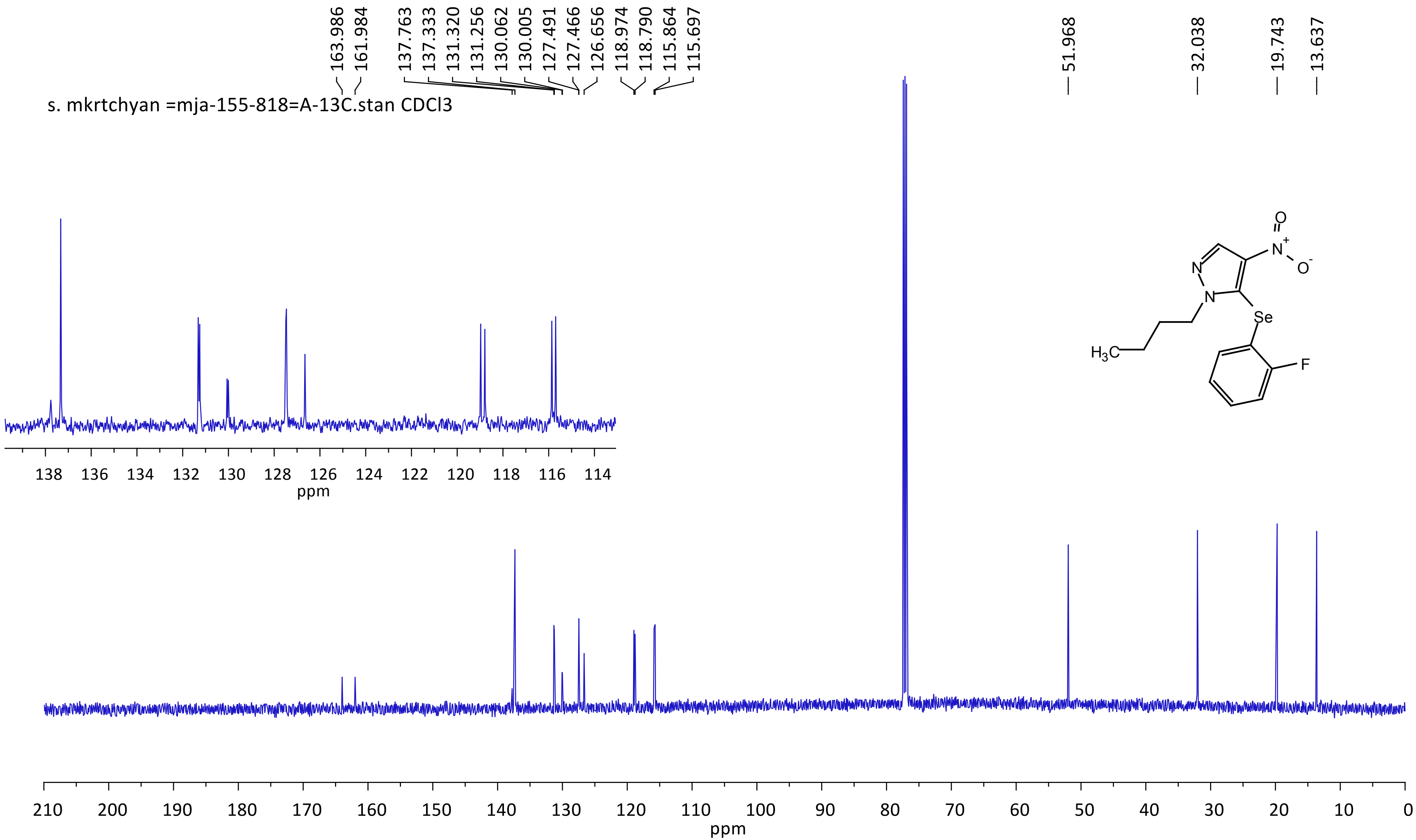

Comment	s. mkrtchyan =mja-155-818=A-13C.stan CDCl3
Number of Scans	512
Spectrometer Frequency	125.76
Spectral Width	36057.7
Spectral Size	65536

Compound **4c**

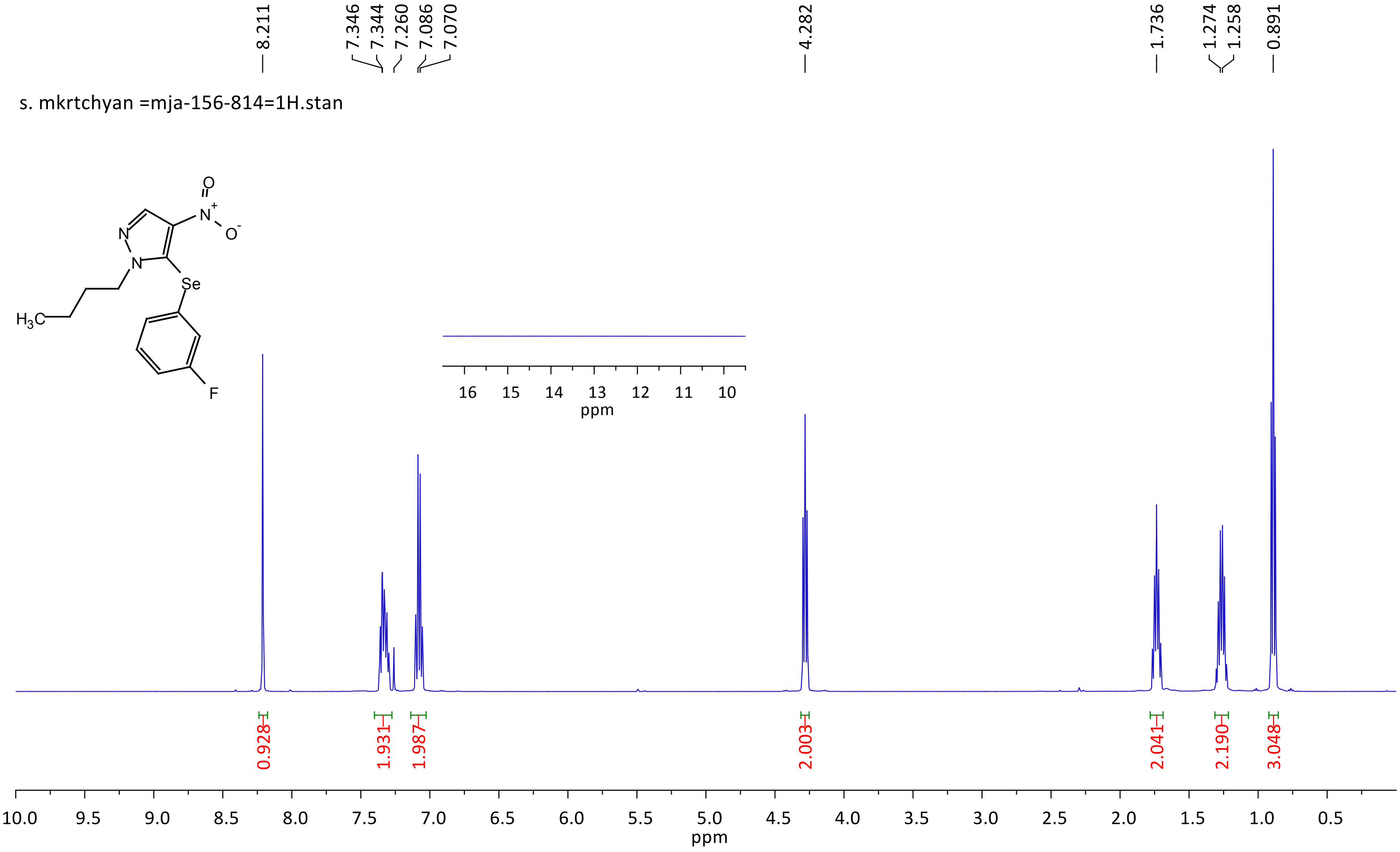

Comment	s. mkrtchyan =mja-156-814=1H.stan
Number of Scans	24
Spectrometer Frequency	500.13
Spectral Width	12335.5
Spectral Size	65536

Compound **4c**

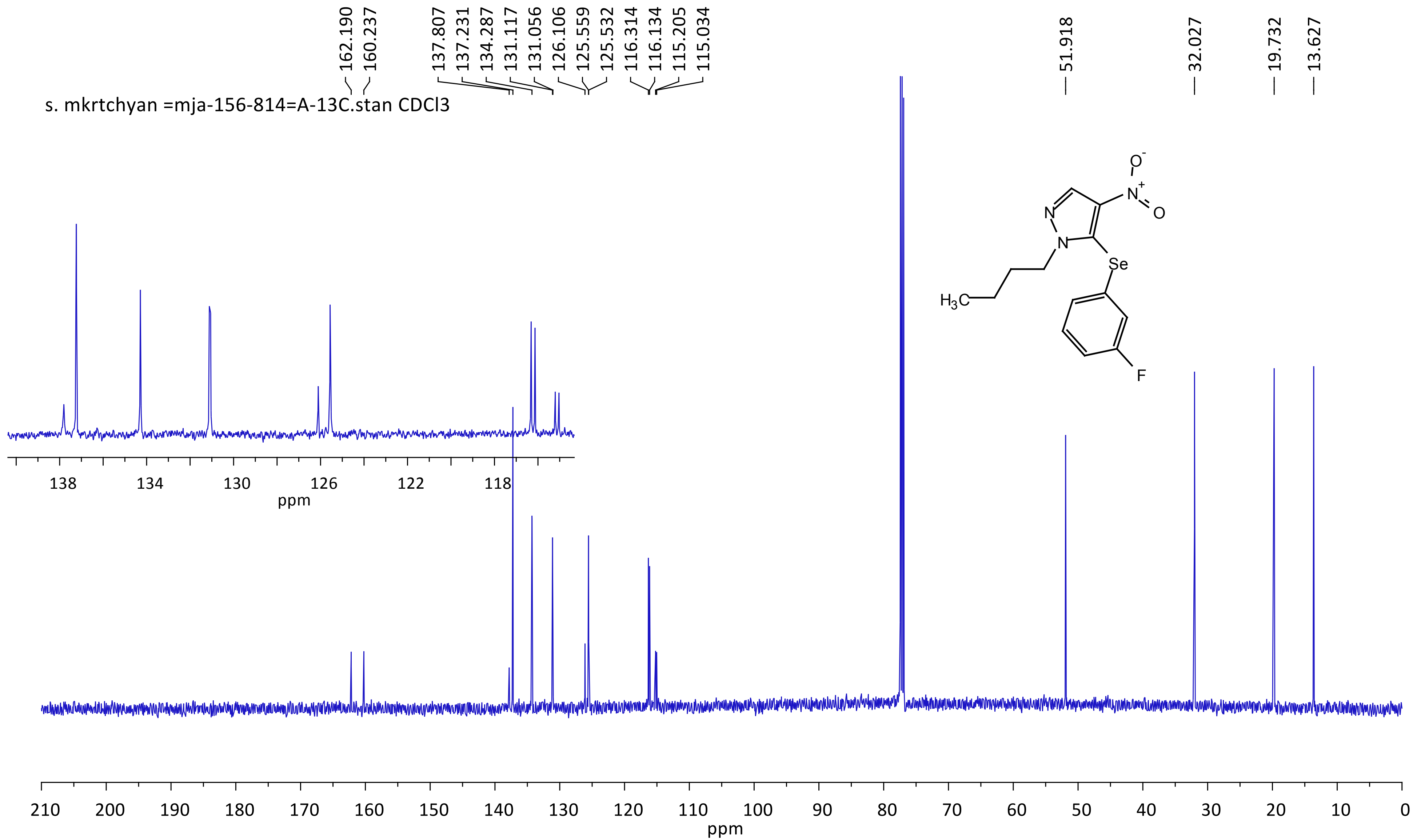

Comment	s. mkrtchyan =mja-156-814=A-13C.stan CDCl3
Number of Scans	512
Spectrometer Frequency	125.76
Spectral Width	36057.7
Spectral Size	65536

Compound **4d**

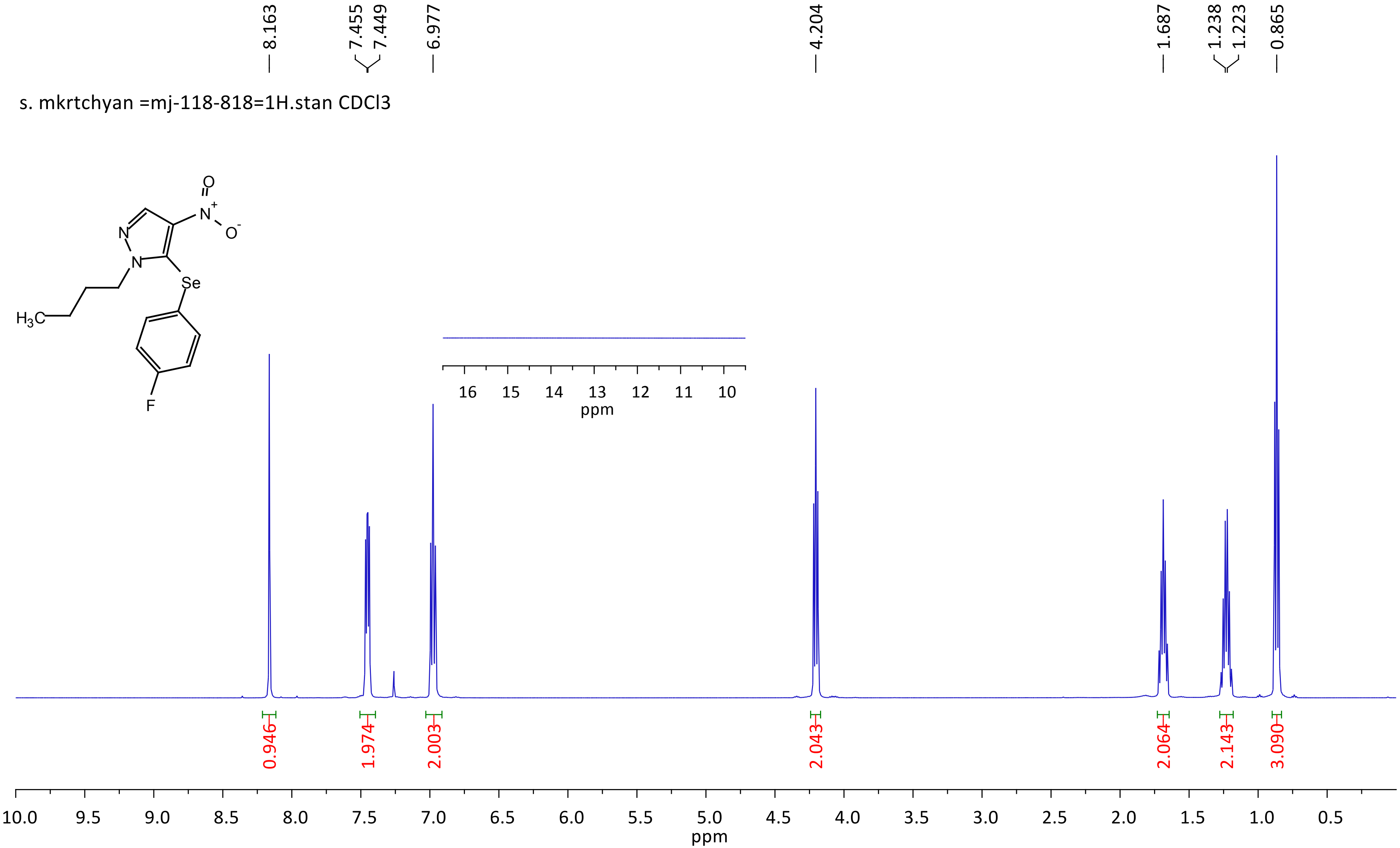

Comment	s. mkrtchyan =mj-118-818=1H.stan CDCl3
Number of Scans	24
Spectrometer Frequency	500.13
Spectral Width	12335.5
Spectral Size	65536

Compound **4d**

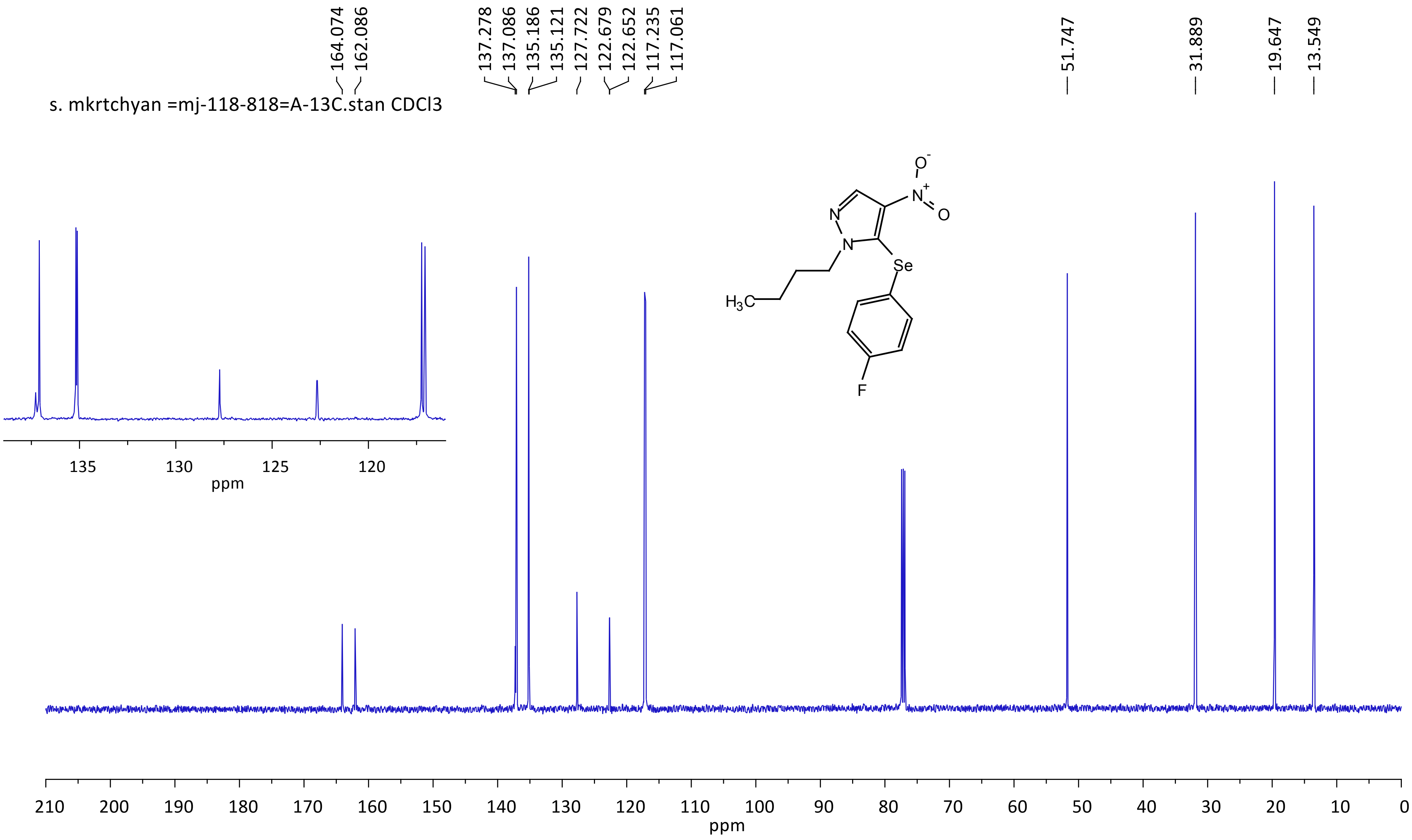

Comment	s. mkrtchyan =mj-118-818=A-13C.stan CDCl3
Number of Scans	256
Spectrometer Frequency	125.76
Spectral Width	36057.7
Spectral Size	65536

Compound **5a**

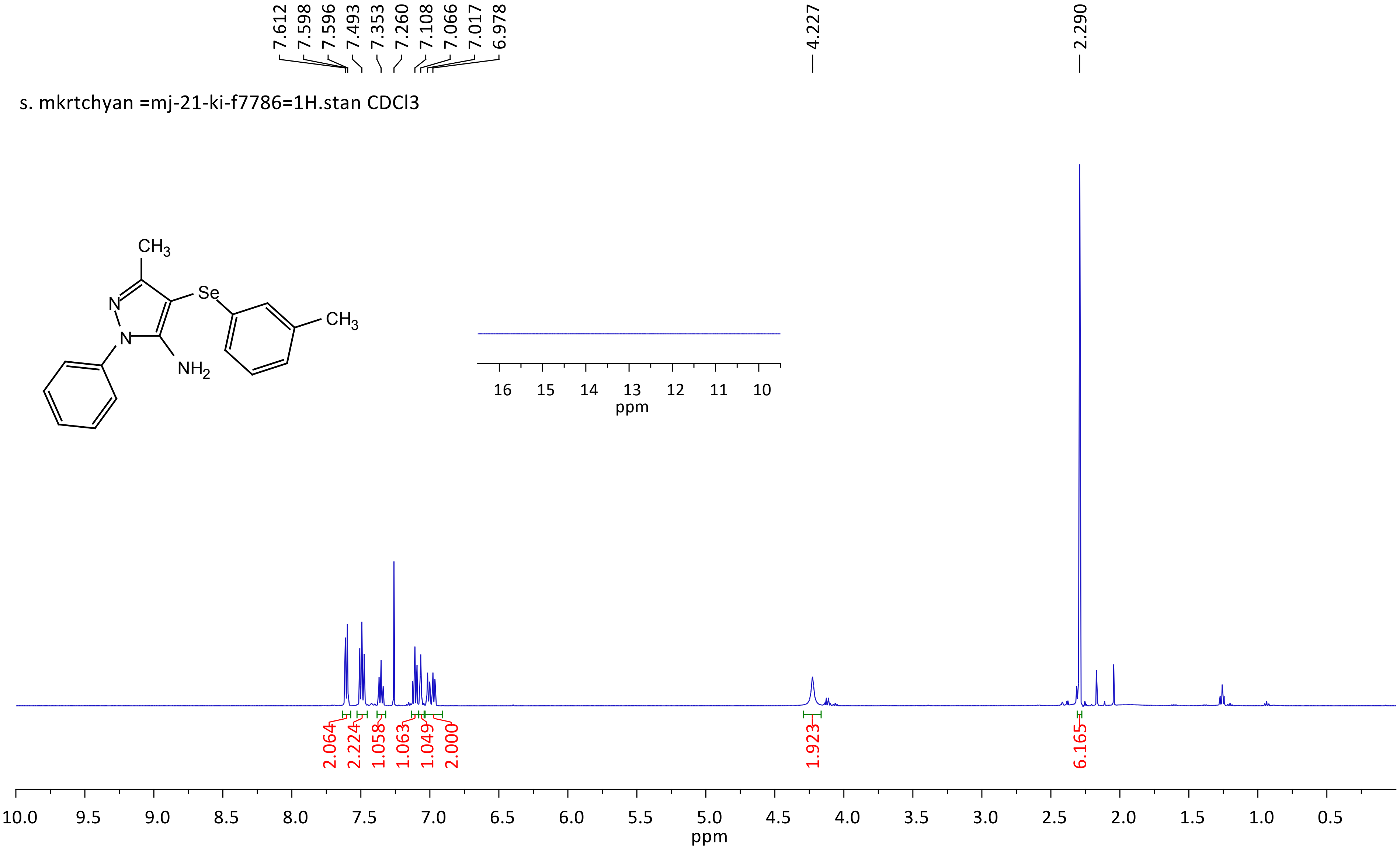

Comment	s. mkrtchyan =mj-21-ki-f7786=1H.stan CDCl3
Number of Scans	24
Spectrometer Frequency	500.13
Spectral Width	12335.5
Spectral Size	65536

Compound **5a**

s. mkrtchyan =mj-21-ki-f7786=A-13C.stan CDCl3

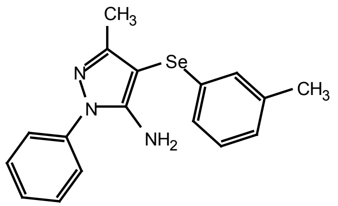

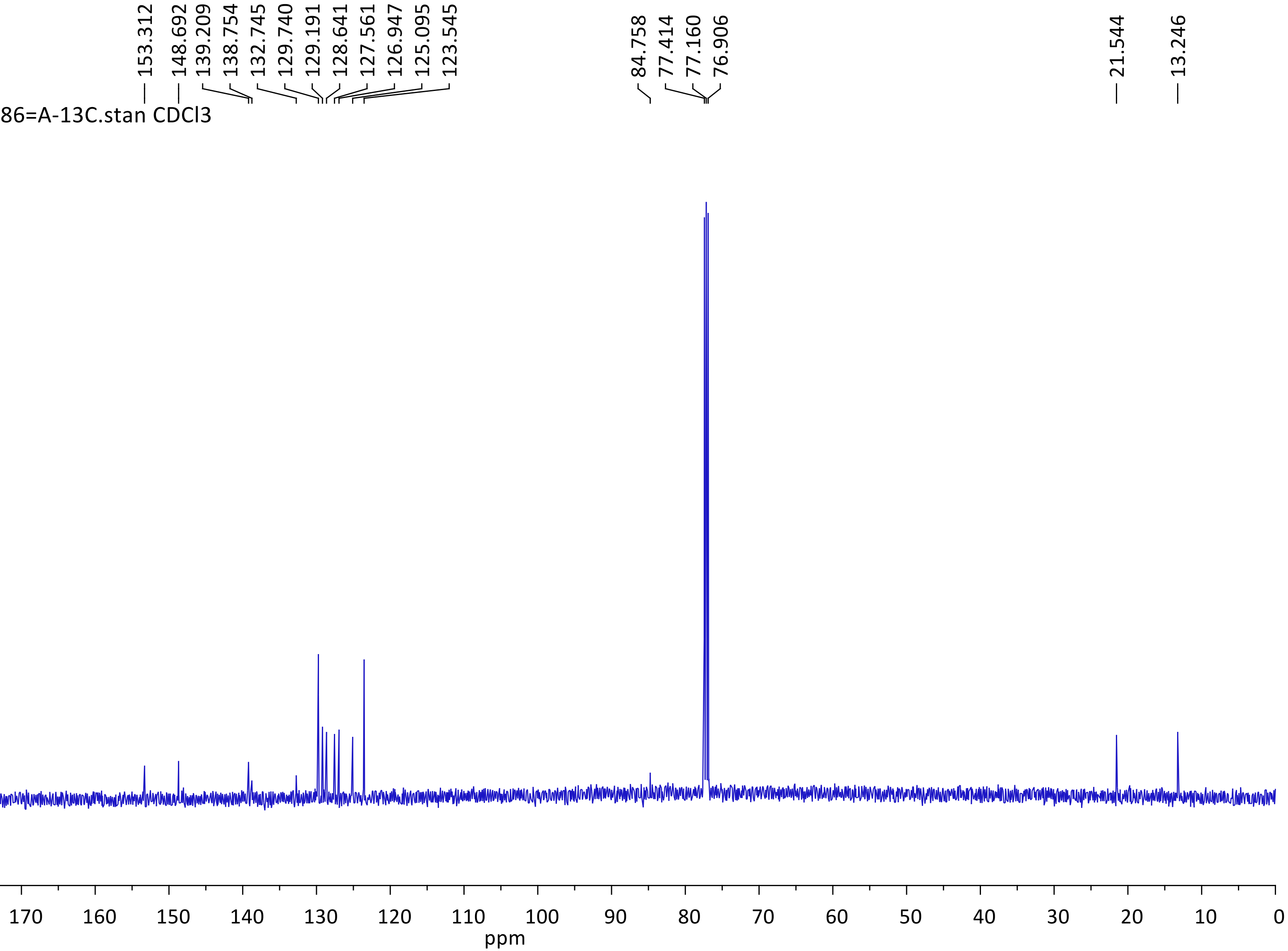

Comment	s. mkrtchyan =mj-21-ki-f7786=A-13C.stan CDCl3
Number of Scans	512
Spectrometer Frequency	125.76
Spectral Width	36057.7
Spectral Size	65536

Compound **5b**

s. mkrtchyan =mj-1-k1f2028=1H.stan CDCl3

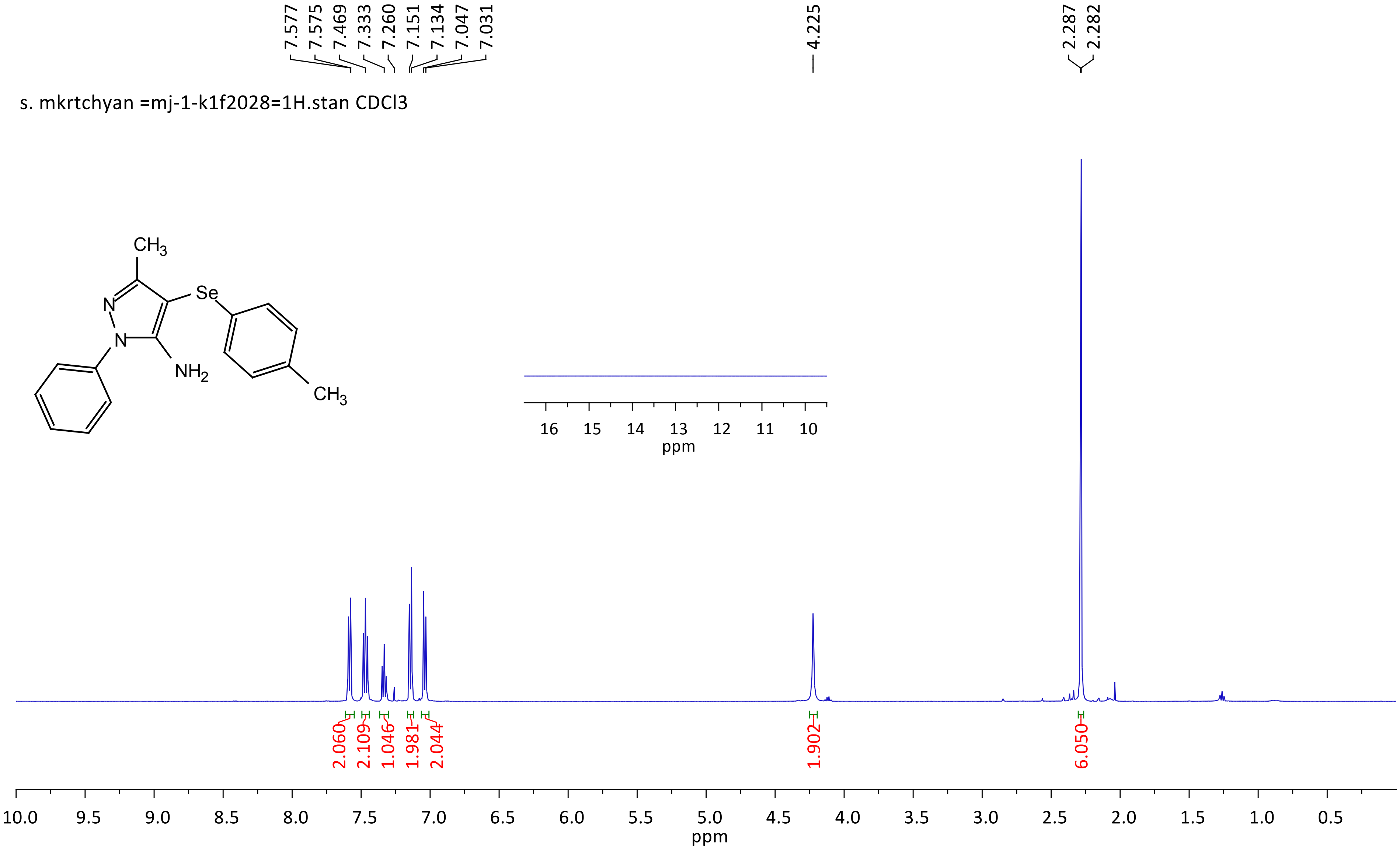

Comment	s. mkrtchyan =mj-1-k1f2028=1H.stan CDCl3
Number of Scans	32
Spectrometer Frequency	500.13
Spectral Width	12335.5
Spectral Size	65536

Compound **5b**

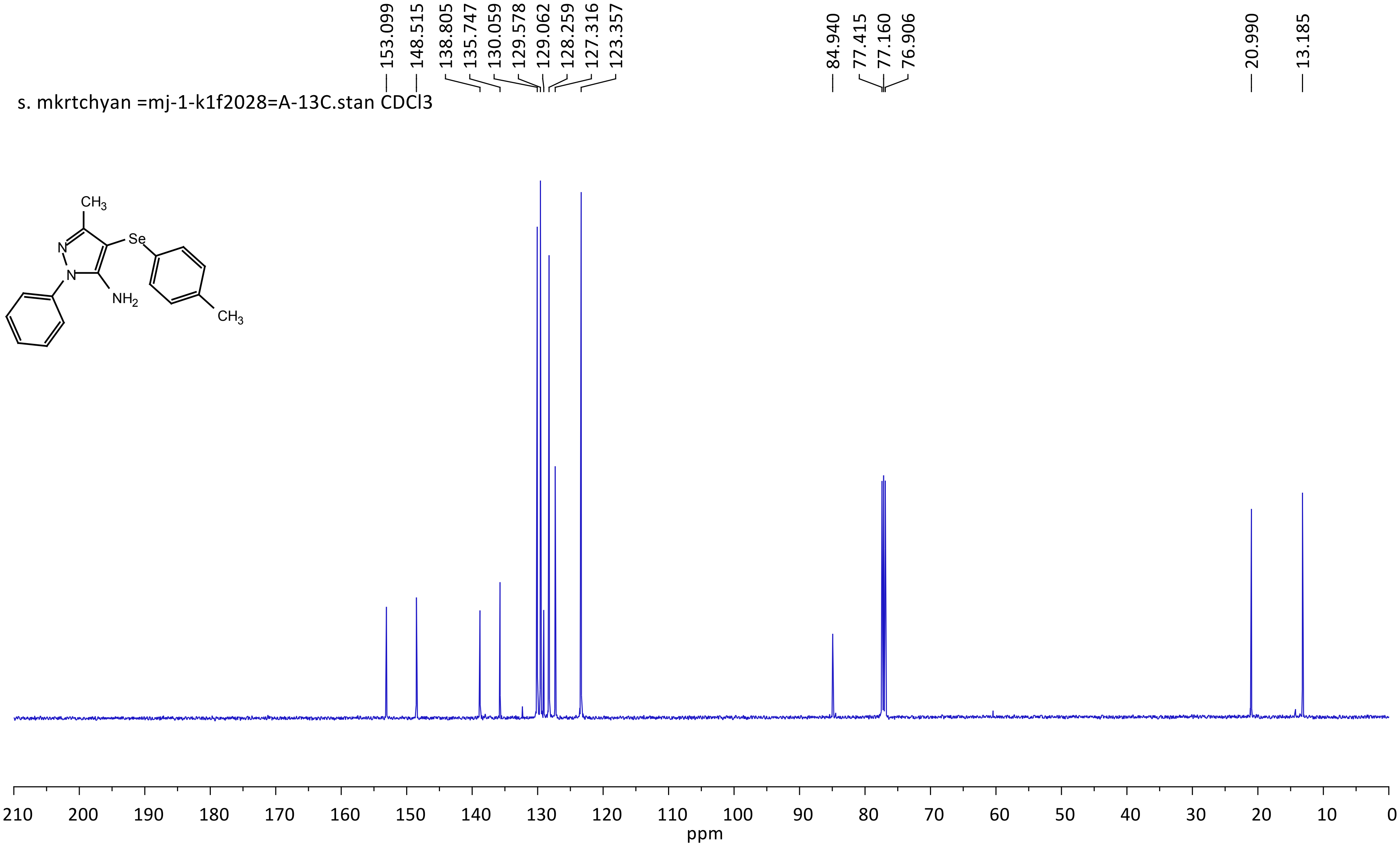

Comment	s. mkrtchyan =mj-1-k1f2028=A-13C.stan CDCl3
Number of Scans	1024
Spectrometer Frequency	125.76
Spectral Width	36057.7
Spectral Size	65536

Compound **5c**

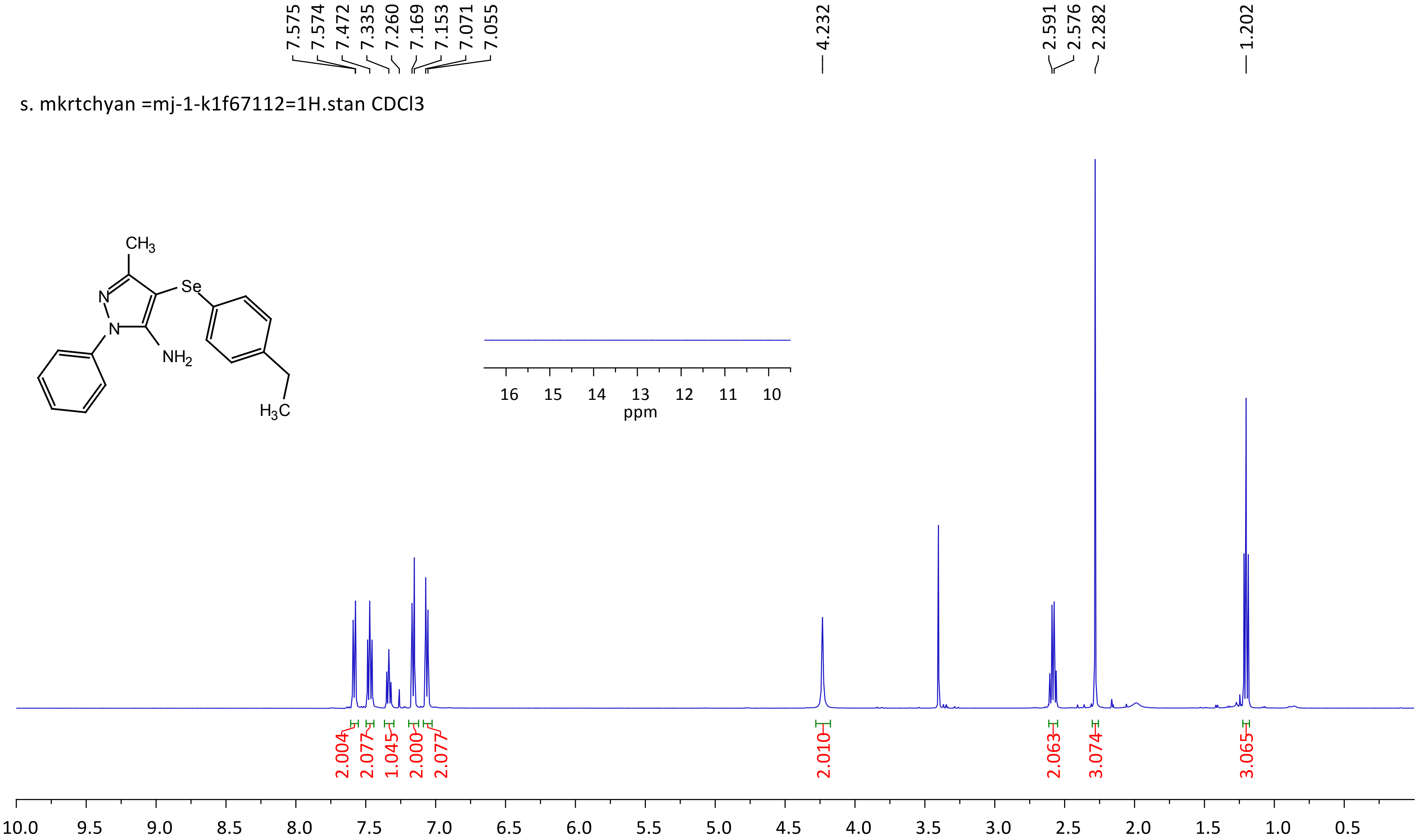

Parameter	Value
Comment	s. mkrtchyan =mj-1-k1f67112=1H.stan CDCl3
Number of Scans	32
Spectrometer Frequency	500.13
Spectral Width	12335.5
Spectral Size	65536

Compound **5c**

s. mkrtchyan =mj-2-k1f67112=A-13C.stan CDCl3

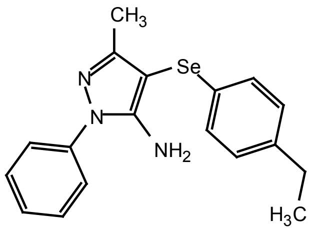

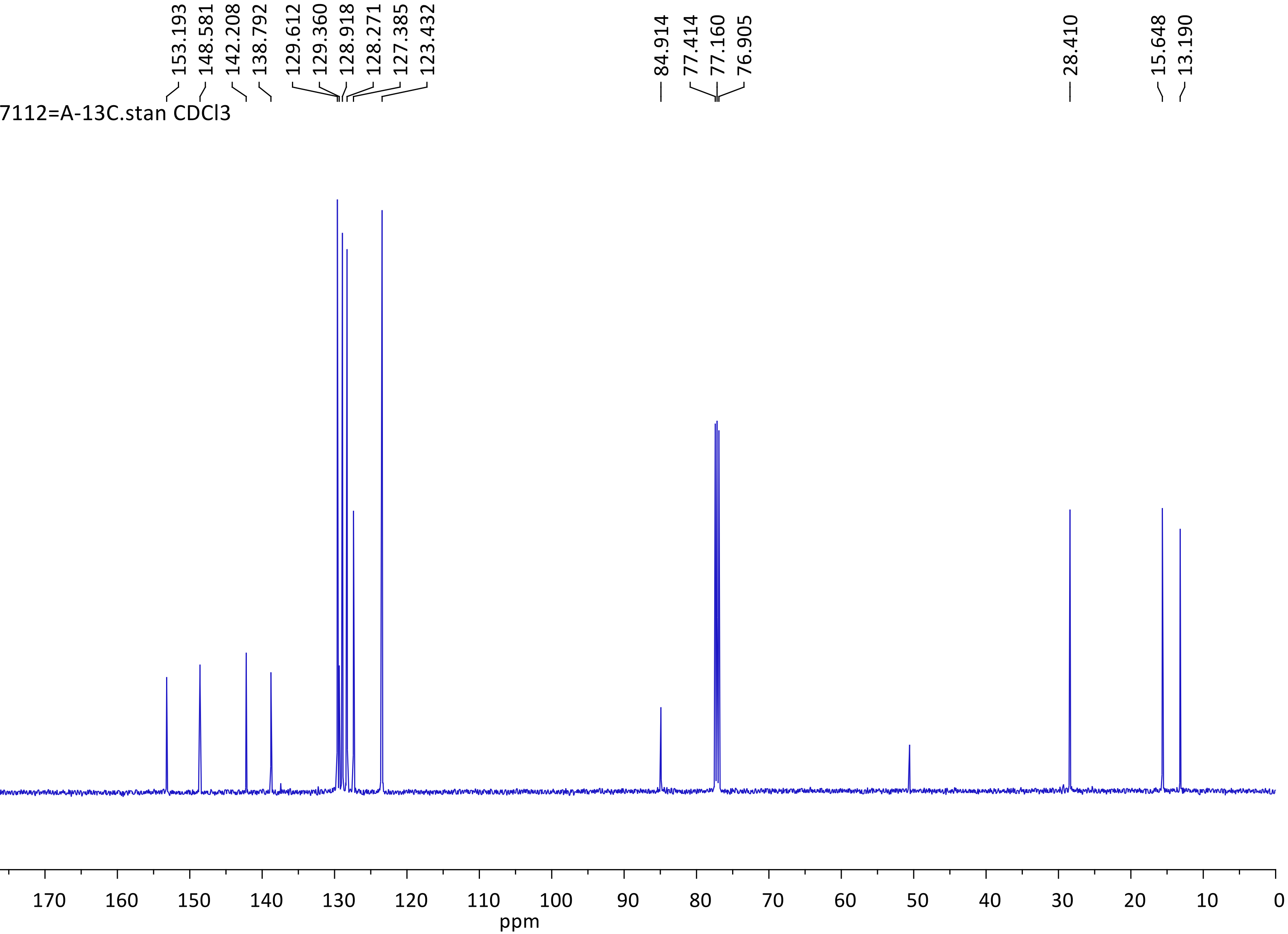

Comment	s. mkrtchyan =mj-2-k1f67112=A-13C.stan CDCl3
Number of Scans	1024
Spectrometer Frequency	125.76
Spectral Width	36057.7
Spectral Size	65536

Compound **5d**

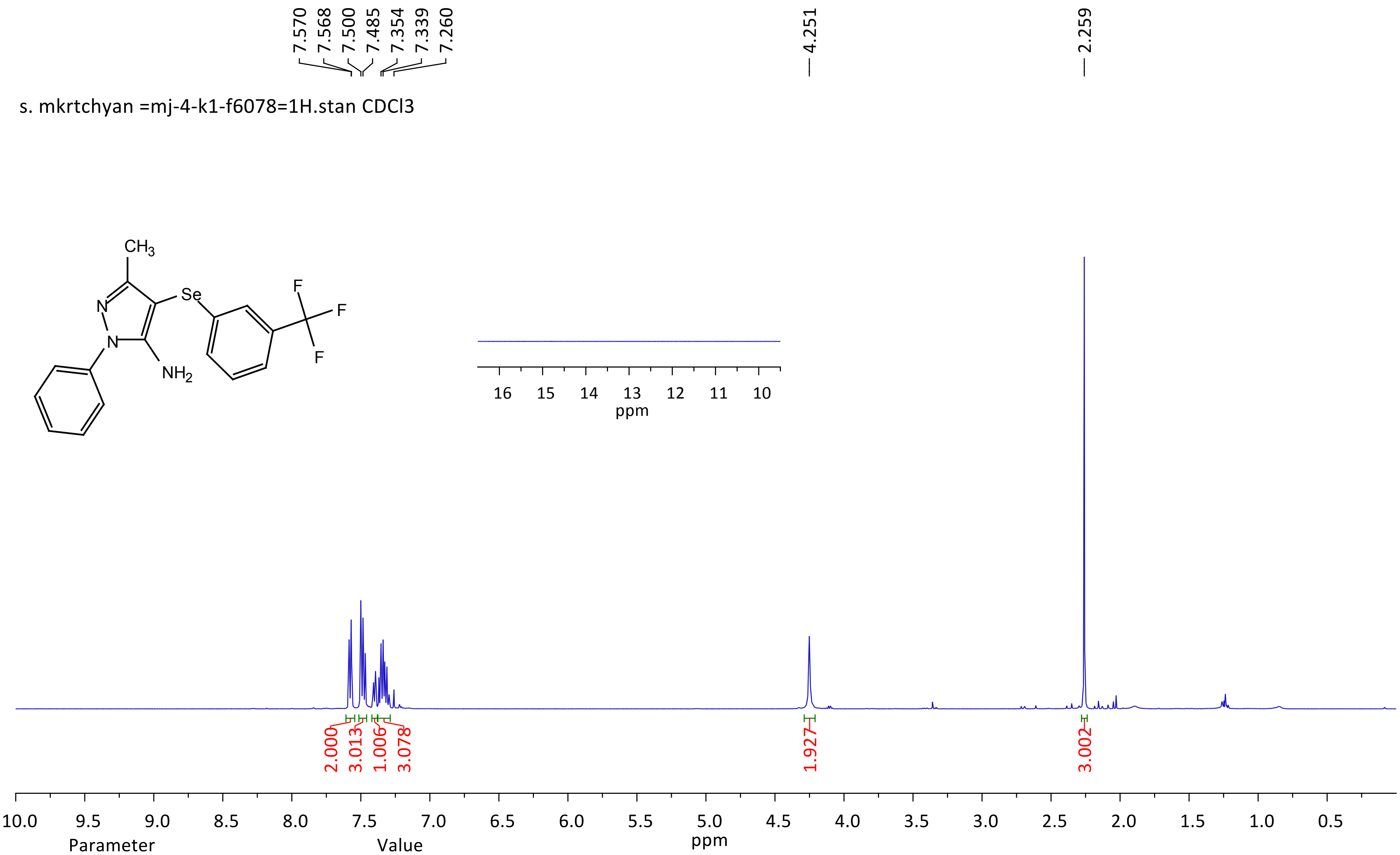

Parameter	Value
Comment	s. mkrtchyan =mj-4-k1-f6078=1H.stan CDCl3
Number of Scans	32
Spectrometer Frequency	500.13
Spectral Width	12335.5
Spectral Size	65536

Compound **5d**

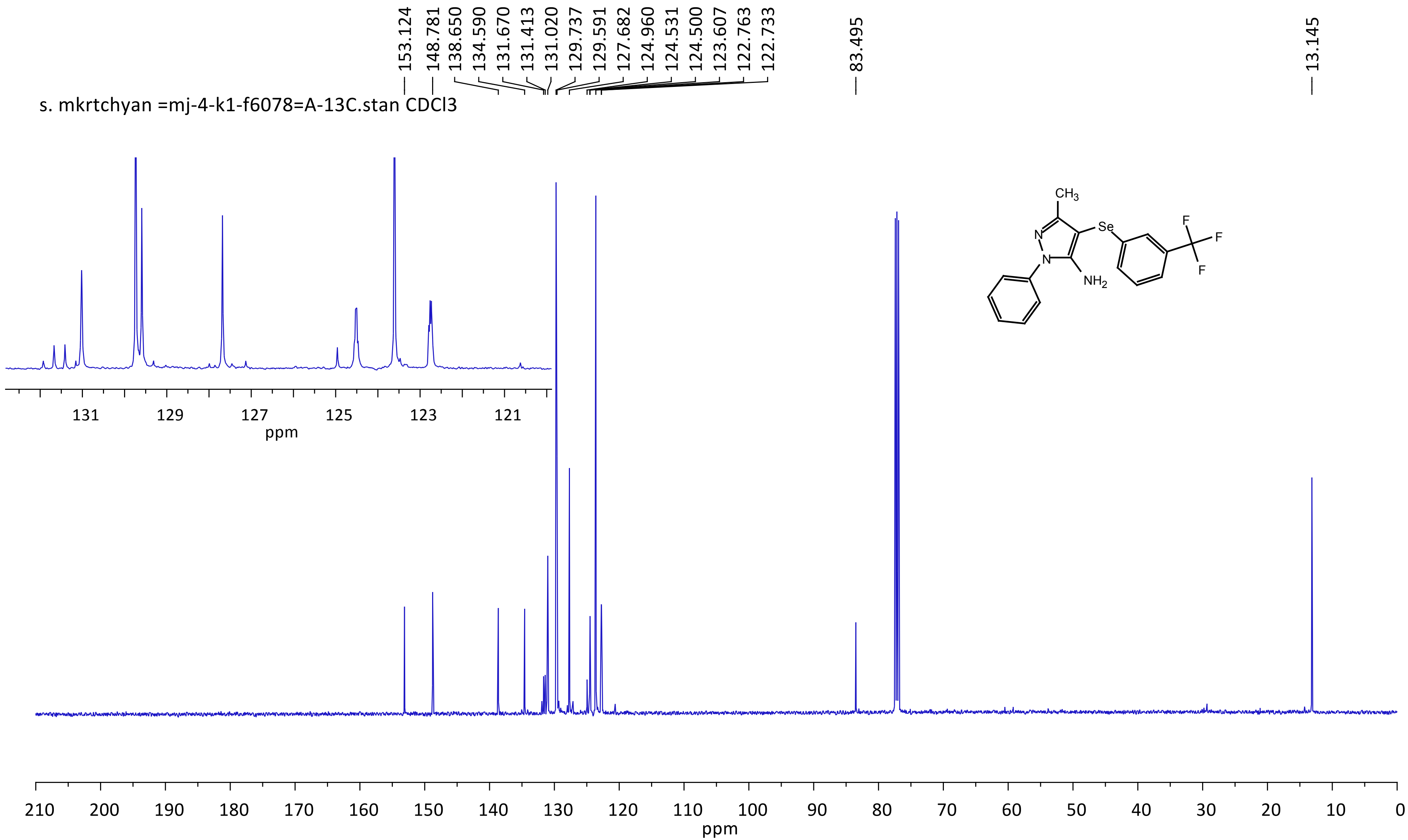

Comment	s. mkrtchyan =mj-4-k1-f6078=A-13C.stan CDCl3
Number of Scans	2560
Spectrometer Frequency	125.76
Spectral Width	36057.7
Spectral Size	65536

Compound **5e**

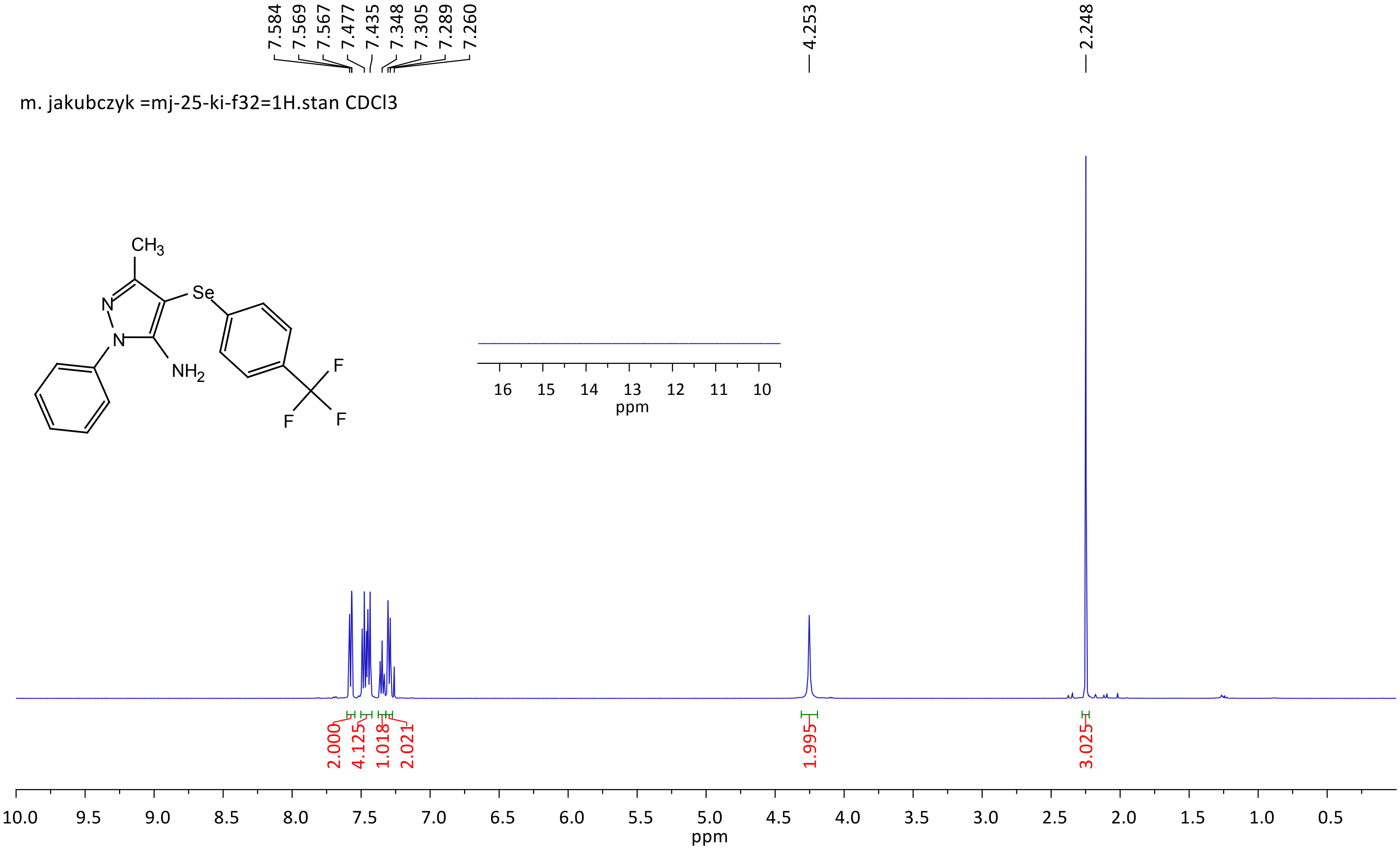

Comment	m. jakubczyk =mj-25-ki-f32=1H.stan CDCl3
Number of Scans	32
Spectrometer Frequency	500.13
Spectral Width	12335.5
Spectral Size	65536

Compound **5e**

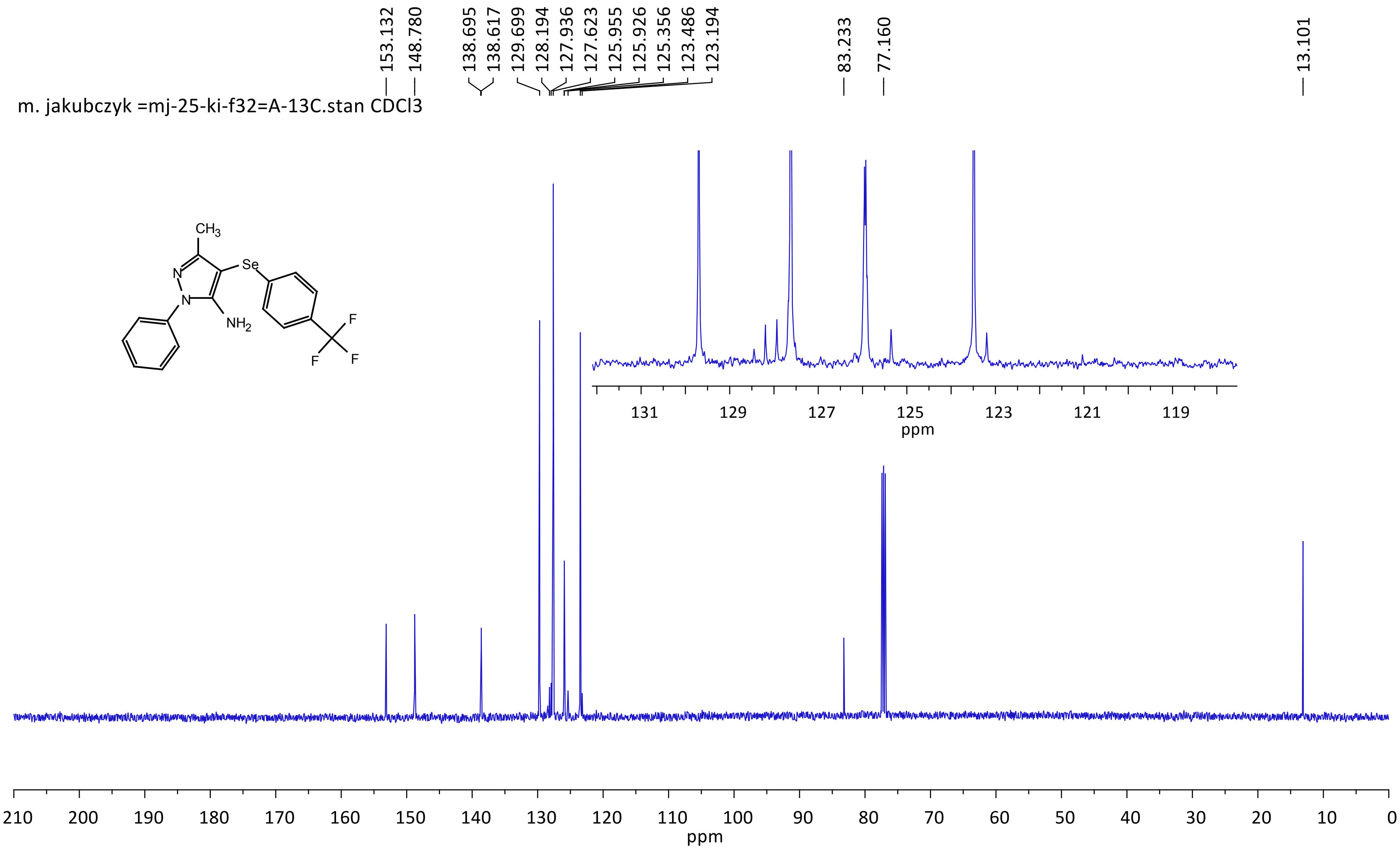

Comment	m. jakubczyk =mj-25-ki-f32=A-13C.stan CDCl3
Number of Scans	256
Spectrometer Frequency	125.76
Spectral Width	36057.7
Spectral Size	65536

Compound **5f**

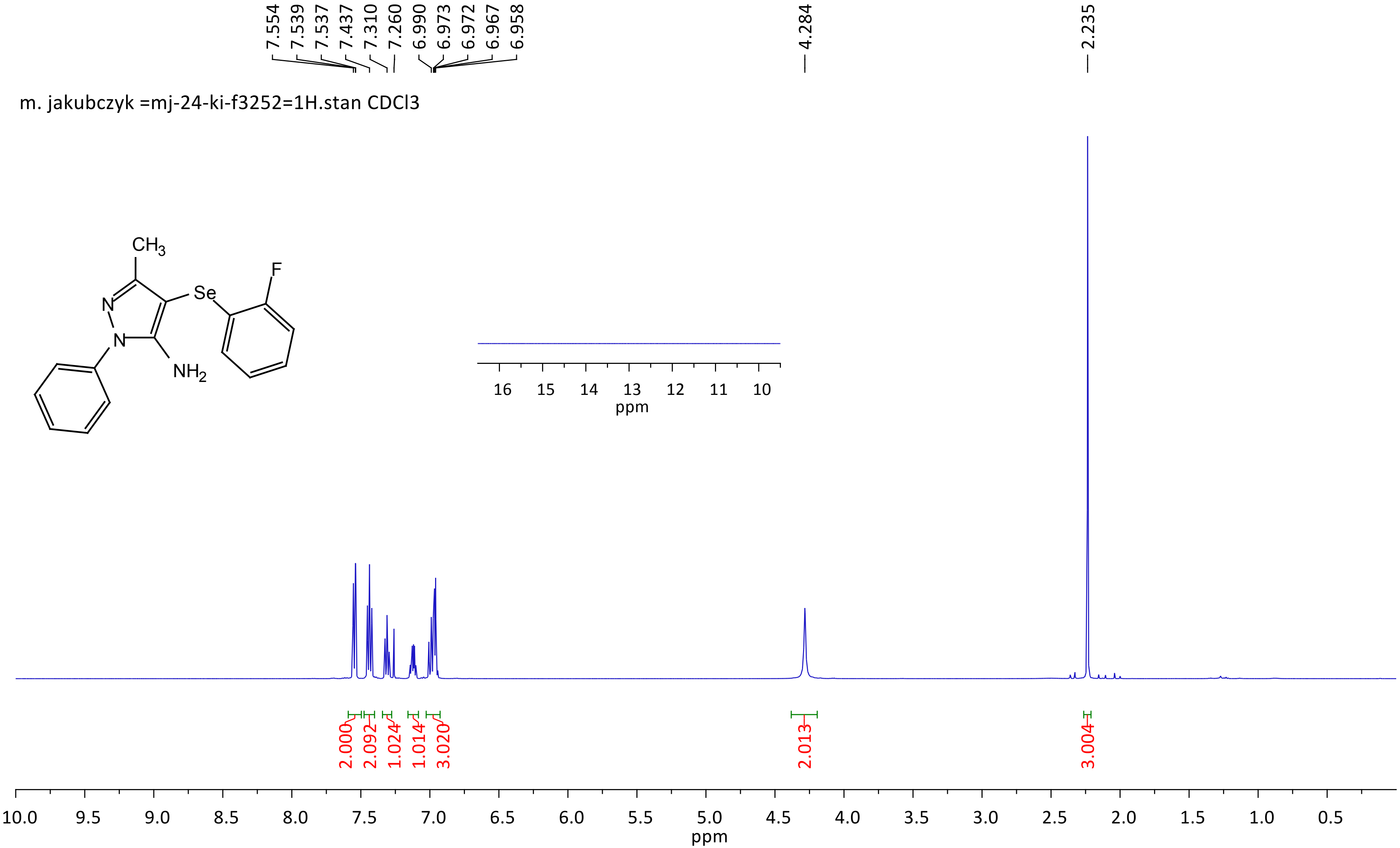

Comment	m. jakubczyk =mj-24-ki-f3252=1H.stan CDCl3
Number of Scans	32
Spectrometer Frequency	500.13
Spectral Width	12335.5
Spectral Size	65536

Compound **5f**

m. jakubczyk =mj-24-kif3252=A-13C.stan CDCl3

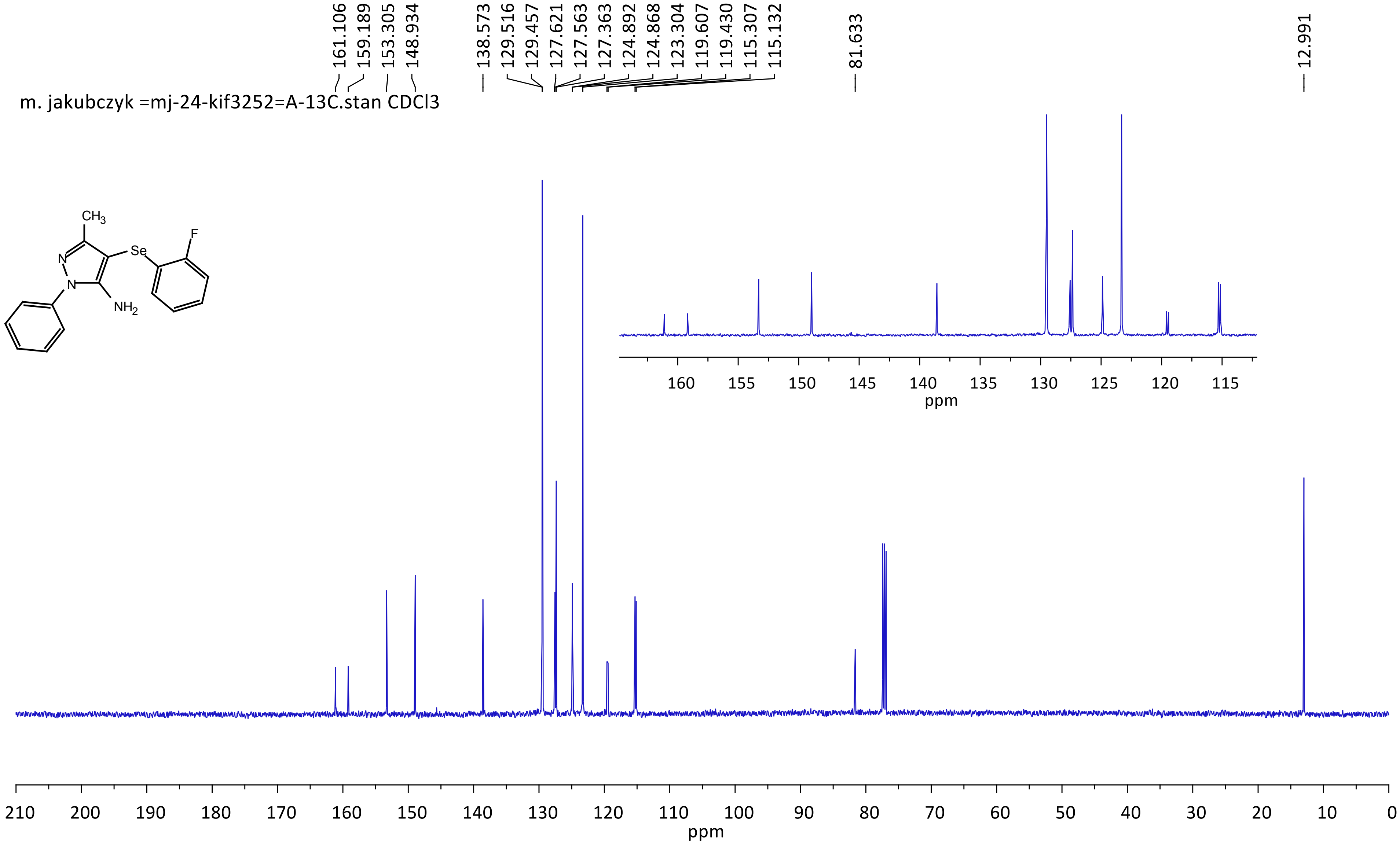

Comment	m. jakubczyk =mj-24-kif3252=A-13C.stan CDCl3
Number of Scans	256
Spectrometer Frequency	125.76
Spectral Width	36057.7
Spectral Size	65536

Compound **5g**

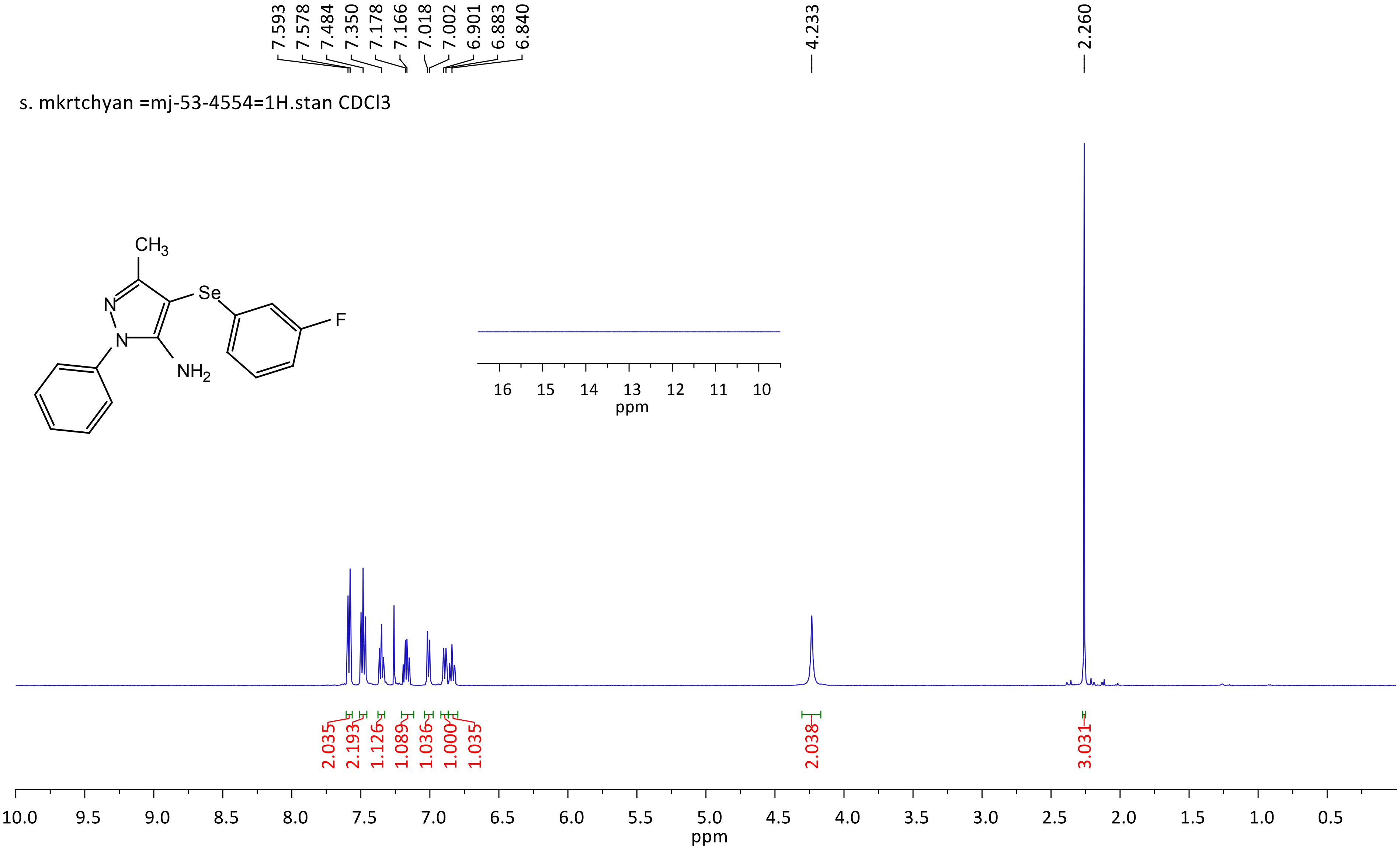

Comment	s. mkrtchyan =mj-53-4554=1H.stan CDCl3
Number of Scans	32
Spectrometer Frequency	500.13
Spectral Width	12335.5
Spectral Size	65536

Compound **5g**

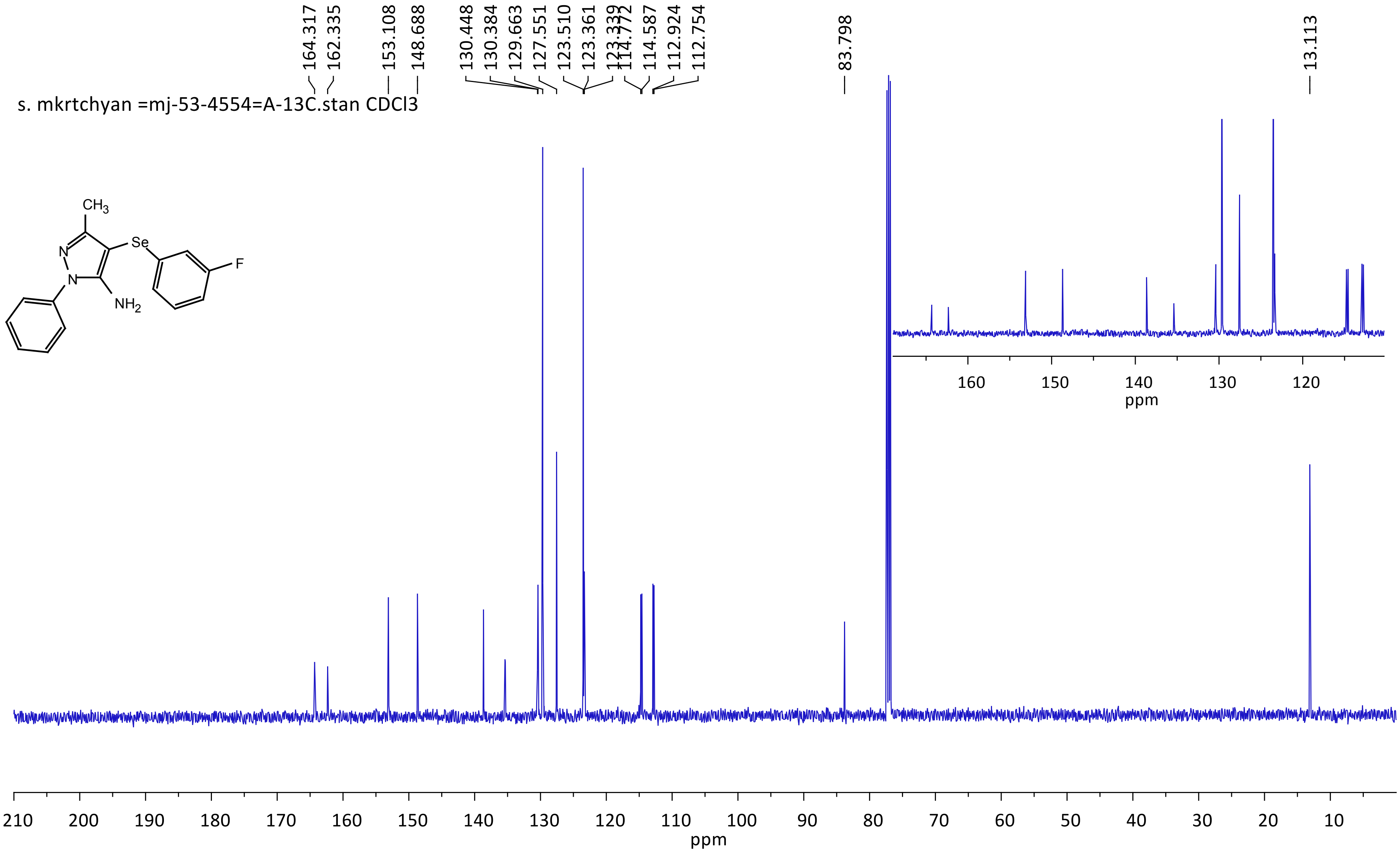

Comment	s. mkrtchyan =mj-53-4554=A-13C.stan CDCl3
Number of Scans	256
Spectrometer Frequency	125.76
Spectral Width	36057.7
Spectral Size	65536

Compound **5h**

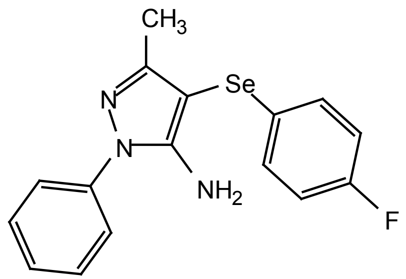

m. jakubczyk =mj-26-ki-f2841=1H.stan CDCl3

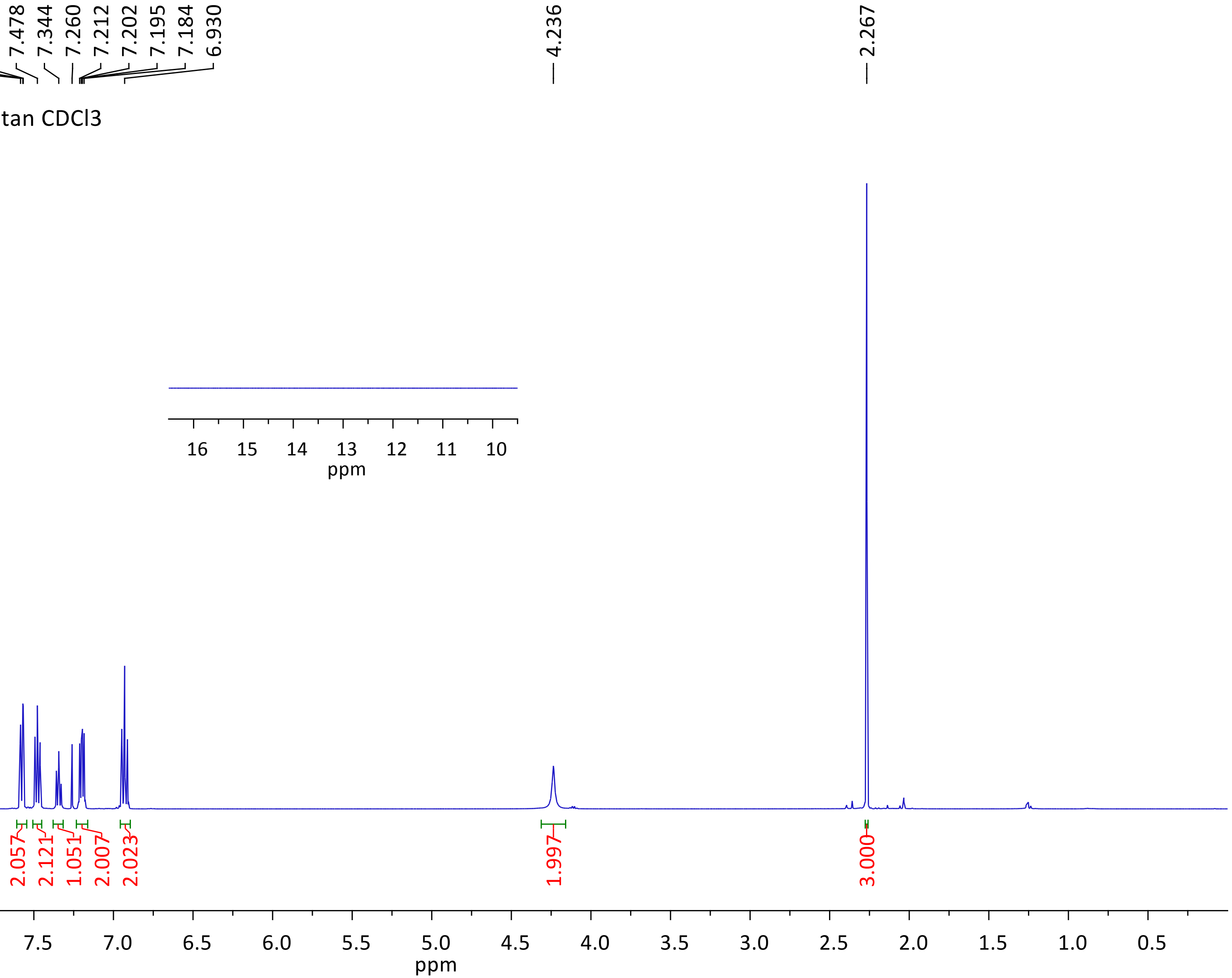

Comment	m. jakubczyk =mj-26-ki-f2841=1H.stan CDCl3
Number of Scans	32
Spectrometer Frequency	500.13
Spectral Width	12335.5
Spectral Size	65536

Compound **5h**

m. jakubczyk =mj-26-ki-f2841=A-13C.stan CDCl3

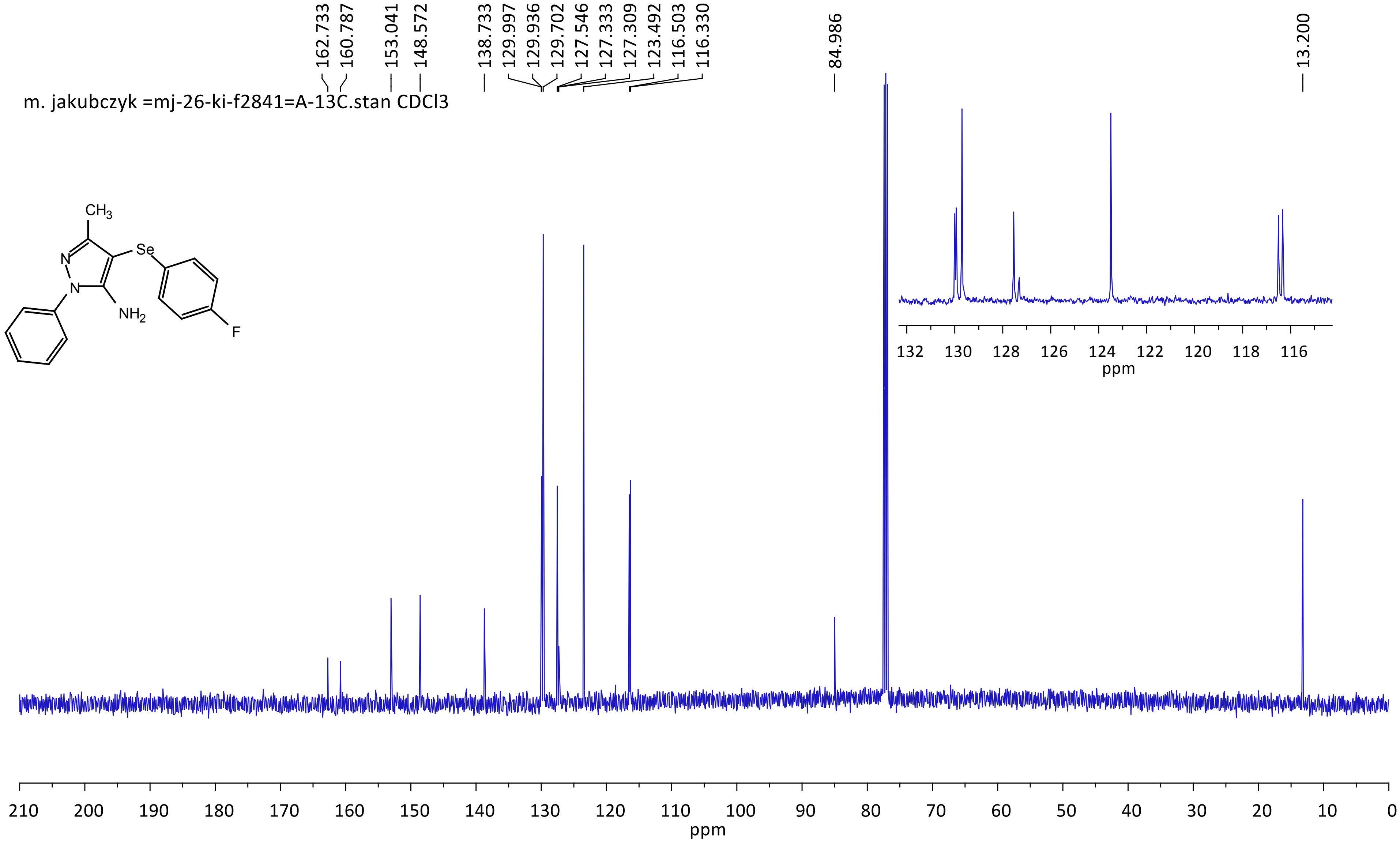

Comment	m. jakubczyk =mj-26-ki-f2841=A-13C.stan CDCl3
Number of Scans	256
Spectrometer Frequency	125.76
Spectral Width	36057.7
Spectral Size	65536

Compound **5i**

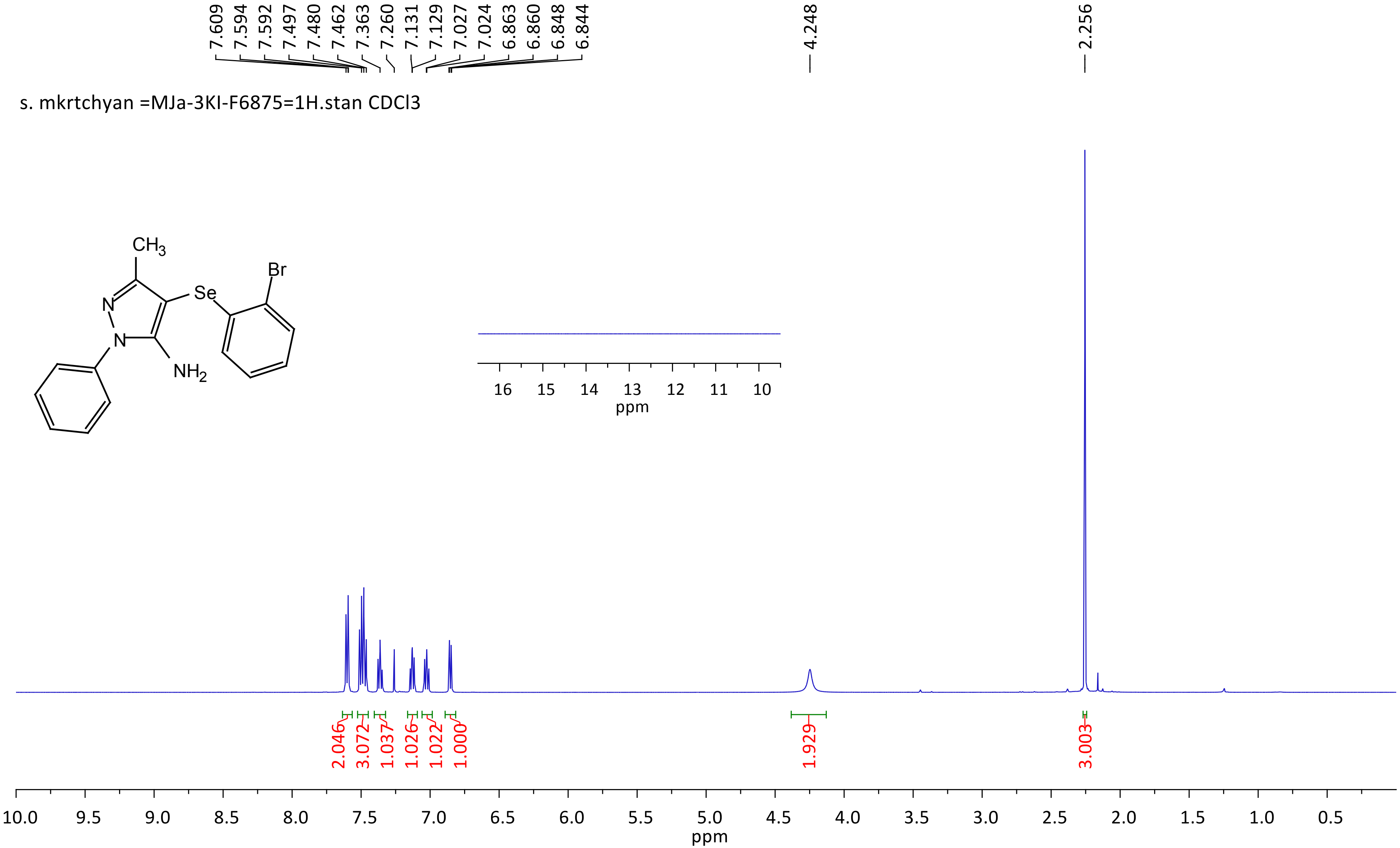

Comment	s. mkrtchyan =MJa-3KI-F6875=1H.stan CDCl3
Number of Scans	32
Spectrometer Frequency	500.13
Spectral Width	12335.5
Spectral Size	65536

Compound **5i**

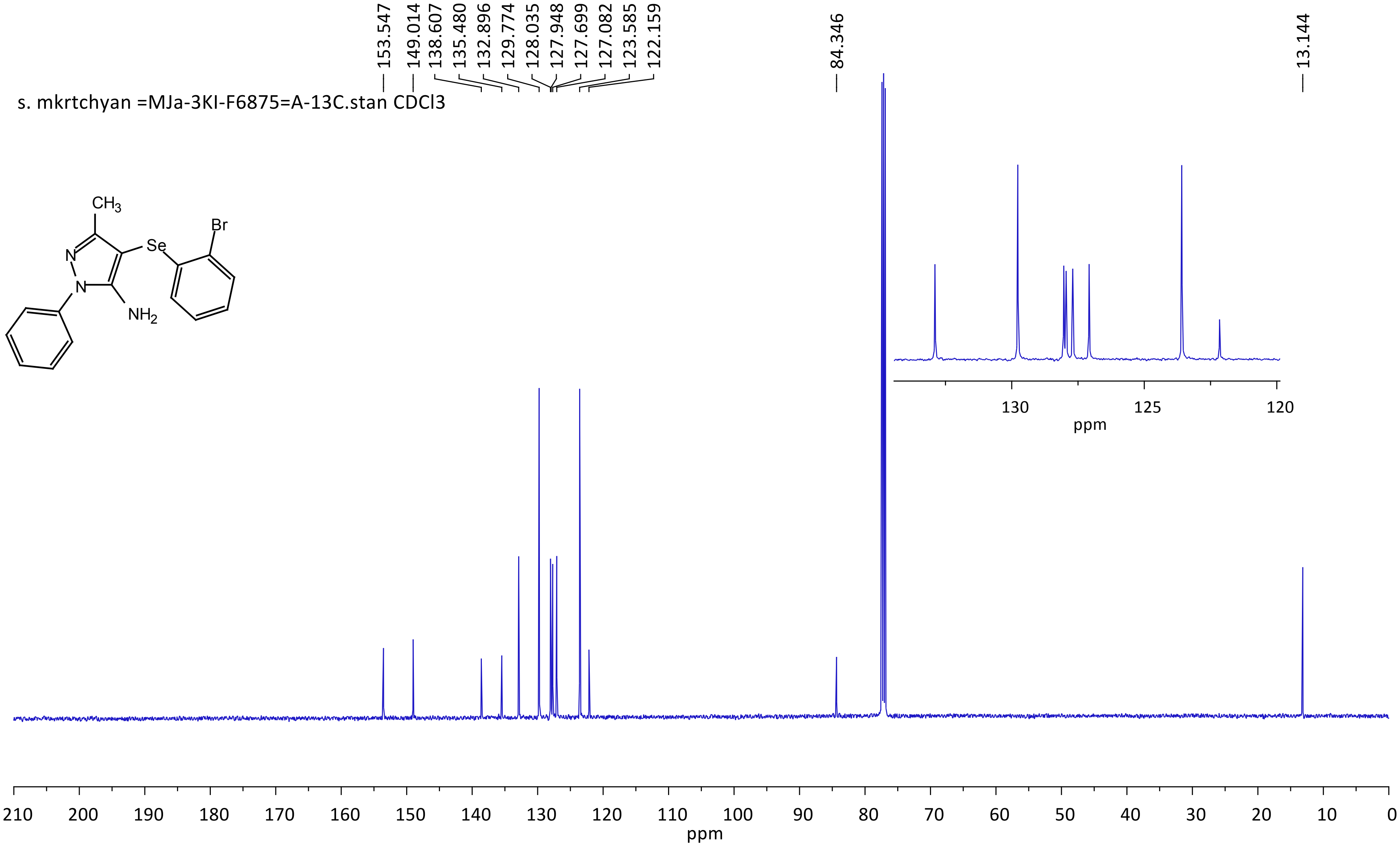

Comment	s. mkrtchyan =MJa-3KI-F6875=A-13C.stan CDCl3
Number of Scans	3072
Spectrometer Frequency	125.76
Spectral Width	36057.7
Spectral Size	65536

Compound **5j**

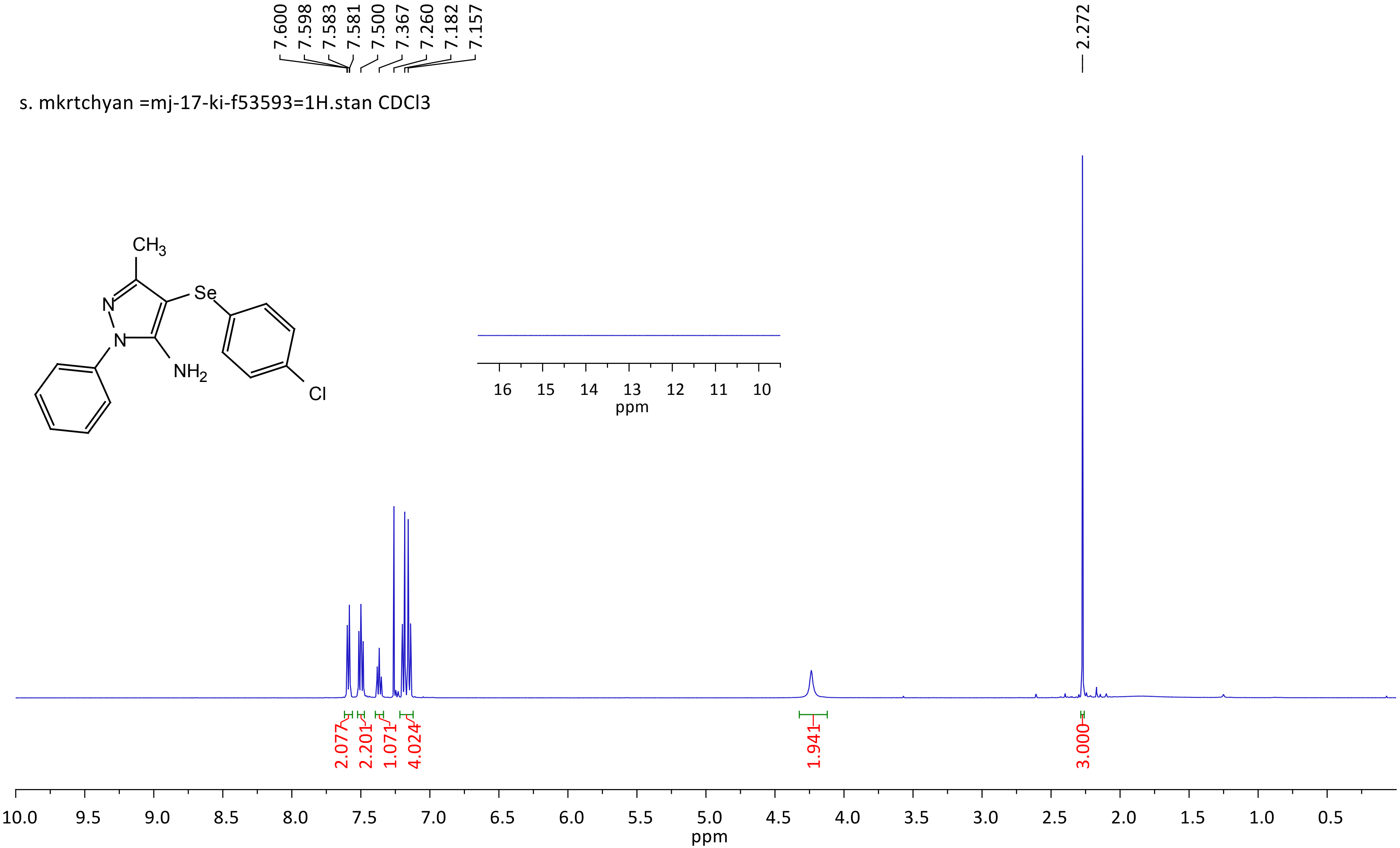

Comment	s. mkrtchyan =mj-17-ki-f53593=1H.stan CDCl3
Number of Scans	24
Spectrometer Frequency	500.13
Spectral Width	12335.5
Spectral Size	65536

Compound **5j**

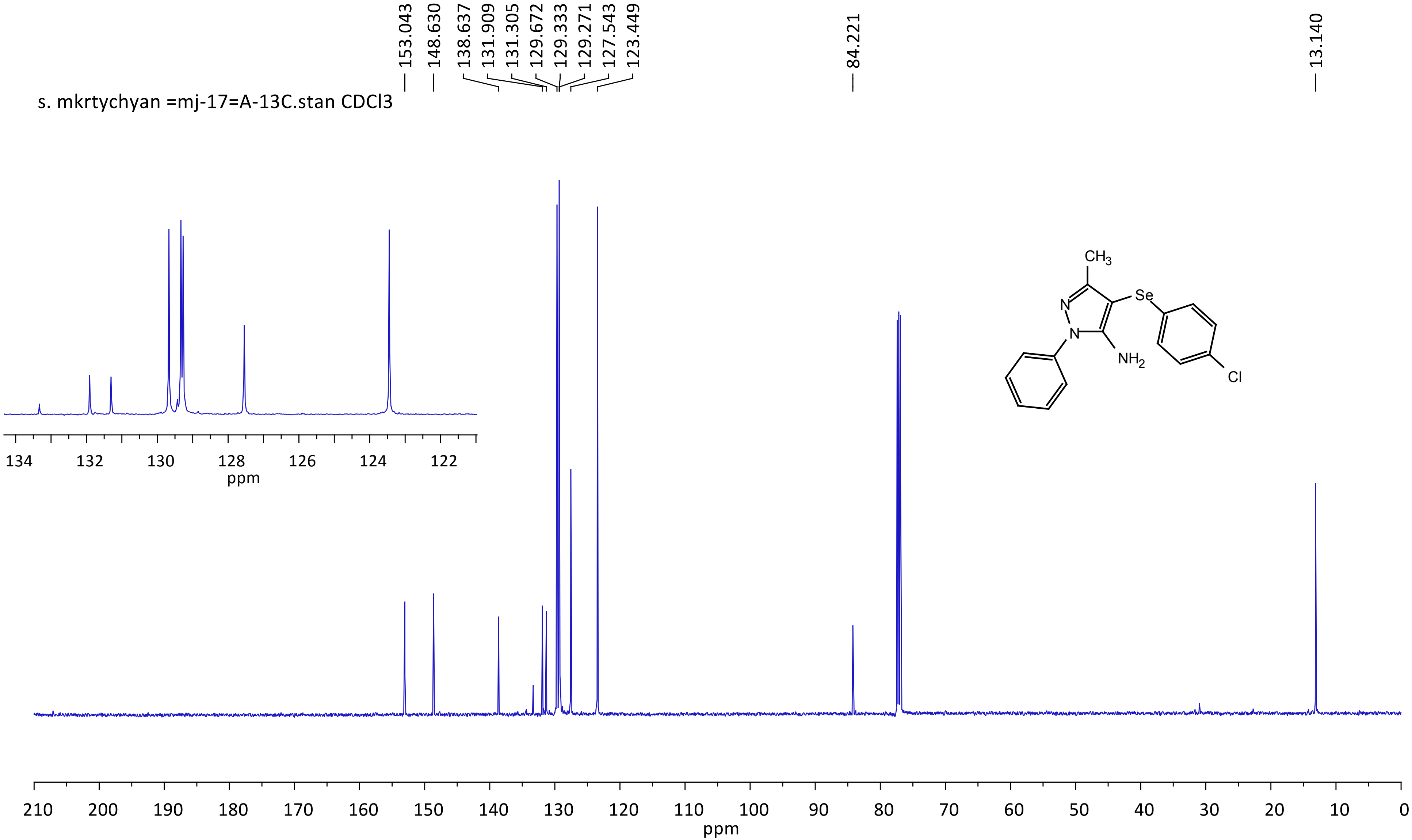

Comment	s. mkrtychyan =mj-17=A-13C.stan CDCl3
Number of Scans	1744
Spectrometer Frequency	125.76
Spectral Width	36057.7
Spectral Size	65536

Compound **5k**

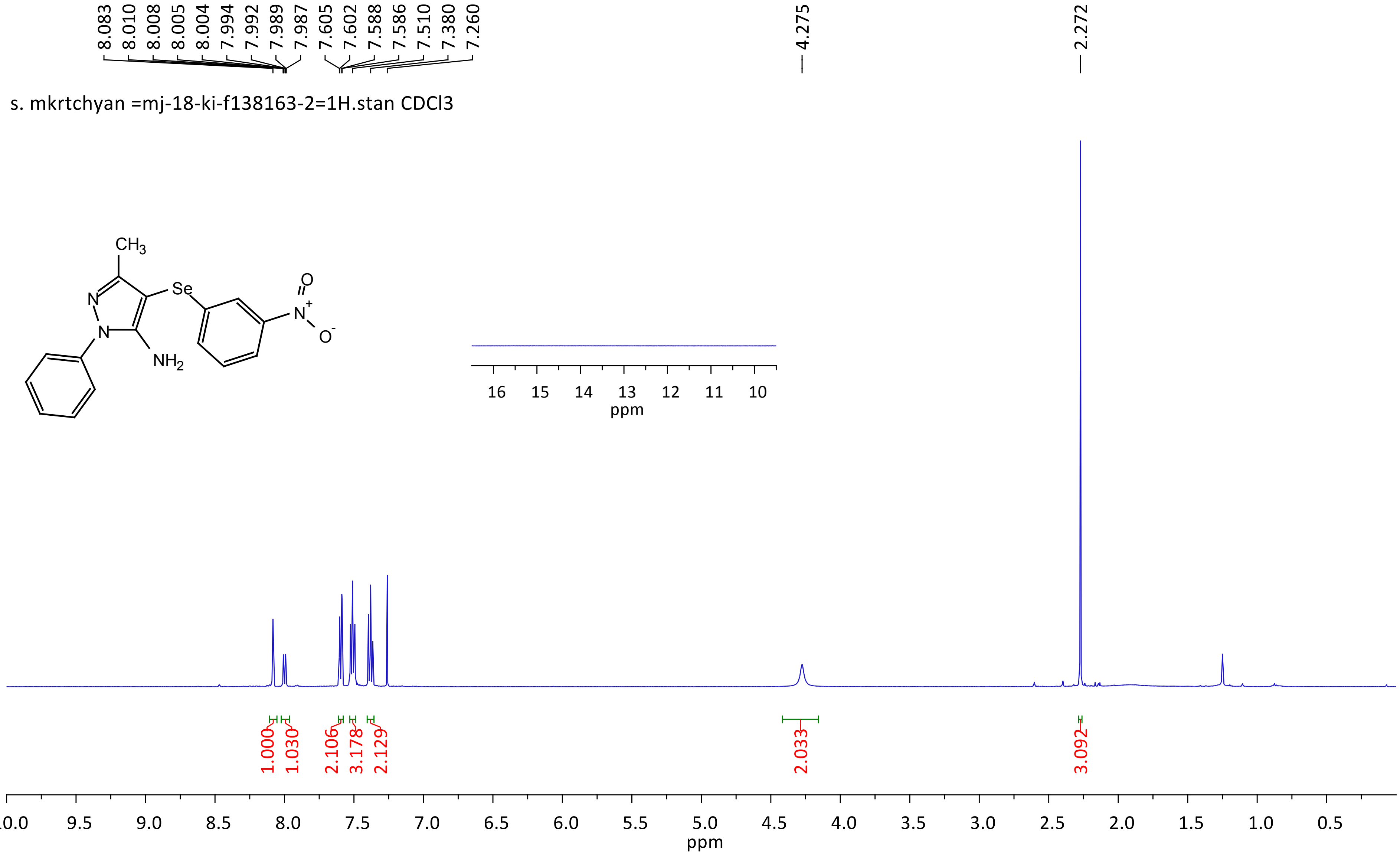

Comment	s. mkrtchyan =mj-18-ki-f138163-2=1H.stan CDCl3
Number of Scans	24
Spectrometer Frequency	500.13
Spectral Width	12335.5
Spectral Size	65536

Compound **5k**

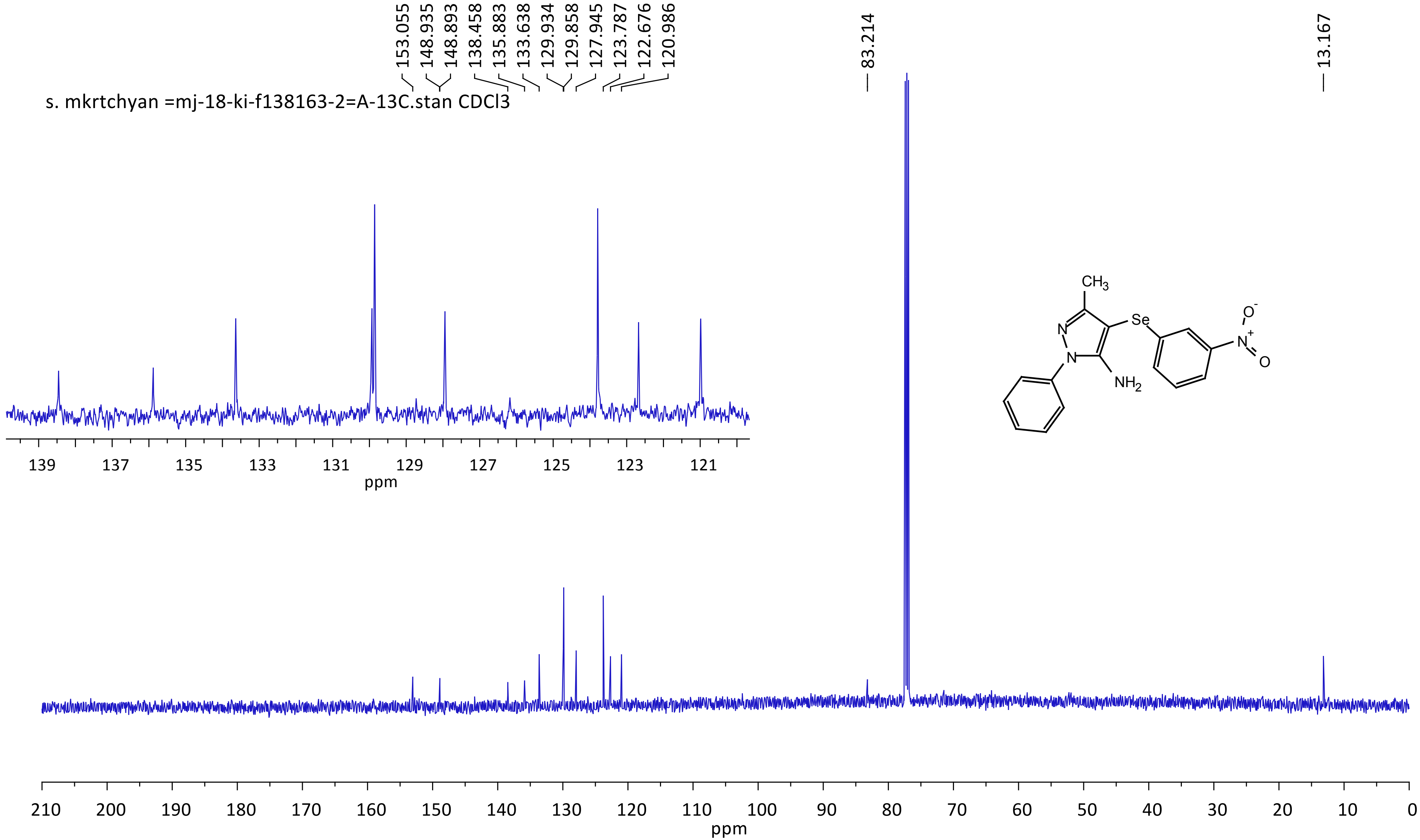

Comment	s. mkrtchyan =mj-18-ki-f138163-2=A-13C.stan CDCl3
Number of Scans	512
Spectrometer Frequency	125.76
Spectral Width	36057.7
Spectral Size	65536

Compound **5l**

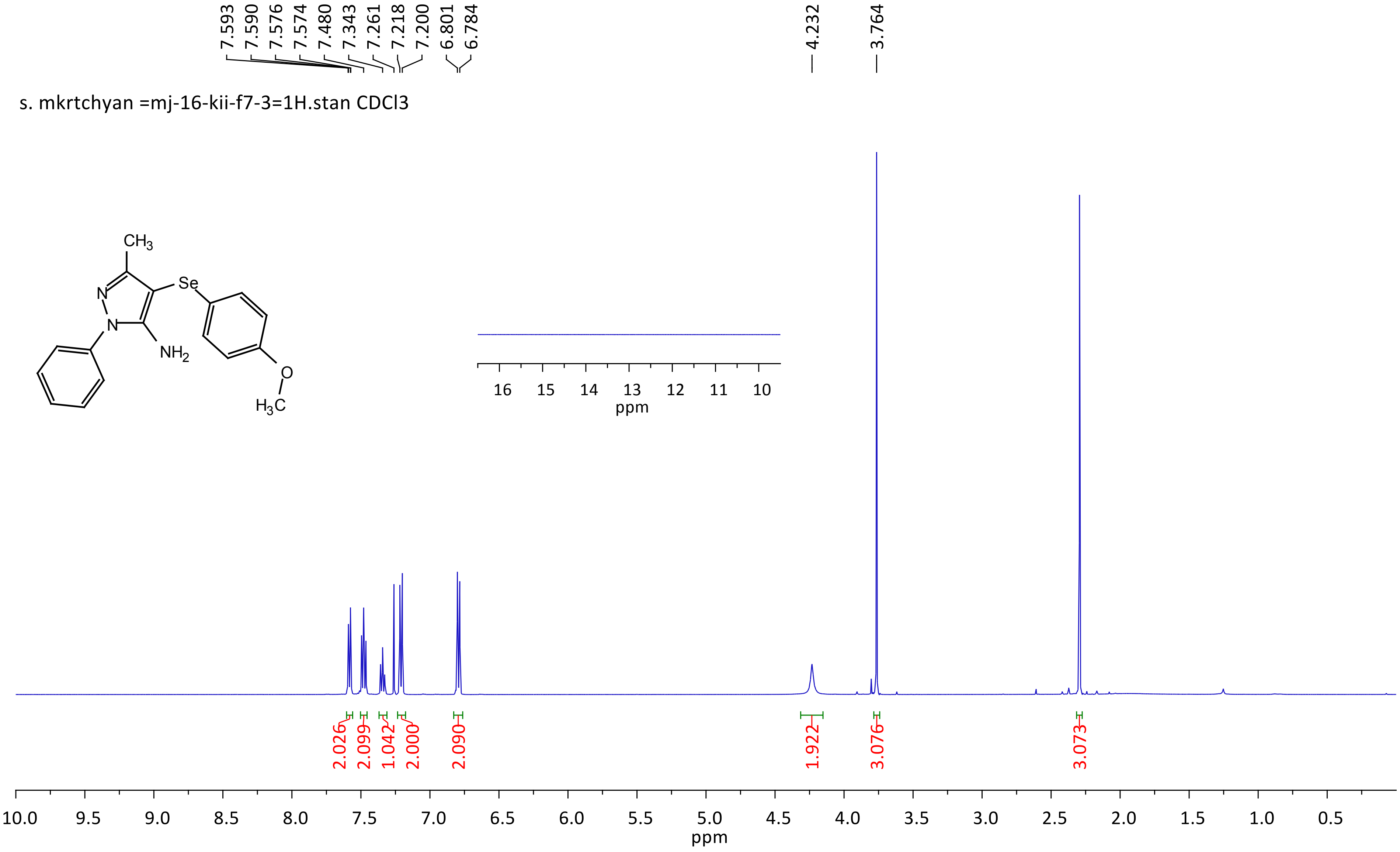

Comment s. mkrtchyan =mj-16-kii-f7-3=1H.stan CDCl3

Number of Scans 24

Spectrometer Frequency 500.13

Spectral Width 12336.0

Spectral Size 65536

Compound **5l**

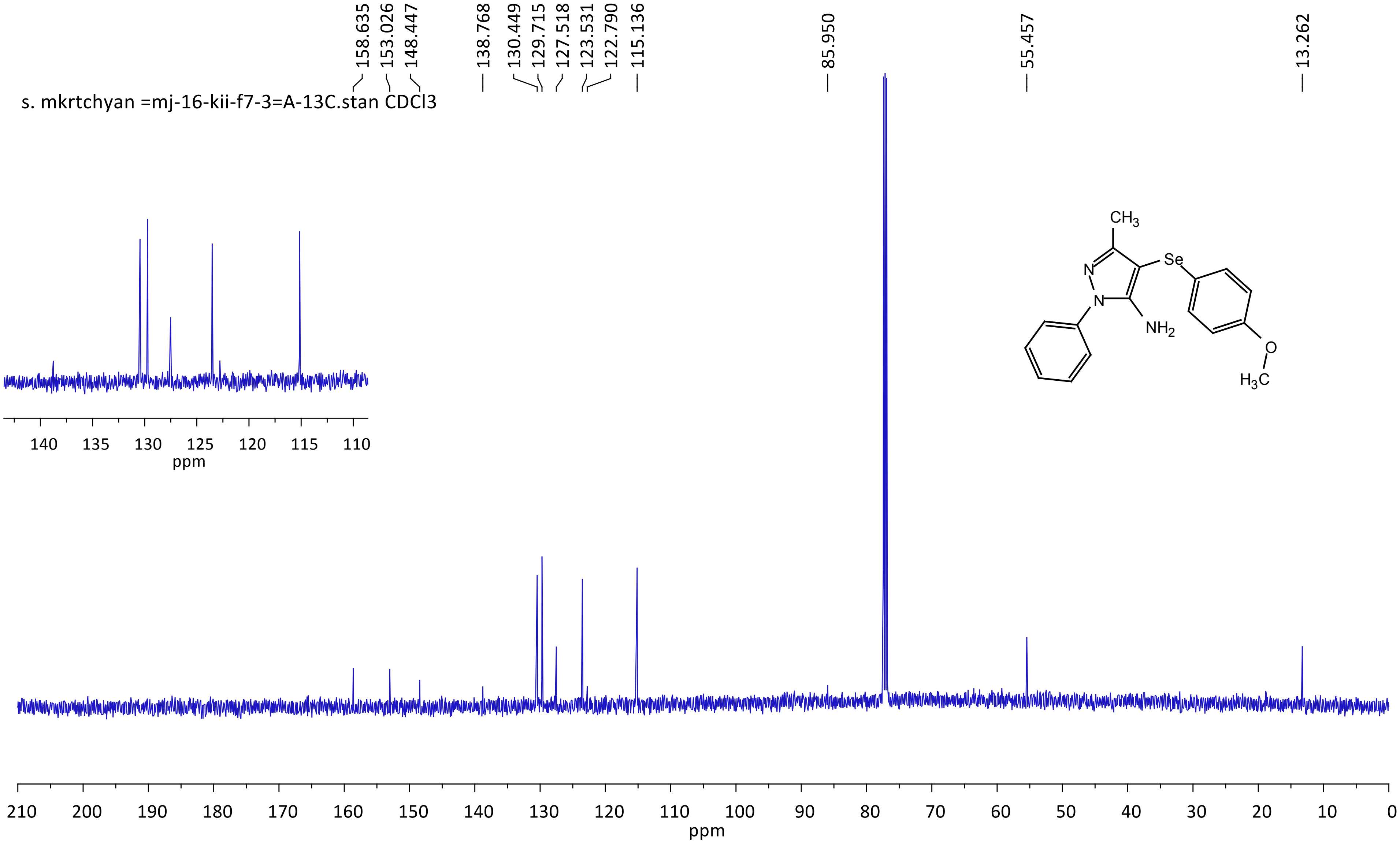

Comment	s. mkrtchyan =mj-16-kii-f7-3=A-13C.stan CDCl3
Number of Scans	512
Spectrometer Frequency	125.76
Spectral Width	36057.7
Spectral Size	65536

Compound **5m**

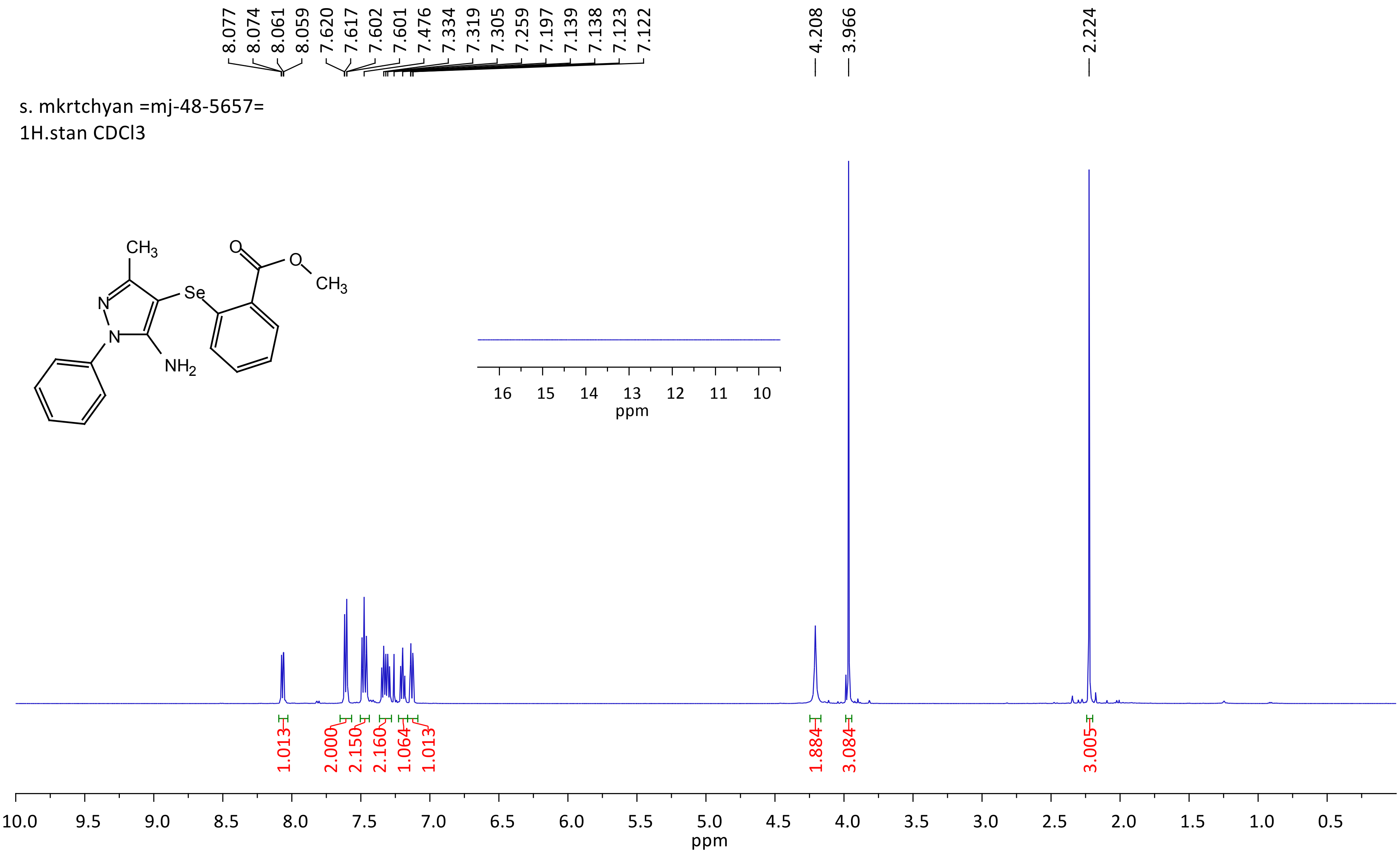

Comment	s. mkrtchyan =mj-48-5657= 1H.stan CDCl3
Number of Scans	24
Spectrometer Frequency	500.13
Spectral Width	12335.5
[illegible]	[illegible]

Compound **5m**

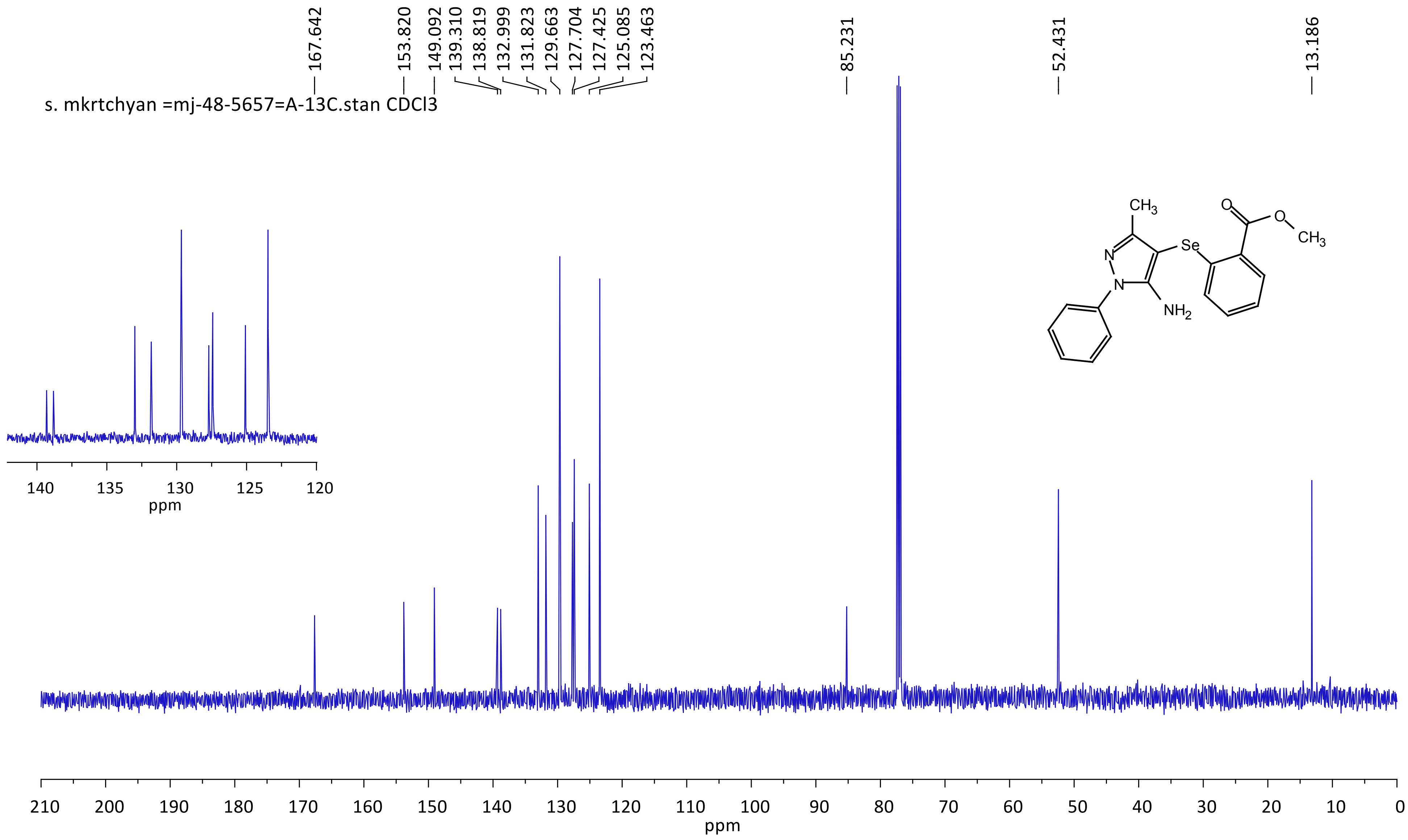

Comment	s. mkrtchyan =mj-48-5657=A-13C.stan CDCl3
Number of Scans	128
Spectrometer Frequency	125.76
Spectral Width	36057.7
Spectral Size	65536

Compound **6**

s. mkrtchyan =mj-143-2935=1H.stan CDCl3

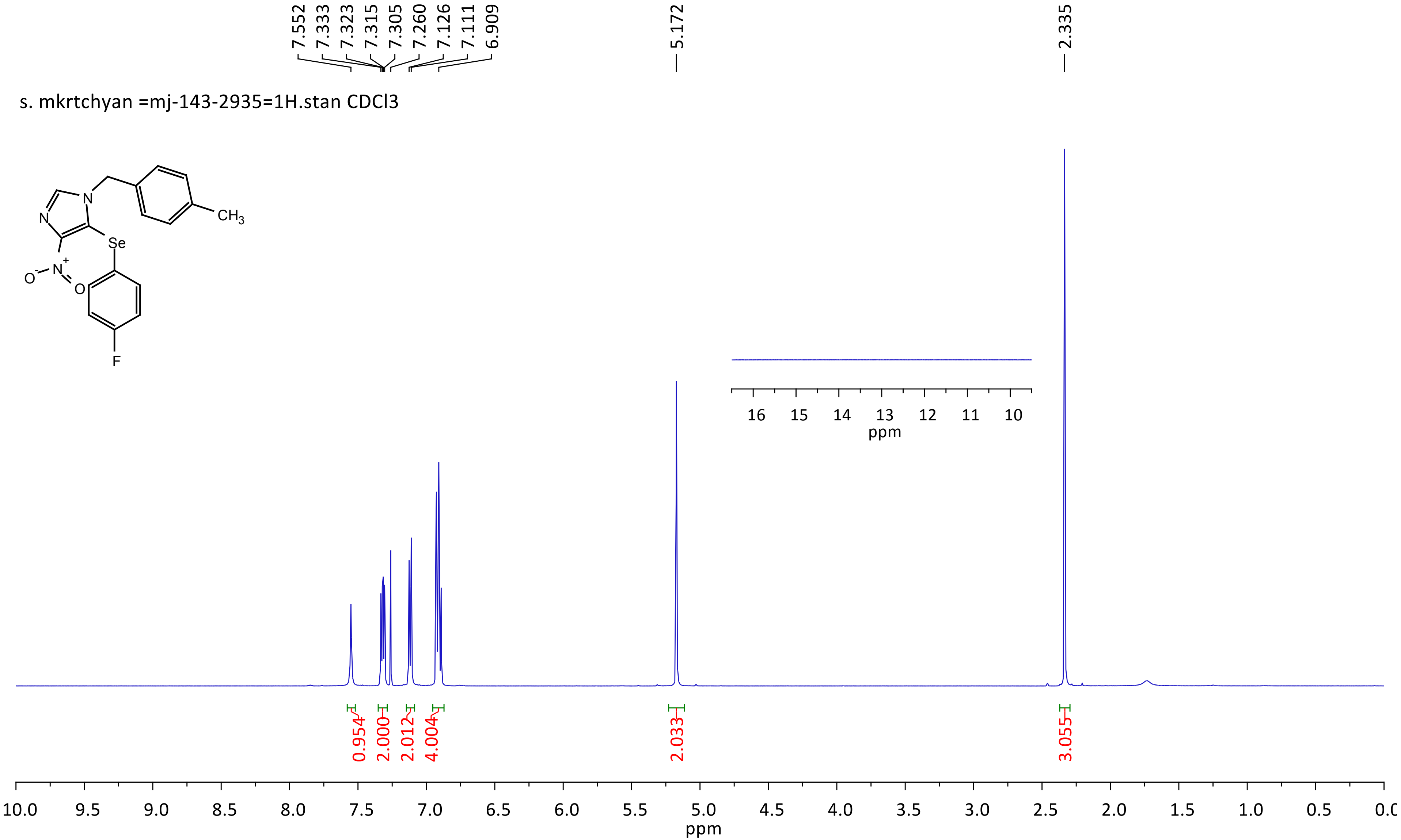

Comment	s. mkrtchyan =mj-143-2935=1H.stan CDCl3
Number of Scans	24
Spectrometer Frequency	500.13
Spectral Width	12335.5
Spectral Size	65536

Compound **6**

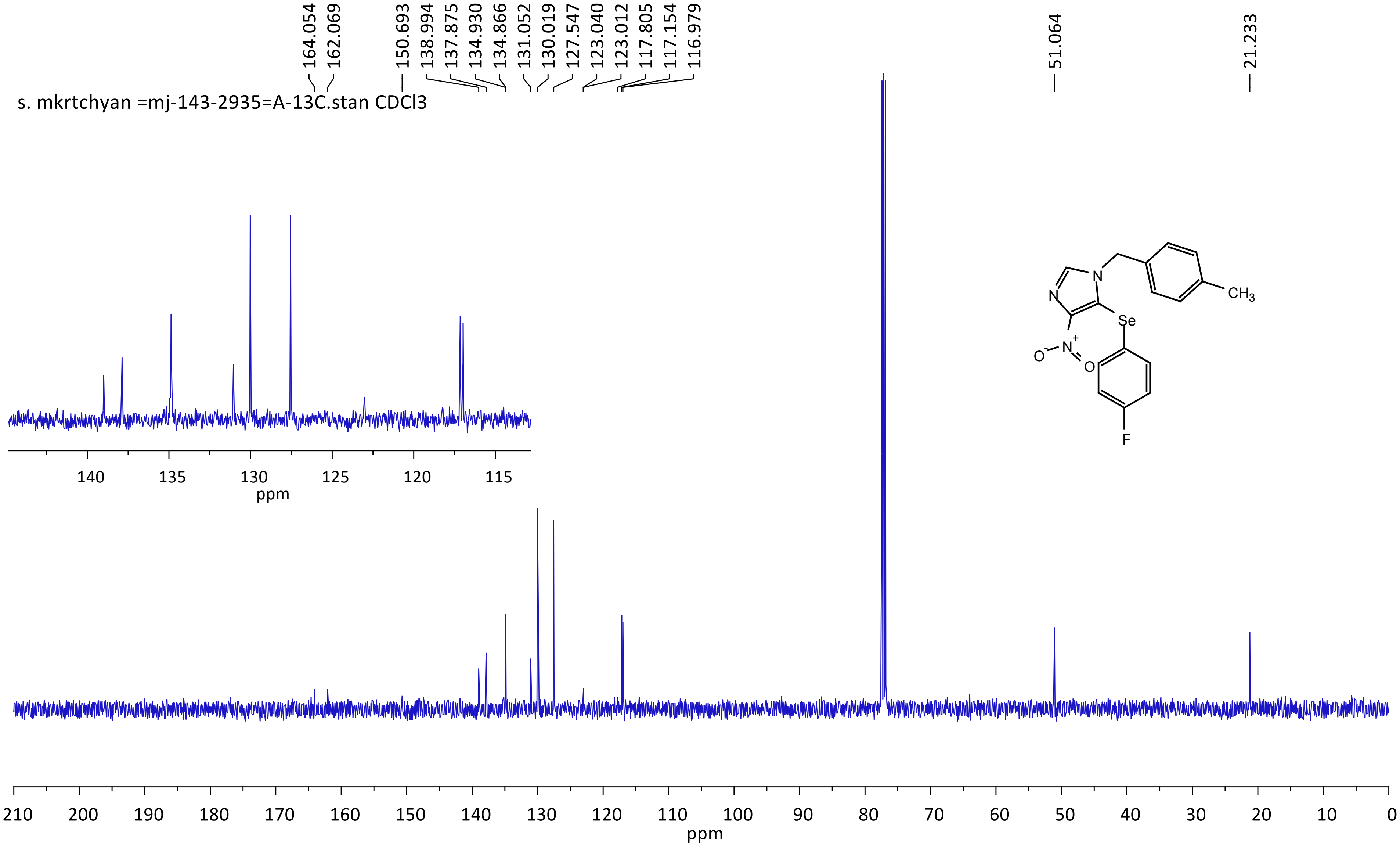

Comment	s. mkrtchyan =mj-143-2935=A-13C.stan CDCl3
Number of Scans	128
Spectrometer Frequency	125.76
Spectral Width	36057.7
Spectral Size	65536

Compound **7a**

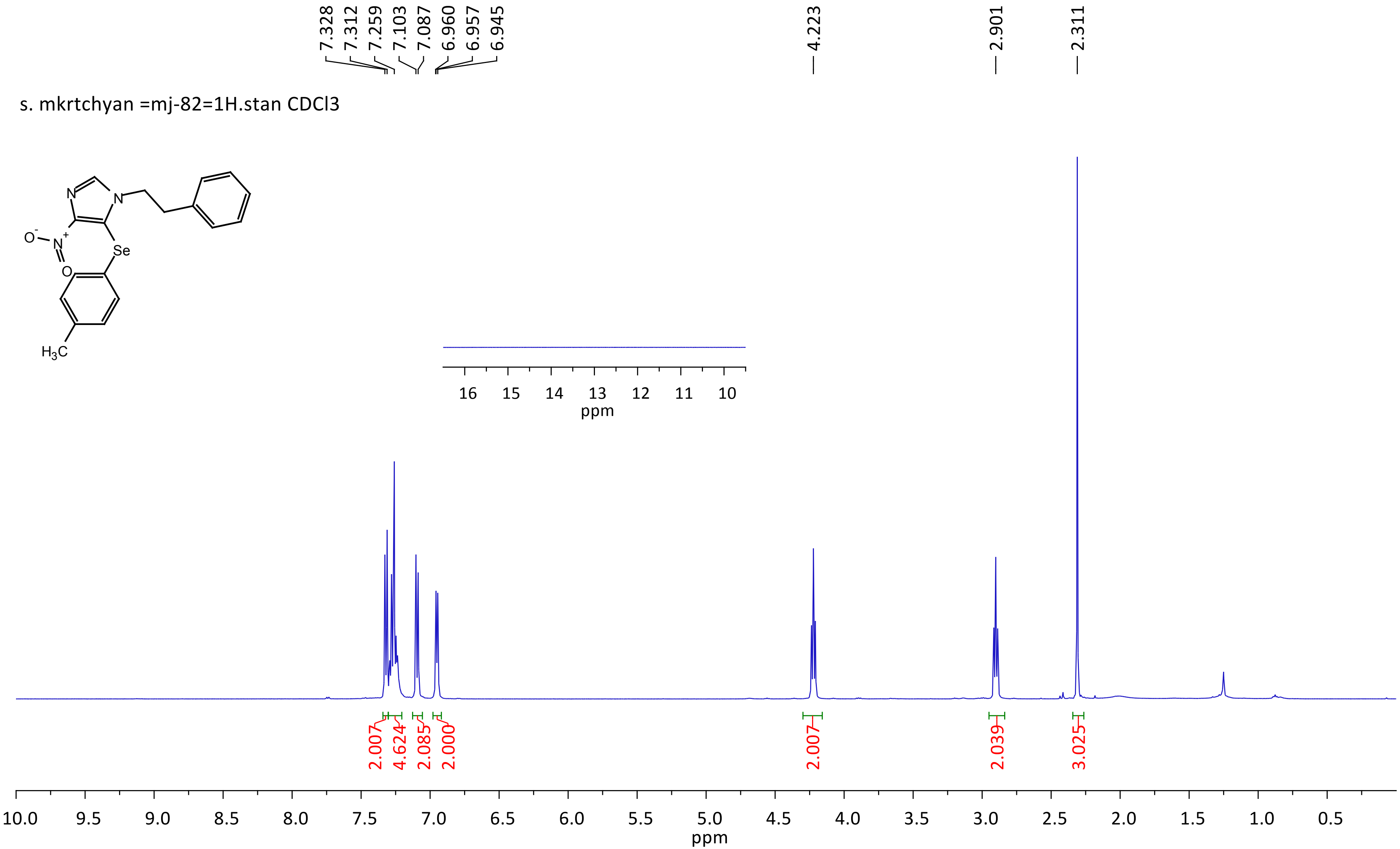

Comment	s. mkrtchyan =mj-82=1H.stan CDCl3
Number of Scans	24
Spectrometer Frequency	500.13
Spectral Width	12335.5
Spectral Size	65536

Compound **7a**

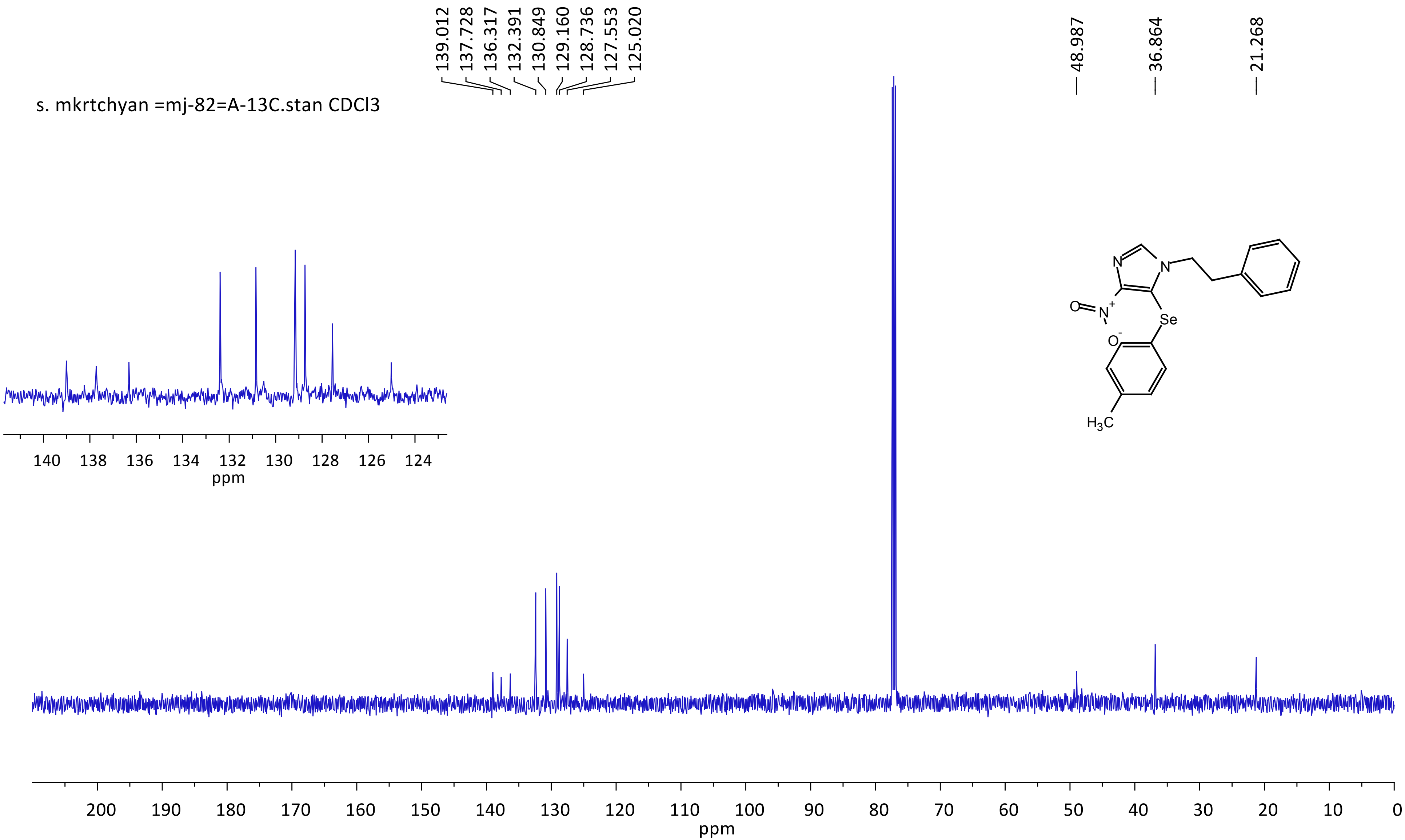

Comment	s. mkrtchyan =mj-82=A-13C.stan CDCl3
Number of Scans	128
Spectrometer Frequency	125.76
Spectral Width	36057.7
Spectral Size	65536

Compound **7b**

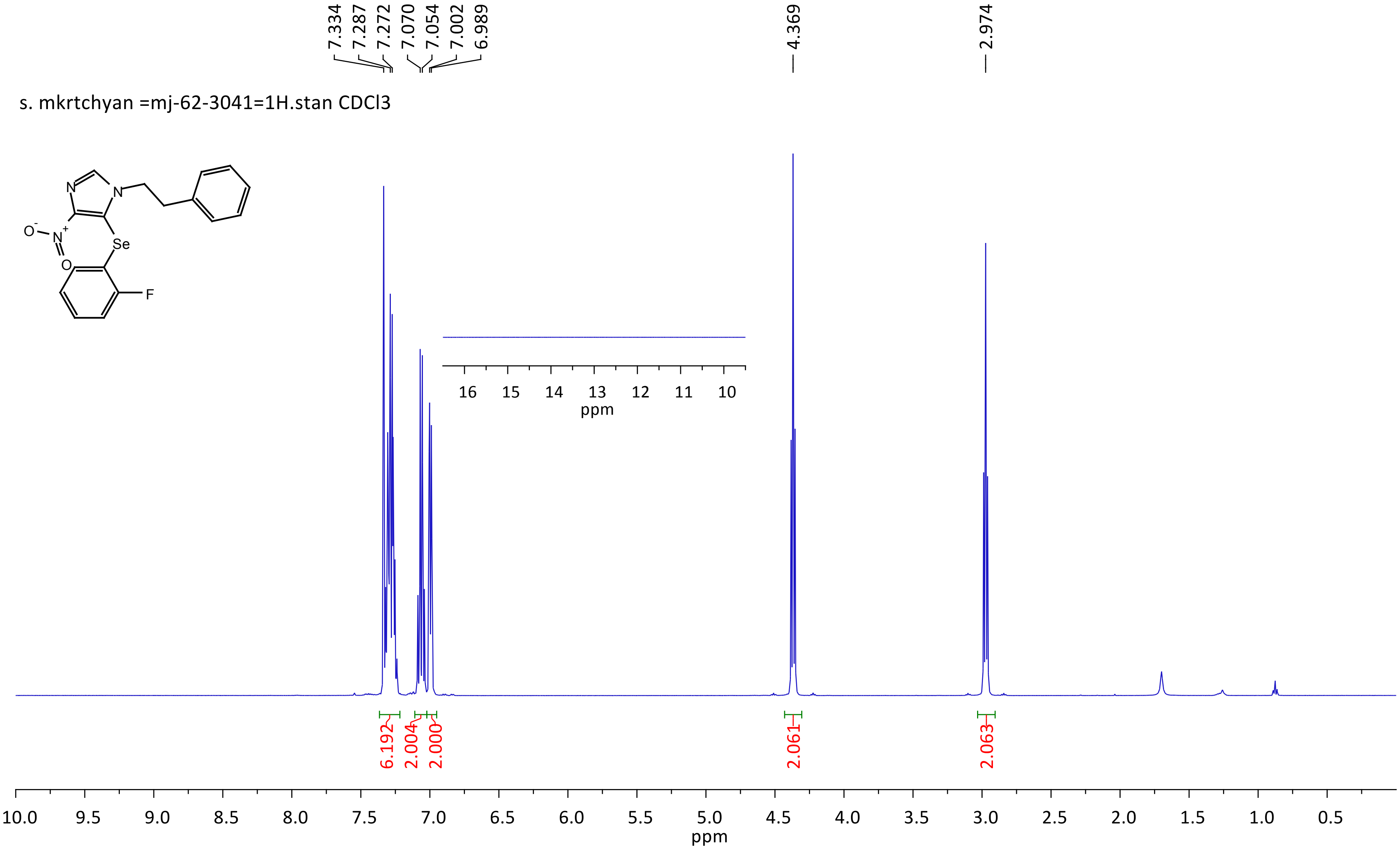

Comment	s. mkrtchyan =mj-62-3041=1H.stan CDCl3
Number of Scans	24
Spectrometer Frequency	500.13
Spectral Width	12335.5
Spectral Size	65536

Compound **7b**

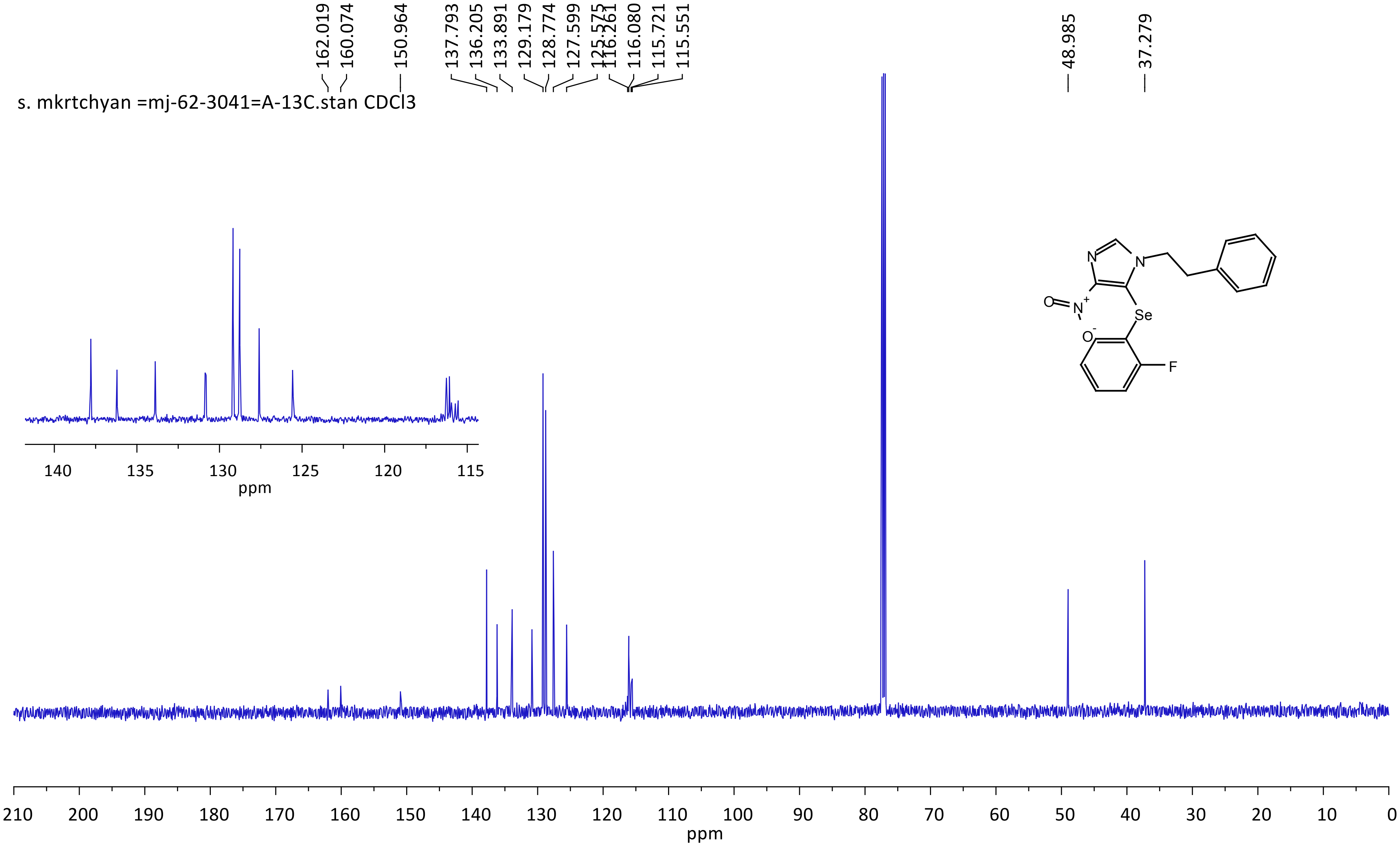

Comment	s. mkrtchyan =mj-62-3041=A-13C.stan CDCl3
Number of Scans	256
Spectrometer Frequency	125.76
Spectral Width	36057.7
Spectral Size	65536

Compound **7c**

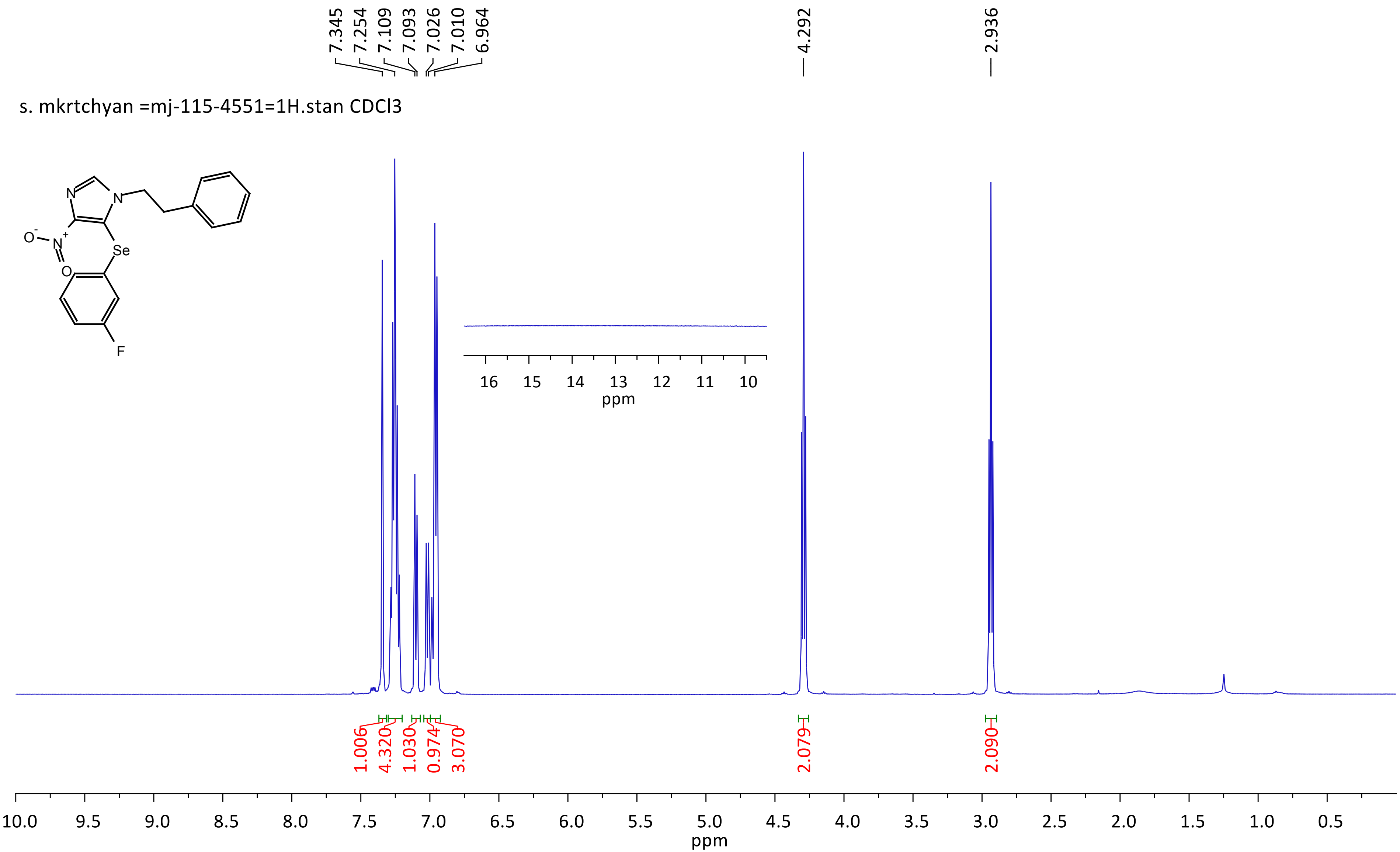

Comment	s. mkrtchyan =mj-115-4551=1H.stan CDCl3
Number of Scans	24
Spectrometer Frequency	500.13
Spectral Width	12335.5
Spectral Size	65536

Compound **7c**

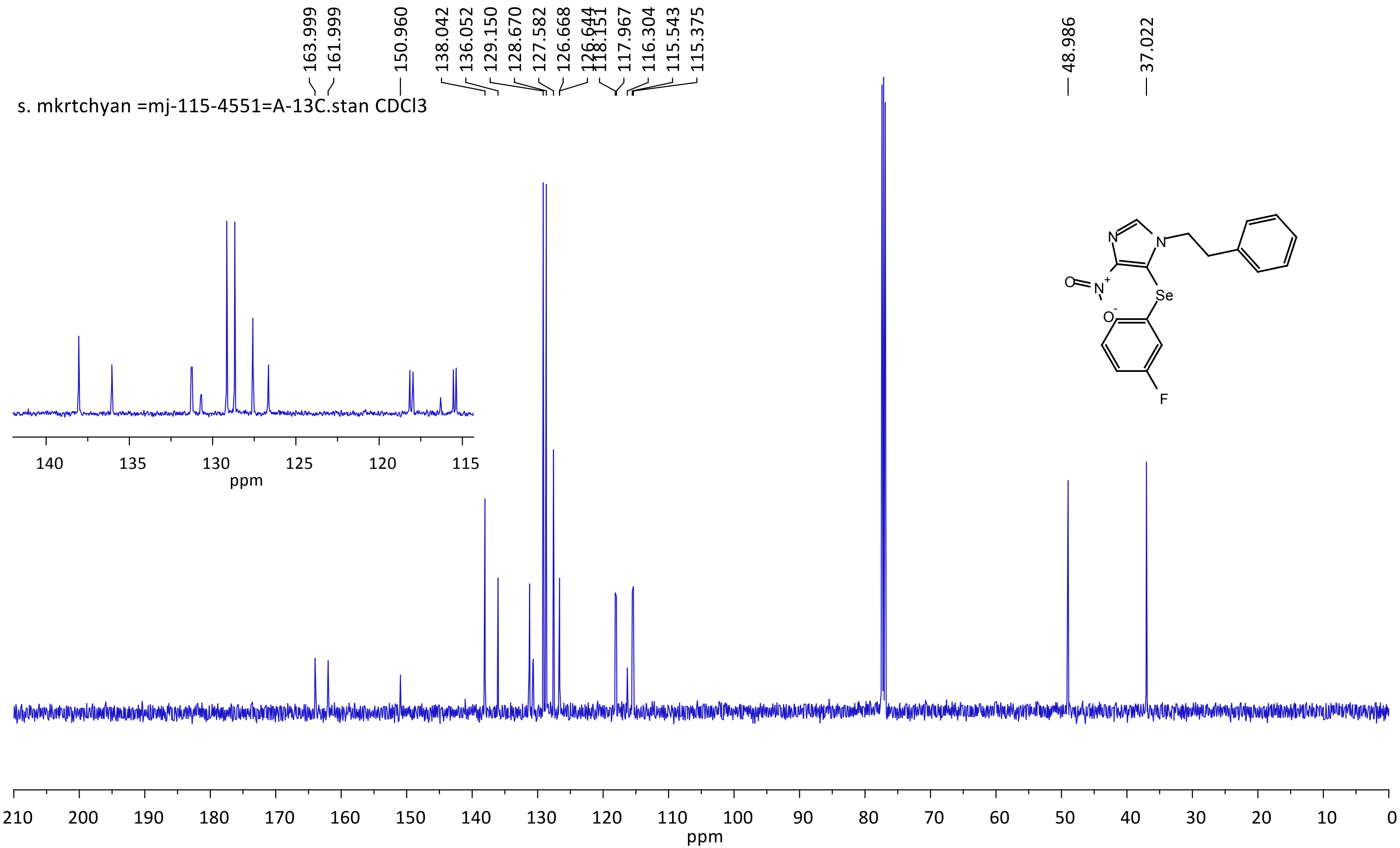

Comment	s. mkrtchyan =mj-115-4551=A-13C.stan CDCl3
Number of Scans	164
Spectrometer Frequency	125.76
Spectral Width	36057.7
Spectral Size	65536

Compound **7d**

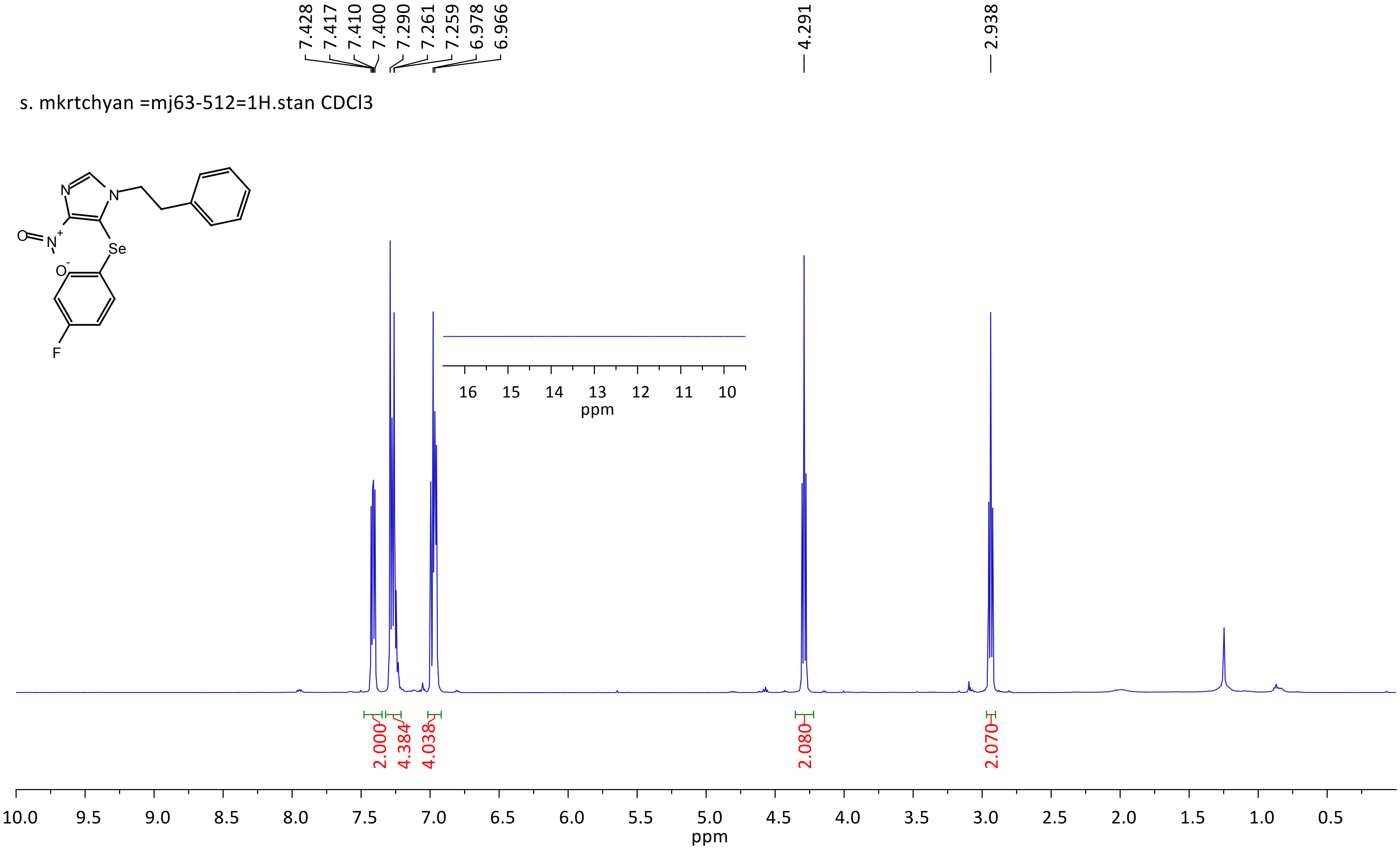

Comment	s. mkrtchyan =mj63-512=1H.stan CDCl3
Number of Scans	24
Spectrometer Frequency	500.13
Spectral Width	12335.5
Spectral Size	65536

Compound **7d**

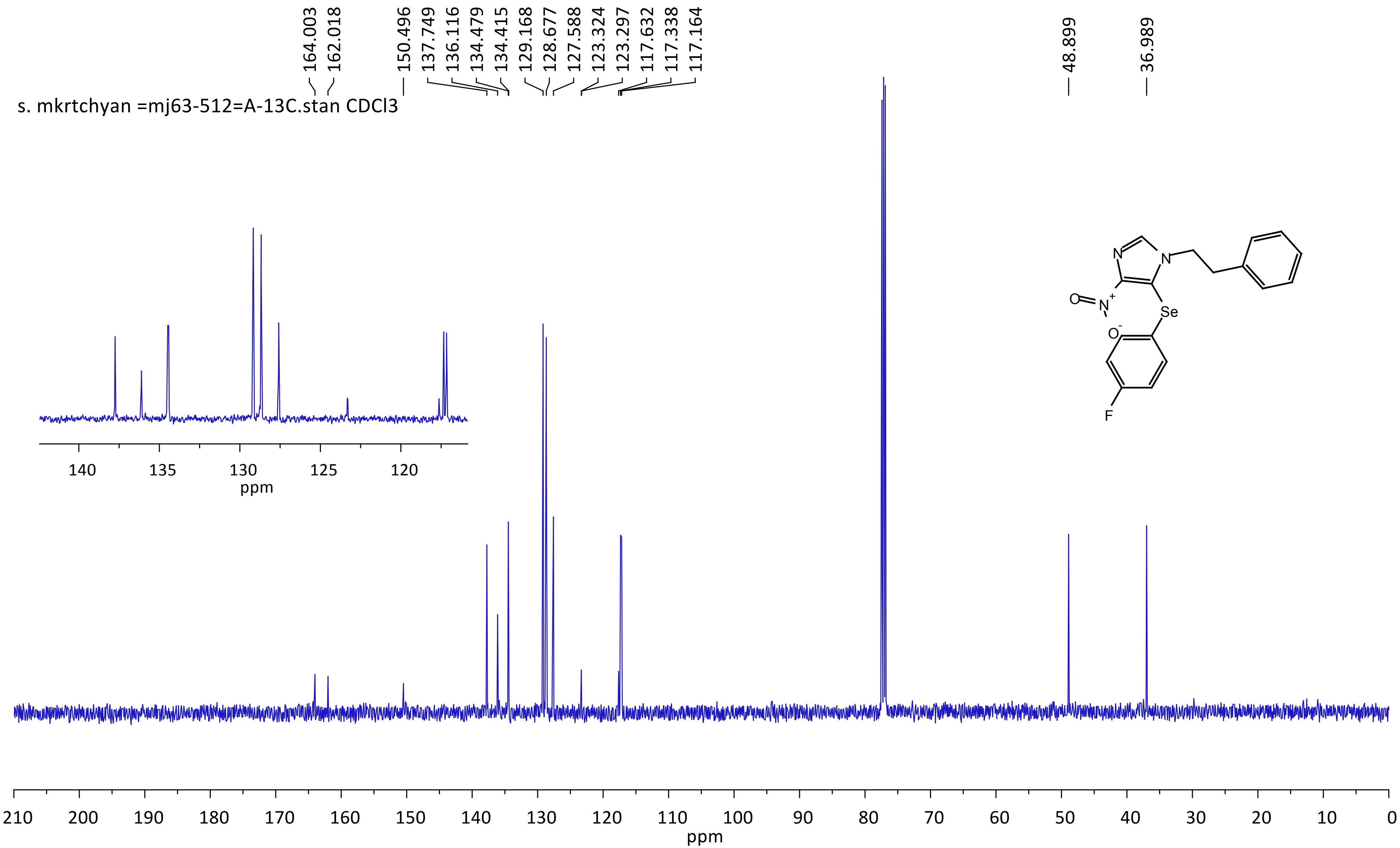

Comment	s. mkrtchyan =mj63-512=A-13C.stan CDCl3
Number of Scans	128
Spectrometer Frequency	125.76
Spectral Width	36057.7
Spectral Size	65536

Compound **8**

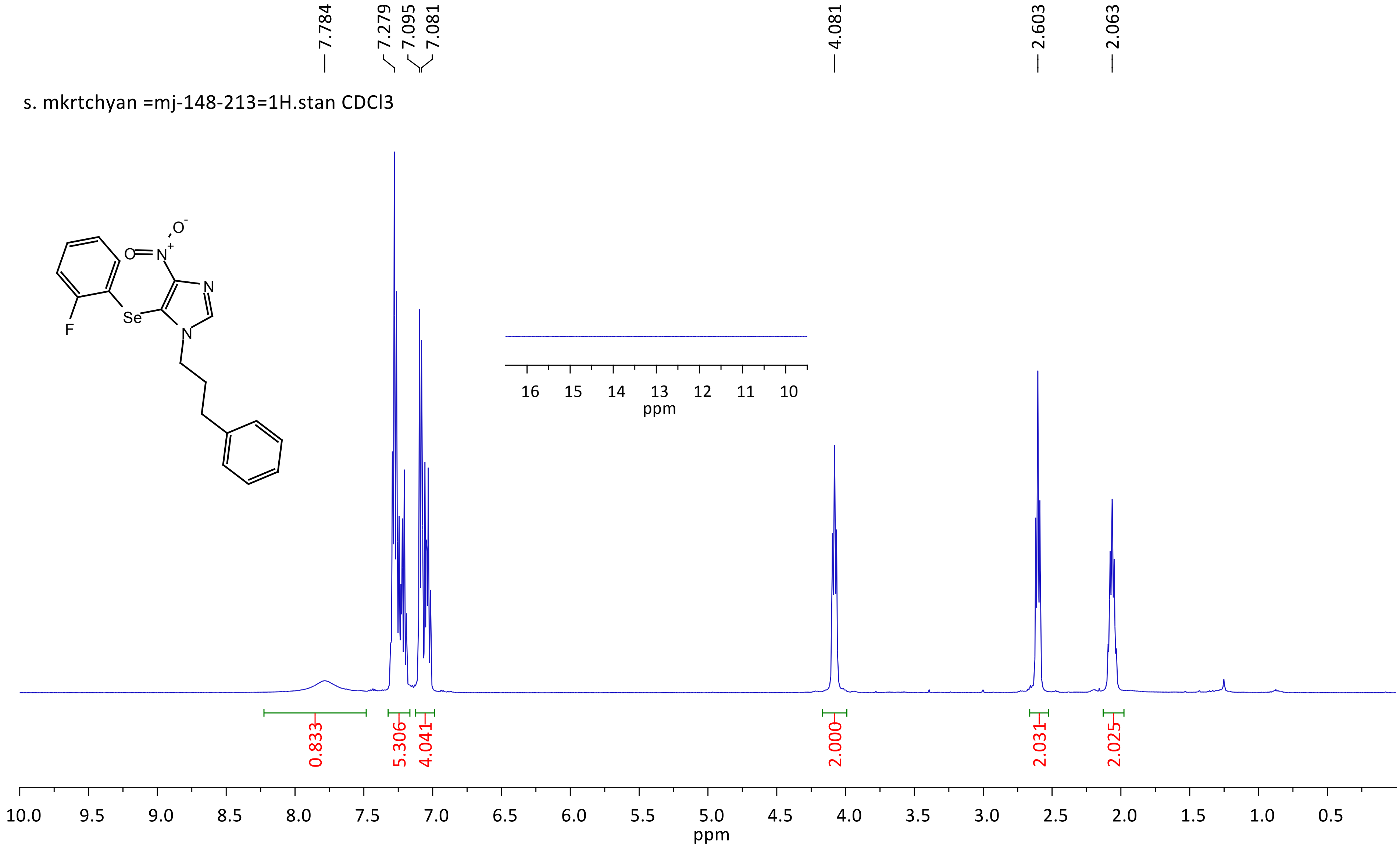

Comment	s. mkrtchyan =mj-148-213=1H.stan CDCl3
Number of Scans	16
Spectrometer Frequency	500.13
Spectral Width	12335.5
Spectral Size	65536

Compound **8**

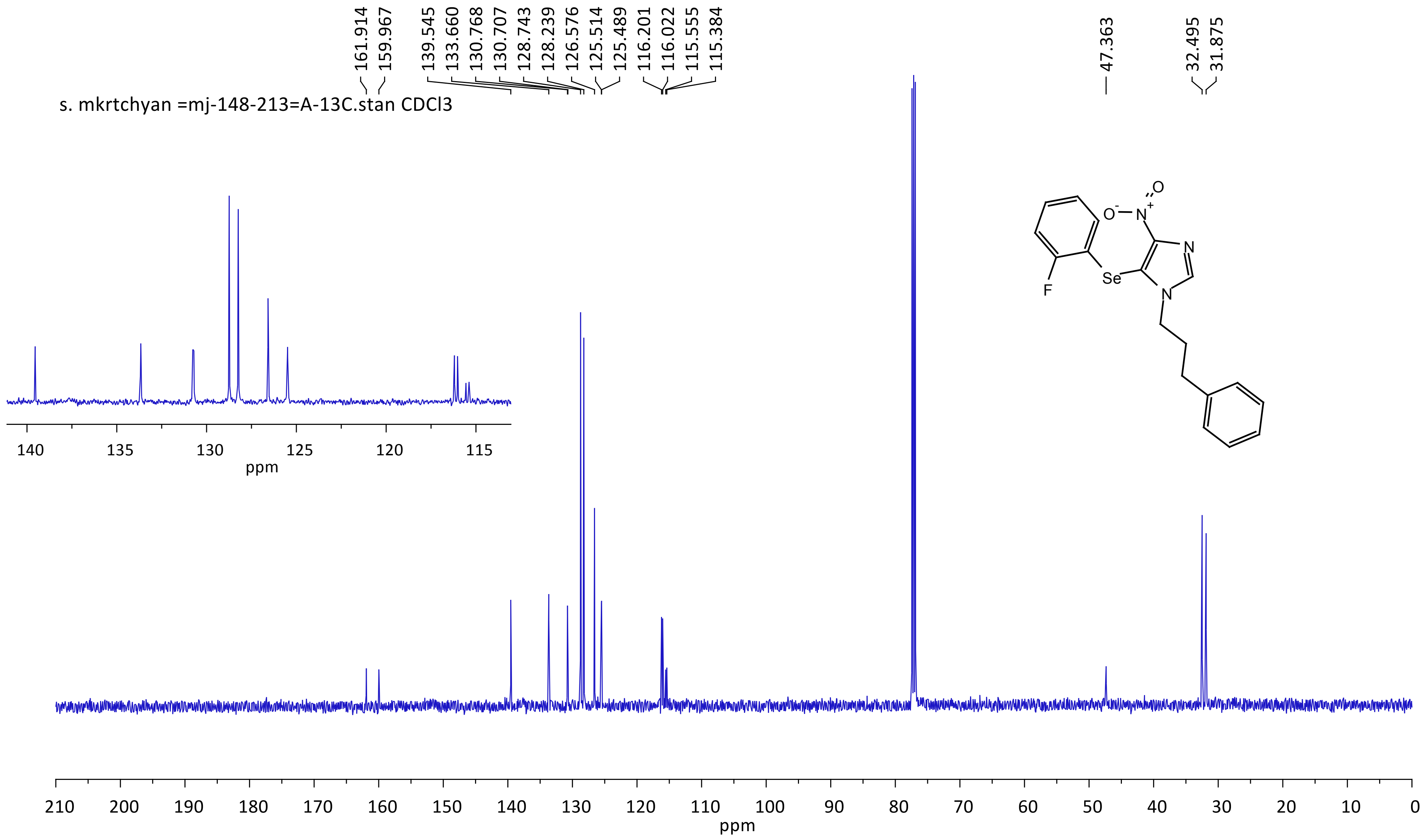

Comment	s. mkrtchyan =mj-148-213=A-13C.stan CDCl3
Number of Scans	148
Spectrometer Frequency	125.76
Spectral Width	36057.7
Spectral Size	65536

Compound **9a**

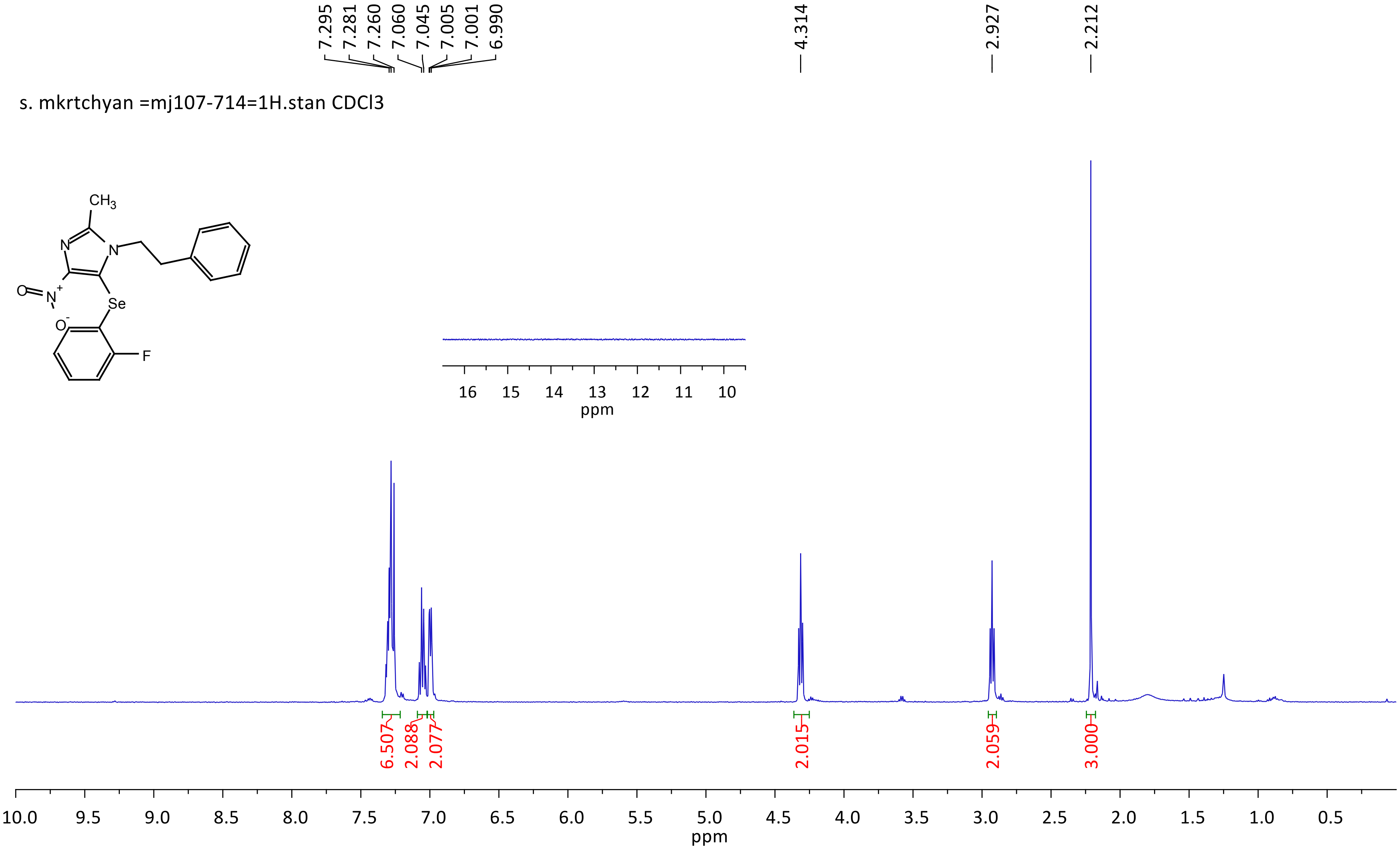

Comment	s. mkrtchyan =mj107-714=1H.stan CDCl3
Number of Scans	32
Spectrometer Frequency	500.13
Spectral Width	12335.5
Spectral Size	65536

Compound **9a**

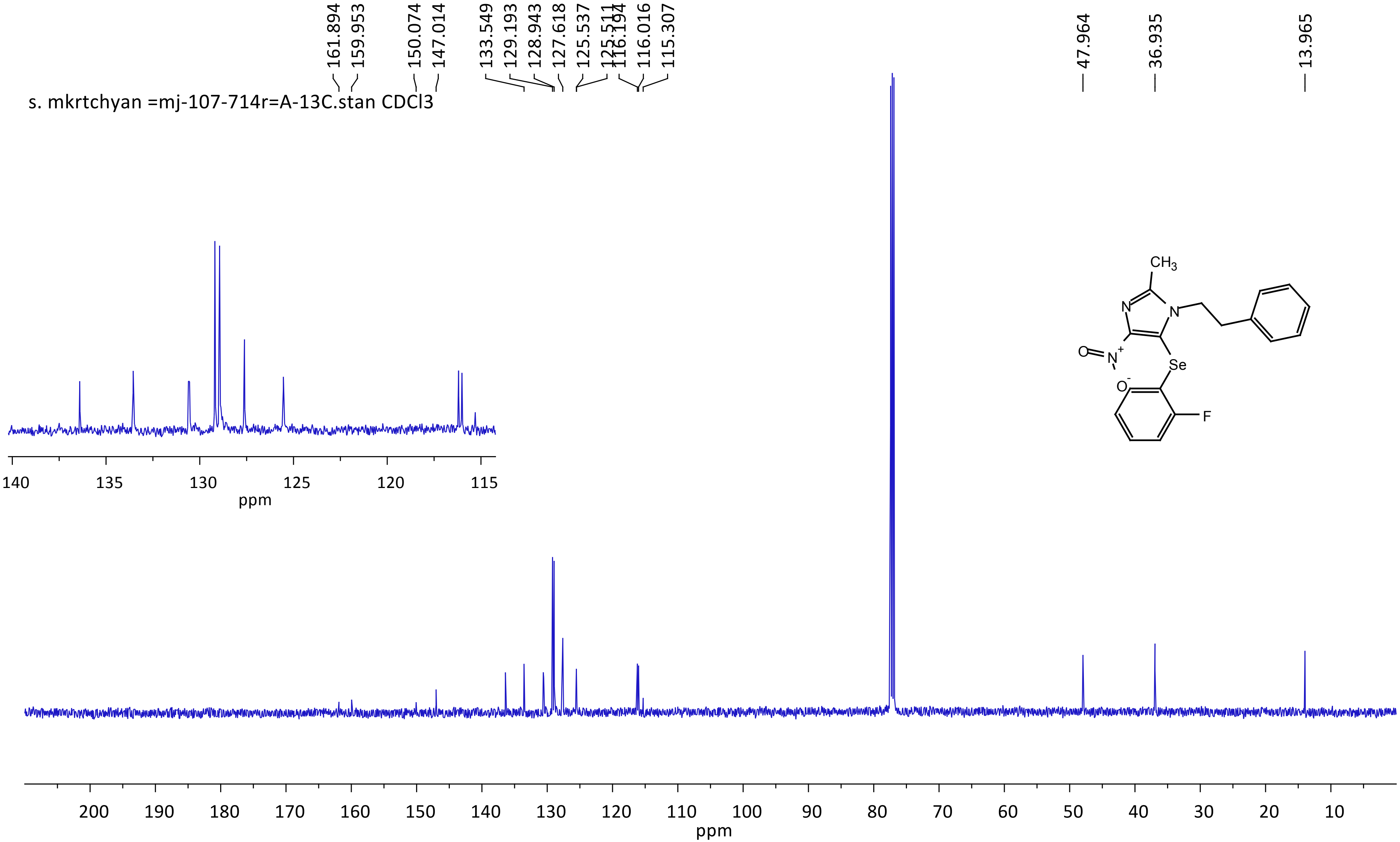

Comment	s. mkrtchyan =mj-107-714r=A-13C.stan CDCl3
Number of Scans	512
Spectrometer Frequency	125.76
Spectral Width	36057.7
Spectral Size	65536

Compound **9b**

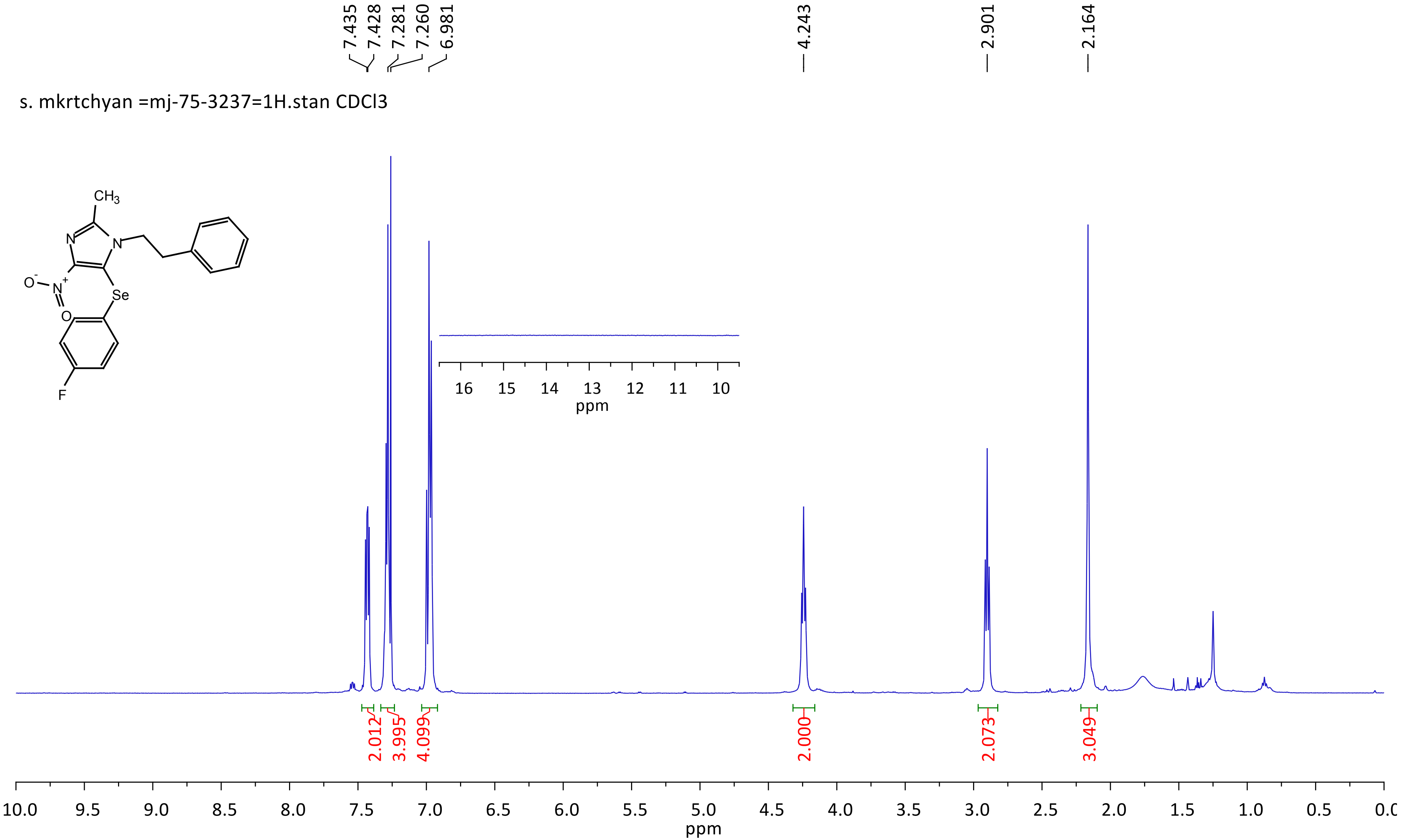

Comment	s. mkrtchyan =mj-75-3237=1H.stan CDCl3
Number of Scans	24
Spectrometer Frequency	500.13
Spectral Width	12335.5
Spectral Size	65536

Compound **9b**

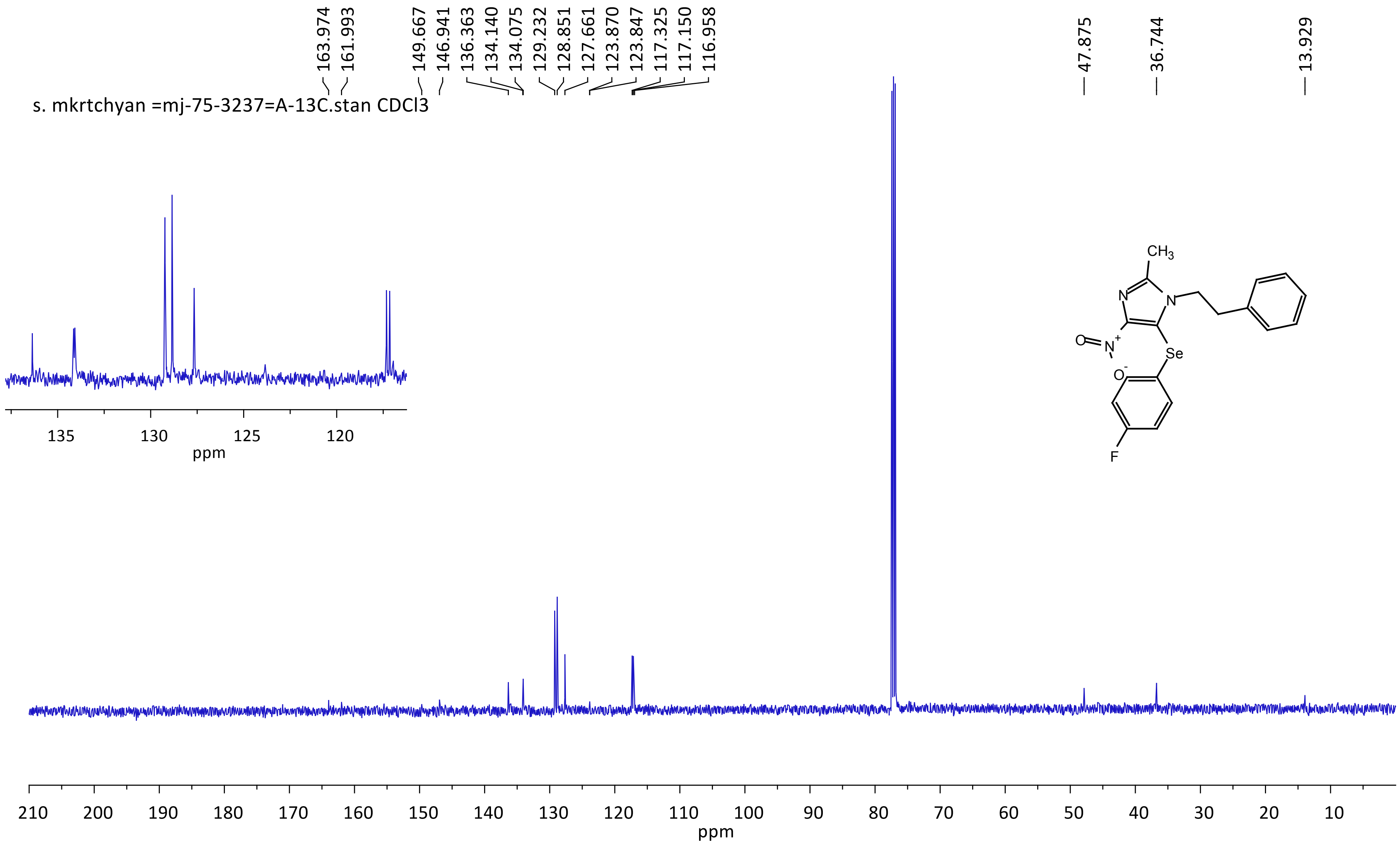

Comment	s. mkrtchyan =mj-75-3237=A-13C.stan CDCl3
Number of Scans	512
Spectrometer Frequency	125.76
Spectral Width	36057.7
Spectral Size	65536

Compound **10a**

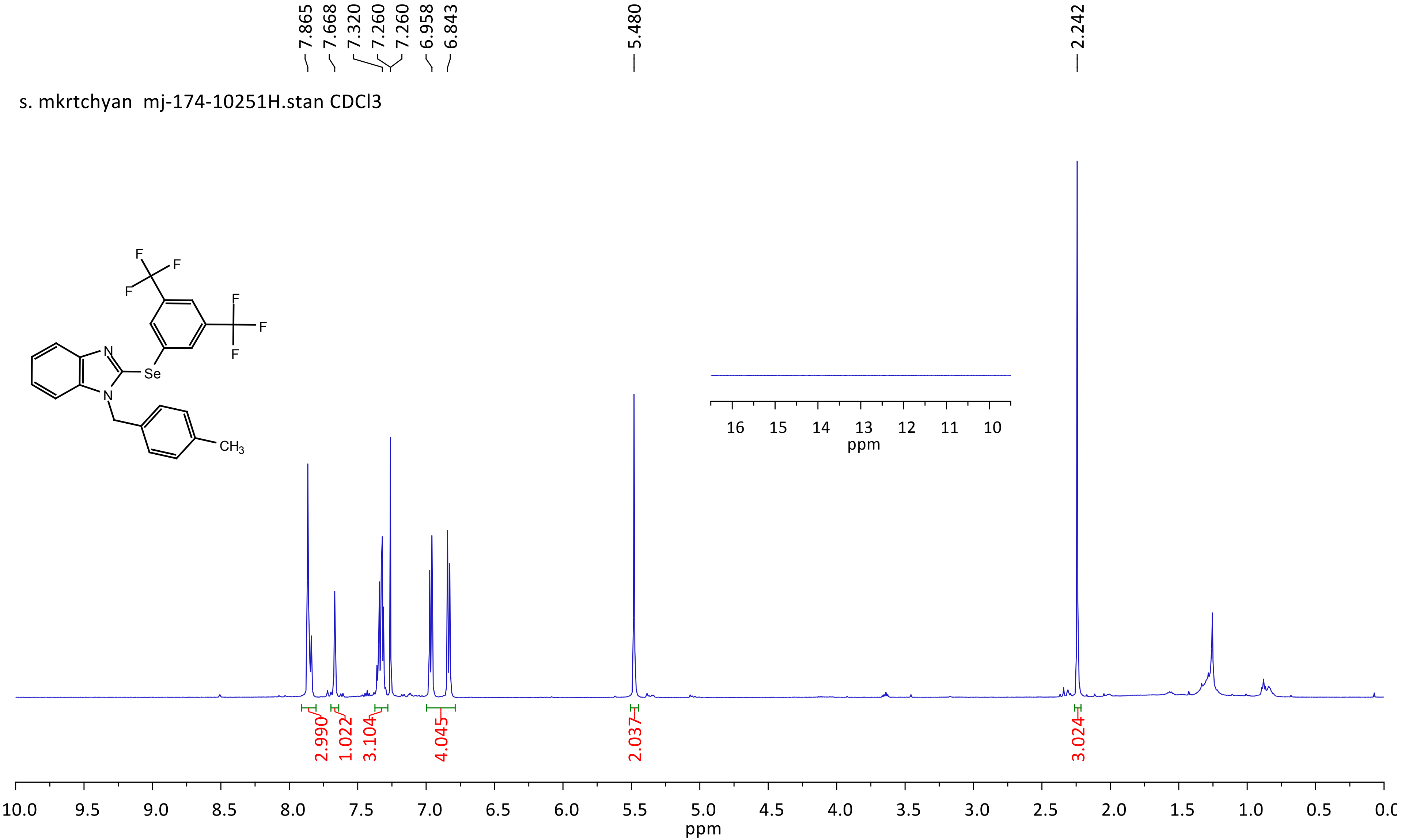

Comment	s. mkrtchyan mj-174-10251H.stan CDCl3
Number of Scans	24
Spectrometer Frequency	500.13
Spectral Width	12335.5
Spectral Size	65536

Compound **10a**

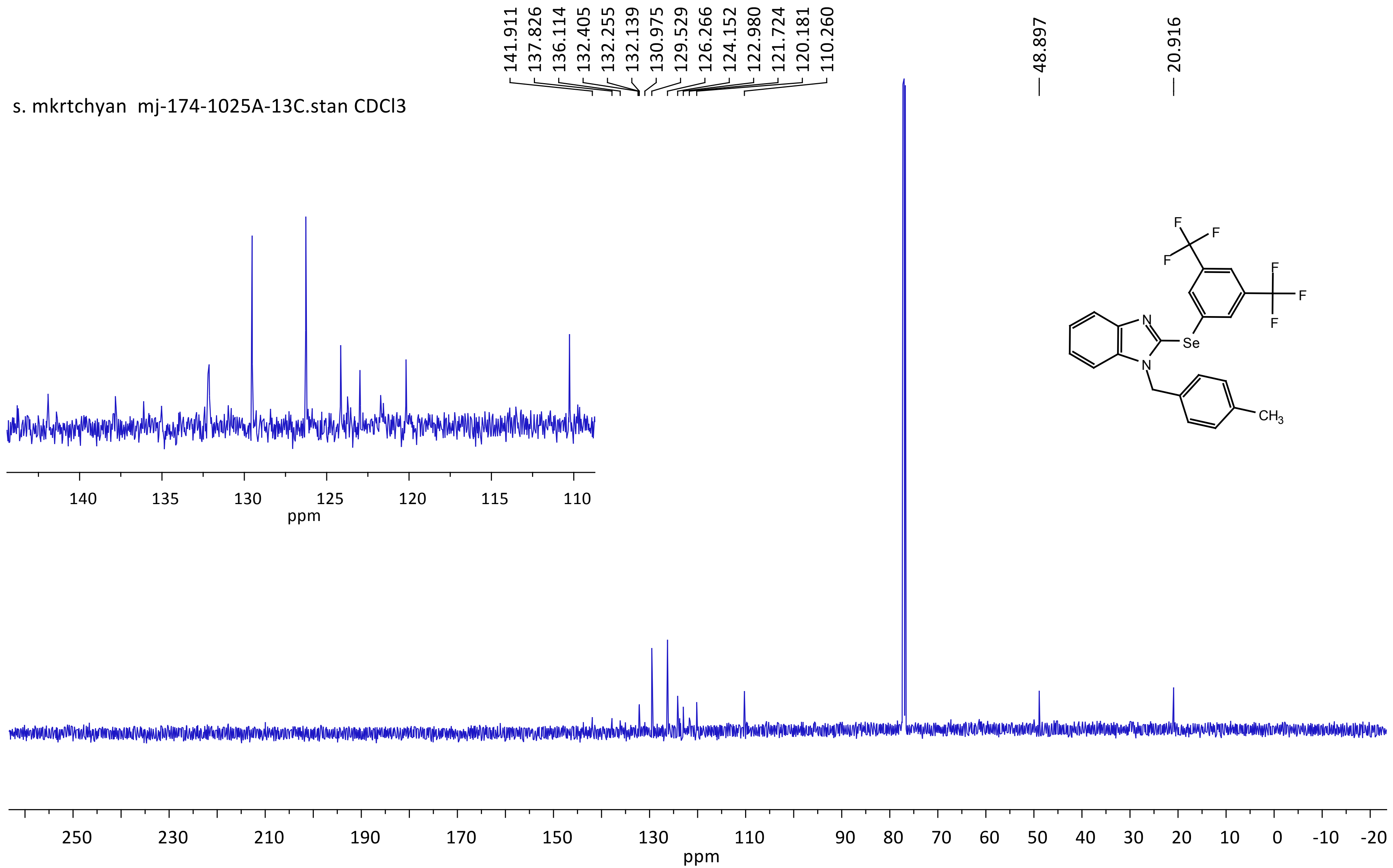

Comment	s. mkrtchyan mj-174-1025A-13C.stan CDCl3
Number of Scans	512
Spectrometer Frequency	125.76
Spectral Width	36057.7
Spectral Size	65536

Compound **10b**

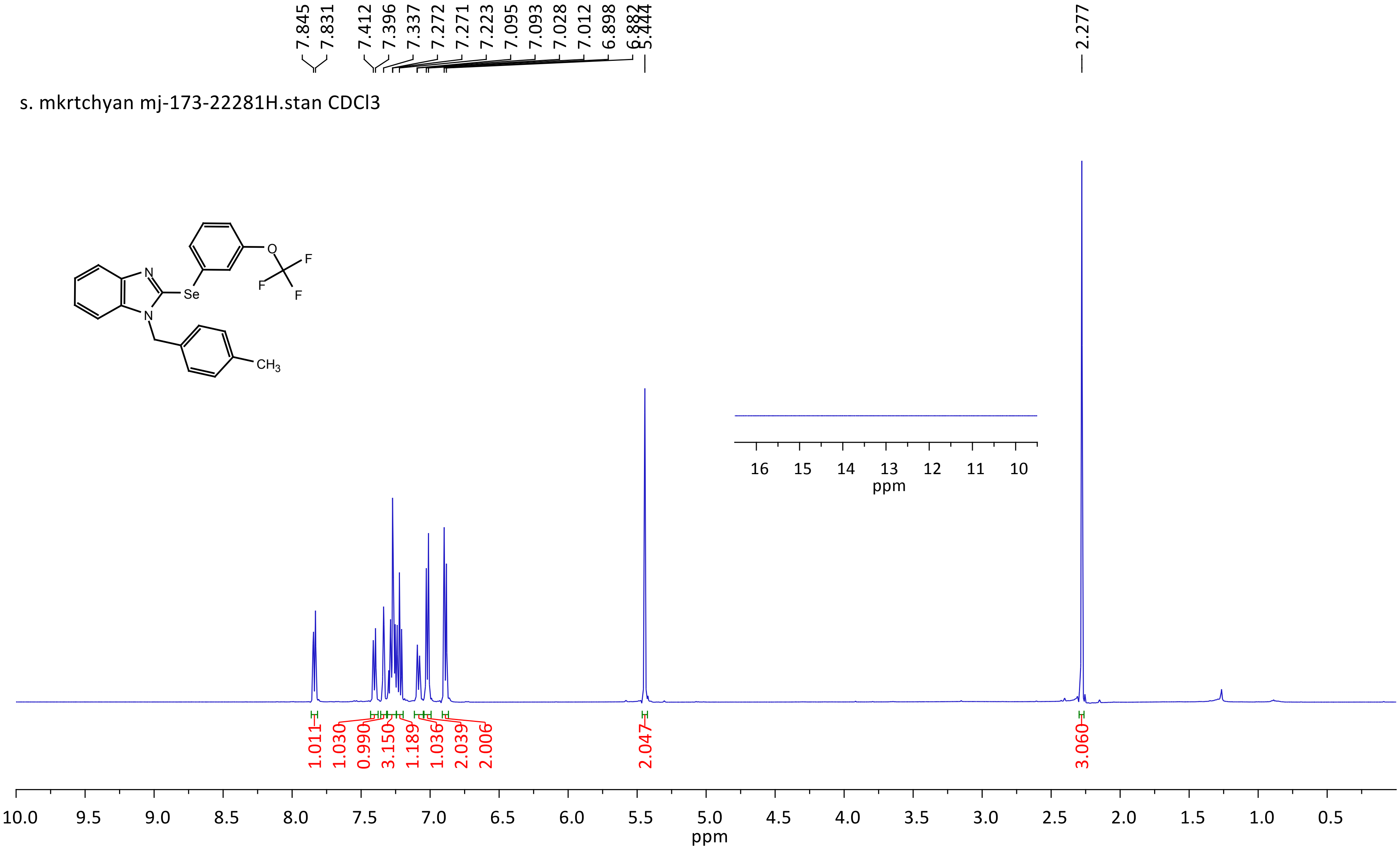

Comment	s. mkrtchyan mj-173-22281H.stan CDCl3
Number of Scans	24
Spectrometer Frequency	500.13
Spectral Width	12335.5
Spectral Size	65536

Compound **10b**

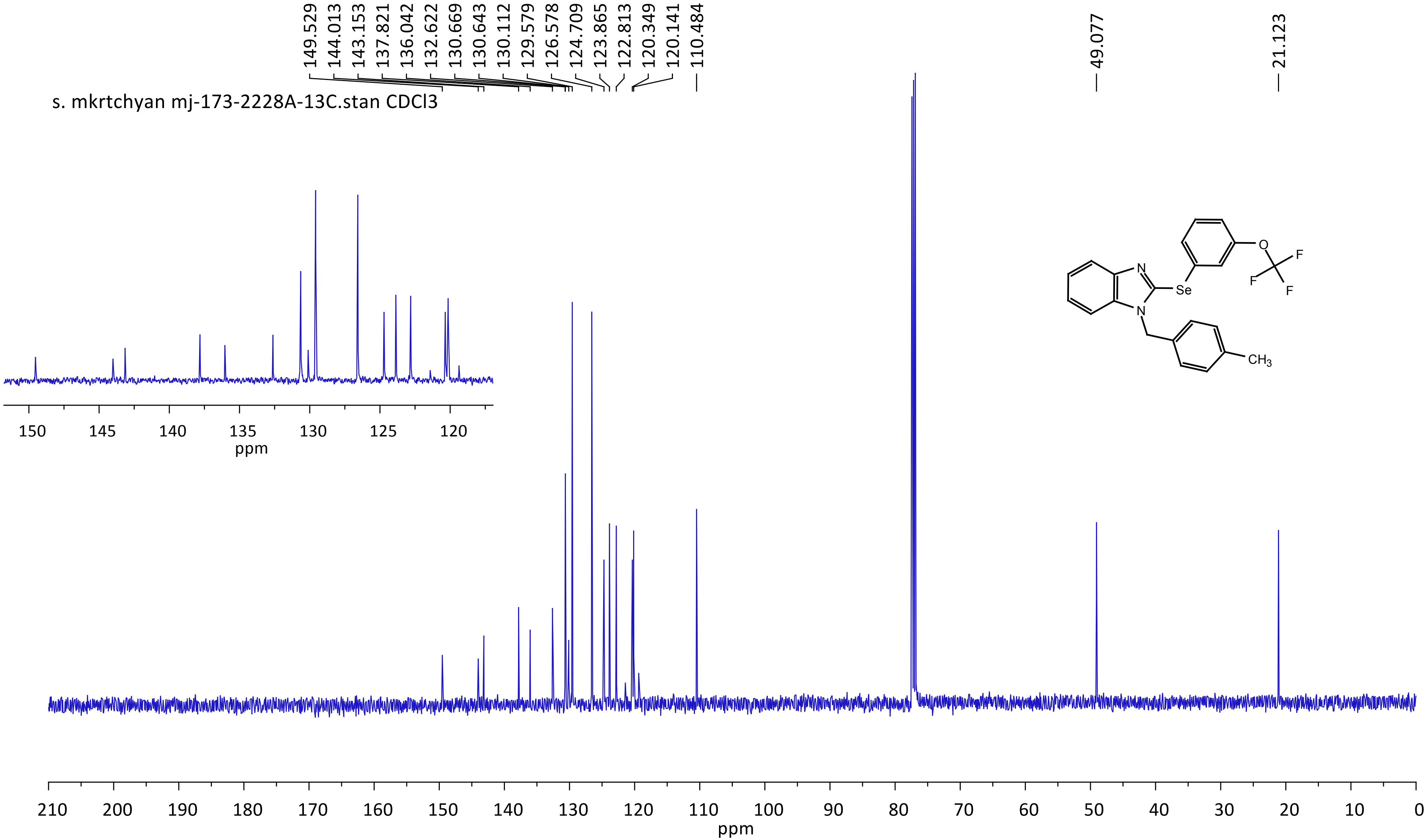

Comment	s. mkrtchyan mj-173-2228A-13C.stan CDCl3
Number of Scans	256
Spectrometer Frequency	125.76
Spectral Width	36057.7
Spectral Size	65536

Compound **11**

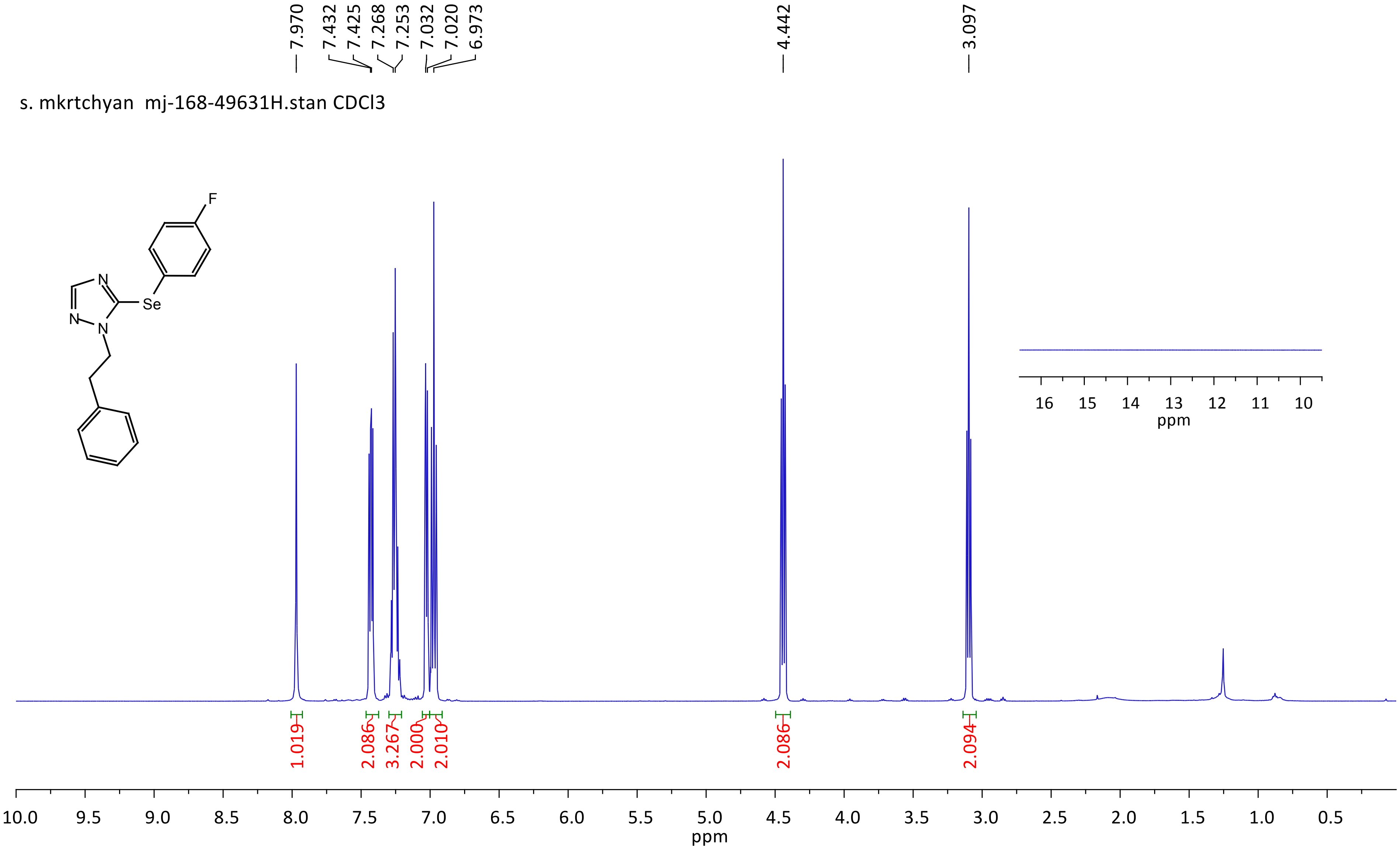

Comment	s. mkrtchyan mj-168-49631H.stan CDCl3
Number of Scans	24
Spectrometer Frequency	500.13
Spectral Width	12335.5
Spectral Size	65536

Compound **11**

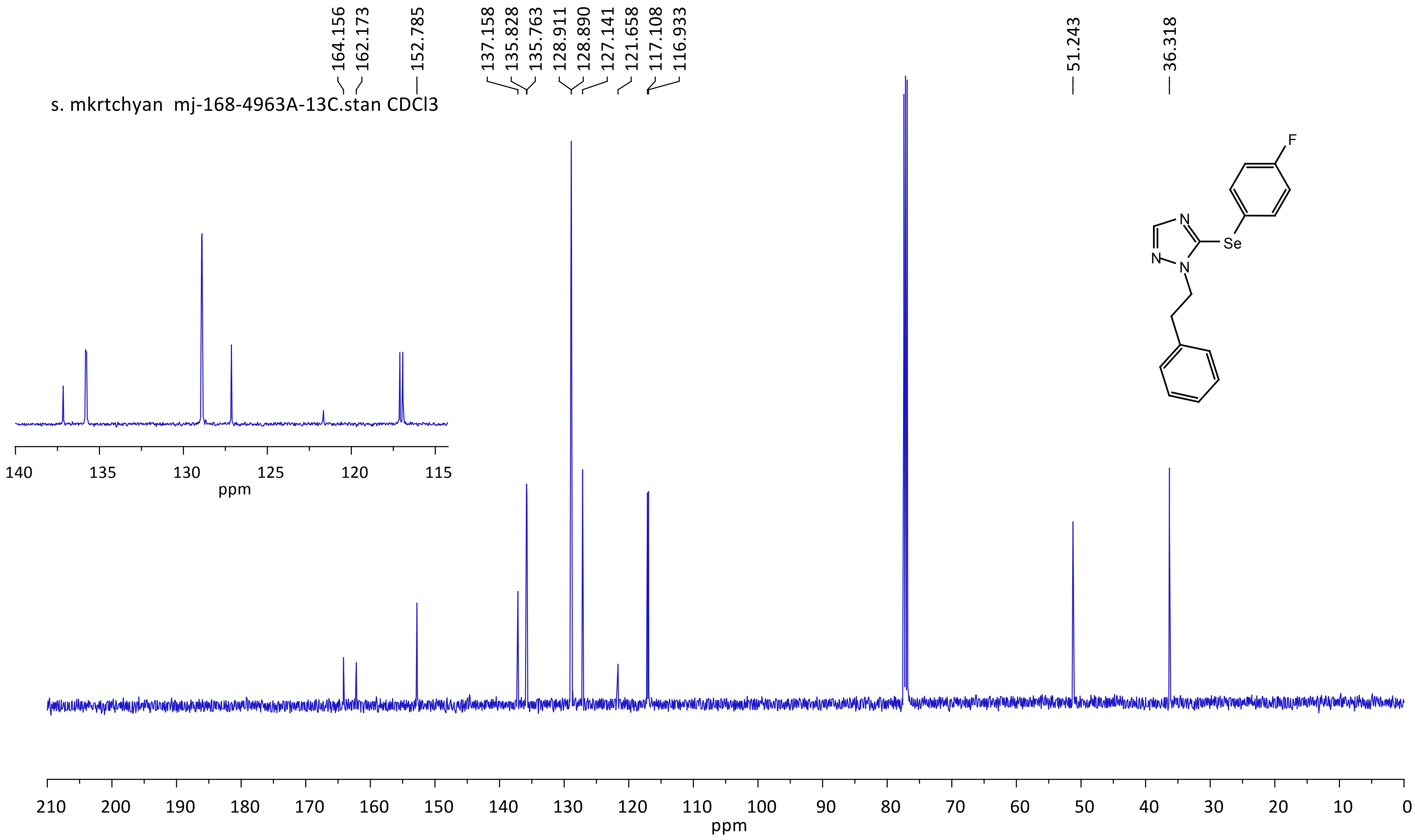

Comment	s. mkrtchyan mj-168-4963A-13C.stan CDCl3
Number of Scans	512
Spectrometer Frequency	125.76
Spectral Width	36057.7
Spectral Size	65536

Compound **12a**

s. mkrtchyan =mj-33-3537=1H.stan CDCl3

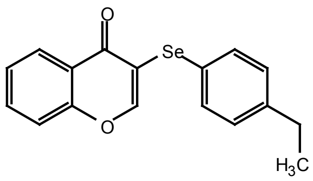

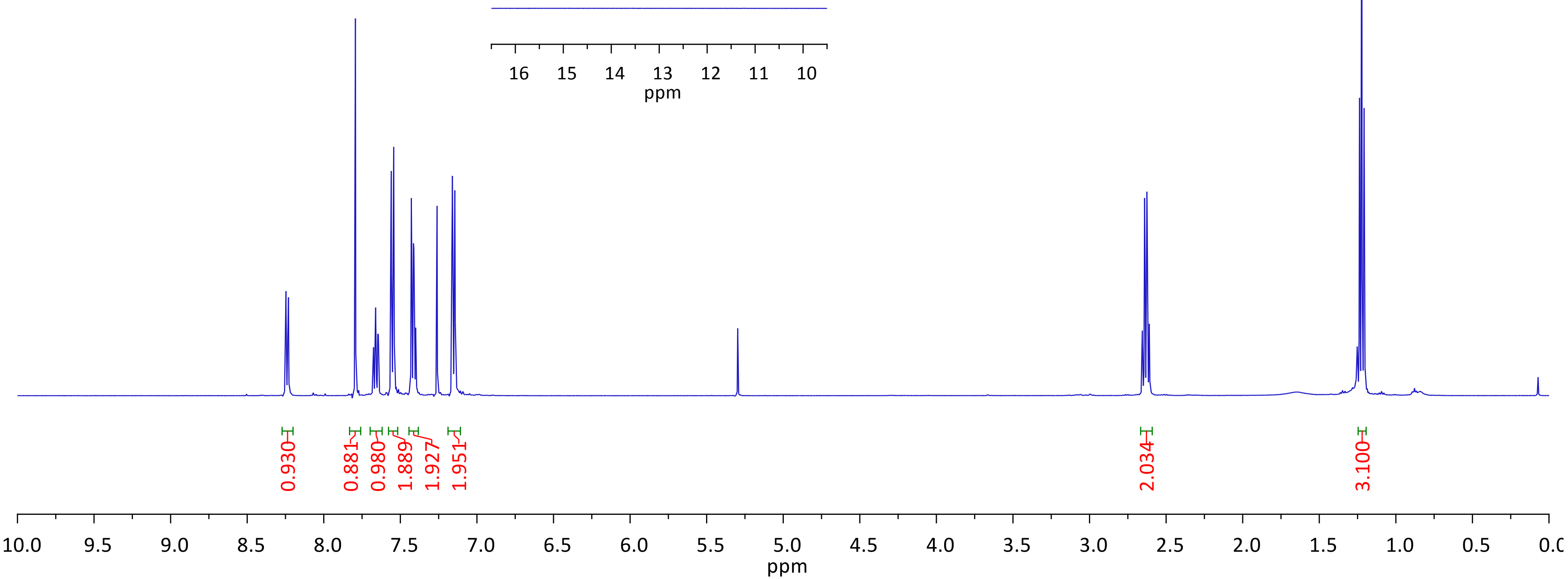

Comment	s. mkrtchyan =mj-33-3537=1H.stan CDCl3
Number of Scans	24
Spectrometer Frequency	500.13
Spectral Width	12335.5
Spectral Size	65536

Compound **12a**

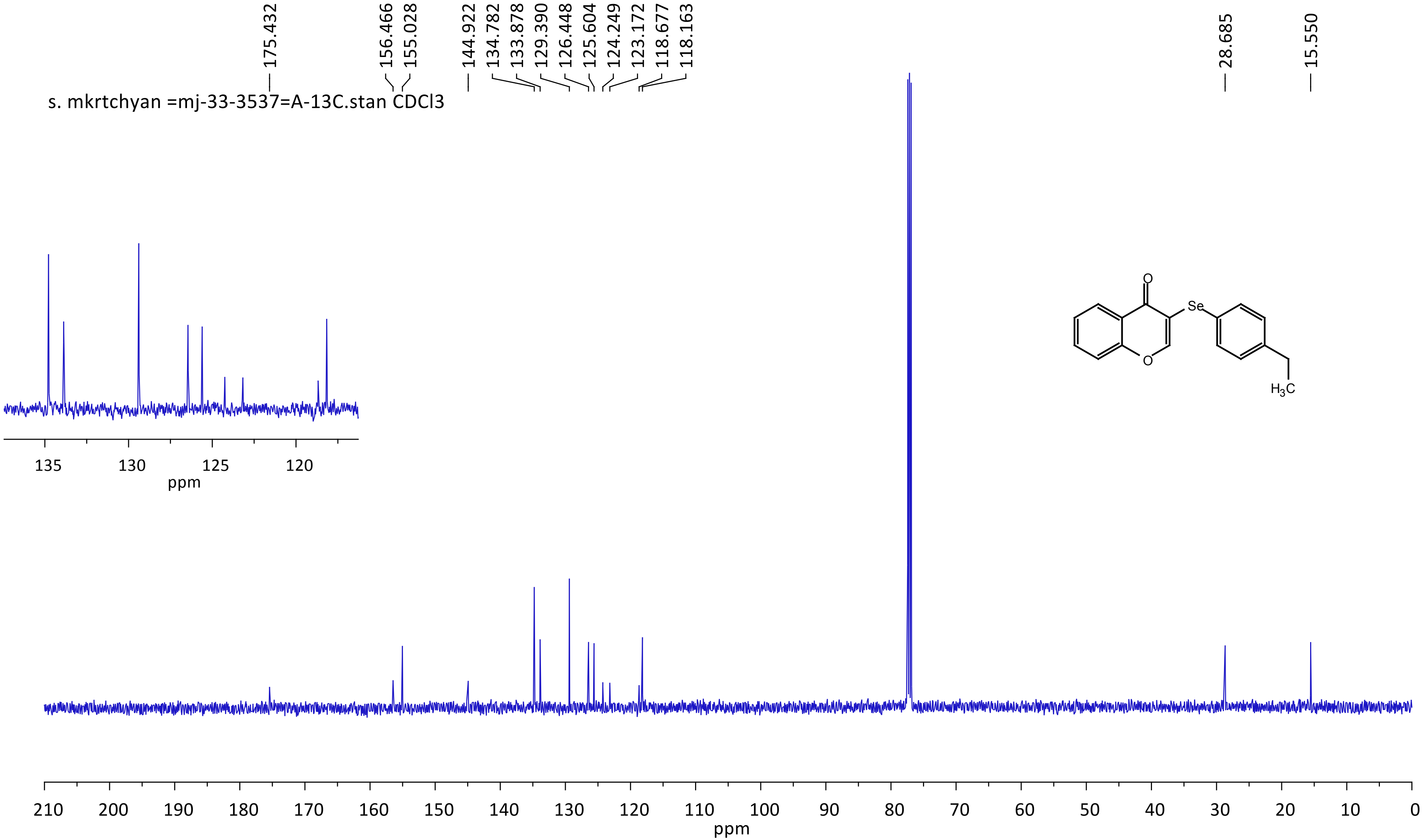

Comment	s. mkrtchyan =mj-33-3537=A-13C.stan CDCl3
Number of Scans	256
Spectrometer Frequency	125.76
Spectral Width	36057.7
Spectral Size	65536

Compound **12b**

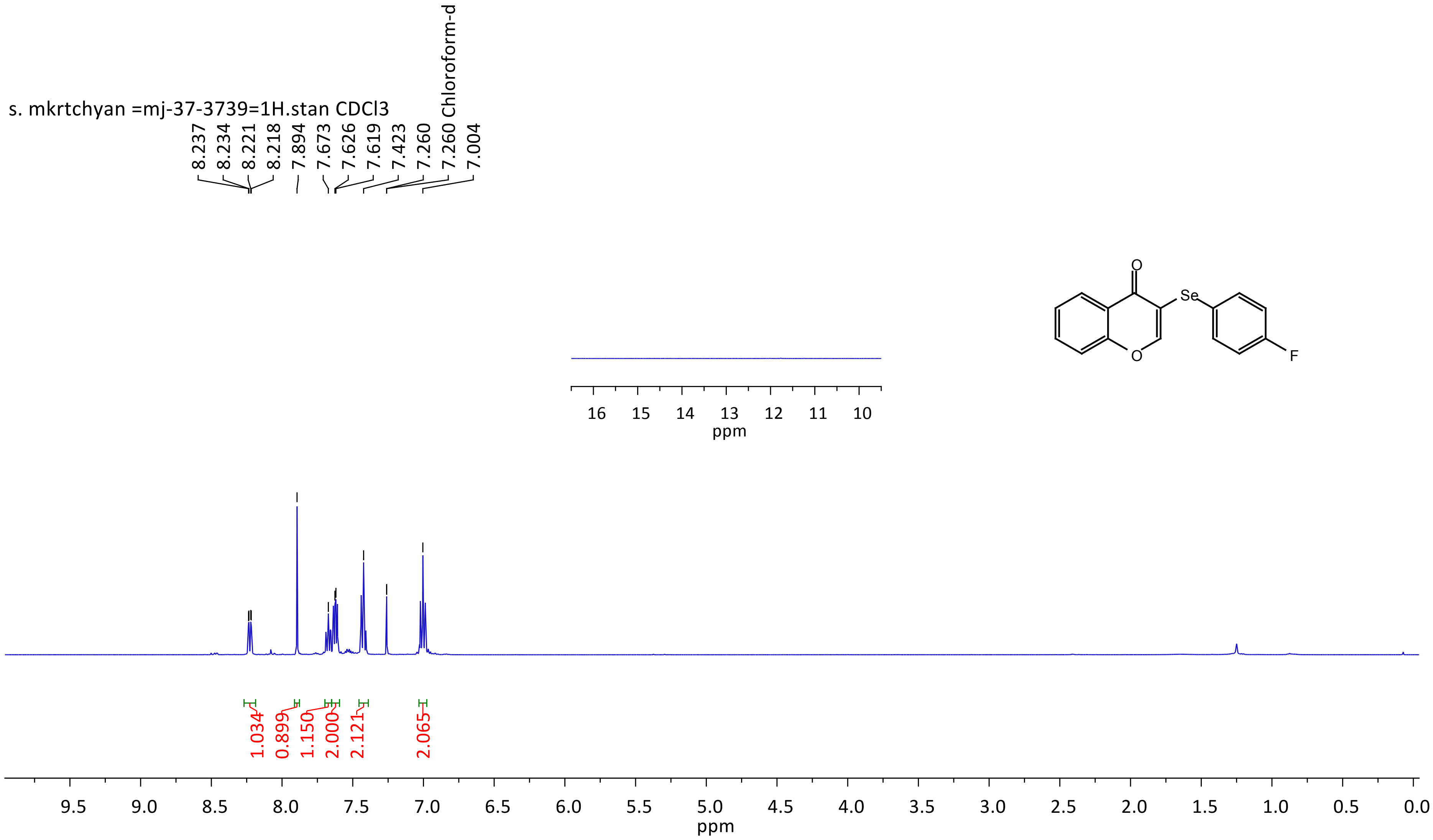

Comment	s. mkrtchyan =mj-37-3739=1H.stan CDCl3
Number of Scans	32
Spectrometer Frequency	500.13
Spectral Width	12335.5
Spectral Size	65536

Compound **12b**

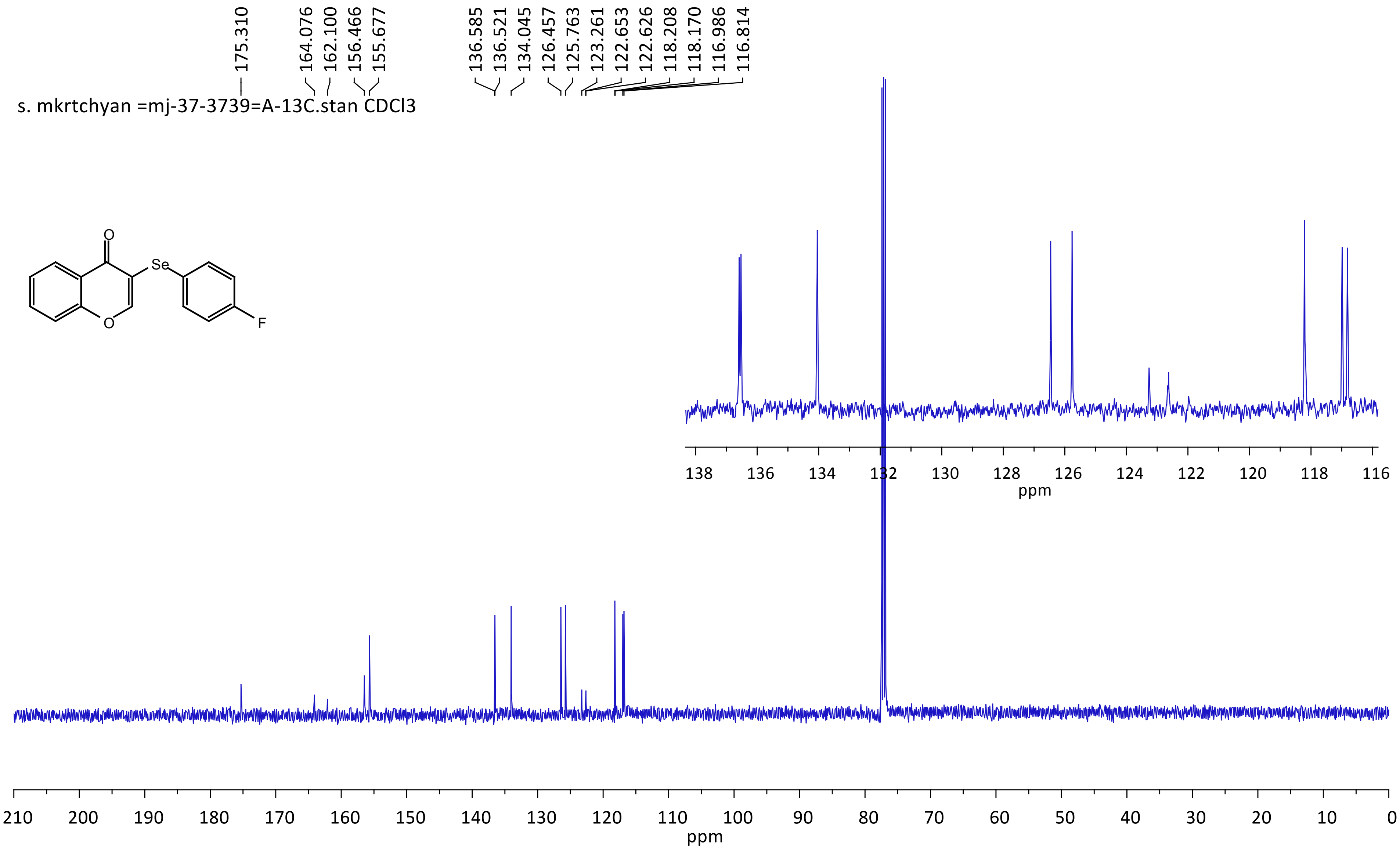

Comment	s. mkrtchyan =mj-37-3739=A-13C.stan CDCl3
Number of Scans	256
Spectrometer Frequency	125.76
Spectral Width	36057.7
Spectral Size	65536

Compound **13a**

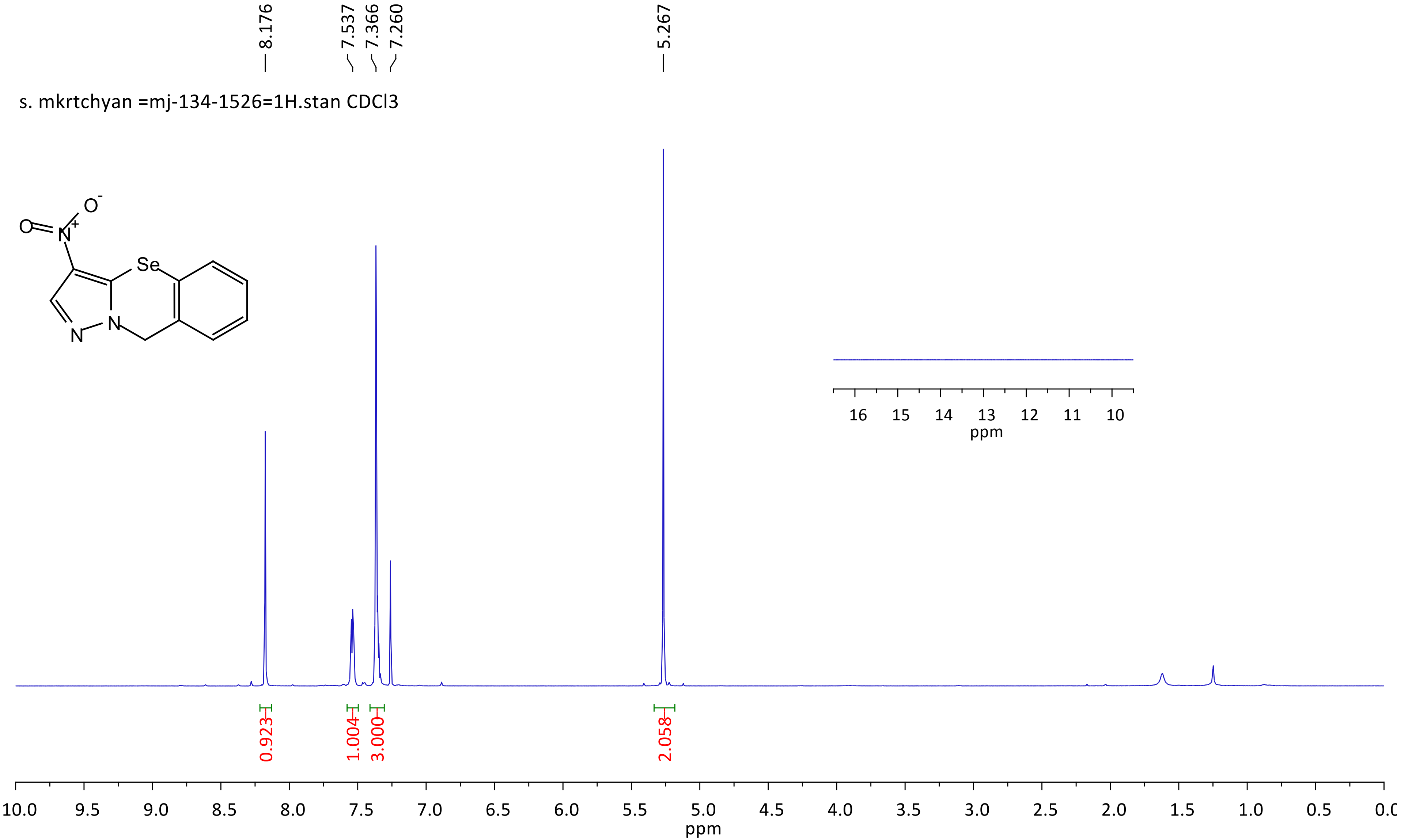

Comment	s. mkrtchyan =mj-134-1526=1H.stan CDCl3
Number of Scans	24
Spectrometer Frequency	500.13
Spectral Width	12335.5
Spectral Size	65536

Compound **13a**

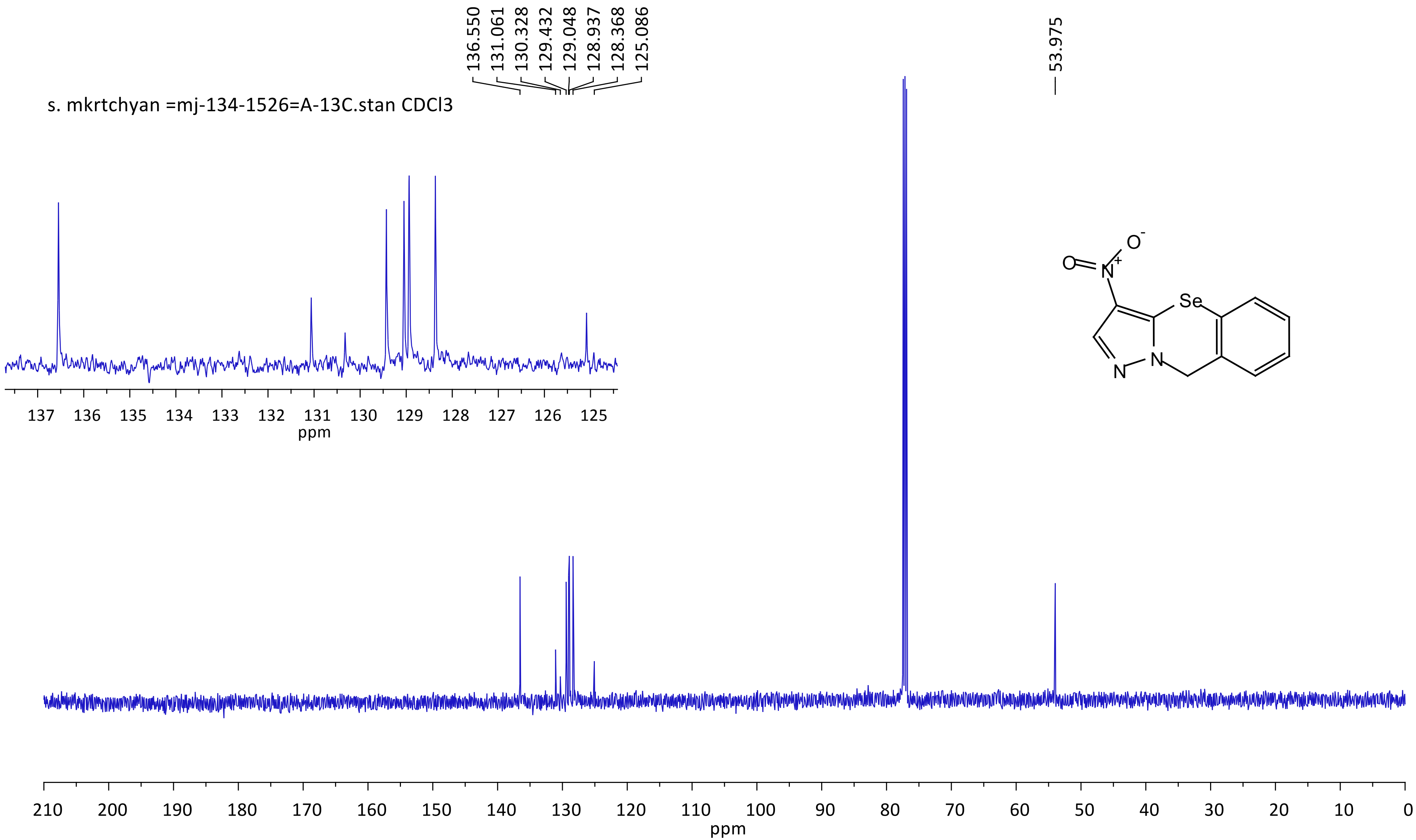

Comment	s. mkrtchyan =mj-134-1526=A-13C.stan CDCl3
Number of Scans	256
Spectrometer Frequency	125.76
Spectral Width	36057.7
Spectral Size	65536

Compound **13b**

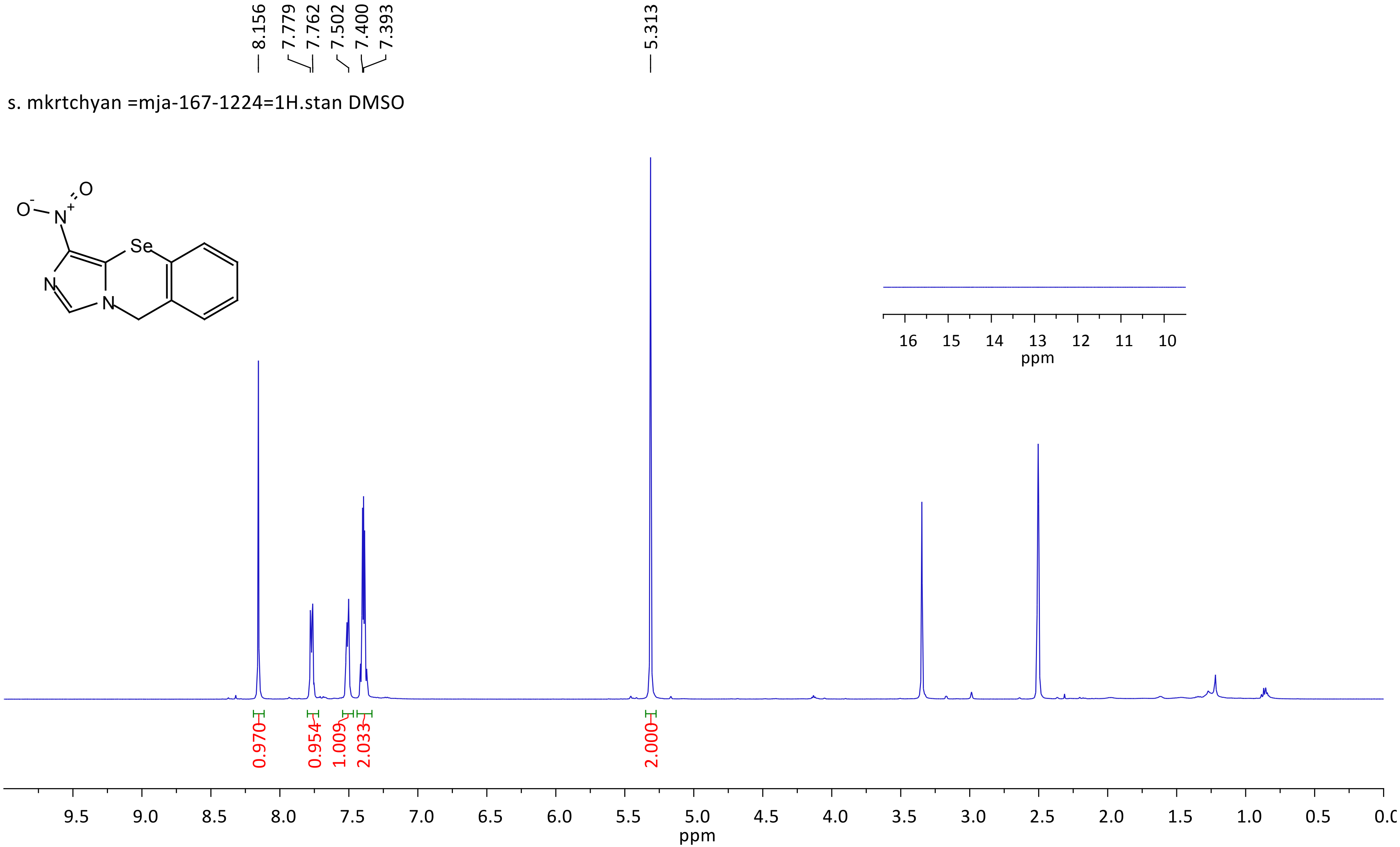

Comment	s. mkrtchyan =mja-167-1224=1H.stan DMSO
Number of Scans	39
Spectrometer Frequency	500.13
Spectral Width	12335.5
Spectral Size	65536

Compound **13b**

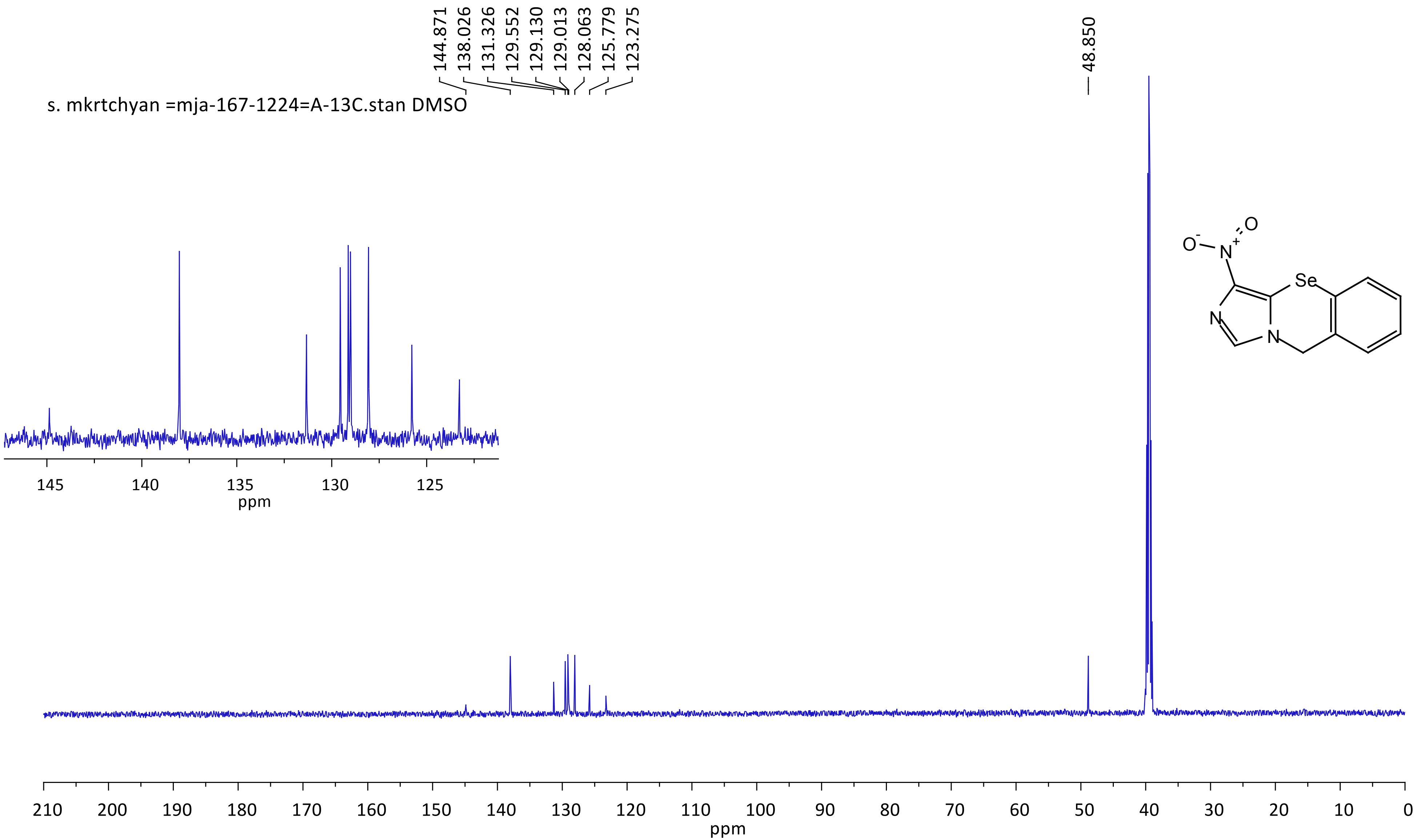

Comment	s. mkrtchyan =mja-167-1224=A-13C.stan DMSO
Number of Scans	512
Spectrometer Frequency	125.76
Spectral Width	36057.7
Spectral Size	65536

Compound **13c**

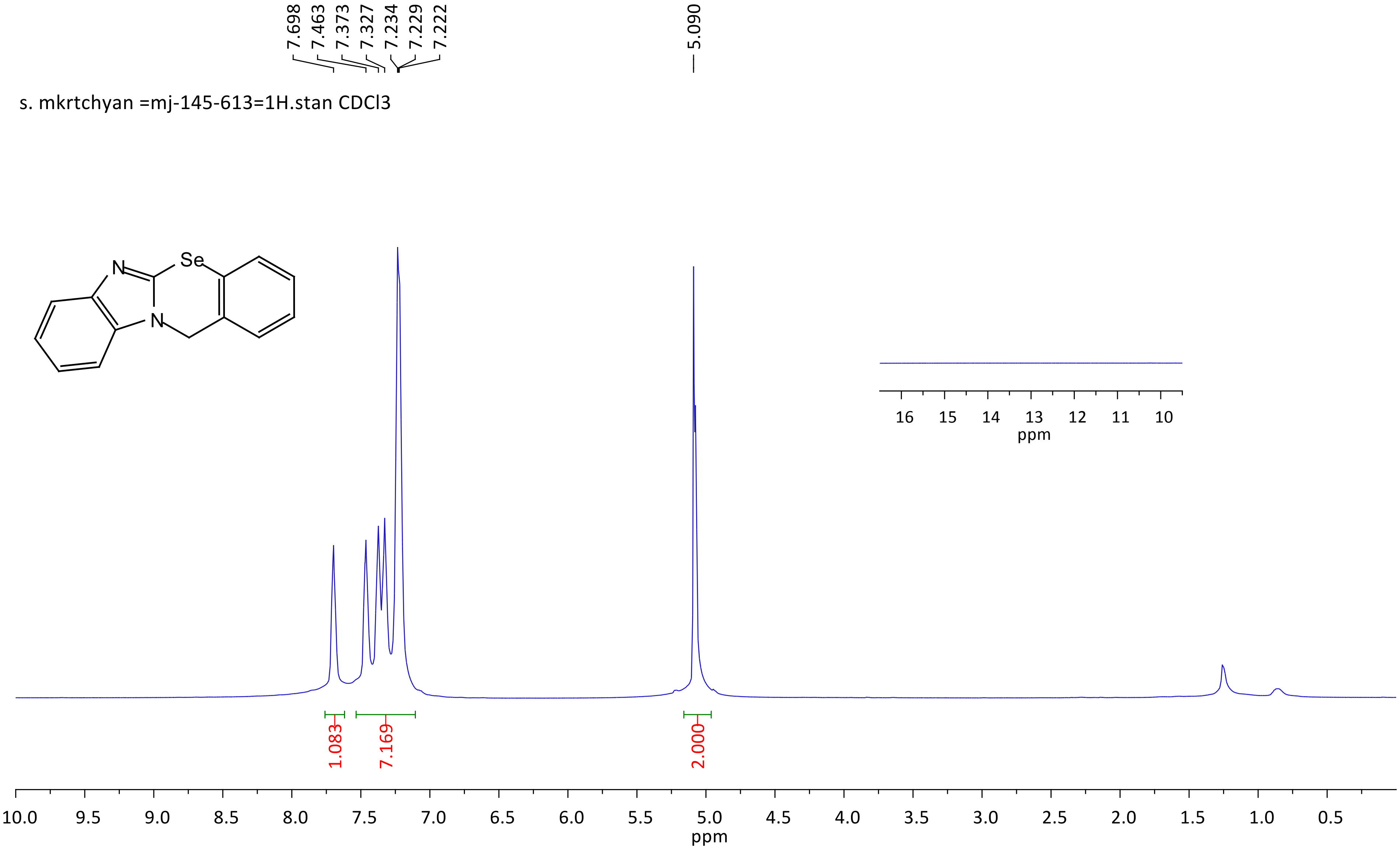

Comment	s. mkrtchyan =mj-145-613=1H.stan CDCl3
Number of Scans	24
Spectrometer Frequency	500.13
Spectral Width	12335.5
Spectral Size	65536

Compound **13c**

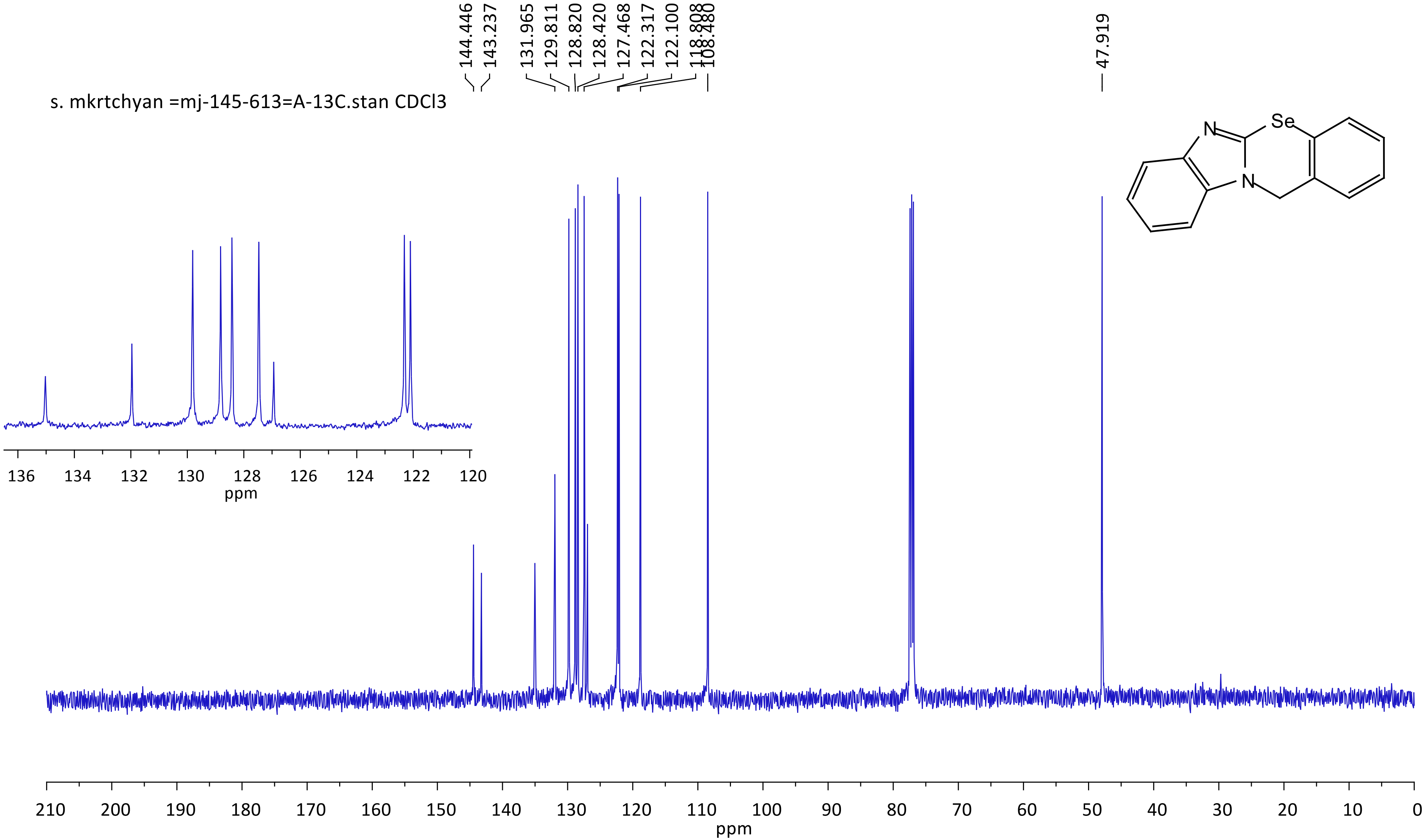

Comment	s. mkrtchyan =mj-145-613=A-13C.stan CDCl3
Number of Scans	512
Spectrometer Frequency	125.76
Spectral Width	36057.7
Spectral Size	65536

Compound **1by**

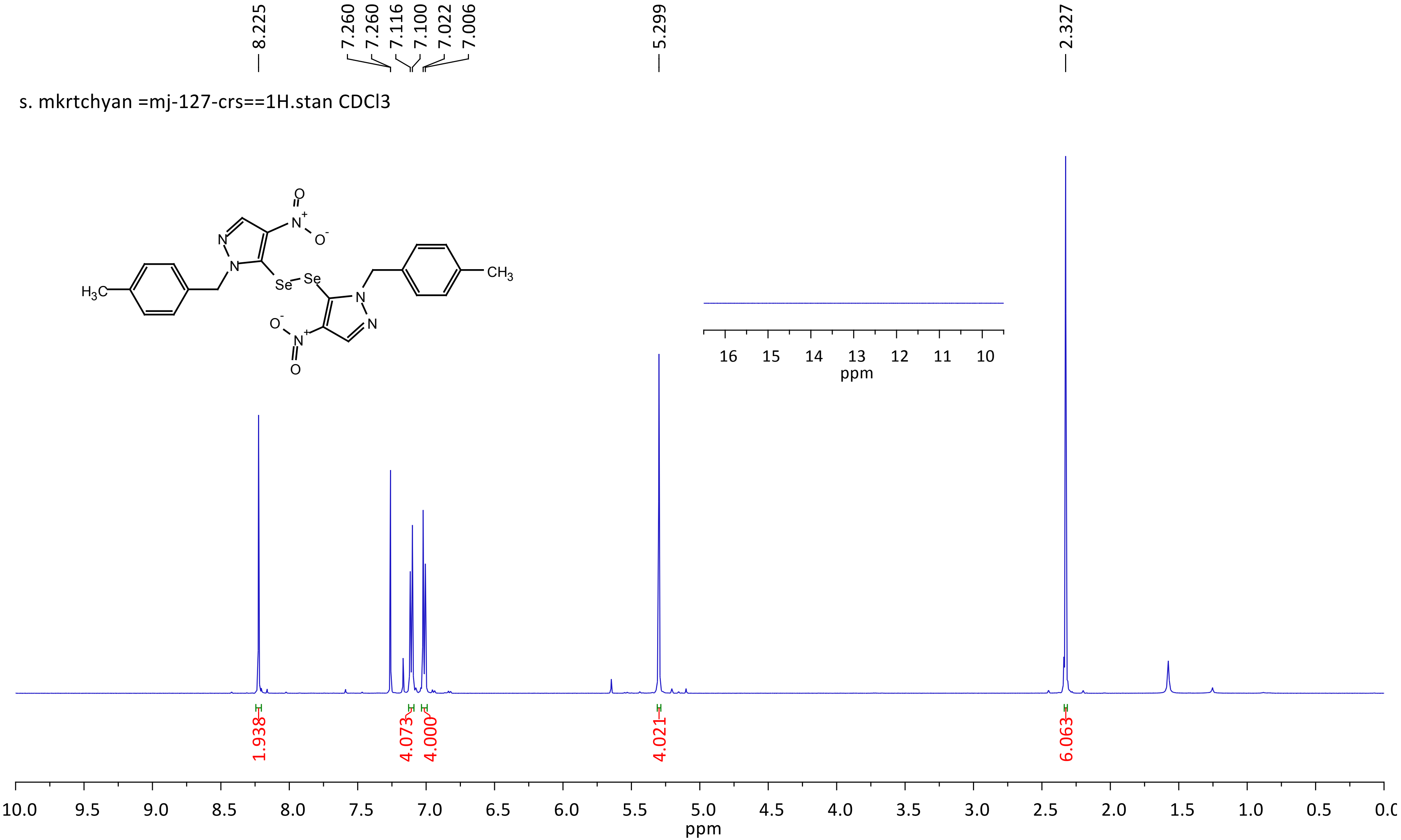

Comment	s. mkrtchyan =mj-127-crs==1H.stan CDCl3
Number of Scans	24
Spectrometer Frequency	500.13
Spectral Width	12335.5
Spectral Size	65536

Compound **1by**

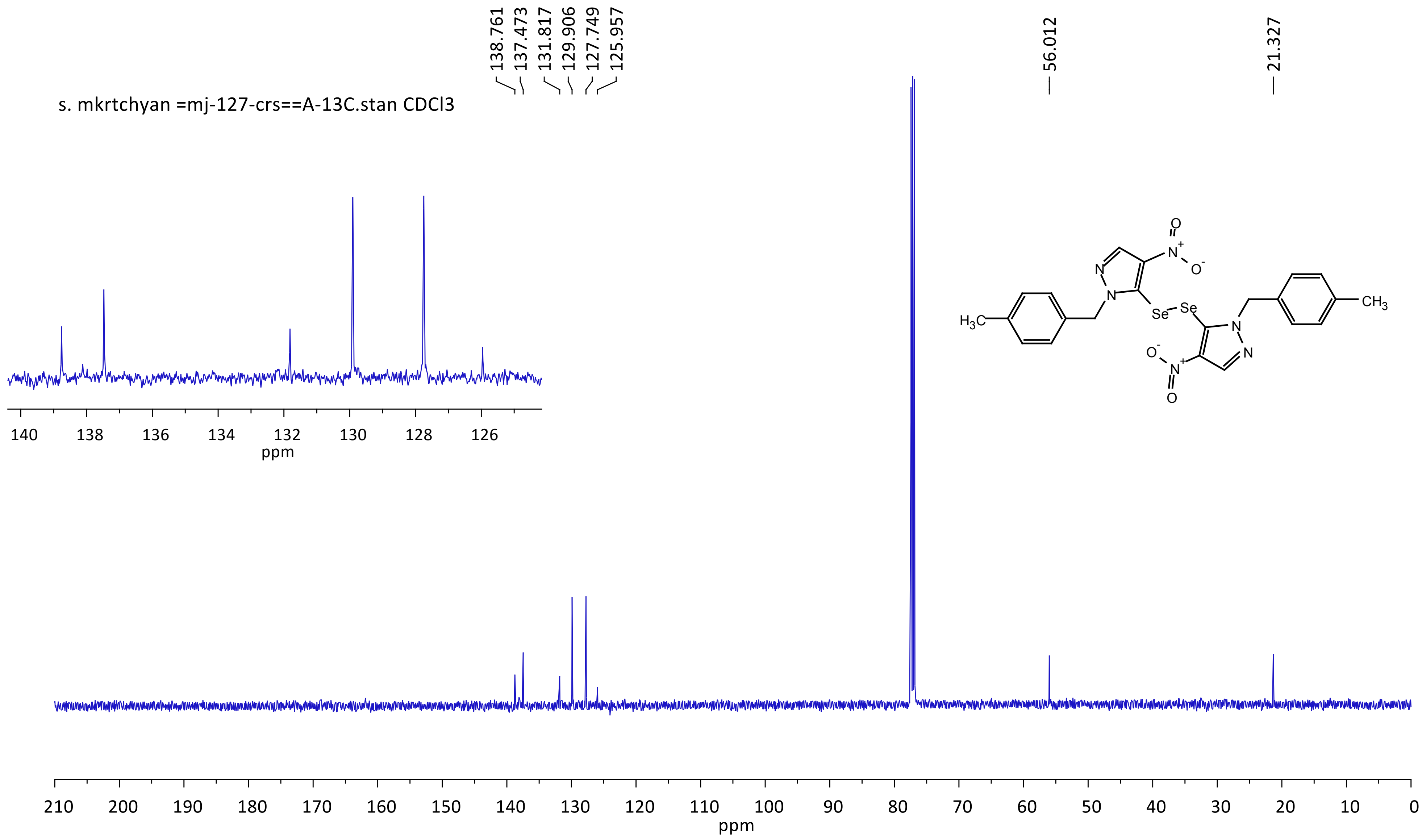

Comment	s. mkrtchyan =mj-127-crs==A-13C.stan CDCl3
Number of Scans	512
Spectrometer Frequency	125.76
Spectral Width	36057.7
Spectral Size	65536

(D) X-ray single crystal analysis of compounds 1c, 2f, 5b, 5d, 5f, 5i, 5k and 5m.

CCDC 1922898-1922905 contain the supplementary crystallographic data for this paper. These data can be obtained free of charge from The Cambridge Crystallographic Data Centre *via* www.ccdc.cam.ac.uk/structures. Figures presenting molecular structures of all structurally determined compounds, tables containing crystals data and data refinement as well as details of X-ray diffraction experiments are given in SI file.

X-ray diffraction experimental details

Data for single crystals suitable for diffraction experiments of the compounds **1c**, **2f**, **5b**, **5d**, **5f**, **5i**, **5k** and **5m** were collected at room temperature on a Gemini A Ultra Diffractometer (Rigaku Oxford Diffraction) with mirror monochromated Mo/Kα radiation (λ = 0.71073 Å). Data collection and data reduction were performed in the CrysAlisPro program [1]. The empirical absorption corrections using spherical harmonics implemented in multi-scan scaling algorithm were applied. To solve and refine the structure OLEX-2 version 1.2 [2] with incorporated SHELXT [3] (direct methods) and SHELXL (the full-matrix least-squares technique) programs [4] was used. All non-hydrogen atoms were refined with anisotropic temperature factors. The H-atoms were placed in calculated positions with fixed isotropic thermal parameters (U_{iso}(H) = 1.2 × [U_{eq}(C)] or 1.5 × [U_{eq}(C)] for aromatic or aliphatic carbon atoms, respectively). In **1c** symptoms of disorder were detected. For the most influenced fragments i.e. selene atom and nitro groups, two positions were considered with free occupancy factors that equalled to 0.79(2) for the major component at the final stage of the refinement. In **5d** the -CF_3 group showed rotational disorder and the final model contains two most probable position of the group with s.o.f. for major component refined to 0.624(14). Crystal data for the investigated compounds are collected in Tables S1a and S1b.

[1] CrysAlisPro 1.171.38.46, Rigaku Oxford Diffraction, 2015.
[2] O.V.Dolomanov, L.J. Bourhis, , R.J. Gildea, J.A.K. Howard, H. Puschmann, , J. Appl. Cryst. 2009, **42**, 339-341. OLEX2: A Complete Structure Solution, Refinement and Analysis Program. http://dx.doi.org/10.1107/S0021889808042726
[3] G. M. Sheldrick, Acta Cryst. 2015, A71, 3–8. SHELXT - Integrated space-group and crystal-structure determination. https://doi.org/10.1107/S2053273314026370
[4] G. M. Sheldrick, Acta Cryst. 2015, C71, 3–8. Crystal structure refinement with SHELXL. https://doi.org/10.1107/S2053229614024218

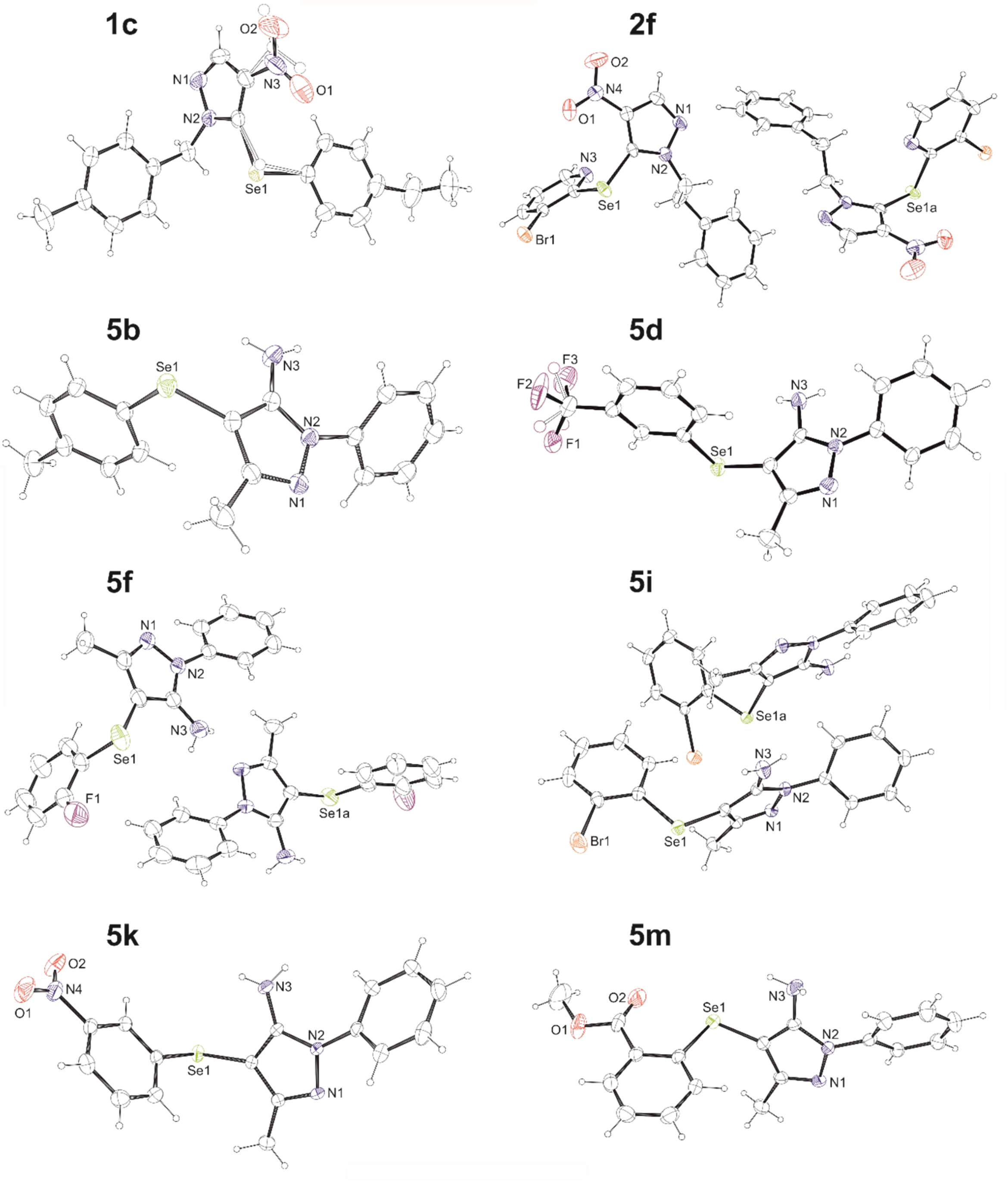
1c
2f
5b
5d
5f
5i
5k
5m
O2
N1
N2
N3
N4
O1
Se1
Se1a
Br1
F1
F2
F3

Figure 1. Ortep[5] drawings of molecular structures. The thermal ellipsoids are drawn with 30% probability. Only the labels for atoms heavier than carbon atom are given. In the cases where two crystallographically molecules were found the labels of the second molecule correspond to the first one and are given with suffix “a”. Minor components of disordered groups are depicted with spheres of arbitrary radius.

Table S1a. Crystal data and data refinement for **1c**, **2f**, **5b** and **5d**

**Compound**	**1c**	**2f**	**5b**	**5d**
CCDC number	1922899	1922903	1922898	1922905
Empirical formula	$C_{19}H_{19}N_3O_2Se$	$C_{16}H_{13}BrN_4O_2Se$	$C_{17}H_{17}N_3Se$	$C_{17}H_{14}F_3N_3Se$
Formula weight	400.33	452.17	342.29	396.27
Temperature/K	293(2)	293(2)	293(2)	293(2)
Crystal system	monoclinic	triclinic	monoclinic	monoclinic
Space group	$P2_1/c$	$P{-}1$	$P2_1/c$	$P2_1/c$
a/Å	4.8207(3)	7.8948(5)	12.5515(5)	5.7537(2)
b/Å	36.9783(15)	7.9369(6)	5.93199(19)	23.2453(8)
c/Å	10.4021(6)	31.3002(16)	21.5896(11)	12.4112(5)
α/°	90	83.404(5)	90	90
β/°	95.860(6)	89.627(5)	101.138(4)	90.937(4)
γ/°	90	60.612(7)	90	90
Volume/Å^3	1844.63(17)	1694.8(2)	1577.19(11)	1659.73(10)
Z	4	4	4	4
ρ_{calc} g/cm^3	1.442	1.772	1.442	1.586
μ/mm^{-1}	2.052	4.590	2.378	2.296
F(000)	816.0	888.0	696.0	792.0
Crystal size/mm^3	0.35 × 0.08 × 0.05	0.5 × 0.3 × 0.3	0.7 × 0.4 × 0.2	0.8 × 0.2 × 0.2
Radiation	MoKα (λ = 0.71073 Å)			
2Θ range for data collection/°	6.612 to 62.856	6.562 to 62.636	6.616 to 54.994	6.566 to 50.992
Index ranges	$-6 \le h \le 7$, $-53 \le k \le 49$, $-14 \le l \le 14$	$-11 \le h \le 10$, $-11 \le k \le 11$, $-44 \le l \le 45$	$-15 \le h \le 16$, $-7 \le k \le 7$, $-27 \le l \le 24$	$-6 \le h \le 6$, $-26 \le k \le 28$, $-15 \le l \le 15$
Reflections collected	25566	24857	14178	9856
Independent reflections	5610 [R_{int} = 0.0730]	9838 [R_{int} = 0.0689]	3579 [R_{int} = 0.0392]	3036 [R_{int} = 0.0249]
Data/restraints/ parameters	5610/21/266	9838/0/433	3579/2/200	3036/103/254
Goodness-of-fit on F^2	1.001	1.006	1.020	1.030
Final *R* indexes [$I > 2\sigma(I)$]	R_1 = 0.0516, wR_2 = 0.0916	R_1 = 0.0604, wR_2 = 0.0631	R_1 = 0.0365, wR_2 = 0.0731	R_1 = 0.0304, wR_2 = 0.0670
Final *R* indexes	R_1 = 0.1563, wR_2 =	R_1 = 0.1642, wR_2 =	R_1 = 0.0596, wR_2 =	R_1 = 0.0408, wR_2 =

[5] L. J. Farrugia, J. Appl. Cryst. 2012, 45, 849–854. WinGX and ORTEP for Windows: an update. https://doi.org/10.1107/S0021889812029111.

[all data]	0.1215	0.0860	0.0819	0.0711
Largest diff. peak/hole / e Å^{-3}	0.28/–0.20	0.68/–0.56	0.37/–0.26	0.39/–0.38

Table S1a. Crystal data and data refinement for **5f**, **5i**, **5k** and **5m**

**Compound**	**5f**	**5i**	**5k**	**5m**
CCDC number	1922901	1922902	1922900	1922904
Empirical formula	$C_{16}H_{14}FN_3Se$	$C_{16}H_{14}BrN_3Se$	$C_{16}H_{14}N_4O_2Se$	$C_{18}H_{17}N_3O_2Se$
Formula weight	346.26	407.17	373.27	386.30
Temperature/K	293(2)	293(2)	293(2)	293(2)
Crystal system	triclinic	monoclinic	monoclinic	orthorhombic
Space group	*P*–1	$P2_1/c$	$P2_1/n$	*Pbca*
a/Å	10.7344(4)	9.4439(4)	8.0441(3)	12.6478(10)
b/Å	11.4734(4)	26.1410(11)	16.3997(6)	10.1017(8)
c/Å	13.5667(5)	12.8829(4)	12.5446(5)	26.833(3)
α/°	92.850(3)	90	90	90
β/°	109.342(3)	91.719(3)	106.994(4)	90
γ/°	97.713(3)	90	90	90
Volume/Å^3	1554.36(10)	3179.0(2)	1582.61(10)	3428.2(5)
Z	4	8	4	8
ρ_{calc} g/cm^3	1.480	1.701	1.567	1.497
μ/mm^{-1}	2.422	4.872	2.387	2.206
F(000)	696.0	1600.0	752.0	1568.0
Crystal size/mm^3	0.42 × 0.36 × 0.28	0.4 × 0.1 × 0.1	0.8 × 0.6 × 0.4	0.28 × 0.26 × 0.08
Radiation	MoKα (λ = 0.71073 Å)			
2Θ range for data collection/°	7.02 to 53.994	6.518 to 52.998	6.792 to 56.484	6.442 to 62.878
Index ranges	$-13 \le h \le 13$, $-14 \le k \le 14$, $-17 \le l \le 17$	$-11 \le h \le 11$, $-32 \le k \le 32$, $-16 \le l \le 16$	$-10 \le h \le 10$, $-21 \le k \le 21$, v16 ≤ $l \le 16$	$-17 \le h \le 17$, $-9 \le k \le 14$, $-28 \le l \le 36$
Reflections collected	35398	33276	29384	24626
Independent reflections	6775 [R_{int} = 0.0432]	6587 [R_{int} = 0.0735]	3906 [R_{int} = 0.0377]	5243 [R_{int} = 0.1042]
Data/restraints/ parameters	6775/4/397	6587/4/397	3906/2/217	5243/0/220
Goodness-of-fit on F^2	1.014	1.020	1.059	0.982
Final *R* indexes [$I > 2\sigma(I)$]	R_1 = 0.0407, wR_2 = 0.0773	R_1 = 0.0403, wR_2 = 0.0647	R_1 = 0.0262, wR_2 = 0.0636	R_1 = 0.0537, wR_2 = 0.0992
Final *R* indexes	R_1 = 0.0830, wR_2 =	R_1 = 0.0859, wR_2 =	R_1 = 0.0336, wR_2	R_1 = 0.1656, wR_2 =

[all data]	0.0932	0.0772	= 0.0669	0.1373
Largest diff. peak/hole / e Å^{-3}	0.59/–0.51	0.42/–0.61	0.26/–0.32	0.65/–0.71
